# Transcriptome analysis of Pará rubber tree (*H. brasiliensis*) seedlings under ethylene stimulation

**DOI:** 10.1186/s12870-021-03196-y

**Published:** 2021-09-13

**Authors:** Yoshimi Nakano, Nobutaka Mitsuda, Kohei Ide, Teppei Mori, Farida Rosana Mira, Syofi Rosmalawati, Norie Watanabe, Kaoru Suzuki

**Affiliations:** 1 Bioproduction Research Institute, National Institute of Advanced Industrial Science and Technology (AIST), Tsukuba, Ibaraki, 305-8566 Japan; 2 Bridgestone Corporation, Kodaira, Tokyo, 187-8531 Japan; 3 Laboratory for Biotechnology, Agency for the Assessment and Application of Technology, Build. 630, Puspiptek area, Serpong, Tangerang, Selatan 15314 Indonesia; 4 Computational Bio Big-Data Open Innovation Laboratory (CBBD-OIL), National Institute of Advanced Industrial Science and Technology (AIST), Tokyo, 169-8555 Japan

**Keywords:** Ethylene, Microarray, Natural rubber, Pará rubber tree, *H. brasiliensis*

## Abstract

**Background:**

Natural rubber (*cis*-1,4-polyioprene, NR) is an indispensable industrial raw material obtained from the Pará rubber tree (*H. brasiliensis*). Natural rubber cannot be replaced by synthetic rubber compounds because of the superior resilience, elasticity, abrasion resistance, efficient heat dispersion, and impact resistance of NR. In NR production, latex is harvested by periodical tapping of the trunk bark. Ethylene enhances and prolongs latex flow and latex regeneration. Ethephon, which is an ethylene-releasing compound, applied to the trunk before tapping usually results in a 1.5- to 2-fold increase in latex yield. However, intense mechanical damage to bark tissues by excessive tapping and/or over-stimulation with ethephon induces severe oxidative stress in laticifer cells, which often causes tapping panel dryness (TPD) syndrome. To enhance NR production without causing TPD, an improved understanding of the molecular mechanism of the ethylene response in the Pará rubber tree is required. Therefore, we investigated gene expression in response to ethephon treatment using Pará rubber tree seedlings as a model system.

**Results:**

After ethephon treatment, 3270 genes showed significant differences in expression compared with the mock treatment. Genes associated with carotenoids, flavonoids, and abscisic acid biosynthesis were significantly upregulated by ethephon treatment, which might contribute to an increase in latex flow. Genes associated with secondary cell wall formation were downregulated, which might be because of the reduced sugar supply. Given that sucrose is an important molecule for NR production, a trade-off may arise between NR production and cell wall formation for plant growth and for wound healing at the tapping panel.

**Conclusions:**

Dynamic changes in gene expression occur specifically in response to ethephon treatment. Certain genes identified may potentially contribute to latex production or TPD suppression. These data provide valuable information to understand the mechanism of ethylene stimulation, and will contribute to improved management practices and/or molecular breeding to attain higher yields of latex from Pará rubber trees.

**Supplementary Information:**

The online version contains supplementary material available at 10.1186/s12870-021-03196-y.

## Background

Natural rubber (*cis*-1,4-polyioprene; NR) is a vitally important industrial raw material because it cannot be replaced by synthetic rubbers on account of the superior resilience, elasticity, abrasion resistance, efficient heat dispersion, and impact resistance of NR [1]. NR accounted for 47.2% of the world rubber production in 2019 (more than 13.6 million tonnes, from Statista web site [2]). Virtually all commercial NR is derived from the Pará rubber tree (*H. brasiliensis*), which is cultivated in tropical and subtropical regions worldwide but predominantly in Southeast Asia. Increased production of NR has been prompted by the increasing global demand for rubber. Given that it is difficult to expand the cultivation area because of competition with production of other important crops and conservation of natural rainforests, further improvement in NR yield per area is desired.

Latex, a rubber-containing cytoplasmic component, is produced in laticifers, which are highly differentiated cells that synthesize and store latex in the inner bark of Pará rubber trees. In NR production, latex is harvested by periodical tapping of the trunk bark. Wounding of the bark caused by tapping induces endogenous ethylene production. Ethylene is a gaseous plant hormone involved in the regulation of diverse biochemical, physiological, and developmental processes in plants. The role of ethylene in defense responses to wounding, herbivory, and pathogen infection has been widely studied in plants [3, 4]. Ethephon, an ethylene releaser, effectively increases the latex yield in Pará rubber trees and ethylene stimulation is commonly practiced in rubber tree plantations worldwide [5]. Ethephon application to the bark enhances and prolongs latex flow and latex regeneration, usually resulting in a 1.5- to 2-fold increase in latex yield [5]. However, intense mechanical damage to bark tissues by excessive tapping and/or over-stimulation with ethephon induces severe oxidative stress in laticifer cells, which often causes the tapping panel dryness (TPD) syndrome in Pará rubber tree. Under TPD, latex flow ceases and in situ coagulation of rubber particles or deep degeneration of tissues are observed [6–9]. The TPD syndrome is characterized by two types of physiological symptoms: a temporary halt in latex flow and histological deformation of the bark. The halt in latex flow is induced by reactive oxygen species (ROS) in laticifer cells and is mitigated during a resting period [10]. Deformation of the bark severely impairs latex flow [11]. Ethylene stimulation is certainly effective to enhance latex production, whereas TPD is undesirable because it leads to severe reduction in latex yield. Susceptibility to TPD is variable and clone dependent; clones that show low latex production may be effectively stimulated by ethylene application and are TPD tolerant, whereas clones that show high latex metabolism are more susceptible to TPD [11]. Therefore, a means of tapping and ethylene stimulation for maximal induction of latex yield without causing TPD is desired. To this end, further studies are required to improve knowledge of the molecular mechanism of the ethylene response in Pará rubber tree.

Several types of approaches, including transcriptome analyses, have been conducted previously to explore the molecular mechanisms involved in responses to ethylene of the Pará rubber tree [11–18]. Transcriptome analysis is a powerful approach to understand gene regulatory mechanisms. However, previous studies have provided only limited information on ethylene-specific events in Pará rubber trees because ethephon was applied to tapping panels of mature trees in a plantation before or after tapping and, therefore, the effects of wounding were not excluded [15, 19]. Thus, an experimental system suitable for analysis of the ethylene-specific response must be established. In the present study, we used young seedlings of Pará rubber tree for ethephon and mock treatments to obtain a time-dependent gene expression profile as a model system. A large number of seedlings of Pará rubber tree can be readily cultivated in a greenhouse and an experimental garden, and can be uniformly treated, in contrast to mature trees in plantations. Dynamic and specific changes in gene expression profiles were revealed in response to ethephon treatment. The study provides valuable information on the biochemical and metabolic responses to ethylene in Pará rubber trees, in addition to the establishment of a model experimental system useful for studying latex biology and rubber biosynthesis.

## Materials and methods

### Plant materials

Pará rubber tree (*H. brasiliensis* clone PB260) seeds were collected in the field of PT. Bridgestone Sumatra Rubber Estate, Serbalawan, North Sumatra, Indonesia. These seeds were germinated and the seedlings cultivated in the experimental greenhouse of the Agency for the Assessment and Application of Technology (BPPT), Serpong, Tangerang Selatan, Indonesia. Approximately 6-week-old seedlings, which were about 50 cm in height, were used for experiments. The stem region 2–5 cm below the shoot apex was swabbed with either 2.5% Ethrel® (containing 24% ethephon; Bayer CropScience, Inc.) or distilled water (mock control) using small brushes at around 09:00 in the greenhouse. At three time points (6, 24, and 48 h) after treatment, the treated stem region (3 cm long) was collected and placed in small plastic bags, immediately frozen in liquid nitrogen, and stored at − 80 °C until used for RNA extraction.

### RNA extraction, reverse transcription (RT), and quantitative RT-PCR

For RNA extraction, the stem segment was ground with a mortar and pestle in liquid nitrogen. The ground sample was homogenized in cetyltrimethylammonium bromide (CTAB) reagent containing 2% (w/v) CTAB, 2.5% (w/v) PVP-40, 100 mM Tris-HCl (pH 7.5), 25 mM EDTA (pH 8.0), 2 M NaCl, and 2% 2-mercaptoethanol, then treated with chloroform:isoamyl alcohol (24:1, v/v). The aqueous phase was incubated with 3 M LiCl at 4 °C overnight, then RNA was precipitated and the solution was centrifuged at 13000 rpm for 20 min at 4 °C. The pellet was purified with the Plant RNeasy Mini Kit (QIAGEN) following the manufacturer’s instructions. To degrade genomic DNA, the RNA was treated with recombinant DNase I (TAKARA Bio). The quality of the RNA was analyzed with an Agilent 2100 Bioanalyzer using the RNA 6000 Nano Kit (Agilent). One microgram of RNA and 2.5 μM oligo(dT) primer were used for reverse transcription with the PrimeScript® RT Reagent Kit (TAKARA Bio). The cDNA was appropriately diluted and used for quantitative RT-PCR analysis with the Power SYBR® Green PCR Master Mix and Applied Biosystems 7300 Real Time PCR System (Thermo Fisher Scientific). Primer sets used for each gene are listed in Table S1.

### Microarray and data analysis

Two hundred nanograms of RNA was used for microarray analysis using the custom microarray for *H. brasiliensis* clone PB260 (8 × 60 K) in accordance with the manufacturer’s instructions (Agilent). Three biological replicates of each sample were analyzed using the one-color method. The total signal value was divided by the median value of all probes for global normalization of the microarray data. The statistical significance of the difference between the ethephon and mock treatments was examined using Welch’s *t*-test. The differentially expressed genes (DEGs) were selected as the genes induced more than 10-fold in the ethephon-treated sample with *P* < 0.01 at either time point. The corresponding *Q* value [20] for each time point was 0.0279, 0.0155, and 0.0506; therefore, we consider the false discovery rate to have been adequately controlled. The collected 3270 genes were classified into 6 groups by *k*-means clustering of Cluster3 software with default setting [21]. For the enrichment analysis, the collected 3270 genes were mapped to non-redundant 1775 Arabidopsis loci by BLASTX search against Arabidopsis TAIR10 coding sequence dataset with the cut-off E-value = 0.00001 (https://www.arabidopsis.org/) because gene annotation information in rubber tree is not sufficient. Then, enrichment of particular Arabidopsis gene ontology term was assessed one by one with a self-made program installed within an in-house database system, which uses the binomial test function of R software (https://www.r-project.org/). The *P* value less than 0.05 was considered as statistically significant. All custom microarray data including platform data has been deposited in Gene Expression Omnibus of NCBI under the accession number GSE174832.

## Results and discussion

### Sample preparation

Gene expression in response to ethylene or ethephon, an ethylene-releasing compound, has been analyzed previously in latex or bark of mature Pará rubber trees subject to tapping [11, 13, 14]. However, ethylene- or ethephon-specific gene expression in virginal mature trees has not been reported. By contrast, Duan et al. (2010) treated 3-month-old young trees with ethylene gas without wounding to analyze the expression of a set of 25 selected genes [12]. In the present study, we used approximately 6-week-old seedlings to examine changes in gene expression profiles in response to ethylene treatment by means of microarray analysis. Rubber tree stems were swabbed with ethephon or water (mock control). At 6 and 24 h after treatment, no visible change was observed between the treatments, whereas some leaves had become yellow and abscised at 48 h after ethephon treatment. This response was interpreted as ethephon-induced senescence and is a typical ethylene response in plants [22]. At 6, 24, and 48 h after treatment, the treated stem segments were harvested for microarray analysis. The amount of latex exudate from the ethephon-treated stems was much higher than that of the mock control (data not shown). Taken together, these results suggested that the experimental system successfully mimicked, at least in part, the physiological and biochemical responses to ethylene stimulation.

### Differentially expressed gene set

**Fig. 1:**
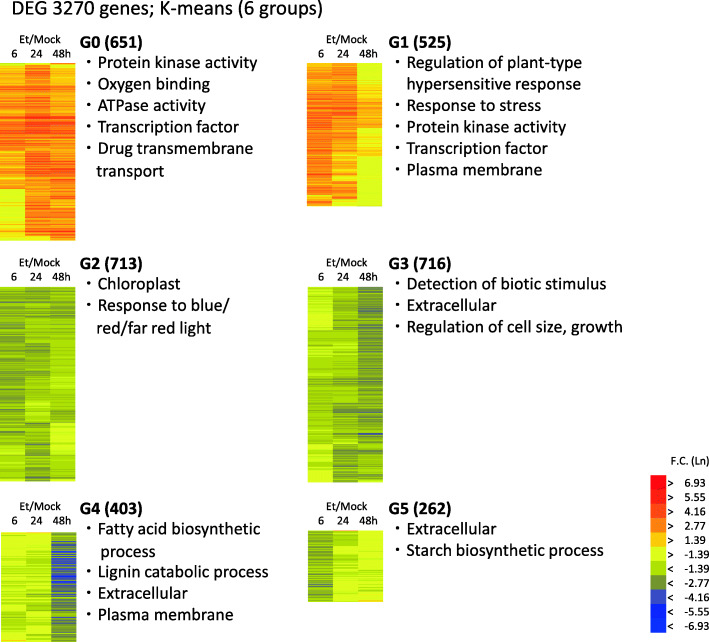
Heat map for *K*-means-clustered differentially expressed genes (DEG). Genes that showed a significant difference in expression after ethephon treatment (6, 24, and 48 h), compared with that of the mock control, were selected and classified into six groups (G0 to G5) by *K*-means classification. Each gene set was characterized by enrichment analysis using gene ontology terms and representative over-represented terms are listed in the right side of each heat map. Fold change in gene expression induced by ethephon treatment compared with that of the mock control is indicated as natural logarithm (ln)

In this study, we used a custom microarray that contained 61,657 probes corresponding to each transcript, of which 41,656 showed sequence homology to Arabidopsis genes. We compared the expression level between the ethephon and mock treatments at each sampling time point to assess changes in the gene expression profile in response to ethephon treatment. As a result, 3270 genes showed a significant difference in expression between the ethephon and mock treatments (|fold change| ≥ 10 at any time point; *p* < 0.01). To characterize the DEGs, we categorized the genes into six groups (groups G0 to G5) using *K-*means clustering and performed enrichment analysis using gene ontology terms (Fig. 1, Table S2). Approximately one-third of the DEGs were upregulated after ethephon treatment and were categorized as groups G0 or G1. Genes in G0 showed continuous upregulation from 6 or 24 h to 48 h after ethephon treatment, whereas genes in G1 showed upregulation from 6 h but expression subsequently declined almost to the control level at 48 h. Genes for protein kinases, stress responses, and transcription factors were enriched in these groups. Genes that showed continuous downregulation until the end of the experimental period were categorized as G2 or G3. These groups contained many genes associated with chloroplasts and involved in cell growth. Genes that showed striking downregulation at 48 h after treatment were categorized as G4. Genes involved in secondary cell wall biosynthesis or cuticle development were enriched in this group. The smallest group was G5. Genes in this group showed downregulation immediately after ethephon treatment, and thereafter expression declined almost to the control level at 24 or 48 h after treatment. Genes involved in amino acid transport and metabolic processes were enriched in G5. We examined the expression of the DEGs more extensively in the following analyses.

### Rubber biosynthesis genes

**Fig. 2:**
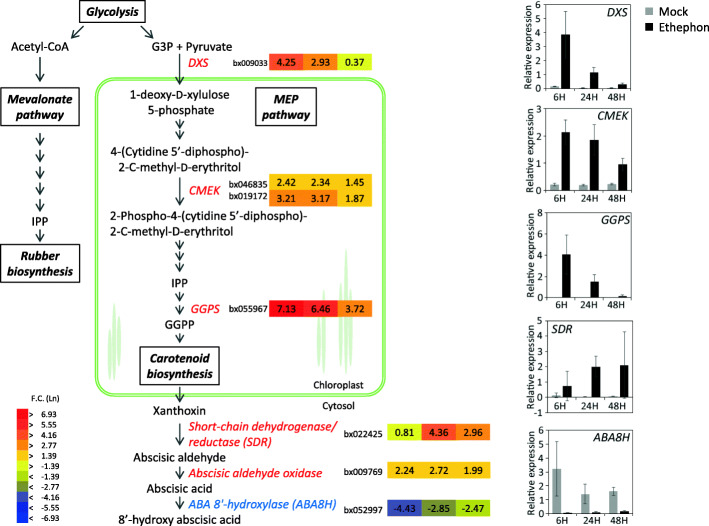
Differentially expressed genes (DEGs) involved in the MEP pathway and ABA biosynthesis. Fold change in gene expression for DEGs involved in the MEP pathway and ABA biosynthesis is displayed as color bars (from left to right; 6, 24, and 48 h after ethephon treatment). Graphs show actual gene expression for these genes, which was confirmed by quantitative RT-PCR analysis. Gray and black bars represent the expression level in mock and ethephon samples, respectively

Natural rubber is predominantly composed of *cis*-1,4-polyisoprene, which is a polymer of an isoprenyl unit derived from isopentenyl diphosphate (IPP). IPP is synthesized by the cytosolic mevalonate (MVA) pathway and the plastidic 2-*C*-methyl-D-erythritol 4-phosphate (MEP) pathway in plants [23]. The former pathway is considered to mainly supply IPP for rubber biosynthesis in Pará rubber tree [18, 24, 25]. Ethephon treatment is reported not to stimulate the MVA pathway and therefore the contribution of this pathway to the increase in yield in response to ethephon treatment would be minimal [5, 26]. As expected, no gene encoding an enzyme involved in the MVA pathway was included among the DEGs. Certain genes, including 3-hydroxy-3-methylglutaryl coenzyme A synthase reductase, were slightly decreased by ethephon treatment, which is consistent with a previous report [16]. In the MEP pathway, however, genes encoding 1-deoxy-D-xylolose 5-phosphate synthase (DXS) and 4-(cytidine 5′-diphospho)-2-*C*-methyl-D-erythritol kinase (CMEK) were significantly upregulated by ethephon treatment (Fig. 2). The MEP pathway is considered to contribute to the supply of IPP for carotenoids biosynthesis [24, 27] and DXS plays an important role in this process [28]. Upregulation of *DXS* and *CMEK* was observed in rubber trees suffering from TPD, under which ROS are accumulated [17]. Carotenoids are believed to act as antioxidants and have been proposed to be sensors or signals of oxidative stress induced by ROS [27]. The upregulation of genes in the MEP pathway in response to ethephon treatment may be for production of carotenoids to scavenge ROS. In addition, plants produce other antioxidants and control the ROS-scavenging system to maintain redox homeostasis [29]. The present microarray data revealed the downregulation of superoxide dismutase (SOD), and the phi and theta families of glutathione *S*-transferase (GST) genes (Table 1). To overcome oxidative stress, GSTs are important and should be abundant. Thus, genes that encode members of the plant-specific tau family of GSTs were highly induced in response to ethephon treatment (Table 1). The tau family of GSTs is considered to be involved in abiotic stress responses and overexpression of these genes confers salt and osmotic tolerance [30, 31]. These results suggest that specific pathways for oxidative stress tolerance were induced in response to ethephon treatment.

**Table 1:** DEGs of SOD and GST family

G^a^	Gene ID	Arabidopsis homologue		Et/Mock (Ln)		
		Locus	Short description	6 h	24 h	48 h
***SOD; superoxide dismutase from SALAD***						
2	bx020280	AT1G08830	CSD1	−3.44	−1.75	− 3.72
3	bx066811	AT1G08830	CSD1	−1.08	−3.23	− 3.10
***Glutathione S-transferase family from TAIR***						
***Phi family***						
3	bx063742	AT3G03190	ATGSTF6, ATGSTF11	−1.09	−1.67	−2.51
***Tau family***						
0	bx026931	AT2G29420	ATGSTU7	3.66	5.29	2.77
1	bx031189	AT2G29420	ATGSTU7	5.78	6.35	3.88
1	bx037937	AT2G29420	ATGSTU7	1.87	3.27	0.26
0	bx038747	AT2G29420	ATGSTU7	4.03	5.20	4.91
4	bx045475	AT2G29420	ATGSTU7	−0.88	−0.95	−2.38
1	bx038733	AT3G09270	ATGSTU8	3.12	3.70	0.98
1	bx001704	AT1G78380	ATGSTU19	6.10	5.91	4.14
2	bx019040	AT1G17180	ATGSTU25	−2.66	−2.13	−2.79
***Theta family***						
5	bx012945	AT5G41210	ATGSTT1	−3.78	−2.51	−0.97

^a^Clustering group

Polymerization of IPP derived from MVA into *cis*-1,4-polyisoprene is catalyzed by *cis*-prenyltransferases (CPTs). It has been demonstrated that CPTs are localized on the surface of rubber particles [32]. In addition, two important protein groups for rubber biosynthesis are localized on the surface of rubber particles: small rubber particle proteins (SRPPs) and rubber elongation factors (REFs) [33]. Two rubber tree genomes have been sequenced to date (Reyan7–33-97 in Tang et al. 2016; RRIM 600 in Lau et al. 2016) [18, 25], and all members of each protein group have been revealed [18, 25]. We first confirmed that the probe sets corresponding to these genes were included in the present microarray because we designed the probe sets based on the comprehensive cDNA sequencing data derived from rubber tree clone PB260, which differs from the clones used for genome-sequencing projects. In our probe set, probes corresponding to CPT1, CPT2, CPT9, CPT11, REF2, REF4, REF6, and SRPP10 were not included because the expression levels of these genes were relatively low [25]. We observed that only one gene for *SRPP4* [25] was induced by ethephon treatment, whereas no other genes that encode CPT, SRPP, or REF proteins were detected among the DEGs (Table S3). The basal expression level of *SRPP4* was low and laticifer-specific genes (*REF1*, *REF3*, *REF7*, and *SRPP1*), for which transcripts were abundant in latex and accounted for 96.8% of the expression of *REF*/*SRPP* genes in latex [25], were not induced by ethephon treatment. Taken together, the results suggested that ethephon treatment might have little direct effect on rubber biosynthesis, which is consistent with previous reports [5, 19, 25].

### Ethylene biosynthesis and signaling

In plants, ethylene induces expression of genes involved in ethylene biosynthesis. For instance, expression of the 1-aminocyclopropane-1-carboxylic acid (ACC) synthase gene is reported to be regulated by a positive feedback mechanism [34]. The present microarray data revealed the upregulation of genes for *S*-adenosyl-methionine synthetase and ACC synthase after ethephon treatment, and that expression of these genes returned to the control level at 48 h after treatment. The present data supported the claim for positive feedback regulation in ethylene biosynthesis after ethephon treatment (Table 2).

**Table 2:** DEGs of ERF family and ethylene biosynthesis

G^a^	Gene ID	Arabidopsis homologue			Et/Mock (Ln)		
		Locus	Short description		6 h	24 h	48 h
***ERF family***			**ERF group**^b^	**ERF No**^b^			
2	bx047672	AT2G44940	IIId	ERF34	−3.57	−3.48	−1.37
2	bx024041	AT5G11590	IIIe	ERF41	−2.40	−2.48	−2.27
3	bx038544	AT5G25190	Va	ERF3	−2.21	−1.39	− 2.65
3	bx046468	AT5G25190	Va	ERF3	−1.20	− 1.38	−2.49
0	bx023675	AT4G11140	VI	ERF63	2.35	2.94	2.71
0	bx017330	AT3G16770	VII	ERF72	1.83	2.48	2.47
0	bx022445	AT3G16770	VII	ERF72	2.81	3.05	2.14
0	bx020251	AT3G15210	VIIIa	ERF78	2.18	2.32	1.96
0	bx039197	AT4G17500	IXa	ERF100	5.74	5.22	4.50
0	bx039254	AT4G17500	IXa	ERF100	2.03	2.69	1.64
0	bx046661	AT4G17500	IXa	ERF100	3.80	6.04	4.57
1	bx046659	AT5G51190	IXb	ERF105	2.69	2.70	1.40
1	bx071140	AT3G23230	IXc	ERF98	5.86	5.63	4.11
0	bx038100	AT3G23240	IXc	ERF92	5.92	7.62	7.16
0	bx040001	AT3G23240	IXc	ERF92	3.81	7.49	8.04
0	bx058590	AT3G23240	IXc	ERF92	4.22	3.35	4.40
1	bx027059	AT3G23240	IXc	ERF92	5.24	4.95	3.18
0	bx011862	AT5G43410	IXc	ERF96	4.48	5.01	4.10
1	bx031374	AT5G50080	Xa	ERF110	4.36	3.87	2.47
0	bx037951	AT5G61890	Xa	ERF114	1.90	3.62	3.89
3	bx059815	AT4G37750	–	–	−2.35	−2.46	−3.53
3	bx007515	AT5G10510	–	–	− 2.37	−2.32	−3.60
***Ethylene biosynthesis gene***							
1	bx000299	AT4G01850	SAMS2		2.69	2.50	0.14
1	bx063180	AT3G61510	ACS1		3.19	2.62	0.09
1	bx040584	AT4G11280	ACS6		3.27	3.66	−0.41

^a^Clustering group

^b^ERF group and No. from Nakano et al. 2006

### ABA biosynthesis

The present results indicated that the MEP pathway is activated and supplied IPP for carotenoids biosynthesis in response to ethephon treatment, and that these carotenoids may contribute to protection from ROS. Abscisic acid (ABA) is derived from carotenoids [27]. We observed that ethephon treatment induced the expression of genes involved in ABA biosynthesis and repressed expression of genes that participate in ABA catabolism (Fig. 2), which suggests de novo ABA biosynthesis and accumulation. Previous studies have demonstrated the induction of ABA biosynthesis by ethylene [35, 36]. Thus, carotenoids production induced by ethephon treatment would supply not only antioxidants but also precursors of ABA. It is well known that ABA induces leaf abscission and ethylene accelerates this process in the presence of ABA [37, 38]. We observed that some leaves abscised at 48 h after ethephon treatment (data not shown), which might be initiated by the induced ABA.

### Transporters

Abscisic acid is synthesized in response to drought stress and can induce stomatal closure to prevent water loss by transpiration. Previous experiments have shown that ABA signaling induces certain aquaporins, which results in increased hydraulic conductivity and water potential [39–42]. A remarkable response to ethephon treatment is the prolonged flow of latex. The prolonged latex flow may be because water influx into laticifer cells mediated by aquaporins is increased [14, 43–45]. In the present study, expression of genes that encode plasma membrane intrinsic protein (PIP) 1;2 and 1;5 is induced, whereas the expression of PIP2;7 and tonoplast intrinsic protein (TIP) 1;1 is decreased by ethephon treatment (Table 3). PIPs import water into laticifer cells, thus the turgor pressure is increased in laticifer cells [44], whereas TIPs maintain the stability of lutoid membranes and/or cell osmotic balance [46]. The present findings suggested that water status may be dramatically changed in the cells in response to ethephon treatment.

**Table 3:** DEGs of transporters

G^a^	Gene ID	Arabidopsis homologue		Et/Mock (Ln)		
		Locus	Short description	6H	24H	48H
***Aquaporin***						
0	bx000682	AT2G45960	PIP1;2	0.69	2.50	2.64
0	bx000130	AT2G45960	PIP1;2	0.76	3.43	1.91
0	bx013984	AT2G45960	PIP1;2	0.60	3.33	1.80
0	bx081354	AT4G23400	PIP1;5	0.98	2.42	2.86
5	bx005180	AT4G35100	PIP2;7	−2.65	−2.61	−0.06
5	bx071027	AT4G35100	PIP2;7	−2.64	− 2.58	− 0.04
5	bx013710	At2g36830	TIP1;1	−3.17	−1.19	−1.00
5	bx000052	At2g36830	TIP1;1	−3.63	−1.28	−1.25
5	bx079866	At2g36830	TIP1;1	−3.33	−1.30	− 1.11
***Monosaccharide transporter***						
***Monosaccharide (hexoses / pentoses)-H+ symporter family (plasma membrane)***						
4	bx001787	AT1G11260	AtSTP1	−0.25	−0.39	−2.77
1	bx025866	AT5G61520	AtSTP3	1.85	2.64	1.17
1	bx012963	AT5G61520	AtSTP3	2.52	1.96	0.72
0	bx006914	AT5G26250	AtSTP8	1.85	0.26	2.76
1	bx027564	AT5G26340	AtSTP13	5.35	5.31	2.58
2	bx055460	AT1G77210	AtSTP14	−3.04	−3.23	−2.81
***H + -Symporter family for polyols and monosaccharides (plasma membrane)***						
0	bx046600	AT2G18480	AtPLT3	6.25	6.26	6.34
***Putative monosaccharide transporter family (ERD-group = induced by early dehydration)***						
0	bx021636	AT2G48020	Major facilitator superfamily protein	2.01	2.93	2.45
0	bx037209	AT2G48020	Major facilitator superfamily protein	2.01	2.98	2.49
2	bx019596	AT3G05160	Major facilitator superfamily protein	−1.84	−2.66	−1.41
***Sucrose-proton symporter, SUS, SWEET family***						
4	bx012374	AT1G22710	SUT1	−0.83	−1.54	−2.59
4	bx013239	AT1G22710	SUT1	−0.83	−1.30	−2.41
5	bx049225	AT5G50790	SWEET10	−3.89	−1.96	0.16
5	bx019194	AT5G50790	SWEET10	−4.13	−1.95	0.18
2	bx038944	AT5G13170	SWEET15	−2.05	−2.70	−0.85
2	bx055911	AT4G15920	SWEET17	−2.49	−1.72	−1.35
***ABC transporter***						
4	bx013345	AT2G36910	AtABCB1	−0.93	−1.00	−2.56
3	bx063540	AT4G18050	AtABCB9	−0.91	−1.89	−2.62
5	bx008871	AT3G28345	AtABCB15	−2.65	−0.18	−1.20
5	bx047965	AT3G28345	AtABCB15	−2.62	−0.08	−1.16
0	bx059322	AT3G13080	AtABCC3	3.02	2.49	2.46
0	bx053101	AT2G47800	AtABCC4	2.06	2.47	1.64
1	bx013696	AT2G47800	AtABCC4	2.71	2.83	1.75
1	bx050933	AT3G21250	AtABCC8	2.74	2.29	2.02
0	bx002770	AT3G21250	AtABCC8	3.43	3.44	2.73
0	bx029139	AT3G21250	AtABCC8	3.39	3.38	2.67
0	bx014388	AT3G21250	AtABCC8	3.21	2.77	2.44
0	bx072313	AT3G21250	AtABCC8	2.98	2.73	2.44
1	bx049452	AT3G21250	AtABCC8	3.18	2.82	2.32
0	bx043154	AT3G21250	AtABCC8	2.81	2.37	2.33
1	bx026865	AT3G21250	AtABCC8	2.85	2.01	2.03
1	bx040637	AT3G21250	AtABCC8	2.84	2.17	1.96
0	bx044179	AT3G21250	AtABCC8	3.09	2.47	2.40
0	bx072464	AT3G21250	AtABCC8	3.22	3.06	2.60
1	bx072417	AT3G60160	AtABCC9	2.35	0.66	0.85
0	bx019866	AT3G59140	AtABCC10	1.92	2.98	2.59
3	bx041286	AT3G21090	ABCG15	−2.75	−3.96	−4.81
1	bx078841	AT3G16340	ABCG29	2.72	0.68	−0.36
1	bx005490	AT1G15210	ABCG35	2.94	1.83	0.50
1	bx081688	AT1G59870	ABCG36	2.96	1.82	0.46
0	bx063709	AT1G15520	ABCG40	1.84	3.02	1.91
5	bx003268	AT1G15520	ABCG40	−2.86	−0.63	−0.25
0	bx008762	AT1G15520	ABCG40	1.89	2.96	1.81
0	bx052699	AT1G15520	ABCG40	1.72	2.94	1.96
5	bx039067	AT1G15520	ABCG40	−2.69	−0.65	−1.51

^a^Clustering group

Sucrose is an important molecule for rubber production because it is the unique precursor of IPP and is expected to be imported into laticifer cells from surrounding cells. Previous studies indicate that expression of sugar transporters is induced by ethylene, which contributes to high latex production [47–49]. Six genes encoding sucrose transporters (SUTs) have been cloned from Pará rubber tree [47–49]. We observed that the probe sets shared high nucleotide sequence identities (~ 99%) with these genes. Although previous studies have reported upregulation of certain *SUT* genes by ethylene [47–49], the present microarray analysis detected downregulation of two such genes (Table 3). Moreover, several genes encoding glucose and/or sucrose efflux transporter family proteins (SWEET) were downregulated (Table 3). In addition to these transporters, genes encoding a sugar transport protein (STP) and one polyol transporter (PLT) were upregulated by ethephon treatment. The STPs are monosaccharide/H^+^ symporters and uptake hexoses from the apoplastic space into cells through the plasma membrane [50]. Sucrose is the predominant molecule utilized for carbon partitioning between sources and sinks in higher plants, and is transported directly into the sink cells. Sucrose is otherwise cleaved into glucose and fructose by cell wall-type invertases (INVs), and these monosaccharides are transported into the cell by the STPs [51, 52]. The present microarray data revealed the upregulation of a gene that encodes a cell wall-type INV (Table 3), which suggests possible co-upregulation of STPs and INVs for monosaccharide uptake. Substantially higher and continuous induction of a gene encoding a PLT, which is a polyol/H^+^ symporter localized to the plasma membrane or tonoplast, was observed in response to ethephon treatment (Table 3). Two *HbPLT* genes (*HbPLT1* and *HbPLT2*) have been identified and their expression pattern in response to ethephon has been demonstrated [53], but the present microarray data detected no significant changes in the expression of these genes. These data suggested the possibility of a tissue- and/or age-dependent mechanism for expression of PLT genes in response to ethylene in Pará rubber tree.

**Fig. 3:**
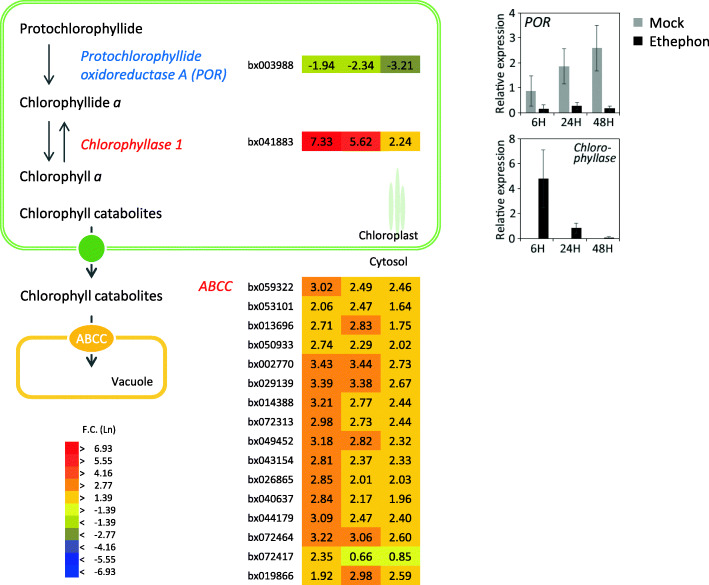
Differentially expressed genes (DEGs) involved in chlorophyll biosynthesis and encoding members of the ABCC transporter family. Fold change in gene expression for DEGs involved in chlorophyll biosynthesis and encoding ABCC transporters is displayed as color bars (from left to right; 6, 24, and 48 h after ethephon treatment). Graphs show actual expression, which was confirmed by quantitative RT-PCR analysis. Gray and black bars represent the expression level in mock and ethephon samples, respectively

The ABC transporters are among the largest protein families in plants and 130 genes have been predicted in the Arabidopsis genome [54]. Given that ABC transporters translocate diverse types of substances, including inorganic compounds, phytohormones, primary products, and lipids, plant ABC transporters are considered to play an important role in plant growth and development, detoxification, response to abiotic stress, and pathogen resistance [54, 55]. Expression of many members of the ABC transporter family is induced in a rubber tree suffering TPD [17]. We detected 46 genes for ABC transporters in the Pará rubber tree transcriptome, of which four are known to be upregulated by ethephon treatment [56]. Although no significant changes in expression of these genes was observed, the present results revealed that expression of other ABC transporters was highly responsive to ethephon treatment of seedlings (Table 3). Several genes encoding ABCB proteins putatively localized in the plasma membrane were downregulated, whereas genes encoding ABCC proteins putatively localized in the vacuolar membrane were upregulated (Table 3). Transporters belonging to the ABCC group transport chlorophyll catabolites into the tonoplast [57, 58]. The present microarray data revealed strong upregulation of a gene encoding chlorophyllase, which catalyzes the first step of chlorophyll degradation, and downregulation (or no induction) of genes involved in photosystem and chlorophyll biosynthesis (Fig. 3, Table 4). Quantitative RT-PCR analysis showed that transcripts corresponding to those of chlorophyllase in all mock samples were below the limit of detection. These data indicated that chlorophyll degradation and suppression of photosynthesis occurred in response to ethephon treatment (Fig. 3). Increased quantities of these chlorophyll catabolites would be transported into the tonoplast by ABCC proteins. The co-regulation of genes encoding ABCC proteins and chlorophyllase may contribute to efficient degradation of chlorophylls during ethylene-induced senescence.

**Table 4:** DEGs of photosystem and chlorophyll biosynthesis

G^a^	Probe ID	Arabidopsis homologue^b^		Et/Mock (Ln)		
		Locus	Short description	6 h	24 h	48 h
5	bx045370	AT5G66190	ferredoxin-NADP(+)-oxidoreductase 1	−3.65	−2.52	− 1.41
2	bx052092	AT4G03280	photosynthetic electron transfer C	−3.06	−2.18	−1.51
2	bx006007	AT4G12800	photosystem I subunit l	−2.88	− 2.06	−1.94
2	bx024863	AT1G08380	photosystem I subunit O	−2.67	−2.67	−1.90
2	bx079126	AT1G08380	photosystem I subunit O	−1.68	−2.47	− 1.53
5	bx022553	AT2G30570	photosystem II reaction center W	−3.24	−2.66	−1.15
2	bx020447	AT3G50820	photosystem II subunit O-2	−2.31	− 2.19	−1.38
3	bx039366	AT2G30790	photosystem II subunit P-2	−2.33	−3.74	−3.22
2	bx049802	AT3G01440	photosystem II oxygen-evolving enhancer protein 3	−4.14	−3.51	− 1.81
3	bx003988	AT5G54190	protochlorophyllide oxidoreductase A	−1.94	−2.34	−3.21
1	bx041883	AT1G19670	Chlorophyllase 1	7.33	5.62	2.24

^a^Clustering group

^b^Genes from KEGG pathway

Several genes encoding ABCG proteins were upregulated or downregulated (Table 3). Arabidopsis ABCG40 (At1g15520), which is involved in ABA uptake [59], is induced by ethylene [60]. Certain rubber genes corresponding to *ABCG40* genes were upregulated by ethephon treatment; this response may be correlated with the upregulation of genes involved in ABA biosynthesis and accumulation (Fig. 2).

### Flavonoid biosynthesis

**Fig. 4:**
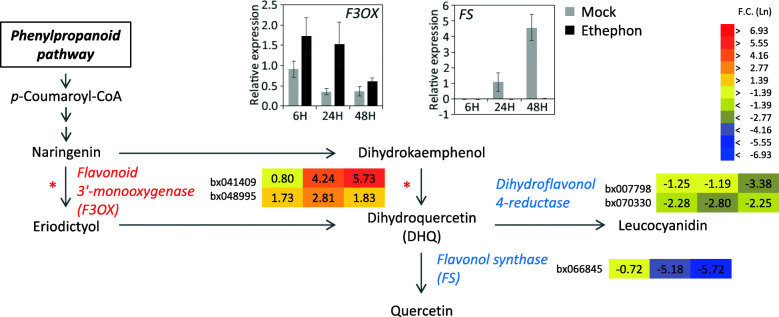
Differentially expressed genes (DEGs) involved in flavonoids biosynthesis. Fold change in gene expression for DEGs involved in flavonoids biosynthesis is displayed as color bars (from left to right; 6, 24, and 48 h after ethephon treatment). Graphs show actual expression, which was confirmed by quantitative RT-PCR analysis. Gray and black bars represent the expression level in mock and ethephon samples, respectively. Asterisks indicate the pathways catalyzed by flavonoid 3′-monooxygenases

In the flavonoid biosynthesis pathway, genes for flavonol synthase and dihydroflavonol 4-reductase were downregulated (Fig. 4). To confirm the microarray data, expression of these genes was examined by quantitative RT-PCR analysis. Expression of flavonol synthase in the mock samples increased with time after the mock treatment, whereas expression was maintained at a low level and showed little increase at 48 h after ethephon treatment. These results suggested that ethephon application may increase dihydroquercetin (DHQ) production. DHQ is a strong antioxidant and increases the stability of the tonoplast membrane, on account of its antioxidant properties, and decreases membrane permeability by suppressing ion channels [61]. Therefore, DHQ may play a role in stabilization of lutoid membranes, which are particularly sensitive to osmotic stress. Water influx into the laticifer cells caused by tapping leads to rupture of lutoids and the release of their contents; subsequently, coagulation of rubber particles and damaged lutoids occurs, which would cause the arrest of latex flow for wound healing and recovery in Pará rubber trees. Thus, lutoid stability influences latex flow [62]. However, inhibition of membrane oxidation by DHQ leads to activation of H^+^-ATPase [61]. In the present microarray analysis, upregulation of several genes encoding H^+^-ATPases was observed (Table 5). Given that tonoplast H^+^-ATPase is activated by ethephon treatment [63], the current results are reasonable. H^+^-ATPase in the lutoid membrane plays an important role by pumping protons from the cytosol into the lutoid during active carbohydrate metabolism to supply carbon sources for latex biosynthesis by maintaining a cell pH suitable for pH-dependent enzymes, such as INV [63, 64]. High-yielding rubber clones show high activity of H^+^-ATPase in lutoids [64]. These responses would be natural mechanisms for defense and recovery from wounding caused by tapping. Taken together, ethephon treatment-induced enhancement of DHQ biosynthesis might be involved in enhanced latex yield via activation of the substrate supply for rubber biosynthesis and the extension of latex exudation by stabilizing lutoid membranes and consequent restraint of coagulation. Based on this hypothesis, excess tapping and/or ethylene stimulation may cause catastrophic changes resulting in TPD.

**Table 5:** DEGs of ATPase family

G^a^	Gene ID	Arabidopsis homologue		Et/Mock (Ln)		
		Locus	Short description	6 h	24 h	48 h
1	bx014049	AT2G24520	H^+^-ATPase 5	3.32	2.37	2.10
1	bx041129	AT2G22950	Autoregulated Ca2 + −ATPase 7	3.60	2.89	0.35
0	bx047876	AT5G57110	Autoinhibited Ca2+ − ATPase8	1.49	1.13	2.97
1	bx038015	AT3G63380	ATPase E1-E2 type family protein	4.82	4.40	2.58
0	bx021597	AT3G01390	Vacuolar membrane ATPase 10	1.71	2.43	1.70

^a^Clustering group

### Transcription factors

During the ethylene response, dynamic changes in the expression of a diverse array of genes occur in plants [65]. Transcription factors control gene transcription in response to various environmental cues and developmental factors. In the present microarray analysis, 2641 probes detected transcription factor genes, of which 236 were among the DEGs (Table S4). Certain transcription factor families, including the ERF, NAC, and WRKY families, are involved in stress responses and ethephon treatment affected expression of these genes.

The ERF family, which is a major constituent of the AP2/ERF superfamily, is a large and plant-specific transcription factor family and members are classified into 10 groups [66]. The microarray data revealed that genes belonging to group IX were highly upregulated by ethephon treatment (Table 2). In addition, genes classified in groups VI, VII, VIII, and X were upregulated, whereas genes of groups III and V were downregulated.

The WRKY transcription factor family is a plant-specific transcription factor family that participates in abiotic/biotic stresses responses, and diverse developmental and physiological processes [67, 68]. Two WRKY transcription factors (WRKY18 and 40) are induced by ABA, and physically and functionally interact under biotic and abiotic stresses in Arabidopsis [69, 70]. In the current study, expression of genes corresponding to WRKY18 and 40 was induced by ethephon treatment (Table 6). This finding is consistent with the gene expression changes suggesting that ABA content might be increased in Pará rubber trees in response to ethephon treatment (Fig. 2). Previous studies of Arabidopsis have shown that several WRKY transcription factors are involved in the promotion (WRKY6, 53, and 75) or delay (WRKY54 and 70) of leaf senescence. *WRKY75* expression is induced in senescent leaves and knockout of *WRKY75* results in a delayed-senescence phenotype in Arabidopsis, which indicates that WRKY75 is a positive regulator of leaf senescence [71]. The present microarray analysis detected upregulation of genes corresponding to WRKY53, 70, and 75. In particular, expression of the gene corresponding to *WRKY75* was highly increased by ethephon treatment and the upregulation was sustained for 48 h (Table 6).

**Table 6:** DEGs of WRKY family

G^a^	Gene ID	Arabidopsis homologue^b^			Et/Mock (Ln)		
		Locus	WRKY group	WRKY No	6 h	24 h	48 h
0	bx007583	AT1G13960	I	WRKY4	2.95	3.80	2.79
0	bx005246	AT1G29860	I	WRKY71	1.40	1.98	2.70
0	bx049005	AT1G29860	I	WRKY71	0.80	2.42	1.26
0	bx051016	AT1G29860	I	WRKY71	1.08	1.72	2.45
1	bx029622	AT2G30250	I	WRKY25	2.85	2.31	1.53
1	bx026110	AT2G38470	I	WRKY33	3.80	3.35	0.50
1	bx026599	AT2G38470	I	WRKY33	2.81	2.34	1.11
1	bx050263	AT2G38470	I	WRKY33	2.76	2.81	1.96
1	bx066153	AT2G38470	I	WRKY33	3.58	2.95	0.15
1	bx081907	AT4G31800	II-a	WRKY18	3.81	3.79	1.60
1	bx023458	AT1G80840	II-a	WRKY40	2.95	3.67	−0.47
1	bx038227	AT1G80840	II-a	WRKY40	5.96	4.82	3.37
1	bx047918	AT1G80840	II-a	WRKY40	3.78	2.80	0.65
1	bx059684	AT1G80840	II-a	WRKY40	3.83	3.77	1.45
0	bx077810	AT4G22070	II-b	WRKY31	1.79	3.12	3.73
0	bx008303	AT1G62300	II-b	WRKY6	2.62	2.78	2.68
1	bx042142	AT1G62300	II-b	WRKY6	2.41	1.88	1.58
0	bx006204	AT5G13080	II-c	WRKY75	5.38	6.97	6.83
0	bx046970	AT5G13080	II-c	WRKY75	4.74	4.91	4.52
0	bx054083	AT5G13080	II-c	WRKY75	6.34	7.10	6.22
0	bx061693	AT5G13080	II-c	WRKY75	5.31	5.97	5.82
1	bx032062	AT5G64810	II-c	WRKY51	4.48	4.55	1.34
2	bx065502	AT5G43290	II-c	WRKY49	−4.24	−2.96	−4.20
2	bx009275	AT5G28650	II-d	WRKY74	−2.78	−1.44	−2.05
1	bx026667	AT4G11070	III	WRKY41	2.80	3.67	0.27
1	bx062508	AT4G23810	III	WRKY53	2.84	2.87	0.01
1	bx002887	AT3G56400	III	WRKY70	2.15	2.95	−0.09
1	bx023296	AT3G56400	III	WRKY70	2.02	2.74	−0.36

^a^Clustering group

^b^WRKY group and number from TAIR

The NAC transcription factor family is a plant-specific transcription factor family that comprises many members. The NAC transcription factors are involved in many developmental processes, including stress response and secondary cell wall formation [72]. Expression of *ANAC029* is induced by leaf senescence and the gene directly regulates *AAO3* expression in Arabidopsis [73, 74]. Enhanced expression of *AAO3* increases ABA content, which may induce chlorophyll degradation during leaf senescence [74]. We observed a similar expression profile in response to ethephon treatment (Figs. 2 and 3, Table 7). Genes encoding NST1 and SND2, which regulate secondary cell wall formation [75], were downregulated following ethephon treatment (Table 7). Their downstream genes, including *Irregular Xylem* (*IRX*), were also downregulated (Table 8). Thus, ethephon treatment may prevent stem growth, including development of xylem and/or fiber cells.

**Table 7:** DEGs of NAC family

G^a^	Gene ID	Arabidopsis homologue^b^			Et/Mock (Ln)		
		Locus	NAC No	Other name	6 h	24 h	48 h
0	bx038862	AT1G69490	ANAC029	NAP	1.24	2.30	3.09
0	bx074452	AT1G69490	ANAC029	NAP	1.12	2.19	2.97
1	bx024871	AT2G17040	ANAC036		2.97	3.35	1.88
1	bx071860	AT5G22380	ANAC090		3.52	1.96	0.51
3	bx064839	AT5G22380	ANAC090		−0.29	−1.86	−2.55
4	bx009169	AT2G46770	ANAC043	NST1	1.12	−2.46	− 2.68
4	bx029117	AT2G46770	ANAC043	NST1	−1.35	−2.06	−4.05
4	bx003373	AT4G28500	ANAC073	SND2	−1.57	−0.69	−3.11
4	bx041479	AT4G28500	ANAC073	SND2	−1.53	−0.91	−4.57
4	bx065762	AT4G28500	ANAC073	SND2	−1.45	− 1.10	−4.37
4	bx065953	AT4G28500	ANAC073	SND2	−1.26	−0.46	−3.48
5	bx026186	AT3G15510	ANAC056		−2.75	−1.62	−1.52
5	bx036393	AT3G15510	ANAC056		−2.41	−1.41	− 1.39
5	bx040482	AT3G15510	ANAC056		−3.06	−0.89	−2.12
5	bx052893	AT3G15510	ANAC056		−2.54	−1.45	−1.36
5	bx070347	AT3G15510	ANAC056		−2.45	−1.38	−1.32

^a^Clustering group

^b^NAC group and number from TAIR

**Table 8:** DEGs of IRX genes

G^a^	Gene ID	Arabidopsis homologue			Et/Mock (Ln)		
		Locus	IRX No	Short description	6 h	24 h	48 h
4	bx048993	AT4G18780	IRX1	CesA8	−1.26	−1.49	−5.17
4	bx059326	AT4G18780	IRX1	CesA8	−1.16	− 1.22	−4.90
4	bx002167	AT5G17420	IRX3	CesA7	−1.11	−1.62	−4.41
4	bx025747	AT5G44030	IRX5	CesA4	−1.56	−2.00	−4.44
4	bx073720	AT5G44030	IRX5	CesA4	−1.69	− 1.72	−5.59
3	bx006479	AT5G15630	IRX6	COBRA-LIKE4	−2.02	−1.82	−2.69
4	bx007065	AT5G15630	IRX6	COBRA-LIKE4	−1.35	−1.66	−5.85
4	bx059178	AT2G37090	IRX9	GT43	−1.11	− 1.61	− 5.64
3	bx073251	AT2G37090	IRX9	GT43	−0.77	−2.40	−3.57
2	bx048506	AT1G27600	IRX9-L	GT43	−3.20	−2.60	−2.58
4	bx004786	AT1G27440	IRX10	GT47	−1.37	−1.40	−5.06
4	bx041075	AT1G27440	IRX10	GT47	−1.69	−1.84	−4.79
4	bx080788	AT1G27440	IRX10	GT47	−1.06	− 1.18	−4.54
4	bx007461	AT2G38080	IRX12	LACCASE 4	−1.83	−1.80	−5.35
4	bx023195	AT2G38080	IRX12	LACCASE 4	−1.13	− 1.47	−5.28
4	bx072438	AT2G38080	IRX12	LACCASE 4	−1.05	−2.22	−5.01
4	bx038921	AT5G03170	IRX13	FLA11	−1.53	−1.39	−5.14
3	bx022936	AT5G67210	IRX15-L	DUF579	−2.50	−2.37	−2.91
4	bx025175	AT5G67210	IRX15-L	DUF579	−1.38	−1.79	−6.14

^a^Clustering group

## Conclusion

**Fig. 5:**
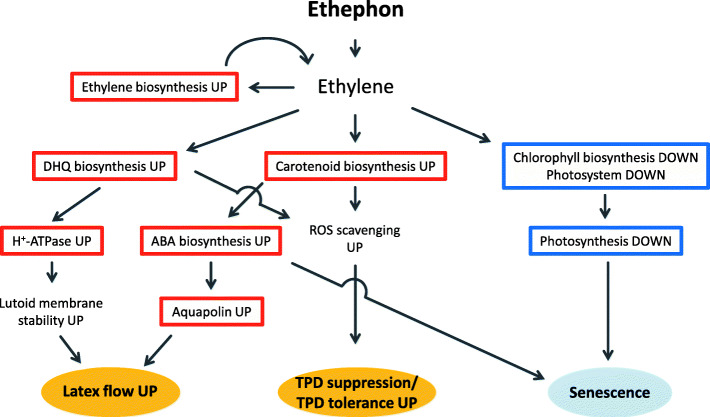
Schematic representation of the Pará rubber tree response to ethephon treatment. The mechanisms deduced from the present results in response to ethephon treatment are summarized. Responses that are directly deduced from microarray data are enclosed in a red box (upregulated) or blue box (downregulated)

The aim of this study was to assess the molecular mechanism of the response to ethylene stimulation in Pará rubber tree with the goal of improving latex production by inhibition of TPD. We established an experimental system using young seedlings and obtained comprehensive transcriptome data during the treatment response. Based on the present results, we propose a schematic model of the events in response to ethephon treatment (Fig. 5). The ethephon treatment induces changes in gene expression associated with carotenoids production. Carotenoids and a downstream product, ABA, may induce expression of PIP genes [27, 38–40], which serve to import water into laticifer cells, resulting in enhanced latex flow. In addition, changes in expression of genes that participate in flavonoids biosynthesis, especially genes associated with DHQ production, were observed. Given that DHQ is believed to contribute to stabilization of lutoid membranes [61], latex exudation would be prolonged, which may suppress coagulation of rubber particles. This series of molecular events may be involved in the increase in latex yield caused by ethylene stimulation. Given that carotenoids and DHQ are known ROS scavengers, ethephon-induced upregulation of genes involved in their biosynthesis may enhance defense responses and contribute to prevention of TPD. It should be noted that ethylene is produced in Pará rubber tree following wounding; therefore, these events may naturally occur during tapping as defense responses or for wound healing.

Ethephon treatment induced chlorophyll degradation, which is the most typical event of leaf senescence [76]. In the present study, some leaves became yellow and abscised at 48 h after ethylene stimulation; thus, ethephon treatment might induce leaf senescence. The induction of many senescence-associated genes is observed in TPD-affected trees subject to excessive tapping or overstimulation with ethylene [9, 77]. Therefore, upregulation of genes involved in chlorophyll degradation might be an indicator of the TPD syndrome derived from overstimulation with ethylene. To prevent chlorophyll degradation and maintain photosynthesis is important for sucrose production, which influences NR biosynthesis.

The present results indicate that ethephon treatment may affect the growth and/or differentiation of cambium and/or laticifer cells. In this study, we observed that genes associated with secondary cell wall formation were highly downregulated at 48 h after ethephon treatment. In woody plants, secondary cell walls are an important carbon sink [78]. Thus, these results suggest that there is a trade-off in carbon supply between rubber biosynthesis and secondary cell wall synthesis after tapping and ethylene stimulation. Otherwise, secondary cell wall formation or biosynthesis might be aberrantly regulated in the secondary phloem because phloem necrosis and abnormal cell layers in the secondary phloem and parenchyma tissues are also observed as symptoms of irreversible TPD [79, 80].

In this study, we have demonstrated that ethephon treatment caused dramatic positive and negative changes in expression of a diverse array of genes involved in metabolism, defense responses, growth, and development. It is suggested that ethephon treatment induced the changes negatively associated with rubber production and regulation of the trade-off between rubber production and other events, in addition to the changes positively associated with rubber production. These changes may cause induction of TPD, which can be triggered by excess tapping and/or ethylene stimulation. The results provide valuable information to understand the mechanism of ethylene stimulation, and will contribute to improved management practices and/or molecular breeding to attain higher yields of latex from Pará rubber trees.

## Supplementary Information

**Additional file 1 **: **Table S1.** Primer sets used for quantitative RT-PCR analysis.

**Additional file 2 **: **Table S2.** Probe list and fold change of 3270 genes that showed a significant difference in expression between the ethephon and mock treatments.

**Additional file 3 **: **Table S3.** Corresponding probe sets for *CPT*, *REF*, and *SRPP* genes.

**Additional file 4 **: **Table S4.** Probe lists for 234 differentially expressed genes encoding transcription factors.

## Data Availability

All data analyzed in this study is available upon request. All custom microarray data including platform data has been deposited in Gene Expression Omnibus of NCBI under the accession number GSE174832.

## Abbreviations

ABA: Abscisic acid
ACC: 1-aminocyclopropane-1-carboxylic acid
CMEK: 4-(cytidine 5′-diphospho)-2-*C*-methyl-D-erythritol kinase
CPT: *cis*-prenyltransferase
DEG: Differentially expressed gene
DHQ: Dihydroquercetin
DSX: 1-deoxy-D-xylolose 5-phosphate synthase
GGPPS: Geranylgeranyl diphosphate synthase
GST: Glutathione *S*-transferase
INV: Invertase
IPP: Isopentenyl diphosphate
MEP: 2-*C*-methyl-D-erythritol 4-phosphate
MVA: Mevalonate
NR: Natural rubber
PIP: Plasma membrane intrinsic protein
PLT: Polyol transporter
REF: Rubber elongation factor
ROS: Reactive oxygen species
SOD: Superoxide dismutase
SRPP: Small rubber particle protein
STP: Sugar transport protein
SUT: Sucrose transporter
TIP: Tonoplast intrinsic protein
TPD: Tapping panel dryness
