# Supplementary material for: Transcriptome analysis of Pará rubber tree (H. brasiliensis) seedlings under ethylene stimulation
Source: BMC Plant Biol. 2021 Sep 13;21:420. doi: 10.1186/s12870-021-03196-y (PMC8436496; doi:10.1186/s12870-021-03196-y)
Supplement: Supplementary file 1 — Additional file 1 : Table S1. Primer sets used for quantitative RT-PCR analysis. [file 12870_2021_3196_MOESM1_ESM.docx]

| Nakano et al. Supplemental Table 1. | | |  |  |
| --- | --- | --- | --- | --- |
| **Primer Name** | **Target** | **Target description** | **Seq** | **Amplicon length** |
| HbABA8H_qF | bx052997 | abscisic acid 8'-hydroxylase (ABA8H) | atgcttgtcctggcaatgaactc | 153 bp |
| HbABA8H_qR |  |  | aaaatctggctggtaatccttgct |  |
| HbChlase1_qF2 | bx041883 | Chlorophyllase 1 | taacggacatagttgggagattgac | 132 bp |
| HbChlase1_qR2 |  |  | ctccgctttcaccttcaaaataaac |  |
| HbCMEK_qF | bx046835 | CMEK | tactttgtgccgatggtagttttgt | 149 bp |
| HbCMEK_qR |  |  | ttgcaaagtgaacaattcgtcg |  |
| HbDXS_qF | bx009033 | DXS | ctctaccttctagcaaatgggtcgt | 144 bp |
| HbDXS_qR |  |  | gcagggaaagatttatcctgccta |  |
| HbF3OX_qF3 | bx048995 | flavonoid 3'-monooxygenase (F3OX) | ttttaagtggaaattgccaggagac | 140 bp |
| HbF3OX_qR3 |  |  | atcacagagagtaaacatgggatgg |  |
| HbFS_qF | bx066845 | flavonol synthase (FS) | ttccaaataatcaaccatggcatac | 145 bp |
| HbFS_qR |  |  | gaagagatgttccatagccttccat |  |
| HbGGPS_qF | bx055967 | GGPS | atccatcaatgtcttcatctcttgg | 141 bp |
| HbGGPS_qR |  |  | tggtaatgatgatgatgaagaagaagg |  |
| HbPOR_qF | bx003988 | protochlorophyllide reductase (POR) | cagcataatcgcagataaatgatcc | 145 bp |
| HbPOR_qR |  |  | caattgacagaaatcacgacatcag |  |
| HbSDR_qF | bx022425 | short chain dehydrogenase/reductase (SDR) | tggcaatcaaaagcttactttcttc | 148 bp |
| HbSDR_qR |  |  | attagacaccacagttcaacccaag |  |
| HbADF4_F1 | bx027864 | actin depolymerizing factor 4 (ADF4) | TTATGCTAGTTCCAAGGACAGGTTC | 154 bp |
| HbADF4_R1 |  |  | cattacgcttgtccatcacattg |  |
