# Supplementary material for: Transcriptome analysis of Pará rubber tree (H. brasiliensis) seedlings under ethylene stimulation
Source: BMC Plant Biol. 2021 Sep 13;21:420. doi: 10.1186/s12870-021-03196-y (PMC8436496; doi:10.1186/s12870-021-03196-y)
Supplement: Supplementary file 2 — Additional file 2 : Table S2. Probe list and fold change of 3270 genes that showed a significant difference in expression between the ethephon and mock treatments. [file 12870_2021_3196_MOESM2_ESM.docx]

| Nakano et al. Supplemental Table 2. | | | |  |  |  |  |
| --- | --- | --- | --- | --- | --- | --- | --- |
|  |  |  |  |  | Et/Mock F.C. (Ln) | | |
| Group | probe | Hb ID | Arabidopsis locus | Short description | 6H | 24H | 48H |
| 0 | 9 | bx000015 | AT2G47110 | ubiquitin 6 | 1.654 | 2.454 | 1.476 |
| 0 | 71 | bx000130 | AT2G45960 | TRANSMEMBRANE PROTEIN A, NAMED PLASMA MEMBRANE INTRINSIC PROTEIN 1;2, plasma membrane intrinsic protein 1B | 0.761 | 3.430 | 1.907 |
| 0 | 84 | bx000157 | AT5G11770 | NADH-ubiquinone oxidoreductase 20 kDa subunit, mitochondrial | 0.192 | 2.282 | 2.507 |
| 0 | 140 | bx000261 | AT5G39570 | unknown protein | 2.870 | 5.365 | 4.849 |
| 0 | 162 | bx000300 |  |  | 0.215 | 2.534 | 2.731 |
| 0 | 197 | bx000361 | AT1G60590 | Pectin lyase-like superfamily protein | 0.810 | 3.187 | 3.173 |
| 0 | 370 | bx000657 |  |  | -0.146 | 2.912 | 2.578 |
| 0 | 384 | bx000682 | AT2G45960 | TRANSMEMBRANE PROTEIN A, NAMED PLASMA MEMBRANE INTRINSIC PROTEIN 1;2, plasma membrane intrinsic protein 1B | 0.692 | 2.496 | 2.636 |
| 0 | 413 | bx000735 | AT5G35735 | Auxin-responsive family protein | 2.806 | 3.021 | 2.235 |
| 0 | 537 | bx000949 | AT4G37990 | CINNAMYL-ALCOHOL DEHYDROGENASE B2, ARABIDOPSIS THALIANA CINNAMYL-ALCOHOL DEHYDROGENASE 8, elicitor-activated gene 3-2 | 4.211 | 5.492 | 4.897 |
| 0 | 594 | bx001051 |  |  | 0.576 | 3.182 | 3.234 |
| 0 | 703 | bx001246 | AT5G58660 | 2-oxoglutarate (2OG) and Fe(II)-dependent oxygenase superfamily protein | 1.532 | 3.314 | 1.267 |
| 0 | 758 | bx001340 | AT3G54420 | CHITINASE CLASS IV, homolog of carrot EP3-3 chitinase | 0.991 | 2.500 | 2.565 |
| 0 | 961 | bx001656 | AT4G12300 | cytochrome P450, family 706, subfamily A, polypeptide 4, cytochrome P450, family 706, subfamily A, polypeptide 4 | 0.711 | 3.561 | 2.656 |
| 0 | 983 | bx001695 |  |  | 2.714 | 3.444 | 3.262 |
| 0 | 1034 | bx001774 | AT2G25490 | EIN3-binding F box protein 1 | 2.032 | 2.896 | 2.843 |
| 0 | 1296 | bx002187 | AT4G23100 | ROOT MERISTEMLESS 1, PHYTOALEXIN DEFICIENT 2, CADMIUM SENSITIVE 2, glutamate-cysteine ligase | 1.630 | 2.540 | 2.089 |
| 0 | 1302 | bx002197 | AT5G63160 | BTB and TAZ domain protein 1 | 2.182 | 2.579 | 1.747 |
| 0 | 1374 | bx002314 | AT1G09970 | receptor-like kinase 7, Leucine-rich receptor-like protein kinase family protein | 2.583 | 3.143 | 2.230 |
| 0 | 1520 | bx002526 | AT2G27830 | unknown protein | 1.576 | 2.516 | 2.175 |
| 0 | 1536 | bx002552 | AT5G49360 | beta-xylosidase 1 | 0.402 | 1.867 | 3.742 |
| 0 | 1557 | bx002580 | AT3G06880 | Transducin/WD40 repeat-like superfamily protein | 2.646 | 3.619 | 2.626 |
| 0 | 1677 | bx002770 | AT3G21250 | ARABIDOPSIS THALIANA MULTIDRUG RESISTANCE-ASSOCIATED PROTEIN 6, ATP-binding cassette C8, multidrug resistance-associated protein 6 | 3.427 | 3.435 | 2.729 |
| 0 | 1735 | bx002859 | AT5G49700 | Predicted AT-hook DNA-binding family protein | 2.112 | 3.979 | 3.028 |
| 0 | 1874 | bx003070 | AT5G43440 | 2-oxoglutarate (2OG) and Fe(II)-dependent oxygenase superfamily protein | 1.418 | 2.746 | 3.364 |
| 0 | 1955 | bx003200 | AT4G17030 | expansin-like B1 | 5.272 | 6.137 | 3.949 |
| 0 | 1970 | bx003226 | AT2G41230 | ORGAN SIZE RELATED 1, unknown protein | 6.496 | 8.906 | 7.551 |
| 0 | 1995 | bx003259 | AT3G61660 | unknown protein | 5.305 | 4.898 | 5.217 |
| 0 | 2168 | bx003519 | AT5G40780 | lysine histidine transporter 1 | 2.182 | 3.058 | 1.657 |
| 0 | 2217 | bx003596 | AT2G39980 | HXXXD-type acyl-transferase family protein | 4.810 | 6.319 | 8.048 |
| 0 | 2228 | bx003613 | AT5G05580 | fatty acid desaturase 8 | 1.808 | 3.536 | 2.770 |
| 0 | 2256 | bx003652 |  |  | 1.408 | 3.404 | 1.738 |
| 0 | 2307 | bx003729 | AT3G22200 | POLLEN-PISTIL INCOMPATIBILITY 2, HEXENAL RESPONSE1, GAMMA-AMINOBUTYRATE TRANSAMINASE, Pyridoxal phosphate (PLP)-dependent transferases superfamily protein | 2.638 | 3.939 | 4.180 |
| 0 | 2382 | bx003836 | AT1G62790 | Bifunctional inhibitor/lipid-transfer protein/seed storage 2S albumin superfamily protein | 1.553 | 2.945 | 1.168 |
| 0 | 2415 | bx003883 | AT3G16510 | Calcium-dependent lipid-binding (CaLB domain) family protein | 1.760 | 2.571 | 1.985 |
| 0 | 2426 | bx003900 | AT4G02340 | alpha/beta-Hydrolases superfamily protein | 1.213 | 2.587 | 2.325 |
| 0 | 2533 | bx004062 | AT1G24020 | MLP-like protein 423 | 3.950 | 5.027 | 6.054 |
| 0 | 2714 | bx004343 | AT1G47530 | MATE efflux family protein | 2.146 | 2.888 | 2.535 |
| 0 | 2716 | bx004346 | AT3G21760 | HYPOSTATIN RESISTANCE 1, UDP-Glycosyltransferase superfamily protein | 5.265 | 5.422 | 4.625 |
| 0 | 3031 | bx004808 | AT1G18850 | unknown protein | 1.700 | 2.399 | 1.847 |
| 0 | 3046 | bx004829 | AT5G19380 | CRT (chloroquine-resistance transporter)-like transporter 1 | 2.211 | 2.481 | 1.889 |
| 0 | 3186 | bx005034 | AT4G39640 | gamma-glutamyl transpeptidase 1 | 4.315 | 4.424 | 4.218 |
| 0 | 3316 | bx005222 | AT5G52450 | MATE efflux family protein | 2.611 | 2.689 | 2.405 |
| 0 | 3326 | bx005236 | AT1G79460 | GA REQUIRING 2, ARABIDOPSIS THALIANA ENT-KAURENE SYNTHASE 1, ARABIDOPSIS THALIANA ENT-KAURENE SYNTHASE, Terpenoid cyclases/Protein prenyltransferases superfamily protein | 3.397 | 4.952 | 5.376 |
| 0 | 3335 | bx005246 | AT1G29860 | WRKY DNA-binding protein 71 | 1.403 | 1.979 | 2.700 |
| 0 | 3398 | bx005331 |  |  | 1.750 | 3.725 | 3.639 |
| 0 | 3409 | bx005347 | AT3G54700 | phosphate transporter 1;7 | -0.221 | 2.802 | 3.652 |
| 0 | 3439 | bx005402 | AT2G21220 | SAUR-like auxin-responsive protein family | 2.717 | 4.170 | 3.455 |
| 0 | 3463 | bx005434 | AT1G58420 | Uncharacterised conserved protein UCP031279 | 1.803 | 4.225 | 4.187 |
| 0 | 3496 | bx005480 | AT3G47960 | Major facilitator superfamily protein | 4.881 | 5.561 | 4.426 |
| 0 | 3507 | bx005496 | AT2G36630 | Sulfite exporter TauE/SafE family protein | 3.371 | 2.813 | 3.163 |
| 0 | 3550 | bx005569 | AT5G55380 | MBOAT (membrane bound O-acyl transferase) family protein | 3.073 | 3.853 | 2.410 |
| 0 | 3591 | bx005626 | AT1G21780 | BTB/POZ domain-containing protein | 2.229 | 2.868 | 3.599 |
| 0 | 3812 | bx005945 | AT5G64410 | ARABIDOPSIS THALIANA OLIGOPEPTIDE TRANSPORTER 4, oligopeptide transporter 4 | 2.228 | 2.723 | 2.025 |
| 0 | 3827 | bx005963 | AT4G23470 | PLAC8 family protein | 1.499 | 2.976 | 1.324 |
| 0 | 3864 | bx006010 | AT1G07710 | Ankyrin repeat family protein | 1.026 | 1.655 | 2.328 |
| 0 | 3986 | bx006204 | AT5G13080 | ARABIDOPSIS THALIANA WRKY DNA-BINDING PROTEIN 75, WRKY DNA-binding protein 75 | 5.378 | 6.971 | 6.835 |
| 0 | 4014 | bx006242 | AT5G61820 | unknown protein | 1.575 | 3.040 | 2.221 |
| 0 | 4391 | bx006796 | AT5G41670 | 6-phosphogluconate dehydrogenase family protein | 2.147 | 2.622 | 1.742 |
| 0 | 4403 | bx006814 | AT1G07090 | LIGHT SENSITIVE HYPOCOTYLS 6, Protein of unknown function (DUF640) | 1.736 | 2.775 | 2.161 |
| 0 | 4474 | bx006914 | AT5G26250 | Major facilitator superfamily protein | 1.848 | 0.261 | 2.764 |
| 0 | 4491 | bx006940 |  |  | 1.082 | 2.642 | 1.504 |
| 0 | 4521 | bx006982 | AT5G60900 | receptor-like protein kinase 1 | 2.569 | 2.053 | 2.840 |
| 0 | 4648 | bx007172 | AT3G47570 | Leucine-rich repeat protein kinase family protein | 3.343 | 4.244 | 3.122 |
| 0 | 4649 | bx007173 | AT1G74240 | Mitochondrial substrate carrier family protein | 2.263 | 2.412 | 1.987 |
| 0 | 4760 | bx007323 | AT2G37770 | Chloroplastic aldo-keto reductase, Aldo-keto reductase family 4 member C9, NAD(P)-linked oxidoreductase superfamily protein | 0.023 | 2.619 | 2.872 |
| 0 | 4764 | bx007330 | AT4G17350 | Plant protein of unknown function (DUF828) with plant pleckstrin homology-like region | 3.933 | 4.043 | 3.993 |
| 0 | 4927 | bx007564 | AT1G59960 | NAD(P)-linked oxidoreductase superfamily protein | 2.392 | 3.123 | 3.399 |
| 0 | 4936 | bx007577 | AT3G06350 | MATERNAL EFFECT EMBRYO ARREST 32, EMBRYO DEFECTIVE 3004, dehydroquinate dehydratase, putative / shikimate dehydrogenase, putative | 4.253 | 6.923 | 6.269 |
| 0 | 4940 | bx007583 | AT1G13960 | WRKY DNA-binding protein 4 | 2.955 | 3.804 | 2.790 |
| 0 | 5060 | bx007762 | AT1G09970 | receptor-like kinase 7, Leucine-rich receptor-like protein kinase family protein | 2.481 | 3.172 | 2.226 |
| 0 | 5067 | bx007770 | AT3G18295 | Protein of unknown function (DUF1639) | 0.736 | 3.561 | 3.458 |
| 0 | 5150 | bx007883 | AT1G22370 | UDP-glucosyl transferase 85A5 | 2.484 | 5.883 | 3.029 |
| 0 | 5170 | bx007912 | AT4G17030 | expansin-like B1 | 6.173 | 8.102 | 6.781 |
| 0 | 5339 | bx008152 | AT5G11720 | Glycosyl hydrolases family 31 protein | 1.016 | 3.382 | 2.984 |
| 0 | 5446 | bx008303 | AT1G62300 | WRKY family transcription factor | 2.624 | 2.775 | 2.683 |
| 0 | 5588 | bx008509 | AT3G51680 | short-chain dehydrogenase/reductase 2, NAD(P)-binding Rossmann-fold superfamily protein | 5.202 | 5.460 | 4.192 |
| 0 | 5622 | bx008560 | AT2G44080 | ARGOS-like | 4.642 | 4.956 | 5.365 |
| 0 | 5760 | bx008762 | AT1G15520 | Arabidopsis thaliana ATP-binding cassette G40, ATP-binding cassette G40, pleiotropic drug resistance 12 | 1.888 | 2.959 | 1.814 |
| 0 | 5792 | bx008805 | AT5G43420 | RING/U-box superfamily protein | 1.790 | 3.630 | 2.757 |
| 0 | 6011 | bx009088 | AT3G26590 | MATE efflux family protein | 1.275 | 2.688 | 2.268 |
| 0 | 6088 | bx009188 | AT1G10586 | basic helix-loop-helix (bHLH) DNA-binding superfamily protein | 1.911 | 2.720 | 1.939 |
| 0 | 6098 | bx009199 | AT4G21600 | endonuclease 5 | 0.860 | 3.291 | 3.229 |
| 0 | 6186 | bx009322 | AT4G20980 | Eukaryotic translation initiation factor 3 subunit 7 (eIF-3) | 1.624 | 2.303 | 1.253 |
| 0 | 6210 | bx009363 | AT1G68320 | myb domain protein 62 | 5.327 | 5.597 | 5.262 |
| 0 | 6237 | bx009400 | AT4G31970 | cytochrome P450, family 82, subfamily C, polypeptide 2, cytochrome P450, family 82, subfamily C, polypeptide 2 | 7.147 | 7.445 | 7.798 |
| 0 | 6256 | bx009428 | AT2G37760 | Aldo-keto reductase family 4 member C8, NAD(P)-linked oxidoreductase superfamily protein | 0.374 | 3.826 | 2.949 |
| 0 | 6269 | bx009451 | AT4G17030 | expansin-like B1 | 3.862 | 6.871 | 3.812 |
| 0 | 6304 | bx009492 | AT1G03220 | Eukaryotic aspartyl protease family protein | 2.013 | 4.519 | 3.786 |
| 0 | 6485 | bx009769 | AT2G27150 | Arabidopsis thaliana aldehyde oxidase 3, Aldehyde oxidase delta, abscisic aldehyde oxidase 3 | 2.243 | 2.721 | 1.989 |
| 0 | 6614 | bx009951 | AT1G17860 | Kunitz family trypsin and protease inhibitor protein | -0.563 | 2.380 | 2.321 |
| 0 | 6615 | bx009953 | AT5G19600 | sulfate transporter 3;5 | 0.508 | 4.196 | 3.773 |
| 0 | 6829 | bx010237 | AT2G21340 | MATE efflux family protein | 1.062 | 3.502 | 3.355 |
| 0 | 6868 | bx010296 | AT5G47920 | unknown protein | 1.804 | 3.198 | 2.854 |
| 0 | 7009 | bx010499 | AT1G73500 | MAP kinase kinase 9 | 2.172 | 2.412 | 1.695 |
| 0 | 7051 | bx010568 |  |  | 3.868 | 4.021 | 3.014 |
| 0 | 7155 | bx010716 | AT4G29210 | gamma-glutamyl transpeptidase 3, gamma-glutamyl transpeptidase 4 | 2.280 | 2.714 | 2.074 |
| 0 | 7184 | bx010755 | AT5G23810 | amino acid permease 7 | 2.511 | 3.146 | 2.157 |
| 0 | 7428 | bx011084 | AT2G29630 | PYRIMIDINE REQUIRING, thiaminC | 1.842 | 4.110 | 3.382 |
| 0 | 7437 | bx011097 | AT2G41230 | ORGAN SIZE RELATED 1, unknown protein | 2.174 | 3.687 | 3.210 |
| 0 | 7449 | bx011113 | AT1G31230 | aspartate kinase-homoserine dehydrogenase i | 1.313 | 2.554 | 1.188 |
| 0 | 7458 | bx011127 | AT2G31480 | unknown protein | 0.309 | 2.647 | 3.444 |
| 0 | 7638 | bx011386 | AT2G02800 | protein kinase 2B | 2.227 | 2.901 | 2.248 |
| 0 | 7778 | bx011589 | AT4G22530 | S-adenosyl-L-methionine-dependent methyltransferases superfamily protein | 3.961 | 4.675 | 4.205 |
| 0 | 7872 | bx011737 | AT2G22420 | Peroxidase superfamily protein | 2.434 | 1.732 | 2.530 |
| 0 | 7965 | bx011862 | AT5G43410 | Integrase-type DNA-binding superfamily protein | 4.479 | 5.007 | 4.095 |
| 0 | 7984 | bx011885 |  |  | 0.819 | 3.016 | 2.944 |
| 0 | 8071 | bx011999 | AT1G73830 | BR enhanced expression 3 | 1.831 | 4.056 | 3.588 |
| 0 | 8189 | bx012163 | AT1G33540 | serine carboxypeptidase-like 18 | 0.902 | 2.605 | 2.456 |
| 0 | 8272 | bx012280 | AT5G22920 | CHY-type/CTCHY-type/RING-type Zinc finger protein | 2.346 | 2.554 | 2.060 |
| 0 | 8442 | bx012523 | AT4G15480 | UDP-Glycosyltransferase superfamily protein | 0.976 | 1.991 | 2.567 |
| 0 | 8465 | bx012563 | AT1G14540 | Peroxidase superfamily protein | 6.462 | 6.124 | 5.607 |
| 0 | 8600 | bx012753 | AT3G49680 | branched-chain aminotransferase 3 | 0.556 | 3.364 | 0.652 |
| 0 | 8638 | bx012805 | AT2G27880 | ARGONAUTE 5, Argonaute family protein | -1.872 | -0.493 | 4.218 |
| 0 | 8647 | bx012815 | AT3G21690 | MATE efflux family protein | 2.019 | 6.615 | 6.163 |
| 0 | 8681 | bx012862 | AT2G32260 | phosphorylcholine cytidylyltransferase | 0.636 | 3.086 | 3.211 |
| 0 | 8714 | bx012912 |  |  | 0.605 | 3.436 | 3.622 |
| 0 | 8819 | bx013052 | AT3G22660 | rRNA processing protein-related | 1.271 | 2.318 | 1.591 |
| 0 | 8882 | bx013143 | AT1G75250 | RADIALIS-LIKE SANT/MYB 3, RAD-like 6 | 1.078 | 2.531 | 1.697 |
| 0 | 8916 | bx013190 | AT4G00870 | basic helix-loop-helix (bHLH) DNA-binding superfamily protein | 2.615 | 3.580 | 1.920 |
| 0 | 8945 | bx013234 | AT2G36730 | Pentatricopeptide repeat (PPR) superfamily protein | 1.806 | 2.807 | 1.664 |
| 0 | 8955 | bx013253 | AT1G33540 | serine carboxypeptidase-like 18 | -0.638 | 0.166 | 2.412 |
| 0 | 8998 | bx013321 | AT1G59960 | NAD(P)-linked oxidoreductase superfamily protein | 2.620 | 3.885 | 3.433 |
| 0 | 9045 | bx013386 |  |  | 0.260 | 3.098 | 2.949 |
| 0 | 9076 | bx013429 | AT1G33440 | Major facilitator superfamily protein | 1.594 | 2.397 | 1.738 |
| 0 | 9103 | bx013466 | AT4G31985 | Ribosomal protein L39 family protein | 1.219 | 2.335 | 1.299 |
| 0 | 9390 | bx013899 | AT1G09970 | receptor-like kinase 7, Leucine-rich receptor-like protein kinase family protein | 2.583 | 3.165 | 2.194 |
| 0 | 9434 | bx013959 | AT2G37190 | Ribosomal protein L11 family protein | -1.053 | 0.280 | 2.818 |
| 0 | 9449 | bx013984 | AT2G45960 | TRANSMEMBRANE PROTEIN A, NAMED PLASMA MEMBRANE INTRINSIC PROTEIN 1;2, plasma membrane intrinsic protein 1B | 0.602 | 3.328 | 1.798 |
| 0 | 9672 | bx014297 | AT1G76680 | ARABIDOPSIS 12-OXOPHYTODIENOATE REDUCTASE 1, 12-oxophytodienoate reductase 1 | 3.497 | 3.823 | 2.947 |
| 0 | 9733 | bx014388 | AT3G21250 | ARABIDOPSIS THALIANA MULTIDRUG RESISTANCE-ASSOCIATED PROTEIN 6, ATP-binding cassette C8, multidrug resistance-associated protein 6 | 3.206 | 2.770 | 2.442 |
| 0 | 10056 | bx014841 | AT1G74240 | Mitochondrial substrate carrier family protein | 2.281 | 2.374 | 1.957 |
| 0 | 10078 | bx014871 | AT5G02500 | HEAT SHOCK PROTEIN 70-1, ARABIDOPSIS THALIANA HEAT SHOCK COGNATE PROTEIN 70-1, heat shock cognate protein 70-1 | -0.544 | 2.701 | 3.306 |
| 0 | 10132 | bx014945 | AT3G02230 | ARABIDOPSIS THALIANA REVERSIBLY GLYCOSYLATED POLYPEPTIDE 1, reversibly glycosylated polypeptide 1 | 2.194 | 2.730 | 2.009 |
| 0 | 10197 | bx015052 | AT2G26070 | REVERSION-TO-ETHYLENE SENSITIVITY1, Protein of unknown function (DUF778) | 1.871 | 2.847 | 2.636 |
| 0 | 10310 | bx015243 | AT1G22380 | UDP-glucosyl transferase 85A3 | 0.717 | 2.129 | 2.750 |
| 0 | 10832 | bx016090 | AT5G61820 | unknown protein | 0.293 | 2.986 | 3.259 |
| 0 | 10961 | bx016299 | AT1G65840 | polyamine oxidase 4 | 2.527 | 3.045 | 2.157 |
| 0 | 11017 | bx016389 | AT2G26070 | REVERSION-TO-ETHYLENE SENSITIVITY1, Protein of unknown function (DUF778) | 1.649 | 2.446 | 2.110 |
| 0 | 11046 | bx016437 | AT2G26070 | REVERSION-TO-ETHYLENE SENSITIVITY1, Protein of unknown function (DUF778) | 1.670 | 2.477 | 2.195 |
| 0 | 11211 | bx016713 | AT2G40180 | phosphatase 2C5 | 2.740 | 3.166 | 1.955 |
| 0 | 11267 | bx016804 | AT3G16565 | alanine-tRNA ligases;nucleic acid binding;ligases, forming aminoacyl-tRNA and related compounds;nucleotide binding;ATP binding | 1.953 | 2.847 | 1.500 |
| 0 | 11333 | bx016898 | AT1G33110 | MATE efflux family protein | 2.460 | 4.141 | 3.560 |
| 0 | 11605 | bx017330 | AT3G16770 | RELATED TO AP2 3, ETHYLENE RESPONSE FACTOR 72, ethylene-responsive element binding protein | 1.834 | 2.482 | 2.465 |
| 0 | 11663 | bx017418 | AT3G16565 | alanine-tRNA ligases;nucleic acid binding;ligases, forming aminoacyl-tRNA and related compounds;nucleotide binding;ATP binding | 1.818 | 2.797 | 1.408 |
| 0 | 11875 | bx017773 | AT5G03740 | HISTONE DEACETYLASE 3, histone deacetylase 2C | 1.818 | 1.836 | 3.526 |
| 0 | 11934 | bx017867 | AT3G62840 | Small nuclear ribonucleoprotein family protein | 2.060 | 2.785 | 1.638 |
| 0 | 11989 | bx017965 | AT5G25940 | early nodulin-related | 2.488 | 3.033 | 1.932 |
| 0 | 12102 | bx018165 | AT2G44480 | beta glucosidase 17 | 2.684 | 3.241 | 3.048 |
| 0 | 12198 | bx018313 | AT3G60770 | Ribosomal protein S13/S15 | 1.480 | 2.448 | 1.237 |
| 0 | 12241 | bx018378 | AT5G35400 | Pseudouridine synthase family protein | 0.335 | 2.407 | 2.500 |
| 0 | 12271 | bx018418 | AT1G20925 | Auxin efflux carrier family protein | 4.922 | 4.453 | 4.181 |
| 0 | 12624 | bx018920 | AT4G35980 | unknown protein | 0.365 | 2.584 | 3.033 |
| 0 | 12661 | bx018964 | AT5G13700 | polyamine oxidase 1 | 2.565 | 2.442 | 2.327 |
| 0 | 12877 | bx019258 | AT1G17530 | translocase of inner mitochondrial membrane 23 | 2.498 | 2.312 | 1.973 |
| 0 | 12887 | bx019274 | AT3G45620 | Transducin/WD40 repeat-like superfamily protein | 1.574 | 2.368 | 2.351 |
| 0 | 13001 | bx019421 | AT3G22160 | VQ motif-containing protein | 3.794 | 5.471 | 3.783 |
| 0 | 13114 | bx019577 | AT1G23100 | GroES-like family protein | 1.403 | 2.401 | 1.130 |
| 0 | 13303 | bx019833 | AT1G78780 | pathogenesis-related family protein | 4.348 | 5.054 | 4.620 |
| 0 | 13328 | bx019866 | AT3G59140 | ATP-binding cassette C10, multidrug resistance-associated protein 14 | 1.923 | 2.981 | 2.588 |
| 0 | 13621 | bx020251 | AT3G15210 | RELATED TO AP2 5, ethylene responsive element binding factor 4 | 2.181 | 2.324 | 1.957 |
| 0 | 13646 | bx020286 | AT4G13830 | DNAJ-like 20 | 5.430 | 5.226 | 4.432 |
| 0 | 13649 | bx020289 | AT3G23940 | dehydratase family | 2.602 | 2.797 | 1.942 |
| 0 | 13731 | bx020390 | AT2G24580 | FAD-dependent oxidoreductase family protein | 2.375 | 3.950 | 2.376 |
| 0 | 13777 | bx020448 | AT1G79900 | RABIDOPSIS MITOCHONDRIAL BASIC AMINO ACID CARRIER 2, Mitochondrial substrate carrier family protein | 3.897 | 4.163 | 3.456 |
| 0 | 14084 | bx020861 | AT5G22460 | alpha/beta-Hydrolases superfamily protein | 0.838 | 2.517 | 2.268 |
| 0 | 14163 | bx020970 | AT3G26320 | cytochrome P450, family 71, subfamily B, polypeptide 36, cytochrome P450, family 71, subfamily B, polypeptide 36 | 1.166 | 2.794 | 1.981 |
| 0 | 14164 | bx020971 | AT3G46020 | RNA-binding (RRM/RBD/RNP motifs) family protein | -2.087 | -0.533 | 4.005 |
| 0 | 14202 | bx021027 | AT4G15160 | Bifunctional inhibitor/lipid-transfer protein/seed storage 2S albumin superfamily protein | 1.724 | 3.120 | 2.543 |
| 0 | 14358 | bx021231 | AT3G25590 | unknown protein | 1.273 | 2.649 | 2.503 |
| 0 | 14430 | bx021318 | AT4G35630 | phosphoserine aminotransferase | 3.504 | 3.874 | 2.972 |
| 0 | 14490 | bx021395 | AT3G14690 | cytochrome P450, family 72, subfamily A, polypeptide 15, cytochrome P450, family 72, subfamily A, polypeptide 15 | 3.580 | 3.469 | 2.800 |
| 0 | 14503 | bx021412 | AT1G79460 | GA REQUIRING 2, ARABIDOPSIS THALIANA ENT-KAURENE SYNTHASE 1, ARABIDOPSIS THALIANA ENT-KAURENE SYNTHASE, Terpenoid cyclases/Protein prenyltransferases superfamily protein | 5.620 | 6.117 | 4.529 |
| 0 | 14521 | bx021436 | AT1G73300 | serine carboxypeptidase-like 2 | 1.742 | 3.591 | 3.665 |
| 0 | 14637 | bx021597 | AT3G01390 | vacuolar membrane ATPase 10 | 1.709 | 2.428 | 1.698 |
| 0 | 14668 | bx021636 | AT2G48020 | Major facilitator superfamily protein | 2.008 | 2.933 | 2.447 |
| 0 | 14910 | bx021958 | AT1G13080 | cytochrome P450, family 71, subfamily B, polypeptide 2, cytochrome P450, family 71, subfamily B, polypeptide 2 | 3.870 | 4.545 | 2.759 |
| 0 | 14978 | bx022045 | AT1G28110 | serine carboxypeptidase-like 45 | 1.283 | 2.879 | 1.764 |
| 0 | 15221 | bx022359 | AT2G40095 | Alpha/beta hydrolase related protein | 2.888 | 2.678 | 2.358 |
| 0 | 15225 | bx022365 | AT5G19090 | Heavy metal transport/detoxification superfamily protein | 2.618 | 4.314 | 4.051 |
| 0 | 15271 | bx022425 | AT1G52340 | SUGAR-INSENSITIVE 4, SHORT-CHAIN DEHYDROGENASE REDUCTASE 1, IMPAIRED SUCROSE INDUCTION 4, GLUCOSE INSENSITIVE 1, SHORT-CHAIN DEHYDROGENASE/REDUCTASE 1, ARABIDOPSIS THALIANA ABA DEFICIENT 2, ABA DEFICIENT 2, NAD(P)-binding Rossmann-fold superfamily protein | 0.809 | 4.358 | 2.960 |
| 0 | 15283 | bx022445 | AT3G16770 | RELATED TO AP2 3, ETHYLENE RESPONSE FACTOR 72, ethylene-responsive element binding protein | 2.809 | 3.046 | 2.138 |
| 0 | 15419 | bx022620 | AT5G22300 | nitrilase 4 | 2.522 | 3.187 | 2.176 |
| 0 | 15478 | bx022695 | AT4G18100 | Ribosomal protein L32e | 2.056 | 2.983 | 1.643 |
| 0 | 15840 | bx023185 | AT5G41761 | unknown protein | 2.735 | 4.678 | 4.293 |
| 0 | 15860 | bx023207 | AT1G73500 | MAP kinase kinase 9 | 1.629 | 3.366 | 3.120 |
| 0 | 15937 | bx023307 | AT4G34135 | UDP-glucosyltransferase 73B2 | 4.791 | 5.238 | 3.912 |
| 0 | 15973 | bx023356 | AT3G63470 | serine carboxypeptidase-like 40 | 2.568 | 4.222 | 3.537 |
| 0 | 16046 | bx023456 | AT1G32450 | nitrate transporter 1.5 | 6.570 | 6.734 | 5.430 |
| 0 | 16062 | bx023478 | AT5G21940 | unknown protein | 2.453 | 2.547 | 2.782 |
| 0 | 16081 | bx023505 | AT1G58420 | Uncharacterised conserved protein UCP031279 | 2.572 | 2.542 | 2.292 |
| 0 | 16088 | bx023514 |  |  | 1.733 | 3.010 | 2.120 |
| 0 | 16097 | bx023528 | AT4G23990 | ARABIDOPSIS THALIANA CELLULOSE SYNTHASE-LIKE G3, cellulose synthase like G3 | 4.794 | 6.364 | 5.403 |
| 0 | 16106 | bx023539 | AT1G22410 | Class-II DAHP synthetase family protein | 2.591 | 4.883 | 2.886 |
| 0 | 16183 | bx023658 | AT3G59900 | auxin-regulated gene involved in organ size | 2.495 | 2.645 | 2.475 |
| 0 | 16198 | bx023675 | AT4G11140 | cytokinin response factor 1 | 2.345 | 2.936 | 2.707 |
| 0 | 16259 | bx023759 | AT4G05200 | cysteine-rich RLK (RECEPTOR-like protein kinase) 25 | 2.046 | 2.615 | 1.435 |
| 0 | 16311 | bx023827 | AT3G54420 | CHITINASE CLASS IV, homolog of carrot EP3-3 chitinase | 0.206 | 2.957 | 3.907 |
| 0 | 16355 | bx023897 | AT2G17220 | kinase 3, Protein kinase superfamily protein | 1.855 | 2.478 | 2.634 |
| 0 | 16582 | bx024195 | AT2G22480 | phosphofructokinase 5 | 1.887 | 2.827 | 1.929 |
| 0 | 16585 | bx024203 | AT3G29970 | B12D protein | 2.193 | 3.743 | 2.721 |
| 0 | 16701 | bx024382 | AT5G63380 | AMP-dependent synthetase and ligase family protein | 2.583 | 2.979 | 3.458 |
| 0 | 16958 | bx024720 | AT5G65380 | MATE efflux family protein | 5.375 | 6.189 | 5.632 |
| 0 | 17007 | bx024783 | AT3G48690 | ARABIDOPSIS THALIANA CARBOXYESTERASE 12, alpha/beta-Hydrolases superfamily protein | -0.209 | 1.654 | 2.316 |
| 0 | 17175 | bx024997 | AT2G31725 | Eukaryotic protein of unknown function (DUF842) | 1.867 | 2.789 | 1.441 |
| 0 | 17249 | bx025092 | AT5G12890 | UDP-Glycosyltransferase superfamily protein | 2.720 | 3.290 | 3.143 |
| 0 | 17400 | bx025295 | AT3G02800 | plant and fungi atypical dual-speci?city phosphatase 3, Tyrosine phosphatase family protein | 1.901 | 2.453 | 1.818 |
| 0 | 17412 | bx025322 | AT4G12300 | cytochrome P450, family 706, subfamily A, polypeptide 4, cytochrome P450, family 706, subfamily A, polypeptide 4 | 1.114 | 3.874 | 2.544 |
| 0 | 17428 | bx025347 | AT1G21400 | Thiamin diphosphate-binding fold (THDP-binding) superfamily protein | 1.947 | 3.321 | 2.176 |
| 0 | 17513 | bx025462 | AT3G59380 | PLURIPETALA, farnesyltransferase A | 1.711 | 2.384 | 1.421 |
| 0 | 17574 | bx025548 |  |  | 0.776 | 3.326 | 3.383 |
| 0 | 17589 | bx025568 | AT3G11340 | UDP-dependent glycosyltransferase 76B1, UDP-Glycosyltransferase superfamily protein | 6.474 | 6.478 | 6.258 |
| 0 | 17590 | bx025572 | AT5G06570 | alpha/beta-Hydrolases superfamily protein | 2.920 | 3.677 | 2.915 |
| 0 | 17620 | bx025610 | AT2G18690 | unknown protein | 1.883 | 2.857 | 1.393 |
| 0 | 17697 | bx025717 | AT3G53810 | Concanavalin A-like lectin protein kinase family protein | 2.410 | 3.857 | 3.429 |
| 0 | 17949 | bx026046 |  |  | 5.473 | 6.633 | 6.055 |
| 0 | 18002 | bx026112 | AT1G25275 | unknown protein | 2.898 | 5.108 | 2.737 |
| 0 | 18300 | bx026508 | AT1G19020 | unknown protein | 3.310 | 3.779 | 2.423 |
| 0 | 18355 | bx026579 | AT1G73500 | MAP kinase kinase 9 | 2.324 | 2.513 | 1.809 |
| 0 | 18374 | bx026598 | AT1G56300 | Chaperone DnaJ-domain superfamily protein | 0.462 | 2.845 | 2.191 |
| 0 | 18511 | bx026788 | AT4G17350 | Plant protein of unknown function (DUF828) with plant pleckstrin homology-like region | 2.858 | 3.036 | 3.695 |
| 0 | 18629 | bx026931 | AT2G29420 | GLUTATHIONE S-TRANSFERASE 25, glutathione S-transferase tau 7 | 3.655 | 5.294 | 2.771 |
| 0 | 18692 | bx027026 | AT1G02030 | C2H2-like zinc finger protein | 0.133 | 0.743 | 2.510 |
| 0 | 18826 | bx027210 | AT2G01290 | ribose-5-phosphate isomerase 2 | 1.909 | 2.330 | 1.835 |
| 0 | 18970 | bx027415 | AT5G24910 | EUI-like p450 A1, cytochrome P450, family 714, subfamily A, polypeptide 1 | 1.204 | 4.111 | 3.340 |
| 0 | 19122 | bx027613 | AT3G14910 | unknown protein | 0.208 | 2.098 | 2.551 |
| 0 | 19127 | bx027618 | AT1G59960 | NAD(P)-linked oxidoreductase superfamily protein | 2.166 | 3.313 | 3.583 |
| 0 | 19187 | bx027698 | AT4G29210 | gamma-glutamyl transpeptidase 3, gamma-glutamyl transpeptidase 4 | 2.044 | 2.493 | 1.742 |
| 0 | 19207 | bx027728 | AT1G65840 | polyamine oxidase 4 | 2.503 | 3.044 | 2.167 |
| 0 | 19335 | bx027904 | AT1G26780 | LATERAL ORGAN FUSION 1, myb domain protein 117 | 2.430 | 3.559 | 3.347 |
| 0 | 19428 | bx028030 | AT1G09970 | receptor-like kinase 7, Leucine-rich receptor-like protein kinase family protein | 2.935 | 3.416 | 2.258 |
| 0 | 19430 | bx028032 | AT1G23300 | MATE efflux family protein | 3.862 | 3.008 | 3.016 |
| 0 | 19518 | bx028146 | AT4G38250 | Transmembrane amino acid transporter family protein | 1.991 | 2.457 | 3.369 |
| 0 | 19523 | bx028151 |  |  | 5.340 | 5.477 | 3.941 |
| 0 | 19567 | bx028203 |  |  | 1.315 | 3.120 | 3.240 |
| 0 | 19596 | bx028235 | AT3G09200 | Ribosomal protein L10 family protein | 0.973 | 2.809 | 1.713 |
| 0 | 19621 | bx028268 | AT1G09970 | receptor-like kinase 7, Leucine-rich receptor-like protein kinase family protein | 2.752 | 3.784 | 2.673 |
| 0 | 19649 | bx028300 | AT4G15480 | UDP-Glycosyltransferase superfamily protein | 2.119 | 3.484 | 2.825 |
| 0 | 19662 | bx028320 | AT2G02220 | phytosulfokin receptor 1 | 0.962 | 2.409 | 1.917 |
| 0 | 19805 | bx028511 | AT5G49700 | Predicted AT-hook DNA-binding family protein | 1.353 | 3.167 | 3.253 |
| 0 | 19812 | bx028521 |  |  | 3.089 | 2.969 | 2.963 |
| 0 | 19887 | bx028619 | AT5G01990 | Auxin efflux carrier family protein | 2.643 | 2.974 | 2.401 |
| 0 | 19894 | bx028628 | AT1G59950 | NAD(P)-linked oxidoreductase superfamily protein | 3.349 | 5.486 | 4.715 |
| 0 | 19902 | bx028640 | AT4G18210 | purine permease 10 | 0.648 | 2.911 | 3.174 |
| 0 | 19905 | bx028644 | AT1G74240 | Mitochondrial substrate carrier family protein | 2.270 | 2.751 | 2.078 |
| 0 | 20120 | bx028975 | AT3G07700 | Protein kinase superfamily protein | 3.517 | 2.009 | 2.772 |
| 0 | 20176 | bx029046 | AT5G17760 | P-loop containing nucleoside triphosphate hydrolases superfamily protein | 2.157 | 2.453 | 1.552 |
| 0 | 20231 | bx029118 | AT1G77280 | Protein kinase protein with adenine nucleotide alpha hydrolases-like domain | 4.869 | 5.165 | 4.433 |
| 0 | 20237 | bx029125 | AT3G57240 | beta-1,3-glucanase 3, beta-1,3-glucanase 3 | 4.509 | 6.352 | 7.118 |
| 0 | 20251 | bx029139 | AT3G21250 | ARABIDOPSIS THALIANA MULTIDRUG RESISTANCE-ASSOCIATED PROTEIN 6, ATP-binding cassette C8, multidrug resistance-associated protein 6 | 3.389 | 3.384 | 2.672 |
| 0 | 20554 | bx029527 | AT3G48250 | Buthionine sulfoximine-insensitive roots 6, Pentatricopeptide repeat (PPR) superfamily protein | 1.718 | 2.752 | 1.542 |
| 0 | 20598 | bx029588 | AT1G49570 | Peroxidase superfamily protein | 3.151 | 3.248 | 2.547 |
| 0 | 20871 | bx029950 | AT1G15710 | prephenate dehydrogenase family protein | 3.097 | 3.095 | 3.233 |
| 0 | 21022 | bx030159 | AT3G06200 | P-loop containing nucleoside triphosphate hydrolases superfamily protein | 1.553 | 1.877 | 2.598 |
| 0 | 21159 | bx030356 | AT4G15550 | indole-3-acetate beta-D-glucosyltransferase | 3.783 | 6.106 | 5.894 |
| 0 | 21293 | bx030547 | AT1G68320 | myb domain protein 62 | 4.549 | 4.794 | 4.815 |
| 0 | 21510 | bx030831 |  |  | 6.256 | 9.229 | 5.003 |
| 0 | 21515 | bx030837 | AT2G41380 | S-adenosyl-L-methionine-dependent methyltransferases superfamily protein | 2.176 | 4.153 | 1.849 |
| 0 | 21545 | bx030875 | AT3G21760 | HYPOSTATIN RESISTANCE 1, UDP-Glycosyltransferase superfamily protein | 2.629 | 2.103 | 2.458 |
| 0 | 21948 | bx031428 | AT5G09960 | unknown protein | 2.552 | 2.515 | 1.956 |
| 0 | 22184 | bx031768 | AT5G64260 | EXORDIUM like 2 | 2.820 | 3.577 | 2.255 |
| 0 | 22190 | bx031775 | AT2G32540 | CELLULOSE SYNTHASE LIKE B4, cellulose synthase-like B4 | 2.943 | 5.311 | 5.856 |
| 0 | 22394 | bx032068 | AT3G59030 | TRANSPARENT TESTA 12, A. THALIANA TRANSPARENT TESTA, MATE efflux family protein | 0.365 | 1.846 | 2.990 |
| 0 | 22485 | bx032185 | AT4G21910 | MATE efflux family protein | 2.246 | 5.835 | 6.552 |
| 0 | 22660 | bx032445 | AT3G63010 | GA INSENSITIVE DWARF1B, alpha/beta-Hydrolases superfamily protein | 2.047 | 3.100 | 2.828 |
| 0 | 22697 | bx032510 | AT2G44480 | beta glucosidase 17 | 1.087 | 2.315 | 2.087 |
| 0 | 22860 | bx032738 | AT1G49820 | 5-methylthioribose kinase 1, S-methyl-5-thioribose kinase | 5.494 | 5.307 | 4.049 |
| 0 | 22890 | bx032786 | AT5G01520 | ABA Insensitive RING Protein 2, RING/U-box superfamily protein | 0.769 | 1.800 | 2.359 |
| 0 | 22948 | bx032868 | AT1G68440 | unknown protein | 2.930 | 3.365 | 2.179 |
| 0 | 23073 | bx033047 | AT5G32440 | Ubiquitin system component Cue protein | 0.462 | 2.524 | 0.908 |
| 0 | 23161 | bx033168 | AT5G11520 | YELLOW-LEAF-SPECIFIC GENE 4, aspartate aminotransferase 3 | 1.442 | 1.958 | 2.347 |
| 0 | 23286 | bx033355 | AT1G78780 | pathogenesis-related family protein | 3.157 | 5.007 | 4.813 |
| 0 | 23457 | bx033605 | AT1G75190 | unknown protein | 2.718 | 2.784 | 2.292 |
| 0 | 23497 | bx033665 | AT1G20925 | Auxin efflux carrier family protein | 4.789 | 4.474 | 4.000 |
| 0 | 23630 | bx033851 | AT1G02205 | ECERIFERUM 1, Fatty acid hydroxylase superfamily | 3.223 | 4.404 | 4.500 |
| 0 | 23670 | bx033901 | AT1G70080 | Terpenoid cyclases/Protein prenyltransferases superfamily protein | 4.139 | 6.896 | 6.257 |
| 0 | 23685 | bx033922 | AT1G16030 | heat shock protein 70B | -0.263 | 2.987 | 3.571 |
| 0 | 23771 | bx034044 | AT5G35400 | Pseudouridine synthase family protein | 0.319 | 2.413 | 2.437 |
| 0 | 23805 | bx034094 | AT5G63850 | amino acid permease 4 | 2.469 | 2.941 | 2.022 |
| 0 | 24050 | bx034453 | AT1G73270 | serine carboxypeptidase-like 6 | 1.743 | 3.617 | 3.697 |
| 0 | 24248 | bx034731 | AT1G07900 | LOB domain-containing protein 1 | 1.340 | 1.857 | 4.038 |
| 0 | 24250 | bx034734 | AT3G25590 | unknown protein | 1.339 | 2.013 | 2.802 |
| 0 | 24332 | bx034864 | AT3G22160 | VQ motif-containing protein | 3.555 | 3.756 | 2.920 |
| 0 | 24447 | bx035039 | AT2G23690 | unknown protein | 4.160 | 3.887 | 3.898 |
| 0 | 24591 | bx035240 | AT3G26590 | MATE efflux family protein | 0.968 | 2.622 | 2.087 |
| 0 | 24652 | bx035328 | AT1G28110 | serine carboxypeptidase-like 45 | 1.279 | 2.917 | 1.697 |
| 0 | 24898 | bx035674 | AT2G37770 | Chloroplastic aldo-keto reductase, Aldo-keto reductase family 4 member C9, NAD(P)-linked oxidoreductase superfamily protein | 3.868 | 6.705 | 4.638 |
| 0 | 24943 | bx035731 |  |  | 0.876 | 3.909 | 2.444 |
| 0 | 25546 | bx036621 | AT2G44480 | beta glucosidase 17 | 1.055 | 2.352 | 2.030 |
| 0 | 25685 | bx036844 | AT3G26430 | GDSL-like Lipase/Acylhydrolase superfamily protein | 3.422 | 3.621 | 4.313 |
| 0 | 25721 | bx036902 | AT1G20925 | Auxin efflux carrier family protein | 4.665 | 4.263 | 3.921 |
| 0 | 25925 | bx037209 | AT2G48020 | Major facilitator superfamily protein | 2.014 | 2.976 | 2.494 |
| 0 | 25959 | bx037263 | AT3G56710 | sigma factor binding protein 1 | 6.915 | 6.776 | 5.172 |
| 0 | 26095 | bx037495 | AT1G76520 | Auxin efflux carrier family protein | 4.611 | 4.254 | 3.918 |
| 0 | 26172 | bx037632 | AT3G28960 | Transmembrane amino acid transporter family protein | 3.689 | 4.425 | 2.712 |
| 0 | 26215 | bx037690 | AT1G79670 | RESISTANCE TO FUSARIUM OXYSPORUM 1, Wall-associated kinase family protein | 1.310 | 2.622 | 2.121 |
| 0 | 26239 | bx037720 | AT3G57810 | Cysteine proteinases superfamily protein | 2.127 | 2.375 | 1.934 |
| 0 | 26255 | bx037742 | AT5G53980 | homeobox protein 52 | 3.443 | 5.332 | 3.606 |
| 0 | 26267 | bx037760 | AT2G41380 | S-adenosyl-L-methionine-dependent methyltransferases superfamily protein | 4.850 | 5.513 | 3.939 |
| 0 | 26292 | bx037795 | AT4G34050 | caffeoyl coenzyme A O-methyltransferase 1, S-adenosyl-L-methionine-dependent methyltransferases superfamily protein | 3.418 | 3.713 | 2.927 |
| 0 | 26296 | bx037801 | AT4G20970 | basic helix-loop-helix (bHLH) DNA-binding superfamily protein | 2.037 | 2.764 | 1.985 |
| 0 | 26312 | bx037824 | AT1G62660 | Glycosyl hydrolases family 32 protein | 1.500 | 4.099 | 4.308 |
| 0 | 26333 | bx037851 | AT1G68810 | basic helix-loop-helix (bHLH) DNA-binding superfamily protein | 2.381 | 3.173 | 3.594 |
| 0 | 26334 | bx037852 | AT5G59320 | lipid transfer protein 3 | 5.349 | 6.824 | 5.718 |
| 0 | 26339 | bx037863 | AT4G35160 | O-methyltransferase family protein | 6.851 | 6.859 | 6.466 |
| 0 | 26408 | bx037951 | AT5G61890 | Integrase-type DNA-binding superfamily protein | 1.901 | 3.625 | 3.889 |
| 0 | 26496 | bx038068 | AT1G68620 | alpha/beta-Hydrolases superfamily protein | 4.153 | 4.282 | 3.897 |
| 0 | 26519 | bx038100 | AT3G23240 | ethylene response factor 1 | 5.920 | 7.621 | 7.165 |
| 0 | 26542 | bx038135 | AT1G68380 | Core-2/I-branching beta-1,6-N-acetylglucosaminyltransferase family protein | 3.487 | 3.911 | 3.236 |
| 0 | 26619 | bx038233 | AT3G13610 | 2-oxoglutarate (2OG) and Fe(II)-dependent oxygenase superfamily protein | 1.599 | 2.941 | 1.388 |
| 0 | 26718 | bx038369 | AT3G51680 | short-chain dehydrogenase/reductase 2, NAD(P)-binding Rossmann-fold superfamily protein | 2.446 | 3.412 | 1.882 |
| 0 | 26731 | bx038387 | AT2G14580 | basic pathogenesis-related protein 1 | 3.570 | 6.613 | 7.223 |
| 0 | 26825 | bx038510 | AT4G13420 | high affinity K+ transporter 5, ARABIDOPSIS THALIANA HIGH AFFINITY K+ TRANSPORTER 5, high affinity K+ transporter 5 | 2.410 | 3.605 | 1.873 |
| 0 | 26846 | bx038537 | AT4G38540 | FAD/NAD(P)-binding oxidoreductase family protein | 2.638 | 3.033 | 2.963 |
| 0 | 26872 | bx038568 | AT2G29630 | PYRIMIDINE REQUIRING, thiaminC | 1.043 | 3.321 | 2.995 |
| 0 | 27006 | bx038747 | AT2G29420 | GLUTATHIONE S-TRANSFERASE 25, glutathione S-transferase tau 7 | 4.026 | 5.200 | 4.909 |
| 0 | 27009 | bx038750 | AT4G31940 | cytochrome P450, family 82, subfamily C, polypeptide 4, cytochrome P450, family 82, subfamily C, polypeptide 4 | 4.734 | 5.217 | 3.736 |
| 0 | 27096 | bx038862 | AT1G69490 | Arabidopsis NAC domain containing protein 29, NAC-like, activated by AP3/PI | 1.238 | 2.301 | 3.090 |
| 0 | 27243 | bx039059 | AT1G33540 | serine carboxypeptidase-like 18 | 1.381 | 3.282 | 4.716 |
| 0 | 27315 | bx039159 | AT4G37360 | cytochrome P450, family 81, subfamily D, polypeptide 2, cytochrome P450, family 81, subfamily D, polypeptide 2 | 4.320 | 4.867 | 4.093 |
| 0 | 27320 | bx039164 |  |  | 0.754 | 2.424 | 1.903 |
| 0 | 27347 | bx039197 | AT4G17500 | ethylene responsive element binding factor 1 | 5.740 | 5.215 | 4.499 |
| 0 | 27393 | bx039254 | AT4G17500 | ethylene responsive element binding factor 1 | 2.028 | 2.693 | 1.645 |
| 0 | 27415 | bx039282 |  |  | 1.665 | 2.402 | 1.799 |
| 0 | 27460 | bx039343 |  |  | 2.173 | 2.670 | 2.481 |
| 0 | 27494 | bx039385 |  |  | 2.201 | 3.301 | 2.111 |
| 0 | 27514 | bx039406 | AT1G05675 | UDP-Glycosyltransferase superfamily protein | 1.918 | 4.335 | 4.292 |
| 0 | 27521 | bx039417 |  |  | 1.258 | 1.139 | 2.959 |
| 0 | 27640 | bx039574 | AT3G11210 | SGNH hydrolase-type esterase superfamily protein | -0.066 | 2.152 | 3.271 |
| 0 | 27741 | bx039709 | AT5G60900 | receptor-like protein kinase 1 | 0.294 | 2.140 | 2.425 |
| 0 | 27853 | bx039856 | AT3G26300 | cytochrome P450, family 71, subfamily B, polypeptide 34, cytochrome P450, family 71, subfamily B, polypeptide 34 | 5.755 | 7.276 | 5.579 |
| 0 | 27879 | bx039890 | AT1G74055 | unknown protein | 1.397 | 2.103 | 3.071 |
| 0 | 27912 | bx039931 | AT2G37650 | GRAS family transcription factor | 2.422 | 4.686 | 4.371 |
| 0 | 27956 | bx039995 | AT2G16050 | Cysteine/Histidine-rich C1 domain family protein | 3.910 | 5.039 | 3.849 |
| 0 | 27957 | bx039996 |  |  | 3.423 | 4.085 | 5.096 |
| 0 | 27962 | bx040001 | AT3G23240 | ethylene response factor 1 | 3.806 | 7.491 | 8.038 |
| 0 | 28016 | bx040066 | AT1G68320 | myb domain protein 62 | 2.761 | 4.408 | 2.768 |
| 0 | 28080 | bx040151 | AT5G17240 | SET domain group 40 | 2.445 | 2.763 | 2.122 |
| 0 | 28082 | bx040153 | AT1G13700 | 6-phosphogluconolactonase 1 | 1.559 | 4.203 | 5.783 |
| 0 | 28157 | bx040248 | AT1G70170 | matrix metalloproteinase | 1.220 | 1.683 | 5.689 |
| 0 | 28298 | bx040426 | AT1G76690 | ARABIDOPSIS 12-OXOPHYTODIENOATE REDUCTASE 2, 12-oxophytodienoate reductase 2 | 3.002 | 3.738 | 3.142 |
| 0 | 28410 | bx040568 | AT4G36250 | aldehyde dehydrogenase 3F1 | -1.410 | 5.463 | 1.048 |
| 0 | 28445 | bx040609 | AT1G15385 | unknown protein | 5.674 | 5.884 | 4.517 |
| 0 | 28459 | bx040628 | AT3G45420 | Concanavalin A-like lectin protein kinase family protein | 2.656 | 4.859 | 3.678 |
| 0 | 28759 | bx041012 | AT1G24020 | MLP-like protein 423 | 0.669 | 6.174 | 5.529 |
| 0 | 28862 | bx041146 | AT3G26040 | HXXXD-type acyl-transferase family protein | 3.309 | 5.636 | 3.337 |
| 0 | 28887 | bx041178 | AT3G25717 | DEVIL 6, ROTUNDIFOLIA like 16 | -1.077 | 1.624 | 2.304 |
| 0 | 28961 | bx041268 | AT4G01575 | serine protease inhibitor, Kazal-type family protein | 2.383 | 3.687 | 2.089 |
| 0 | 28986 | bx041299 |  |  | 2.091 | 3.492 | 2.628 |
| 0 | 29074 | bx041409 | AT5G07990 | TRANSPARENT TESTA 7, CYTOCHROME P450 75B1, Cytochrome P450 superfamily protein | 0.801 | 4.238 | 5.726 |
| 0 | 29089 | bx041424 | AT5G62000 | ORESARA 14, HLS1 SUPPRESSOR, ARF1-BINDING PROTEIN, auxin response factor 2 | 3.972 | 5.473 | 4.940 |
| 0 | 29104 | bx041443 | AT5G61820 | unknown protein | 0.219 | 2.270 | 3.469 |
| 0 | 29107 | bx041446 | AT1G72200 | RING/U-box superfamily protein | 1.118 | 3.544 | 4.559 |
| 0 | 29112 | bx041454 | AT3G48140 | B12D protein | 2.887 | 3.360 | 2.624 |
| 0 | 29153 | bx041509 | AT4G33550 | Bifunctional inhibitor/lipid-transfer protein/seed storage 2S albumin superfamily protein | 4.205 | 4.992 | 3.511 |
| 0 | 29225 | bx041596 |  |  | 0.860 | 3.188 | 3.171 |
| 0 | 29355 | bx041768 | AT2G14580 | basic pathogenesis-related protein 1 | 0.840 | 3.158 | 4.283 |
| 0 | 29386 | bx041812 | AT1G09950 | RESPONSE TO ABA AND SALT 1 | 2.767 | 3.274 | 2.509 |
| 0 | 29504 | bx041964 |  |  | 0.878 | 3.420 | 3.481 |
| 0 | 29631 | bx042123 | AT3G57240 | beta-1,3-glucanase 3, beta-1,3-glucanase 3 | 4.328 | 6.429 | 7.540 |
| 0 | 29664 | bx042164 | AT2G22420 | Peroxidase superfamily protein | 2.455 | 1.865 | 2.497 |
| 0 | 29831 | bx042372 | AT1G20030 | Pathogenesis-related thaumatin superfamily protein | 3.188 | 3.397 | 2.989 |
| 0 | 29852 | bx042396 | AT5G49630 | amino acid permease 6 | 2.119 | 2.540 | 1.749 |
| 0 | 30162 | bx042782 | AT5G27730 | Protein of unknown function (DUF1624) | 0.549 | 2.431 | 1.724 |
| 0 | 30201 | bx042835 | AT4G21380 | receptor kinase 3 | 3.115 | 2.676 | 2.457 |
| 0 | 30232 | bx042871 | AT3G23940 | dehydratase family | 2.638 | 2.916 | 1.892 |
| 0 | 30412 | bx043109 | AT5G64250 | Aldolase-type TIM barrel family protein | 5.755 | 7.950 | 8.056 |
| 0 | 30432 | bx043133 | AT4G32280 | indole-3-acetic acid inducible 29 | 2.656 | 2.945 | 1.945 |
| 0 | 30447 | bx043154 | AT3G21250 | ARABIDOPSIS THALIANA MULTIDRUG RESISTANCE-ASSOCIATED PROTEIN 6, ATP-binding cassette C8, multidrug resistance-associated protein 6 | 2.809 | 2.370 | 2.329 |
| 0 | 30694 | bx043474 |  |  | 0.896 | 3.530 | 3.678 |
| 0 | 30828 | bx043641 | AT1G65840 | polyamine oxidase 4 | 2.119 | 2.419 | 1.856 |
| 0 | 30839 | bx043655 | AT4G21090 | ARABIDOPSIS MITOCHONDRIAL FERREDOXIN 2, MITOCHONDRIAL FERREDOXIN 2 | 1.806 | 2.396 | 1.276 |
| 0 | 30891 | bx043726 | AT5G03670 | unknown protein | 3.209 | 3.182 | 3.307 |
| 0 | 31029 | bx043902 | AT4G21903 | MATE efflux family protein | 2.452 | 2.143 | 1.979 |
| 0 | 31058 | bx043939 | AT1G78780 | pathogenesis-related family protein | 5.419 | 6.380 | 6.075 |
| 0 | 31072 | bx043955 | AT4G29090 | Ribonuclease H-like superfamily protein | 1.896 | 2.806 | 3.656 |
| 0 | 31116 | bx044005 | AT3G26770 | NAD(P)-binding Rossmann-fold superfamily protein | 1.144 | 3.225 | 2.593 |
| 0 | 31130 | bx044021 | AT1G20140 | SKP1-like 4 | 2.343 | 2.263 | 1.840 |
| 0 | 31187 | bx044102 | AT2G39980 | HXXXD-type acyl-transferase family protein | 4.907 | 5.167 | 5.032 |
| 0 | 31246 | bx044179 | AT3G21250 | ARABIDOPSIS THALIANA MULTIDRUG RESISTANCE-ASSOCIATED PROTEIN 6, ATP-binding cassette C8, multidrug resistance-associated protein 6 | 3.091 | 2.467 | 2.401 |
| 0 | 31274 | bx044221 |  |  | 1.029 | 2.473 | 1.571 |
| 0 | 31530 | bx044538 |  |  | 2.908 | 2.798 | 3.046 |
| 0 | 31593 | bx044627 | AT1G22370 | UDP-glucosyl transferase 85A5 | 3.682 | 4.921 | 4.817 |
| 0 | 31654 | bx044697 | AT2G36630 | Sulfite exporter TauE/SafE family protein | 3.393 | 2.517 | 3.027 |
| 0 | 31743 | bx044809 | AT5G55050 | GDSL-like Lipase/Acylhydrolase superfamily protein | 4.720 | 4.764 | 5.006 |
| 0 | 31798 | bx044875 | AT4G26090 | RESISTANT TO P. SYRINGAE 2, NB-ARC domain-containing disease resistance protein | 2.482 | 3.152 | 3.946 |
| 0 | 31906 | bx045014 | AT3G47960 | Major facilitator superfamily protein | 5.100 | 5.110 | 4.382 |
| 0 | 32163 | bx045333 | AT3G26330 | cytochrome P450, family 71, subfamily B, polypeptide 37, cytochrome P450, family 71, subfamily B, polypeptide 37 | 3.955 | 4.627 | 5.061 |
| 0 | 32256 | bx045451 |  |  | -0.398 | 6.691 | 3.736 |
| 0 | 32302 | bx045517 | AT3G29180 | Protein of unknown function (DUF1336) | 2.722 | 2.867 | 2.021 |
| 0 | 32310 | bx045526 |  |  | 2.852 | 2.044 | 2.306 |
| 0 | 32534 | bx045799 |  |  | 3.431 | 3.718 | 2.460 |
| 0 | 32722 | bx046031 | AT1G74240 | Mitochondrial substrate carrier family protein | 2.193 | 2.524 | 1.830 |
| 0 | 33069 | bx046466 | AT2G36800 | UDP-GLUCOSYL TRANSFERASE 73C5, don-glucosyltransferase 1 | 3.096 | 5.079 | 5.056 |
| 0 | 33171 | bx046600 | AT2G18480 | Major facilitator superfamily protein | 6.252 | 6.262 | 6.341 |
| 0 | 33222 | bx046661 | AT4G17500 | ethylene responsive element binding factor 1 | 3.803 | 6.037 | 4.569 |
| 0 | 33273 | bx046724 | AT2G34320 | Polynucleotidyl transferase, ribonuclease H-like superfamily protein | 2.522 | 3.170 | 2.969 |
| 0 | 33343 | bx046821 | AT3G50660 | SUPPRESSOR OF NPH4 2, SHADE AVOIDANCE 1, PARTIALLY SUPPRESSING COI1 INSENSITIVITY TO JA 1, DWARF 4, CYTOCHROME P450 90B1, CLOMAZONE-RESISTANT, Cytochrome P450 superfamily protein | 4.923 | 6.076 | 5.145 |
| 0 | 33456 | bx046952 | AT2G26440 | Plant invertase/pectin methylesterase inhibitor superfamily | 1.381 | 2.314 | 1.567 |
| 0 | 33469 | bx046969 | AT5G21940 | unknown protein | 1.411 | 1.979 | 2.629 |
| 0 | 33470 | bx046970 | AT5G13080 | ARABIDOPSIS THALIANA WRKY DNA-BINDING PROTEIN 75, WRKY DNA-binding protein 75 | 4.737 | 4.914 | 4.520 |
| 0 | 33501 | bx047007 | AT5G05320 | FAD/NAD(P)-binding oxidoreductase family protein | 0.902 | 2.482 | 3.261 |
| 0 | 33508 | bx047017 |  |  | 1.763 | 3.989 | 4.735 |
| 0 | 33617 | bx047156 | AT5G25930 | Protein kinase family protein with leucine-rich repeat domain | 3.760 | 4.065 | 2.855 |
| 0 | 33675 | bx047225 |  |  | 0.921 | 2.442 | 1.873 |
| 0 | 33676 | bx047226 |  |  | 5.677 | 8.581 | 6.863 |
| 0 | 33719 | bx047277 | AT5G25610 | RESPONSIVE TO DESSICATION 22, BURP domain-containing protein | 3.246 | 4.137 | 3.438 |
| 0 | 33881 | bx047478 | AT5G60900 | receptor-like protein kinase 1 | 2.815 | 3.173 | 3.937 |
| 0 | 33895 | bx047502 | AT3G55710 | UDP-Glycosyltransferase superfamily protein | 1.127 | 2.805 | 3.791 |
| 0 | 33921 | bx047532 |  |  | 1.520 | 2.581 | 2.049 |
| 0 | 33929 | bx047540 | AT3G30210 | myb domain protein 121 | 2.976 | 2.928 | 3.509 |
| 0 | 33947 | bx047559 |  |  | 1.931 | 4.060 | 3.941 |
| 0 | 33984 | bx047617 |  |  | 1.211 | 2.534 | 1.163 |
| 0 | 34015 | bx047655 | AT5G60900 | receptor-like protein kinase 1 | 2.940 | 3.079 | 3.865 |
| 0 | 34051 | bx047703 |  |  | 1.365 | 2.968 | 2.886 |
| 0 | 34153 | bx047827 | AT1G67265 | DEVIL 3, ROTUNDIFOLIA like 21 | 4.212 | 3.927 | 3.225 |
| 0 | 34192 | bx047876 | AT5G57110 | AUTOINHIBITED CA2+ -ATPASE, ISOFORM 8, autoinhibited Ca2+ -ATPase, isoform 8, autoinhibited Ca2+ -ATPase, isoform 8 | 1.485 | 1.130 | 2.973 |
| 0 | 34259 | bx047958 |  |  | 1.534 | 3.269 | 2.389 |
| 0 | 34423 | bx048161 |  |  | 3.740 | 4.401 | 3.819 |
| 0 | 34456 | bx048208 | AT3G26590 | MATE efflux family protein | 0.979 | 2.544 | 2.199 |
| 0 | 34513 | bx048273 | AT1G07180 | alternative NAD(P)H dehydrogenase 1, ARABIDOPSIS THALIANA INTERNAL NON-PHOSPHORYLATING NAD ( P ) H DEHYDROGENASE, alternative NAD(P)H dehydrogenase 1 | 3.467 | 4.637 | 2.556 |
| 0 | 34636 | bx048424 | AT5G34930 | arogenate dehydrogenase | 2.255 | 3.217 | 3.208 |
| 0 | 34734 | bx048539 | AT5G13110 | glucose-6-phosphate dehydrogenase 2 | 0.970 | 3.210 | 3.422 |
| 0 | 34750 | bx048557 |  |  | 3.489 | 3.963 | 4.038 |
| 0 | 34760 | bx048572 | AT5G11730 | Core-2/I-branching beta-1,6-N-acetylglucosaminyltransferase family protein | 3.892 | 4.418 | 3.327 |
| 0 | 34932 | bx048786 |  |  | 0.709 | 3.547 | 3.502 |
| 0 | 34983 | bx048857 |  |  | 0.039 | 2.032 | 2.568 |
| 0 | 35090 | bx048995 | AT5G07990 | TRANSPARENT TESTA 7, CYTOCHROME P450 75B1, Cytochrome P450 superfamily protein | 1.730 | 2.814 | 1.829 |
| 0 | 35099 | bx049005 | AT1G29860 | WRKY DNA-binding protein 71 | 0.798 | 2.420 | 1.262 |
| 0 | 35154 | bx049075 |  |  | 2.646 | 4.918 | 3.397 |
| 0 | 35188 | bx049116 | AT5G14700 | NAD(P)-binding Rossmann-fold superfamily protein | 0.187 | 2.996 | 3.882 |
| 0 | 35227 | bx049160 |  |  | 1.302 | 3.751 | 3.233 |
| 0 | 35228 | bx049161 | AT2G37770 | Chloroplastic aldo-keto reductase, Aldo-keto reductase family 4 member C9, NAD(P)-linked oxidoreductase superfamily protein | 1.649 | 4.711 | 3.302 |
| 0 | 35242 | bx049178 | AT4G02510 | TRANSLOCON AT THE OUTER ENVELOPE MEMBRANE OF CHLOROPLASTS 86, TRANSLOCON AT THE OUTER ENVELOPE MEMBRANE OF CHLOROPLASTS 160, PLASTID PROTEIN IMPORT 2, translocon at the outer envelope membrane of chloroplasts 159 | 2.570 | 2.211 | 1.949 |
| 0 | 35322 | bx049296 | AT3G51430 | YELLOW-LEAF-SPECIFIC GENE 2, STRICTOSIDINE SYNTHASE-LIKE 5, Calcium-dependent phosphotriesterase superfamily protein | 2.031 | 2.849 | 1.583 |
| 0 | 35414 | bx049408 |  |  | 2.867 | 3.482 | 3.269 |
| 0 | 35451 | bx049450 | AT3G24240 | Leucine-rich repeat receptor-like protein kinase family protein | 3.313 | 5.091 | 3.331 |
| 0 | 35483 | bx049494 | AT2G36800 | UDP-GLUCOSYL TRANSFERASE 73C5, don-glucosyltransferase 1 | 1.998 | 3.781 | 4.071 |
| 0 | 35934 | bx050105 | AT2G25735 | unknown protein | 3.726 | 3.322 | 2.941 |
| 0 | 36023 | bx050211 | AT5G35735 | Auxin-responsive family protein | 2.702 | 2.923 | 2.146 |
| 0 | 36047 | bx050243 | AT1G69790 | Protein kinase superfamily protein | 2.044 | 2.704 | 2.166 |
| 0 | 36087 | bx050295 | AT1G26780 | LATERAL ORGAN FUSION 1, myb domain protein 117 | 2.633 | 3.701 | 3.492 |
| 0 | 36115 | bx050330 | AT3G59900 | auxin-regulated gene involved in organ size | 2.082 | 2.491 | 2.074 |
| 0 | 36242 | bx050470 | AT3G01930 | Major facilitator superfamily protein | 1.605 | 1.999 | 2.650 |
| 0 | 36260 | bx050492 | AT1G66980 | Glycerophosphodiester phosphodiesterase (GDPD) like 2, suppressor of npr1-1 constitutive 4 | 3.330 | 3.754 | 2.808 |
| 0 | 36332 | bx050587 | AT3G10450 | serine carboxypeptidase-like 7 | 3.050 | 5.988 | 4.830 |
| 0 | 36494 | bx050787 |  |  | 0.022 | 2.244 | 3.268 |
| 0 | 36664 | bx051001 | AT5G52450 | MATE efflux family protein | 2.621 | 2.563 | 2.210 |
| 0 | 36677 | bx051016 | AT1G29860 | WRKY DNA-binding protein 71 | 1.081 | 1.723 | 2.453 |
| 0 | 36682 | bx051022 | AT1G05675 | UDP-Glycosyltransferase superfamily protein | 1.755 | 4.424 | 5.029 |
| 0 | 36955 | bx051375 | AT1G73830 | BR enhanced expression 3 | 1.533 | 3.389 | 3.386 |
| 0 | 37001 | bx051436 | AT1G75250 | RADIALIS-LIKE SANT/MYB 3, RAD-like 6 | 1.359 | 2.708 | 2.095 |
| 0 | 37123 | bx051607 | AT1G55230 | Family of unknown function (DUF716) | 6.559 | 7.033 | 6.640 |
| 0 | 37158 | bx051649 | AT2G36630 | Sulfite exporter TauE/SafE family protein | 3.482 | 2.961 | 3.229 |
| 0 | 37195 | bx051692 | AT3G59900 | auxin-regulated gene involved in organ size | 4.298 | 5.098 | 4.452 |
| 0 | 37425 | bx051966 | AT1G17020 | senescence-related gene 1 | 1.331 | 4.195 | 4.634 |
| 0 | 37983 | bx052699 | AT1G15520 | Arabidopsis thaliana ATP-binding cassette G40, ATP-binding cassette G40, pleiotropic drug resistance 12 | 1.718 | 2.937 | 1.964 |
| 0 | 38219 | bx052996 |  |  | 1.869 | 2.313 | 2.644 |
| 0 | 38233 | bx053011 | AT3G63010 | GA INSENSITIVE DWARF1B, alpha/beta-Hydrolases superfamily protein | 2.371 | 2.131 | 2.175 |
| 0 | 38281 | bx053072 | AT5G24090 | chitinase A | 6.162 | 8.166 | 7.945 |
| 0 | 38296 | bx053097 | AT4G02280 | sucrose synthase 3 | 0.809 | 1.102 | 2.434 |
| 0 | 38299 | bx053101 | AT2G47800 | ATP-binding cassette C4, multidrug resistance-associated protein 4 | 2.064 | 2.467 | 1.637 |
| 0 | 38330 | bx053138 |  |  | 2.226 | 3.394 | 3.256 |
| 0 | 38370 | bx053192 | AT1G52565 | unknown protein | 0.235 | 1.062 | 2.523 |
| 0 | 38465 | bx053314 | AT3G55880 | sulphate utilization efficiency 4, Alpha/beta hydrolase related protein | 2.892 | 2.670 | 2.276 |
| 0 | 38512 | bx053383 |  |  | 2.101 | 2.492 | 2.714 |
| 0 | 38796 | bx053744 | AT1G22410 | Class-II DAHP synthetase family protein | 3.157 | 2.491 | 3.620 |
| 0 | 38977 | bx053979 |  |  | 0.481 | 3.672 | 2.953 |
| 0 | 39057 | bx054083 | AT5G13080 | ARABIDOPSIS THALIANA WRKY DNA-BINDING PROTEIN 75, WRKY DNA-binding protein 75 | 6.344 | 7.103 | 6.217 |
| 0 | 39078 | bx054111 | AT1G66920 | Protein kinase superfamily protein | 3.670 | 4.689 | 3.706 |
| 0 | 39440 | bx054629 | AT5G05870 | UDP-glucosyl transferase 76C1 | 3.155 | 3.860 | 3.356 |
| 0 | 39573 | bx054818 | AT1G74240 | Mitochondrial substrate carrier family protein | 2.217 | 2.610 | 1.969 |
| 0 | 39644 | bx054918 | AT5G52390 | PAR1 protein | 1.803 | 3.168 | 2.267 |
| 0 | 39658 | bx054937 | AT1G68810 | basic helix-loop-helix (bHLH) DNA-binding superfamily protein | 2.448 | 3.327 | 3.696 |
| 0 | 39725 | bx055060 | AT2G29220 | Concanavalin A-like lectin protein kinase family protein | 1.966 | 3.384 | 3.771 |
| 0 | 39780 | bx055135 | AT5G60280 | Concanavalin A-like lectin protein kinase family protein | 0.737 | 2.352 | 0.918 |
| 0 | 39900 | bx055293 | AT4G37990 | CINNAMYL-ALCOHOL DEHYDROGENASE B2, ARABIDOPSIS THALIANA CINNAMYL-ALCOHOL DEHYDROGENASE 8, elicitor-activated gene 3-2 | 2.398 | 3.155 | 2.126 |
| 0 | 40130 | bx055595 | AT3G26300 | cytochrome P450, family 71, subfamily B, polypeptide 34, cytochrome P450, family 71, subfamily B, polypeptide 34 | 3.233 | 4.071 | 3.734 |
| 0 | 40194 | bx055672 |  |  | 3.874 | 5.799 | 5.852 |
| 0 | 40219 | bx055704 | AT5G13650 | SUPPRESSOR OF VARIEGATION 3, elongation factor family protein | 1.464 | 2.393 | 2.122 |
| 0 | 40220 | bx055708 | AT5G47610 | RING/U-box superfamily protein | -0.704 | 0.857 | 2.604 |
| 0 | 40393 | bx055913 |  |  | 1.934 | 4.010 | 2.444 |
| 0 | 41014 | bx056682 | AT4G23100 | ROOT MERISTEMLESS 1, PHYTOALEXIN DEFICIENT 2, CADMIUM SENSITIVE 2, glutamate-cysteine ligase | 1.792 | 2.339 | 1.556 |
| 0 | 41155 | bx056843 | AT5G03710 | unknown protein | 0.480 | 2.505 | 3.606 |
| 0 | 41160 | bx056851 | AT4G18210 | purine permease 10 | 0.667 | 1.638 | 2.489 |
| 0 | 41334 | bx057067 |  |  | 2.947 | 3.085 | 2.329 |
| 0 | 41399 | bx057142 | AT5G12890 | UDP-Glycosyltransferase superfamily protein | 3.738 | 4.509 | 3.732 |
| 0 | 41519 | bx057296 | AT1G08080 | A. THALIANA ALPHA CARBONIC ANHYDRASE 7, alpha carbonic anhydrase 7 | 4.158 | 4.583 | 3.326 |
| 0 | 41586 | bx057381 | AT1G73300 | serine carboxypeptidase-like 2 | 2.474 | 6.620 | 4.692 |
| 0 | 41619 | bx057421 | AT1G61260 | Protein of unknown function (DUF761) | 3.073 | 3.184 | 2.455 |
| 0 | 42045 | bx057923 | AT5G39130 | RmlC-like cupins superfamily protein | 1.393 | 5.507 | 5.055 |
| 0 | 42149 | bx058046 | AT5G13110 | glucose-6-phosphate dehydrogenase 2 | 1.020 | 2.814 | 2.539 |
| 0 | 42305 | bx058247 |  |  | -0.021 | 2.153 | 2.441 |
| 0 | 42394 | bx058351 | AT5G16620 | pigment defective embryo 120, translocon at the inner envelope membrane of chloroplasts 40, hydroxyproline-rich glycoprotein family protein | 0.805 | 2.862 | 2.657 |
| 0 | 42431 | bx058392 | AT1G29630 | 5'-3' exonuclease family protein | 0.865 | 3.228 | 3.285 |
| 0 | 42527 | bx058513 | AT2G42140 | VQ motif-containing protein | 2.688 | 2.273 | 2.334 |
| 0 | 42587 | bx058590 | AT3G23240 | ethylene response factor 1 | 4.219 | 3.345 | 4.402 |
| 0 | 42625 | bx058634 | AT5G23810 | amino acid permease 7 | 1.335 | 2.313 | 1.975 |
| 0 | 42805 | bx058860 | AT2G24010 | serine carboxypeptidase-like 23 | 0.517 | 3.808 | 3.280 |
| 0 | 42905 | bx058988 |  |  | 1.487 | 3.891 | 3.274 |
| 0 | 43098 | bx059224 | AT1G11410 | S-locus lectin protein kinase family protein | 1.906 | 3.033 | 2.477 |
| 0 | 43119 | bx059247 | AT1G78780 | pathogenesis-related family protein | 4.896 | 4.959 | 4.914 |
| 0 | 43125 | bx059255 | AT5G09640 | SINAPOYLGLUCOSE ACCUMULATOR 2, serine carboxypeptidase-like 19 | 1.751 | 3.615 | 3.666 |
| 0 | 43152 | bx059287 | AT1G49320 | unknown seed protein like 1 | 2.217 | 3.391 | 4.704 |
| 0 | 43179 | bx059322 | AT3G13080 | MULTIDRUG RESISTANCE PROTEIN 3, ATP-binding cassette C3, multidrug resistance-associated protein 3 | 3.015 | 2.486 | 2.463 |
| 0 | 43305 | bx059468 | AT1G50420 | scarecrow-like 3 | 4.011 | 3.599 | 3.607 |
| 0 | 43344 | bx059519 | AT1G34300 | lectin protein kinase family protein | 1.075 | 2.678 | 2.282 |
| 0 | 43560 | bx059799 | AT4G35190 | LONELY GUY 5, Putative lysine decarboxylase family protein | 3.437 | 4.608 | 2.944 |
| 0 | 43761 | bx060050 | AT5G53970 | tyrosine aminotransferase 7, Tyrosine transaminase family protein | 4.746 | 5.719 | 4.769 |
| 0 | 43813 | bx060108 | AT5G53110 | RING/U-box superfamily protein | 3.129 | 4.136 | 3.303 |
| 0 | 43940 | bx060268 | AT1G12910 | LIGHT-REGULATED WD 1, ANTHOCYANIN11, Transducin/WD40 repeat-like superfamily protein | 0.567 | 2.250 | 3.041 |
| 0 | 44081 | bx060440 | AT5G35550 | TRANSPARENT TESTA 2, MYB DOMAIN PROTEIN 123, Duplicated homeodomain-like superfamily protein | 1.360 | 3.702 | 4.751 |
| 0 | 44087 | bx060447 | AT5G19600 | sulfate transporter 3;5 | 2.861 | 2.893 | 2.080 |
| 0 | 44452 | bx060886 | AT1G17710 | Arabidopsis thaliana phosphoethanolamine/phosphocholine phosphatase 1, Pyridoxal phosphate phosphatase-related protein | 2.207 | 2.862 | 2.765 |
| 0 | 44711 | bx061198 |  |  | 1.382 | 2.689 | 2.805 |
| 0 | 44989 | bx061543 | AT4G11650 | osmotin 34 | 0.879 | 5.370 | 6.714 |
| 0 | 45026 | bx061588 | AT2G02580 | cytochrome P450, family 71, subfamily B, polypeptide 9, cytochrome P450, family 71, subfamily B, polypeptide 9 | 0.907 | 2.680 | 2.478 |
| 0 | 45080 | bx061658 |  |  | 4.048 | 5.653 | 4.372 |
| 0 | 45105 | bx061693 | AT5G13080 | ARABIDOPSIS THALIANA WRKY DNA-BINDING PROTEIN 75, WRKY DNA-binding protein 75 | 5.307 | 5.971 | 5.818 |
| 0 | 45148 | bx061746 | AT4G27590 | Heavy metal transport/detoxification superfamily protein | 3.193 | 3.456 | 2.886 |
| 0 | 45197 | bx061804 | AT3G06490 | BOTRYTIS-SUSCEPTIBLE1, myb domain protein 108 | 1.335 | 3.968 | 4.179 |
| 0 | 45210 | bx061822 | AT3G60890 | LITTLE ZIPPER 2, protein binding | 4.343 | 3.656 | 3.542 |
| 0 | 45231 | bx061846 | AT1G09950 | RESPONSE TO ABA AND SALT 1 | 4.391 | 4.036 | 3.642 |
| 0 | 45232 | bx061847 | AT3G61460 | brassinosteroid-responsive RING-H2 | 3.893 | 2.745 | 3.120 |
| 0 | 45282 | bx061909 | AT3G45440 | Concanavalin A-like lectin protein kinase family protein | 2.550 | 2.799 | 2.077 |
| 0 | 45548 | bx062229 |  |  | 0.371 | 1.581 | 3.005 |
| 0 | 45669 | bx062380 | AT2G41350 | EMBRYO DEFECTIVE 2819, augmin 1, unknown protein | 0.686 | 3.540 | 3.290 |
| 0 | 45893 | bx062687 | AT1G07530 | ARABIDOPSIS THALIANA GRAS (GAI, RGA, SCR) 2, SCARECROW-like 14 | 5.884 | 5.917 | 5.012 |
| 0 | 46516 | bx063484 | AT4G21380 | receptor kinase 3 | 1.823 | 2.706 | 1.682 |
| 0 | 46618 | bx063606 | AT2G37770 | Chloroplastic aldo-keto reductase, Aldo-keto reductase family 4 member C9, NAD(P)-linked oxidoreductase superfamily protein | 3.522 | 6.307 | 4.335 |
| 0 | 46706 | bx063709 | AT1G15520 | Arabidopsis thaliana ATP-binding cassette G40, ATP-binding cassette G40, pleiotropic drug resistance 12 | 1.837 | 3.021 | 1.910 |
| 0 | 46801 | bx063817 | AT1G65840 | polyamine oxidase 4 | 2.532 | 3.116 | 2.181 |
| 0 | 46821 | bx063838 | AT5G48100 | TRANSPARENT TESTA 10, LACCASE-LIKE 15, Laccase/Diphenol oxidase family protein | 1.726 | 3.397 | 3.115 |
| 0 | 46861 | bx063894 |  |  | 2.182 | 4.251 | 1.591 |
| 0 | 47015 | bx064067 | AT2G37710 | receptor lectin kinase | 3.039 | 3.191 | 2.971 |
| 0 | 47054 | bx064109 | AT4G08850 | Leucine-rich repeat receptor-like protein kinase family protein | 3.681 | 4.371 | 3.491 |
| 0 | 47088 | bx064148 |  |  | 2.839 | 4.937 | 3.450 |
| 0 | 47225 | bx064302 |  |  | 4.166 | 4.358 | 3.719 |
| 0 | 47317 | bx064403 | AT1G70740 | Protein kinase superfamily protein | 0.594 | 3.324 | 2.142 |
| 0 | 47467 | bx064580 |  |  | 3.373 | 2.645 | 3.455 |
| 0 | 47582 | bx064707 | AT1G19180 | jasmonate-zim-domain protein 1 | 3.368 | 5.159 | 3.927 |
| 0 | 47809 | bx064957 | AT1G11340 | S-locus lectin protein kinase family protein | 3.594 | 4.717 | 4.162 |
| 0 | 47884 | bx065042 |  |  | 1.482 | 3.494 | 2.955 |
| 0 | 48190 | bx065390 | AT1G47485 | unknown protein | 5.902 | 5.145 | 6.425 |
| 0 | 48621 | bx065940 |  |  | 2.590 | 3.540 | 2.614 |
| 0 | 48641 | bx065965 | AT3G11840 | plant U-box 24 | 3.402 | 2.550 | 2.600 |
| 0 | 48684 | bx066018 | AT3G15115 | unknown protein | 2.428 | 2.347 | 2.588 |
| 0 | 48770 | bx066133 |  |  | 1.844 | 2.488 | 1.665 |
| 0 | 49070 | bx066520 |  |  | 0.848 | 3.315 | 3.229 |
| 0 | 49168 | bx066641 | AT1G71140 | MATE efflux family protein | 2.325 | 2.137 | 1.721 |
| 0 | 49177 | bx066650 |  |  | 0.699 | 1.458 | 2.586 |
| 0 | 49243 | bx066725 | AT4G32300 | S-domain-2 5 | 2.063 | 3.456 | 3.604 |
| 0 | 49245 | bx066728 |  |  | 1.652 | 4.395 | 5.072 |
| 0 | 49264 | bx066751 | AT1G51410 | NAD(P)-binding Rossmann-fold superfamily protein | 0.989 | 3.002 | 1.410 |
| 0 | 49292 | bx066784 | AT2G44080 | ARGOS-like | 5.276 | 4.584 | 5.256 |
| 0 | 49436 | bx066949 | AT3G57270 | beta-1,3-glucanase 1, beta-1,3-glucanase 1 | 4.844 | 6.314 | 7.084 |
| 0 | 49686 | bx067245 |  |  | -0.071 | 0.946 | 2.307 |
| 0 | 49747 | bx067320 | AT3G56620 | nodulin MtN21 /EamA-like transporter family protein | 0.748 | 3.076 | 2.634 |
| 0 | 50010 | bx067622 |  |  | 0.866 | 3.846 | 3.900 |
| 0 | 50068 | bx067692 |  |  | 1.396 | 3.810 | 2.858 |
| 0 | 50136 | bx067768 |  |  | 0.021 | 0.534 | 2.664 |
| 0 | 50392 | bx068054 | AT5G05960 | Bifunctional inhibitor/lipid-transfer protein/seed storage 2S albumin superfamily protein | 1.606 | 4.306 | 3.498 |
| 0 | 50812 | bx068573 | AT3G11210 | SGNH hydrolase-type esterase superfamily protein | 2.935 | 4.159 | 4.310 |
| 0 | 51015 | bx068809 | AT1G33080 | MATE efflux family protein | 3.815 | 3.981 | 4.552 |
| 0 | 51733 | bx069856 |  |  | 5.092 | 6.035 | 4.445 |
| 0 | 51859 | bx070061 | AT5G22300 | nitrilase 4 | 2.565 | 3.846 | 1.967 |
| 0 | 51889 | bx070109 |  |  | 0.850 | 2.598 | 2.124 |
| 0 | 51975 | bx070228 |  |  | 2.969 | 3.619 | 2.792 |
| 0 | 51980 | bx070234 | AT5G36930 | Disease resistance protein (TIR-NBS-LRR class) family | 0.955 | 3.226 | 1.568 |
| 0 | 52280 | bx070634 | AT4G12910 | serine carboxypeptidase-like 20 | -0.087 | 1.869 | 2.476 |
| 0 | 52415 | bx070878 | AT1G73010 | pyrophosphate-specific phosphatase1, phosphate starvation-induced gene 2 | 2.025 | 2.748 | 2.673 |
| 0 | 52473 | bx070957 | AT3G06880 | Transducin/WD40 repeat-like superfamily protein | 2.662 | 3.610 | 2.668 |
| 0 | 52569 | bx071095 |  |  | 2.590 | 3.941 | 2.665 |
| 0 | 52608 | bx071157 | AT4G16260 | Glycosyl hydrolase superfamily protein | 2.585 | 3.654 | 3.468 |
| 0 | 52638 | bx071204 | AT2G41230 | ORGAN SIZE RELATED 1, unknown protein | 5.723 | 7.582 | 6.424 |
| 0 | 52761 | bx071402 |  |  | 2.246 | 2.346 | 2.971 |
| 0 | 52764 | bx071409 |  |  | 2.902 | 3.917 | 3.199 |
| 0 | 52913 | bx071641 |  |  | 3.091 | 3.558 | 3.086 |
| 0 | 53172 | bx071930 | AT1G53430 | Leucine-rich repeat transmembrane protein kinase | 0.618 | 3.081 | 1.986 |
| 0 | 53514 | bx072313 | AT3G21250 | ARABIDOPSIS THALIANA MULTIDRUG RESISTANCE-ASSOCIATED PROTEIN 6, ATP-binding cassette C8, multidrug resistance-associated protein 6 | 2.977 | 2.727 | 2.443 |
| 0 | 53626 | bx072455 |  |  | 3.014 | 3.386 | 2.630 |
| 0 | 53635 | bx072464 | AT3G21250 | ARABIDOPSIS THALIANA MULTIDRUG RESISTANCE-ASSOCIATED PROTEIN 6, ATP-binding cassette C8, multidrug resistance-associated protein 6 | 3.224 | 3.056 | 2.601 |
| 0 | 53647 | bx072480 |  |  | 2.881 | 2.507 | 2.692 |
| 0 | 53658 | bx072495 | AT5G05320 | FAD/NAD(P)-binding oxidoreductase family protein | 1.281 | 1.958 | 2.441 |
| 0 | 53846 | bx072720 |  |  | 0.982 | 2.230 | 2.622 |
| 0 | 53853 | bx072729 |  |  | 2.649 | 3.855 | 2.883 |
| 0 | 53854 | bx072730 |  |  | 2.939 | 3.271 | 3.666 |
| 0 | 53944 | bx072835 | AT2G21340 | MATE efflux family protein | 1.973 | 2.410 | 2.748 |
| 0 | 54985 | bx073995 | AT3G47570 | Leucine-rich repeat protein kinase family protein | 2.690 | 2.824 | 2.180 |
| 0 | 55012 | bx074026 | AT1G50610 | Leucine-rich repeat protein kinase family protein | 2.507 | 2.280 | 2.365 |
| 0 | 55248 | bx074324 |  |  | 2.601 | 3.080 | 3.590 |
| 0 | 55343 | bx074452 | AT1G69490 | Arabidopsis NAC domain containing protein 29, NAC-like, activated by AP3/PI | 1.118 | 2.187 | 2.972 |
| 0 | 55421 | bx074553 | AT3G11340 | UDP-dependent glycosyltransferase 76B1, UDP-Glycosyltransferase superfamily protein | 6.671 | 7.927 | 7.260 |
| 0 | 55428 | bx074562 | AT3G53880 | Aldo-keto reductase family 4 member C11, NAD(P)-linked oxidoreductase superfamily protein | 3.232 | 6.531 | 4.355 |
| 0 | 55438 | bx074578 | AT5G24910 | EUI-like p450 A1, cytochrome P450, family 714, subfamily A, polypeptide 1 | 1.244 | 4.188 | 3.568 |
| 0 | 55475 | bx074626 |  |  | 0.283 | 1.099 | 2.352 |
| 0 | 55563 | bx074730 |  |  | 2.298 | 2.459 | 1.948 |
| 0 | 55624 | bx074813 |  |  | 1.785 | 1.996 | 2.917 |
| 0 | 55660 | bx074864 |  |  | 2.086 | 3.557 | 2.986 |
| 0 | 55695 | bx074936 | AT4G31950 | cytochrome P450, family 82, subfamily C, polypeptide 3, cytochrome P450, family 82, subfamily C, polypeptide 3 | 2.962 | 4.667 | 3.243 |
| 0 | 55730 | bx075024 |  |  | 2.519 | 2.852 | 3.549 |
| 0 | 55927 | bx075311 | AT4G11530 | cysteine-rich RLK (RECEPTOR-like protein kinase) 34 | 2.184 | 2.425 | 1.717 |
| 0 | 55938 | bx075328 |  |  | 0.763 | 3.119 | 3.124 |
| 0 | 55997 | bx075415 | AT3G45410 | Concanavalin A-like lectin protein kinase family protein | 2.712 | 2.717 | 1.978 |
| 0 | 56027 | bx075480 |  |  | 7.368 | 7.335 | 5.729 |
| 0 | 56142 | bx075632 | AT3G06490 | BOTRYTIS-SUSCEPTIBLE1, myb domain protein 108 | 3.308 | 4.732 | 4.665 |
| 0 | 56223 | bx075735 | AT2G17220 | kinase 3, Protein kinase superfamily protein | 1.894 | 2.493 | 2.585 |
| 0 | 56248 | bx075769 | AT5G06270 | unknown protein | 2.901 | 3.012 | 2.186 |
| 0 | 56254 | bx075778 | AT2G24180 | cytochrome p450 71b6 | 2.925 | 3.009 | 2.348 |
| 0 | 56275 | bx075804 | AT1G33540 | serine carboxypeptidase-like 18 | 1.307 | 4.903 | 4.078 |
| 0 | 56333 | bx075900 | AT4G38540 | FAD/NAD(P)-binding oxidoreductase family protein | 1.366 | 3.086 | 4.703 |
| 0 | 56336 | bx075904 | AT4G27290 | S-locus lectin protein kinase family protein | 3.411 | 3.780 | 2.602 |
| 0 | 56351 | bx075925 |  |  | 1.253 | 3.805 | 2.416 |
| 0 | 56354 | bx075929 |  |  | 2.615 | 3.927 | 5.607 |
| 0 | 56396 | bx075977 | AT1G13280 | allene oxide cyclase 4 | 2.915 | 4.798 | 4.656 |
| 0 | 56478 | bx076076 | AT5G17760 | P-loop containing nucleoside triphosphate hydrolases superfamily protein | 2.160 | 2.459 | 1.569 |
| 0 | 56594 | bx076206 |  |  | 0.866 | 3.186 | 3.208 |
| 0 | 56672 | bx076298 |  |  | 2.405 | 3.374 | 1.673 |
| 0 | 56727 | bx076362 | AT2G39130 | Transmembrane amino acid transporter family protein | -1.486 | 1.275 | 2.342 |
| 0 | 56794 | bx076431 |  |  | 2.357 | 3.675 | 1.773 |
| 0 | 56829 | bx076467 |  |  | 1.823 | 2.572 | 2.117 |
| 0 | 56873 | bx076516 |  |  | 2.970 | 2.705 | 3.641 |
| 0 | 56923 | bx076572 |  |  | 2.517 | 2.742 | 1.925 |
| 0 | 56967 | bx076617 |  |  | 3.539 | 4.582 | 4.430 |
| 0 | 56968 | bx076618 |  |  | 2.970 | 3.325 | 3.690 |
| 0 | 57033 | bx076690 | AT1G55850 | cellulose synthase like E1 | 1.642 | 3.757 | 2.488 |
| 0 | 57285 | bx077006 |  |  | 4.370 | 4.689 | 3.187 |
| 0 | 57923 | bx077810 | AT4G22070 | WRKY DNA-binding protein 31 | 1.794 | 3.121 | 3.727 |
| 0 | 58104 | bx078038 | AT5G39110 | RmlC-like cupins superfamily protein | 6.375 | 9.475 | 8.387 |
| 0 | 58119 | bx078053 | AT5G53980 | homeobox protein 52 | 2.669 | 4.030 | 3.940 |
| 0 | 58752 | bx078789 |  |  | 3.487 | 4.816 | 3.696 |
| 0 | 58871 | bx078938 | AT3G11210 | SGNH hydrolase-type esterase superfamily protein | 4.029 | 4.869 | 4.872 |
| 0 | 58967 | bx079052 |  |  | 2.139 | 2.667 | 2.912 |
| 0 | 59099 | bx079207 | AT5G24080 | Protein kinase superfamily protein | 1.114 | 2.518 | 2.798 |
| 0 | 59119 | bx079230 | AT3G23250 | myb domain protein 15 | 3.986 | 5.718 | 3.082 |
| 0 | 59304 | bx079445 |  |  | 1.439 | 0.833 | 2.324 |
| 0 | 59337 | bx079480 |  |  | 2.185 | 2.886 | 2.076 |
| 0 | 59473 | bx079637 | AT4G23130 | RECEPTOR-LIKE PROTEIN KINASE 6, cysteine-rich RLK (RECEPTOR-like protein kinase) 5 | 3.798 | 5.512 | 5.353 |
| 0 | 59496 | bx079666 | AT2G41820 | Leucine-rich repeat protein kinase family protein | 1.950 | 2.543 | 2.345 |
| 0 | 60416 | bx080838 | AT5G19600 | sulfate transporter 3;5 | 0.581 | 4.229 | 3.869 |
| 0 | 60488 | bx080927 | AT5G62230 | ERECTA-like 1 | 1.895 | 3.217 | 2.309 |
| 0 | 60530 | bx080982 | AT1G02205 | ECERIFERUM 1, Fatty acid hydroxylase superfamily | 2.608 | 4.306 | 3.605 |
| 0 | 60596 | bx081063 |  |  | 2.613 | 2.799 | 1.903 |
| 0 | 60616 | bx081091 |  |  | 1.530 | 3.445 | 3.192 |
| 0 | 60772 | bx081277 | AT4G38070 | basic helix-loop-helix (bHLH) DNA-binding superfamily protein | 2.735 | 2.550 | 2.022 |
| 0 | 60812 | bx081324 | AT1G51920 | unknown protein | 3.495 | 5.551 | 4.121 |
| 0 | 60836 | bx081354 | AT4G23400 | plasma membrane intrinsic protein 1;5 | 0.976 | 2.422 | 2.863 |
| 0 | 60876 | bx081406 | AT3G10450 | serine carboxypeptidase-like 7 | 3.252 | 6.028 | 5.062 |
| 0 | 61101 | bx081687 |  |  | 2.188 | 2.649 | 2.436 |
| 0 | 61222 | bx081854 | AT3G45330 | Concanavalin A-like lectin protein kinase family protein | 2.268 | 3.344 | 3.046 |
| 0 | 61464 | bx082221 | AT5G38970 | brassinosteroid-6-oxidase 1 | 2.368 | 3.458 | 3.644 |
| 1 | 161 | bx000299 | AT4G01850 | S-adenosylmethionine synthetase 2 | 2.685 | 2.501 | 0.139 |
| 1 | 179 | bx000329 |  |  | 2.754 | 3.122 | 1.345 |
| 1 | 199 | bx000364 | AT1G22930 | T-complex protein 11 | 2.890 | 2.783 | 1.056 |
| 1 | 345 | bx000616 | AT4G11570 | Haloacid dehalogenase-like hydrolase (HAD) superfamily protein | 2.355 | 1.963 | 1.275 |
| 1 | 455 | bx000811 | AT5G24105 | arabinogalactan protein 41 | 4.281 | 4.022 | 1.699 |
| 1 | 501 | bx000883 | AT1G51760 | JASMONIC ACID RESPONSIVE 3, IAA-ALANINE RESISTANT 3, peptidase M20/M25/M40 family protein | 2.896 | 2.639 | 0.932 |
| 1 | 565 | bx000999 | AT3G22560 | Acyl-CoA N-acyltransferases (NAT) superfamily protein | 3.394 | 3.514 | 2.286 |
| 1 | 582 | bx001026 | AT2G36020 | HVA22-like protein J | 2.642 | 1.833 | 1.419 |
| 1 | 709 | bx001257 | AT3G27320 | alpha/beta-Hydrolases superfamily protein | 3.086 | 1.908 | 0.618 |
| 1 | 857 | bx001493 | AT1G32928 | unknown protein | 2.371 | 2.172 | 0.398 |
| 1 | 952 | bx001641 | AT5G17540 | HXXXD-type acyl-transferase family protein | 2.652 | 1.881 | 0.153 |
| 1 | 969 | bx001673 | AT1G10740 | alpha/beta-Hydrolases superfamily protein | 2.352 | -0.083 | -0.281 |
| 1 | 991 | bx001704 | AT1G78380 | GLUTATHIONE TRANSFERASE 8, A. THALIANA GLUTATHIONE S-TRANSFERASE TAU 19, glutathione S-transferase TAU 19 | 6.099 | 5.910 | 4.137 |
| 1 | 1080 | bx001842 | AT5G47530 | Auxin-responsive family protein | 2.564 | 2.446 | 1.730 |
| 1 | 1270 | bx002147 | AT1G27730 | salt tolerance zinc finger | 3.111 | 2.017 | 1.427 |
| 1 | 1475 | bx002457 | AT2G05940 | RPM1-induced protein kinase, Protein kinase superfamily protein | 2.184 | 2.919 | 0.716 |
| 1 | 1481 | bx002469 | AT4G26270 | phosphofructokinase 3 | 5.307 | 1.321 | 0.286 |
| 1 | 1589 | bx002637 | AT5G60900 | receptor-like protein kinase 1 | 3.353 | 2.023 | 1.237 |
| 1 | 1741 | bx002869 | AT3G48990 | AMP-dependent synthetase and ligase family protein | 3.245 | 2.355 | 0.419 |
| 1 | 1751 | bx002887 | AT3G56400 | ARABIDOPSIS THALIANA WRKY DNA-BINDING PROTEIN 70, WRKY DNA-binding protein 70 | 2.150 | 2.945 | -0.088 |
| 1 | 1842 | bx003030 | AT2G34930 | disease resistance family protein / LRR family protein | 1.944 | 2.460 | -1.331 |
| 1 | 1865 | bx003059 | AT5G46760 | Basic helix-loop-helix (bHLH) DNA-binding family protein | 3.002 | 2.994 | 2.158 |
| 1 | 2119 | bx003446 | AT3G60770 | Ribosomal protein S13/S15 | 2.044 | 2.609 | 1.172 |
| 1 | 2154 | bx003502 | AT5G25930 | Protein kinase family protein with leucine-rich repeat domain | 2.562 | 2.640 | 0.579 |
| 1 | 2306 | bx003728 | AT5G02500 | HEAT SHOCK PROTEIN 70-1, ARABIDOPSIS THALIANA HEAT SHOCK COGNATE PROTEIN 70-1, heat shock cognate protein 70-1 | 2.438 | 3.586 | 1.493 |
| 1 | 2367 | bx003816 | AT1G24140 | Matrixin family protein | 3.049 | 2.936 | 1.268 |
| 1 | 2487 | bx003997 | AT4G37260 | myb domain protein 73 | 5.203 | 4.679 | 3.450 |
| 1 | 2594 | bx004158 | AT5G14040 | phosphate transporter 3;1 | 4.785 | 2.471 | 0.678 |
| 1 | 2809 | bx004476 | AT1G59900 | pyruvate dehydrogenase complex E1 alpha subunit | 2.765 | 3.614 | -0.216 |
| 1 | 2837 | bx004520 | AT2G22590 | UDP-Glycosyltransferase superfamily protein | 2.881 | 0.394 | 0.023 |
| 1 | 2966 | bx004719 | AT3G60450 | Phosphoglycerate mutase family protein | 3.029 | 2.590 | 0.875 |
| 1 | 3091 | bx004894 |  |  | 2.283 | 2.695 | 0.883 |
| 1 | 3115 | bx004930 | AT3G57450 | unknown protein | 2.676 | 1.889 | -0.787 |
| 1 | 3183 | bx005030 | AT1G43910 | P-loop containing nucleoside triphosphate hydrolases superfamily protein | 2.920 | 3.450 | 2.055 |
| 1 | 3317 | bx005223 | AT2G16430 | purple acid phosphatase 10 | 2.534 | 3.321 | 1.349 |
| 1 | 3347 | bx005262 |  |  | 2.440 | 2.302 | 1.385 |
| 1 | 3362 | bx005284 | AT4G36830 | GNS1/SUR4 membrane protein family | 2.368 | 1.173 | 0.234 |
| 1 | 3440 | bx005404 | AT1G02850 | beta glucosidase 11 | 2.248 | 3.233 | 0.880 |
| 1 | 3490 | bx005471 | AT2G42360 | RING/U-box superfamily protein | 3.255 | 2.580 | 0.321 |
| 1 | 3502 | bx005490 | AT1G15210 | ATP-binding cassette G35, pleiotropic drug resistance 7 | 2.938 | 1.829 | 0.498 |
| 1 | 3531 | bx005537 | AT3G62990 | unknown protein | 2.555 | 2.366 | 0.817 |
| 1 | 3547 | bx005565 | AT2G21210 | SAUR-like auxin-responsive protein family | 2.682 | 1.726 | -0.120 |
| 1 | 3579 | bx005608 | AT2G22500 | DICARBOXYLATE CARRIER 1, PLANT UNCOUPLING MITOCHONDRIAL PROTEIN 5, uncoupling protein 5 | 2.691 | 3.552 | 0.821 |
| 1 | 3638 | bx005690 |  |  | 2.457 | 2.547 | 1.092 |
| 1 | 3686 | bx005757 | AT5G40010 | ATPase-in-Seed-Development, AAA-ATPase 1 | 4.132 | 4.527 | 0.593 |
| 1 | 3689 | bx005761 | AT4G02380 | Arabidopsis thaliana late embryogenensis abundant like 5, senescence-associated gene 21 | 3.256 | 2.462 | 0.942 |
| 1 | 3792 | bx005917 | AT2G38060 | phosphate transporter 4;2 | 4.227 | 1.457 | 0.246 |
| 1 | 3900 | bx006074 | AT2G37000 | TCP family transcription factor | 2.312 | 2.225 | 1.224 |
| 1 | 4041 | bx006277 | AT3G52450 | plant U-box 22 | 2.606 | 0.371 | -0.014 |
| 1 | 4085 | bx006344 | AT5G22540 | Plant protein of unknown function (DUF247) | 2.734 | 1.319 | -0.030 |
| 1 | 4127 | bx006406 |  |  | 3.047 | 1.255 | 0.869 |
| 1 | 4333 | bx006708 | AT3G16510 | Calcium-dependent lipid-binding (CaLB domain) family protein | 2.620 | 1.503 | -0.064 |
| 1 | 4344 | bx006729 |  |  | 4.025 | 4.985 | 2.216 |
| 1 | 4528 | bx006994 | AT2G37040 | PHE ammonia lyase 1 | 2.503 | 0.579 | -0.828 |
| 1 | 4543 | bx007016 | AT3G21760 | HYPOSTATIN RESISTANCE 1, UDP-Glycosyltransferase superfamily protein | 4.816 | 3.632 | 2.776 |
| 1 | 4646 | bx007170 | AT1G11340 | S-locus lectin protein kinase family protein | 2.109 | 3.799 | 0.405 |
| 1 | 4701 | bx007242 | AT1G53440 | Leucine-rich repeat transmembrane protein kinase | 4.037 | 3.159 | 1.327 |
| 1 | 4833 | bx007431 | AT4G22810 | Predicted AT-hook DNA-binding family protein | 2.586 | 2.924 | 0.369 |
| 1 | 4917 | bx007552 | AT3G50930 | cytochrome BC1 synthesis | 3.133 | 3.576 | -0.430 |
| 1 | 5006 | bx007679 | AT5G67080 | mitogen-activated protein kinase kinase kinase 19 | 4.478 | 3.321 | 2.681 |
| 1 | 5103 | bx007824 | AT4G02780 | GA REQUIRING 1, CPP synthase, ARABIDOPSIS THALIANA ENT-COPALYL DIPHOSPHATE SYNTHETASE 1, Terpenoid cyclases/Protein prenyltransferases superfamily protein | 5.592 | 5.361 | 2.739 |
| 1 | 5134 | bx007866 | AT4G14746 | unknown protein | 2.564 | 1.899 | 1.022 |
| 1 | 5267 | bx008041 | AT2G37710 | receptor lectin kinase | 2.710 | 3.509 | 1.713 |
| 1 | 5275 | bx008053 |  |  | 5.823 | 3.628 | 1.045 |
| 1 | 5320 | bx008127 | AT1G07530 | ARABIDOPSIS THALIANA GRAS (GAI, RGA, SCR) 2, SCARECROW-like 14 | 2.734 | 2.755 | 1.123 |
| 1 | 5401 | bx008241 | AT1G20860 | phosphate transporter 1;8 | 4.461 | 3.052 | 0.496 |
| 1 | 5417 | bx008258 | AT4G34860 | alkaline/neutral invertase B, Plant neutral invertase family protein | 2.830 | 2.166 | 1.700 |
| 1 | 5426 | bx008268 | AT4G33300 | ADR1-like 1 | 2.649 | 1.848 | 1.052 |
| 1 | 5664 | bx008613 | AT1G47655 | Dof-type zinc finger DNA-binding family protein | 2.450 | 1.985 | -0.457 |
| 1 | 5722 | bx008711 |  |  | 4.161 | 3.583 | 2.596 |
| 1 | 5768 | bx008774 | AT1G61340 | F-box family protein | 4.337 | 3.024 | 0.568 |
| 1 | 5771 | bx008778 | AT5G65380 | MATE efflux family protein | 2.952 | 2.707 | 0.512 |
| 1 | 5919 | bx008968 | AT5G46330 | FLAGELLIN-SENSITIVE 2, Leucine-rich receptor-like protein kinase family protein | 3.353 | 2.951 | 1.294 |
| 1 | 5954 | bx009007 | AT2G37770 | Chloroplastic aldo-keto reductase, Aldo-keto reductase family 4 member C9, NAD(P)-linked oxidoreductase superfamily protein | 1.773 | 2.479 | 0.839 |
| 1 | 5975 | bx009033 | AT4G15560 | 1-DEOXY-D-XYLULOSE 5-PHOSPHATE SYNTHASE 2, CLOROPLASTOS ALTERADOS 1, Deoxyxylulose-5-phosphate synthase | 4.246 | 2.933 | 0.365 |
| 1 | 6060 | bx009149 | AT3G27890 | NADPH:quinone oxidoreductase | 2.559 | 3.166 | 0.413 |
| 1 | 6063 | bx009154 | AT2G34500 | cytochrome P450, family 710, subfamily A, polypeptide 1 | 2.878 | 3.152 | 1.414 |
| 1 | 6155 | bx009282 | AT2G24600 | Ankyrin repeat family protein | 3.840 | 3.430 | 0.126 |
| 1 | 6192 | bx009330 | AT1G24020 | MLP-like protein 423 | 5.361 | 4.204 | 2.995 |
| 1 | 6228 | bx009391 | AT5G47530 | Auxin-responsive family protein | 2.398 | 2.491 | 1.392 |
| 1 | 6325 | bx009516 | AT5G07360 | Amidase family protein | 2.823 | 2.653 | 0.595 |
| 1 | 6407 | bx009642 |  |  | 3.201 | 1.450 | 1.226 |
| 1 | 6422 | bx009670 | AT1G28680 | HXXXD-type acyl-transferase family protein | 2.686 | 1.719 | 0.310 |
| 1 | 6424 | bx009672 | AT3G47180 | RING/U-box superfamily protein | 5.929 | 6.120 | 3.195 |
| 1 | 6542 | bx009851 | AT3G47570 | Leucine-rich repeat protein kinase family protein | 2.414 | 1.623 | 0.695 |
| 1 | 6715 | bx010092 | AT4G25820 | xyloglucan endotransglycosylase 9, xyloglucan endotransglucosylase/hydrolase 14 | 3.447 | 2.085 | 1.232 |
| 1 | 6780 | bx010180 | AT1G27060 | Regulator of chromosome condensation (RCC1) family protein | 1.595 | 2.331 | 0.995 |
| 1 | 6866 | bx010293 | AT4G18380 | F-box family protein | 2.607 | 1.794 | 0.402 |
| 1 | 7161 | bx010723 | AT5G63450 | cytochrome P450, family 94, subfamily B, polypeptide 1, cytochrome P450, family 94, subfamily B, polypeptide 1 | 2.272 | 2.663 | 0.304 |
| 1 | 7251 | bx010840 | AT4G13180 | NAD(P)-binding Rossmann-fold superfamily protein | 2.417 | 2.389 | 1.355 |
| 1 | 7548 | bx011258 | AT2G16250 | Leucine-rich repeat protein kinase family protein | 2.742 | 3.363 | 1.861 |
| 1 | 7589 | bx011313 | AT5G40850 | urophorphyrin methylase 1 | 3.315 | 2.971 | 1.662 |
| 1 | 7727 | bx011521 | AT3G48280 | cytochrome P450, family 71, subfamily A, polypeptide 25, cytochrome P450, family 71, subfamily A, polypeptide 25 | 4.616 | 4.303 | 2.185 |
| 1 | 7976 | bx011875 | AT3G06350 | MATERNAL EFFECT EMBRYO ARREST 32, EMBRYO DEFECTIVE 3004, dehydroquinate dehydratase, putative / shikimate dehydrogenase, putative | 3.668 | 1.473 | 0.123 |
| 1 | 8095 | bx012032 | AT5G05690 | DWARF 3, CYTOCHROME P450 90A1, CONSTITUTIVE PHOTOMORPHOGENIC DWARF, CABBAGE 3, Cytochrome P450 superfamily protein | 3.099 | 2.758 | 2.148 |
| 1 | 8116 | bx012059 | AT3G07070 | Protein kinase superfamily protein | 2.639 | 2.291 | 1.251 |
| 1 | 8241 | bx012236 | AT4G03230 | S-locus lectin protein kinase family protein | 3.265 | 3.813 | 1.814 |
| 1 | 8407 | bx012470 | AT4G39830 | Cupredoxin superfamily protein | 4.018 | 3.112 | -0.213 |
| 1 | 8412 | bx012477 | AT4G35750 | SEC14 cytosolic factor family protein / phosphoglyceride transfer family protein | 2.581 | 1.966 | 1.159 |
| 1 | 8438 | bx012517 | AT2G18193 | P-loop containing nucleoside triphosphate hydrolases superfamily protein | 2.070 | 2.629 | 0.586 |
| 1 | 8520 | bx012641 | AT2G42010 | phospholipase D beta 1 | 2.802 | 2.816 | 1.747 |
| 1 | 8628 | bx012791 | AT2G37980 | O-fucosyltransferase family protein | 3.070 | 2.870 | 1.631 |
| 1 | 8659 | bx012834 |  |  | 2.742 | 1.975 | 0.619 |
| 1 | 8676 | bx012856 | AT1G25560 | TEMPRANILLO 1, ETHYLENE RESPONSE DNA BINDING FACTOR 1, AP2/B3 transcription factor family protein | 3.103 | 3.117 | 1.996 |
| 1 | 8718 | bx012918 | AT2G22590 | UDP-Glycosyltransferase superfamily protein | 3.910 | 2.656 | 1.341 |
| 1 | 8753 | bx012961 | AT4G28490 | RECEPTOR-LIKE PROTEIN KINASE 5, HAESA, Leucine-rich receptor-like protein kinase family protein | 4.979 | 5.112 | 2.786 |
| 1 | 8755 | bx012963 | AT5G61520 | Major facilitator superfamily protein | 2.515 | 1.961 | 0.721 |
| 1 | 8792 | bx013020 | AT1G47490 | RNA-binding protein 47C | 2.289 | 2.561 | 1.578 |
| 1 | 8994 | bx013315 |  |  | 2.314 | 2.066 | 1.451 |
| 1 | 9172 | bx013583 |  |  | 2.635 | 2.721 | 0.985 |
| 1 | 9254 | bx013696 | AT2G47800 | ATP-binding cassette C4, multidrug resistance-associated protein 4 | 2.708 | 2.831 | 1.750 |
| 1 | 9497 | bx014049 | AT2G24520 | H(+)-ATPase 5, H(+)-ATPase 5, H(+)-ATPase 5 | 3.319 | 2.370 | 2.101 |
| 1 | 9547 | bx014115 | AT1G24020 | MLP-like protein 423 | 3.427 | 4.152 | 1.173 |
| 1 | 9553 | bx014124 | AT5G11630 | unknown protein | 3.167 | 1.548 | 1.284 |
| 1 | 9573 | bx014154 | AT5G11630 | unknown protein | 3.200 | 1.612 | 1.574 |
| 1 | 9609 | bx014200 |  |  | 1.822 | 2.490 | 0.763 |
| 1 | 9632 | bx014237 | AT5G67080 | mitogen-activated protein kinase kinase kinase 19 | 3.916 | 3.165 | 2.599 |
| 1 | 9670 | bx014295 | AT5G14040 | phosphate transporter 3;1 | 4.258 | 1.969 | 0.715 |
| 1 | 9705 | bx014345 | AT5G41340 | ubiquitin conjugating enzyme 4 | 1.969 | 3.135 | 1.182 |
| 1 | 9844 | bx014541 | AT5G40010 | ATPase-in-Seed-Development, AAA-ATPase 1 | 1.678 | 2.933 | 0.464 |
| 1 | 9872 | bx014584 |  |  | 4.247 | 4.891 | 0.304 |
| 1 | 10365 | bx015339 | AT2G42280 | basic helix-loop-helix (bHLH) DNA-binding superfamily protein | 2.815 | 2.751 | 1.658 |
| 1 | 10593 | bx015719 | AT3G19480 | D-3-phosphoglycerate dehydrogenase | 2.610 | 2.305 | 1.149 |
| 1 | 10597 | bx015731 | AT3G09830 | Protein kinase superfamily protein | 2.305 | 2.360 | 1.360 |
| 1 | 10609 | bx015747 | AT5G26710 | Glutamyl/glutaminyl-tRNA synthetase, class Ic | 1.832 | 2.318 | 1.244 |
| 1 | 10714 | bx015910 | AT2G18390 | TITAN 5, HALLIMASCH, ARF-LIKE 2, ADP-ribosylation factor family protein | 3.913 | 1.540 | 1.733 |
| 1 | 11169 | bx016645 | AT3G06700 | Ribosomal L29e protein family | 3.810 | 2.412 | 2.465 |
| 1 | 12001 | bx017988 | AT1G67830 | Arabidopsis thaliana alpha-fucosidase 1, alpha-fucosidase 1 | 5.194 | 4.203 | 3.475 |
| 1 | 12065 | bx018105 | AT3G12360 | INCREASED TOLERANCE TO NACL, Ankyrin repeat family protein | 2.332 | 2.006 | -0.228 |
| 1 | 12264 | bx018409 | AT5G41330 | BTB/POZ domain with WD40/YVTN repeat-like protein | 2.992 | 3.184 | 1.826 |
| 1 | 12495 | bx018747 | AT3G57550 | GUANYLATE KINAS 2, guanylate kinase | 4.145 | 4.268 | 1.939 |
| 1 | 12546 | bx018816 | AT5G16930 | AAA-type ATPase family protein | 1.890 | 2.307 | 1.155 |
| 1 | 12635 | bx018932 | AT3G10985 | ARABIDOPSIS THALIANA WOUND-INDUCED PROTEIN 12, senescence associated gene 20 | 2.306 | 2.444 | 1.326 |
| 1 | 12732 | bx019052 | AT3G15630 | unknown protein | 2.609 | 1.559 | 0.588 |
| 1 | 12748 | bx019079 | AT1G01490 | Heavy metal transport/detoxification superfamily protein | 3.101 | 3.379 | 1.365 |
| 1 | 12816 | bx019172 | AT2G26930 | PIGMENT DEFECTIVE 277, 4-(cytidine 5′-diphospho)-2-C-methyl-d-erythritol kinase, 4-(cytidine 5'-phospho)-2-C-methyl-D-erithritol kinase | 3.208 | 3.172 | 1.866 |
| 1 | 12850 | bx019215 | AT4G13830 | DNAJ-like 20 | 3.838 | 2.762 | 2.304 |
| 1 | 12903 | bx019294 | AT5G01210 | HXXXD-type acyl-transferase family protein | 2.746 | 2.790 | 1.493 |
| 1 | 13142 | bx019615 | AT5G28050 | Cytidine/deoxycytidylate deaminase family protein | 3.208 | 2.560 | 1.225 |
| 1 | 13168 | bx019652 | AT3G12580 | ARABIDOPSIS HEAT SHOCK PROTEIN 70, heat shock protein 70 | 3.167 | 3.616 | 2.075 |
| 1 | 13192 | bx019685 | AT5G02050 | Mitochondrial glycoprotein family protein | 2.250 | 2.666 | 0.330 |
| 1 | 13536 | bx020141 | AT1G22150 | sulfate transporter 1;3 | 1.605 | 2.932 | 0.474 |
| 1 | 13821 | bx020502 | AT5G53110 | RING/U-box superfamily protein | 2.806 | 2.492 | -0.306 |
| 1 | 14095 | bx020875 | AT2G23970 | Class I glutamine amidotransferase-like superfamily protein | 0.754 | 2.350 | -0.096 |
| 1 | 14147 | bx020949 | AT4G20840 | FAD-binding Berberine family protein | 2.061 | 2.428 | 0.379 |
| 1 | 14681 | bx021653 | AT2G18710 | SECY homolog 1 | 4.586 | 2.467 | 1.960 |
| 1 | 14773 | bx021769 | AT4G32690 | ARABIDOPSIS HEMOGLOBIN 3, hemoglobin 3 | 3.321 | 3.579 | 1.922 |
| 1 | 14809 | bx021822 | AT1G10522 | unknown protein | 2.177 | 2.504 | 1.134 |
| 1 | 15017 | bx022099 | AT1G03210 | Phenazine biosynthesis PhzC/PhzF protein | 2.566 | 3.079 | 1.770 |
| 1 | 15071 | bx022164 | AT1G30135 | jasmonate-zim-domain protein 8 | 2.444 | 2.410 | 1.723 |
| 1 | 15138 | bx022254 | AT1G26780 | LATERAL ORGAN FUSION 1, myb domain protein 117 | 3.228 | 3.273 | 2.330 |
| 1 | 15252 | bx022398 | AT3G44260 | CCR4- associated factor 1a, Polynucleotidyl transferase, ribonuclease H-like superfamily protein | 3.137 | 2.394 | 0.455 |
| 1 | 15444 | bx022650 | AT5G04590 | sulfite reductase | 3.414 | 2.871 | 1.206 |
| 1 | 15497 | bx022720 | AT5G64210 | alternative oxidase 2 | 2.471 | 1.937 | 0.862 |
| 1 | 15646 | bx022921 | AT2G28570 | unknown protein | 2.326 | 1.880 | -0.368 |
| 1 | 15700 | bx022985 | AT4G17900 | PLATZ transcription factor family protein | 3.477 | 2.124 | 1.802 |
| 1 | 15827 | bx023170 |  |  | 5.461 | 4.681 | 2.980 |
| 1 | 15835 | bx023179 | AT2G38290 | AMMONIUM TRANSPORTER 2;1, ammonium transporter 2 | 3.902 | 3.181 | 2.249 |
| 1 | 15894 | bx023251 | AT3G26210 | cytochrome P450, family 71, subfamily B, polypeptide 23, cytochrome P450, family 71, subfamily B, polypeptide 23 | 6.375 | 6.650 | 4.422 |
| 1 | 15926 | bx023296 | AT3G56400 | ARABIDOPSIS THALIANA WRKY DNA-BINDING PROTEIN 70, WRKY DNA-binding protein 70 | 2.019 | 2.742 | -0.364 |
| 1 | 15960 | bx023339 | AT5G06570 | alpha/beta-Hydrolases superfamily protein | 3.959 | 3.265 | 2.373 |
| 1 | 15972 | bx023355 | AT5G28500 | unknown protein | 3.015 | 2.702 | 1.845 |
| 1 | 16048 | bx023458 | AT1G80840 | WRKY DNA-binding protein 40 | 2.955 | 3.672 | -0.465 |
| 1 | 16053 | bx023467 |  |  | 4.515 | 2.340 | -0.238 |
| 1 | 16287 | bx023793 | AT5G42510 | Disease resistance-responsive (dirigent-like protein) family protein | 3.662 | 3.738 | 1.272 |
| 1 | 16358 | bx023900 | AT1G28480 | Thioredoxin superfamily protein | 2.378 | 2.815 | 0.929 |
| 1 | 16482 | bx024058 | AT5G53870 | early nodulin-like protein 1 | 4.231 | 3.843 | 0.053 |
| 1 | 16579 | bx024192 | AT5G49480 | Ca2+-binding protein 1, Ca2+-binding protein 1, Ca2+-binding protein 1 | 2.205 | 2.684 | 1.150 |
| 1 | 16580 | bx024193 | AT1G65840 | polyamine oxidase 4 | 2.600 | 2.643 | 1.801 |
| 1 | 16672 | bx024346 | AT4G05020 | NAD(P)H dehydrogenase B2 | 4.513 | 2.780 | 1.818 |
| 1 | 16757 | bx024465 | AT3G06350 | MATERNAL EFFECT EMBRYO ARREST 32, EMBRYO DEFECTIVE 3004, dehydroquinate dehydratase, putative / shikimate dehydrogenase, putative | 3.951 | 1.483 | 0.306 |
| 1 | 16879 | bx024616 | AT1G07180 | alternative NAD(P)H dehydrogenase 1, ARABIDOPSIS THALIANA INTERNAL NON-PHOSPHORYLATING NAD ( P ) H DEHYDROGENASE, alternative NAD(P)H dehydrogenase 1 | 3.417 | 1.980 | -0.081 |
| 1 | 17036 | bx024817 | AT5G61170 | Ribosomal protein S19e family protein | 2.250 | 2.411 | 0.622 |
| 1 | 17041 | bx024822 | AT3G16150 | asparaginase B1, N-terminal nucleophile aminohydrolases (Ntn hydrolases) superfamily protein | 4.220 | 2.893 | 1.928 |
| 1 | 17055 | bx024837 | AT3G19480 | D-3-phosphoglycerate dehydrogenase | 3.109 | 1.806 | 0.084 |
| 1 | 17081 | bx024871 | AT2G17040 | NAC domain containing protein 36 | 2.974 | 3.352 | 1.879 |
| 1 | 17283 | bx025133 | AT4G32400 | SODIUM HYPERSENSITIVE 1, EMBRYO DEFECTIVE 42, EMBRYO DEFECTIVE 104, ARABIDOPSIS THALIANA BRITTLE 1, Mitochondrial substrate carrier family protein | 2.934 | 2.702 | 1.757 |
| 1 | 17285 | bx025135 | AT5G33320 | PHOSPHOENOLPYRUVATE/PHOSPHATE TRANSLOCATOR, CAB UNDEREXPRESSED 1, ARABIDOPSIS THALIANA PHOSPHATE/PHOSPHOENOLPYRUVATE TRANSLOCATOR, Glucose-6-phosphate/phosphate translocator-related | 4.500 | 3.397 | 1.494 |
| 1 | 17310 | bx025169 | AT4G35640 | serine acetyltransferase 3;2 | 2.268 | 2.469 | 0.376 |
| 1 | 17422 | bx025337 | AT5G53800 | unknown protein | 2.528 | 1.303 | -0.353 |
| 1 | 17429 | bx025349 | AT1G59900 | pyruvate dehydrogenase complex E1 alpha subunit | 3.133 | 3.070 | 0.379 |
| 1 | 17496 | bx025440 | AT1G51760 | JASMONIC ACID RESPONSIVE 3, IAA-ALANINE RESISTANT 3, peptidase M20/M25/M40 family protein | 5.812 | 4.679 | 4.185 |
| 1 | 17566 | bx025536 | AT2G47710 | Adenine nucleotide alpha hydrolases-like superfamily protein | 0.910 | 3.468 | -0.013 |
| 1 | 17705 | bx025727 |  |  | 2.337 | 2.314 | 1.624 |
| 1 | 17813 | bx025866 | AT5G61520 | Major facilitator superfamily protein | 1.849 | 2.644 | 1.169 |
| 1 | 18000 | bx026110 | AT2G38470 | WRKY DNA-binding protein 33 | 3.799 | 3.348 | 0.498 |
| 1 | 18039 | bx026161 | AT1G14820 | Sec14p-like phosphatidylinositol transfer family protein | 2.001 | 2.311 | 0.463 |
| 1 | 18047 | bx026170 | AT1G27060 | Regulator of chromosome condensation (RCC1) family protein | 1.666 | 2.388 | 1.100 |
| 1 | 18048 | bx026171 | AT1G13480 | Protein of unknown function (DUF1262) | 3.087 | 2.762 | 1.447 |
| 1 | 18250 | bx026443 |  |  | 3.348 | 3.418 | 2.187 |
| 1 | 18302 | bx026510 | AT2G16790 | P-loop containing nucleoside triphosphate hydrolases superfamily protein | 2.493 | 2.990 | 1.423 |
| 1 | 18375 | bx026599 | AT2G38470 | WRKY DNA-binding protein 33 | 2.813 | 2.343 | 1.112 |
| 1 | 18396 | bx026625 | AT3G07600 | Heavy metal transport/detoxification superfamily protein | 3.830 | 4.597 | 0.596 |
| 1 | 18423 | bx026667 | AT4G11070 | WRKY family transcription factor | 2.799 | 3.668 | 0.270 |
| 1 | 18573 | bx026865 | AT3G21250 | ARABIDOPSIS THALIANA MULTIDRUG RESISTANCE-ASSOCIATED PROTEIN 6, ATP-binding cassette C8, multidrug resistance-associated protein 6 | 2.849 | 2.009 | 2.025 |
| 1 | 18666 | bx026993 | AT2G22590 | UDP-Glycosyltransferase superfamily protein | 4.993 | 2.491 | 1.921 |
| 1 | 18712 | bx027059 | AT3G23240 | ethylene response factor 1 | 5.236 | 4.949 | 3.182 |
| 1 | 18713 | bx027061 |  |  | 2.699 | 2.481 | 0.757 |
| 1 | 19036 | bx027500 | AT5G22300 | nitrilase 4 | 2.705 | 2.774 | 1.794 |
| 1 | 19086 | bx027564 | AT5G26340 | SUGAR TRANSPORT PROTEIN 13, Major facilitator superfamily protein | 5.350 | 5.313 | 2.577 |
| 1 | 19171 | bx027682 | AT5G10830 | S-adenosyl-L-methionine-dependent methyltransferases superfamily protein | 3.653 | 4.000 | 2.087 |
| 1 | 19330 | bx027898 | AT2G02010 | glutamate decarboxylase 4 | 2.736 | 1.596 | 1.547 |
| 1 | 19444 | bx028050 | AT1G21270 | wall-associated kinase 2 | 3.961 | 4.000 | 2.272 |
| 1 | 19496 | bx028111 | AT3G05200 | RING/U-box superfamily protein | 2.590 | 2.570 | 1.734 |
| 1 | 19618 | bx028265 | AT3G53690 | RING/U-box superfamily protein | 2.096 | 2.921 | 1.064 |
| 1 | 19691 | bx028359 |  |  | 3.558 | 2.077 | 0.599 |
| 1 | 19832 | bx028546 | AT3G14690 | cytochrome P450, family 72, subfamily A, polypeptide 15, cytochrome P450, family 72, subfamily A, polypeptide 15 | 2.812 | 2.087 | 0.076 |
| 1 | 20048 | bx028865 | AT2G22590 | UDP-Glycosyltransferase superfamily protein | 3.966 | 2.647 | 1.634 |
| 1 | 20071 | bx028899 | AT4G33300 | ADR1-like 1 | 2.606 | 1.771 | 0.950 |
| 1 | 20116 | bx028966 | AT3G02468 | conserved peptide upstream open reading frame 9 | 2.436 | 0.796 | 0.474 |
| 1 | 20158 | bx029021 | AT1G79750 | Arabidopsis thaliana NADP-malic enzyme 4, NADP-malic enzyme 4 | 2.546 | 1.421 | 0.671 |
| 1 | 20185 | bx029058 |  |  | 4.205 | 3.568 | 0.072 |
| 1 | 20209 | bx029091 | AT3G12500 | PATHOGENESIS-RELATED 3, basic chitinase | 5.446 | 4.507 | 1.556 |
| 1 | 20351 | bx029268 | AT3G51680 | short-chain dehydrogenase/reductase 2, NAD(P)-binding Rossmann-fold superfamily protein | 5.736 | 3.641 | 1.488 |
| 1 | 20483 | bx029440 | AT3G13790 | ARABIDOPSIS THALIANA CELL WALL INVERTASE 1, Glycosyl hydrolases family 32 protein | 5.074 | 3.633 | 0.360 |
| 1 | 20623 | bx029622 | AT2G30250 | WRKY DNA-binding protein 25 | 2.846 | 2.311 | 1.530 |
| 1 | 20688 | bx029719 | AT1G27730 | salt tolerance zinc finger | 3.190 | 2.464 | 2.015 |
| 1 | 20822 | bx029892 | AT3G02550 | LOB domain-containing protein 41 | 3.563 | 2.328 | 0.207 |
| 1 | 20836 | bx029908 | AT1G34300 | lectin protein kinase family protein | 2.481 | 2.827 | 1.442 |
| 1 | 20961 | bx030067 | AT1G12910 | LIGHT-REGULATED WD 1, ANTHOCYANIN11, Transducin/WD40 repeat-like superfamily protein | 2.555 | 1.118 | -0.118 |
| 1 | 21055 | bx030207 | AT1G23720 | Proline-rich extensin-like family protein | 1.801 | 2.626 | 1.190 |
| 1 | 21169 | bx030368 | AT5G49690 | UDP-Glycosyltransferase superfamily protein | 4.122 | 2.755 | 2.227 |
| 1 | 21238 | bx030466 | AT5G39670 | Calcium-binding EF-hand family protein | 3.159 | 2.579 | 0.582 |
| 1 | 21278 | bx030530 | AT1G47530 | MATE efflux family protein | 2.868 | 2.490 | 1.648 |
| 1 | 21289 | bx030542 | AT1G17020 | senescence-related gene 1 | 3.457 | 3.949 | 1.043 |
| 1 | 21401 | bx030682 | AT3G14690 | cytochrome P450, family 72, subfamily A, polypeptide 15, cytochrome P450, family 72, subfamily A, polypeptide 15 | 3.077 | 3.925 | -0.338 |
| 1 | 21459 | bx030751 | AT2G22590 | UDP-Glycosyltransferase superfamily protein | 2.218 | 2.364 | 1.014 |
| 1 | 21598 | bx030935 | AT1G27730 | salt tolerance zinc finger | 3.749 | 3.989 | 1.763 |
| 1 | 21761 | bx031175 | AT4G19840 | phloem protein 2-A1 | 2.600 | 1.973 | 1.651 |
| 1 | 21762 | bx031181 | AT1G49780 | plant U-box 26 | 2.694 | 2.718 | 1.685 |
| 1 | 21769 | bx031189 | AT2G29420 | GLUTATHIONE S-TRANSFERASE 25, glutathione S-transferase tau 7 | 5.779 | 6.349 | 3.877 |
| 1 | 21906 | bx031374 | AT5G50080 | ethylene response factor 110, ethylene response factor 110 | 4.365 | 3.868 | 2.474 |
| 1 | 22359 | bx032026 | AT5G05690 | DWARF 3, CYTOCHROME P450 90A1, CONSTITUTIVE PHOTOMORPHOGENIC DWARF, CABBAGE 3, Cytochrome P450 superfamily protein | 2.477 | 2.371 | 1.555 |
| 1 | 22390 | bx032062 | AT5G64810 | ARABIDOPSIS THALIANA WRKY DNA-BINDING PROTEIN 51, WRKY DNA-binding protein 51 | 4.475 | 4.550 | 1.345 |
| 1 | 22398 | bx032074 | AT2G15220 | Plant basic secretory protein (BSP) family protein | 2.401 | 2.346 | -0.281 |
| 1 | 22427 | bx032110 | AT1G21270 | wall-associated kinase 2 | 3.606 | 3.329 | 1.923 |
| 1 | 23051 | bx033017 | AT5G13220 | TIFY DOMAIN PROTEIN 9, JASMONATE-ASSOCIATED 1, jasmonate-zim-domain protein 10 | 2.815 | 1.457 | 0.005 |
| 1 | 23086 | bx033064 | AT4G20970 | basic helix-loop-helix (bHLH) DNA-binding superfamily protein | 2.747 | 2.921 | 1.531 |
| 1 | 23234 | bx033267 | AT5G19855 | homologue of cyanobacterial RbcX 2, Chaperonin-like RbcX protein | 3.491 | 0.309 | -0.226 |
| 1 | 23936 | bx034286 | AT3G22890 | ATP sulfurylase 1 | 2.461 | 2.320 | 0.424 |
| 1 | 25361 | bx036334 | AT1G65840 | polyamine oxidase 4 | 2.617 | 2.532 | 1.675 |
| 1 | 25997 | bx037328 |  |  | 1.971 | 2.473 | 1.267 |
| 1 | 26203 | bx037674 | AT5G20230 | SENESCENCE ASSOCIATED GENE 14, BLUE COPPER BINDING PROTEIN, blue-copper-binding protein | 3.342 | 3.604 | 1.970 |
| 1 | 26285 | bx037785 | AT1G74360 | Leucine-rich repeat protein kinase family protein | 2.779 | 1.536 | 0.248 |
| 1 | 26289 | bx037791 | AT1G10340 | Ankyrin repeat family protein | 2.669 | 2.513 | -0.666 |
| 1 | 26299 | bx037808 | AT4G12735 | unknown protein | 4.924 | 4.279 | 2.302 |
| 1 | 26303 | bx037814 | AT3G23250 | myb domain protein 15 | 2.356 | 0.207 | -0.925 |
| 1 | 26337 | bx037859 | AT4G11650 | osmotin 34 | 3.940 | 4.609 | 2.132 |
| 1 | 26377 | bx037916 | AT1G28190 | unknown protein | 3.979 | 5.293 | 2.247 |
| 1 | 26397 | bx037937 | AT2G29420 | GLUTATHIONE S-TRANSFERASE 25, glutathione S-transferase tau 7 | 1.871 | 3.274 | 0.256 |
| 1 | 26404 | bx037944 | AT1G55190 | PRENYLATED RAB ACCEPTOR 1.F2, PRA1 (Prenylated rab acceptor) family protein | 4.383 | 5.280 | 2.997 |
| 1 | 26457 | bx038015 | AT3G63380 | ATPase E1-E2 type family protein / haloacid dehalogenase-like hydrolase family protein | 4.817 | 4.397 | 2.579 |
| 1 | 26472 | bx038040 | AT4G28940 | Phosphorylase superfamily protein | 3.811 | 3.981 | 0.582 |
| 1 | 26484 | bx038054 |  |  | 2.276 | 2.643 | 1.267 |
| 1 | 26493 | bx038065 | AT1G47670 | Transmembrane amino acid transporter family protein | 2.294 | 2.802 | 1.199 |
| 1 | 26517 | bx038098 | AT1G33590 | Leucine-rich repeat (LRR) family protein | 3.714 | 3.323 | 2.304 |
| 1 | 26580 | bx038186 | AT1G68180 | RING/U-box superfamily protein | 2.816 | 1.537 | 1.900 |
| 1 | 26613 | bx038227 | AT1G80840 | WRKY DNA-binding protein 40 | 5.962 | 4.815 | 3.367 |
| 1 | 26685 | bx038326 |  |  | 2.541 | 2.889 | -0.645 |
| 1 | 26737 | bx038394 | AT1G10480 | zinc finger protein 5 | 4.025 | 3.079 | 2.166 |
| 1 | 26780 | bx038451 | AT4G32300 | S-domain-2 5 | 1.866 | 3.015 | -0.235 |
| 1 | 26820 | bx038504 |  |  | 3.917 | 4.432 | 2.125 |
| 1 | 26848 | bx038539 | AT1G29290 | unknown protein | 3.574 | 3.430 | 1.765 |
| 1 | 26876 | bx038575 | AT1G72540 | Protein kinase superfamily protein | 2.482 | 2.265 | -0.042 |
| 1 | 26907 | bx038618 | AT1G80160 | glyoxylase I 7, Lactoylglutathione lyase / glyoxalase I family protein | 4.602 | 3.244 | 0.371 |
| 1 | 26996 | bx038733 | AT3G09270 | glutathione S-transferase TAU 8 | 3.119 | 3.698 | 0.981 |
| 1 | 27042 | bx038792 |  |  | 4.044 | 3.769 | 1.640 |
| 1 | 27072 | bx038835 | AT4G13510 | ARABIDOPSIS THALIANA AMMONIUM TRANSPORT 1, ammonium transporter 1;1 | 3.343 | 3.355 | 0.732 |
| 1 | 27100 | bx038869 | AT2G28690 | Protein of unknown function (DUF1635) | 3.490 | 3.763 | 2.315 |
| 1 | 27178 | bx038970 | AT1G14550 | Peroxidase superfamily protein | 3.197 | 2.036 | 2.230 |
| 1 | 27431 | bx039303 | AT1G76070 | unknown protein | 3.044 | 1.411 | 0.634 |
| 1 | 27536 | bx039438 | AT5G51920 | Pyridoxal phosphate (PLP)-dependent transferases superfamily protein | 2.418 | 0.714 | -2.156 |
| 1 | 27588 | bx039503 | AT5G40850 | urophorphyrin methylase 1 | 2.976 | 2.454 | 0.593 |
| 1 | 27719 | bx039680 | AT1G24020 | MLP-like protein 423 | 4.220 | 3.667 | -1.526 |
| 1 | 27743 | bx039711 | AT3G58060 | Cation efflux family protein | 2.877 | 3.126 | 1.057 |
| 1 | 27758 | bx039728 | AT1G15460 | ARABIDOPSIS THALIANA REQUIRES HIGH BORON 4, HCO3- transporter family | 4.897 | 2.323 | 0.038 |
| 1 | 27928 | bx039953 | AT5G22860 | Serine carboxypeptidase S28 family protein | 3.715 | 2.635 | 0.320 |
| 1 | 28172 | bx040265 | AT1G61560 | MILDEW RESISTANCE LOCUS O 6, Seven transmembrane MLO family protein | 3.228 | 2.241 | -1.150 |
| 1 | 28224 | bx040335 | AT3G59080 | Eukaryotic aspartyl protease family protein | 2.338 | 2.272 | 0.792 |
| 1 | 28315 | bx040445 |  |  | 2.583 | 2.579 | 1.543 |
| 1 | 28406 | bx040562 | AT4G32690 | ARABIDOPSIS HEMOGLOBIN 3, hemoglobin 3 | 2.994 | 3.328 | 1.962 |
| 1 | 28423 | bx040584 | AT4G11280 | 1-aminocyclopropane-1-carboxylic acid (acc) synthase 6 | 3.268 | 3.665 | -0.405 |
| 1 | 28461 | bx040630 | AT5G14860 | UDP-Glycosyltransferase superfamily protein | 4.125 | 2.295 | 2.190 |
| 1 | 28468 | bx040637 | AT3G21250 | ARABIDOPSIS THALIANA MULTIDRUG RESISTANCE-ASSOCIATED PROTEIN 6, ATP-binding cassette C8, multidrug resistance-associated protein 6 | 2.836 | 2.171 | 1.964 |
| 1 | 28660 | bx040881 | AT4G37290 | unknown protein | 5.535 | 3.555 | 0.006 |
| 1 | 28697 | bx040928 | AT3G16520 | UDP-glucosyl transferase 88A1 | 5.112 | 3.164 | 0.067 |
| 1 | 28712 | bx040950 | AT4G32300 | S-domain-2 5 | 4.034 | 4.479 | 1.023 |
| 1 | 28731 | bx040976 | AT4G23030 | MATE efflux family protein | 5.954 | 5.190 | 3.393 |
| 1 | 28775 | bx041033 | AT3G06350 | MATERNAL EFFECT EMBRYO ARREST 32, EMBRYO DEFECTIVE 3004, dehydroquinate dehydratase, putative / shikimate dehydrogenase, putative | 3.972 | 1.379 | 0.263 |
| 1 | 28849 | bx041129 | AT2G22950 | auto-regulated Ca2+-ATPase 7, Cation transporter/ E1-E2 ATPase family protein | 3.602 | 2.891 | 0.348 |
| 1 | 28868 | bx041154 | AT5G64120 | Peroxidase superfamily protein | 5.968 | 6.540 | 3.749 |
| 1 | 29007 | bx041328 | AT3G46100 | Histidyl-tRNA synthetase 1 | 4.288 | 3.386 | 2.239 |
| 1 | 29063 | bx041395 | AT4G13440 | Calcium-binding EF-hand family protein | 3.345 | 2.716 | 0.475 |
| 1 | 29084 | bx041419 | AT3G26210 | cytochrome P450, family 71, subfamily B, polypeptide 23, cytochrome P450, family 71, subfamily B, polypeptide 23 | 3.242 | 3.080 | 0.742 |
| 1 | 29168 | bx041531 |  |  | 2.618 | 2.023 | 0.710 |
| 1 | 29192 | bx041557 | AT2G28930 | protein kinase 1B | 2.634 | 2.459 | 1.118 |
| 1 | 29195 | bx041561 | AT4G27290 | S-locus lectin protein kinase family protein | 3.786 | 3.922 | 2.387 |
| 1 | 29272 | bx041655 |  |  | 3.581 | 3.928 | 2.090 |
| 1 | 29354 | bx041767 |  |  | 2.850 | 3.052 | 0.108 |
| 1 | 29426 | bx041859 | AT1G11050 | Protein kinase superfamily protein | 4.612 | 3.745 | -0.383 |
| 1 | 29444 | bx041883 | AT1G19670 | CORONATINE-INDUCED PROTEIN 1, chlorophyllase 1 | 7.328 | 5.621 | 2.240 |
| 1 | 29647 | bx042142 | AT1G62300 | WRKY family transcription factor | 2.408 | 1.881 | 1.584 |
| 1 | 29687 | bx042198 | AT5G39670 | Calcium-binding EF-hand family protein | 2.634 | 1.475 | -0.177 |
| 1 | 29911 | bx042469 | AT1G17020 | senescence-related gene 1 | 3.252 | 3.748 | 0.768 |
| 1 | 29922 | bx042483 | AT1G59590 | ZCF37 | 3.228 | 3.158 | 1.299 |
| 1 | 29924 | bx042485 | AT1G47670 | Transmembrane amino acid transporter family protein | 5.155 | 4.245 | 0.280 |
| 1 | 30093 | bx042684 | AT3G25882 | NIM1-interacting 2 | 4.745 | 5.138 | 1.397 |
| 1 | 30246 | bx042889 | AT3G60080 | RING/U-box superfamily protein | 6.001 | 5.156 | 3.755 |
| 1 | 30381 | bx043069 | AT3G01140 | NOECK, myb domain protein 106 | 2.429 | 0.384 | -0.230 |
| 1 | 30391 | bx043081 | AT1G32450 | nitrate transporter 1.5 | 4.398 | 4.393 | 2.904 |
| 1 | 30540 | bx043267 | AT4G27310 | B-box type zinc finger family protein | 3.484 | 1.378 | 0.538 |
| 1 | 30917 | bx043765 | AT1G35210 | unknown protein | 4.411 | 4.086 | 2.003 |
| 1 | 30993 | bx043861 | AT2G45760 | BON ASSOCIATION PROTEIN 1-LIKE, BON association protein 2 | 3.507 | 3.308 | 0.872 |
| 1 | 31003 | bx043871 | AT1G66350 | RGA-like 1 | 1.926 | 2.646 | 1.268 |
| 1 | 31226 | bx044159 |  |  | 3.827 | 3.027 | -0.005 |
| 1 | 31238 | bx044171 | AT3G22560 | Acyl-CoA N-acyltransferases (NAT) superfamily protein | 3.197 | 1.277 | 1.100 |
| 1 | 31279 | bx044227 | AT3G16150 | asparaginase B1, N-terminal nucleophile aminohydrolases (Ntn hydrolases) superfamily protein | 4.298 | 2.956 | 2.045 |
| 1 | 31413 | bx044400 | AT1G47490 | RNA-binding protein 47C | 2.090 | 2.306 | 1.482 |
| 1 | 31665 | bx044710 | AT4G03140 | NAD(P)-binding Rossmann-fold superfamily protein | 4.855 | 3.272 | 0.018 |
| 1 | 31681 | bx044728 | AT5G40460 | unknown protein | 2.411 | 1.681 | -0.758 |
| 1 | 31753 | bx044819 | AT5G40010 | ATPase-in-Seed-Development, AAA-ATPase 1 | 3.860 | 4.501 | 2.207 |
| 1 | 31757 | bx044823 | AT2G15780 | Cupredoxin superfamily protein | 3.386 | 3.301 | 2.362 |
| 1 | 32170 | bx045343 | AT3G12120 | fatty acid desaturase 2 | 2.515 | 2.175 | 0.565 |
| 1 | 32394 | bx045625 | AT2G47780 | Rubber elongation factor protein (REF) | 2.131 | 2.350 | 1.075 |
| 1 | 32567 | bx045841 |  |  | 4.496 | 4.146 | 2.298 |
| 1 | 32578 | bx045859 | AT4G00870 | basic helix-loop-helix (bHLH) DNA-binding superfamily protein | 5.743 | 4.135 | 3.222 |
| 1 | 32613 | bx045896 | AT4G21920 | unknown protein | 3.280 | 1.654 | -0.241 |
| 1 | 32938 | bx046303 | AT5G04870 | calcium dependent protein kinase 1 | 4.339 | 3.182 | 0.804 |
| 1 | 32996 | bx046375 | AT3G02875 | IAA-LEUCINE RESISTANT 1, Peptidase M20/M25/M40 family protein | 2.497 | 1.156 | 0.771 |
| 1 | 33188 | bx046621 | AT2G47700 | RED AND FAR-RED INSENSITIVE 2, RING/U-box superfamily protein | 3.405 | 4.479 | 0.731 |
| 1 | 33220 | bx046659 | AT5G51190 | Integrase-type DNA-binding superfamily protein | 2.695 | 2.701 | 1.398 |
| 1 | 33279 | bx046734 | AT3G07070 | Protein kinase superfamily protein | 2.718 | 2.293 | 1.167 |
| 1 | 33353 | bx046835 | AT2G26930 | PIGMENT DEFECTIVE 277, 4-(cytidine 5′-diphospho)-2-C-methyl-d-erythritol kinase, 4-(cytidine 5'-phospho)-2-C-methyl-D-erithritol kinase | 2.420 | 2.338 | 1.451 |
| 1 | 33462 | bx046959 | AT5G66070 | RING/U-box superfamily protein | 3.903 | 3.415 | 2.006 |
| 1 | 33465 | bx046963 | AT2G31180 | ARABIDOPSIS THALIANA MYB DOMAIN PROTEIN 14, myb domain protein 14 | 3.146 | 3.704 | 1.867 |
| 1 | 33546 | bx047067 | AT5G40850 | urophorphyrin methylase 1 | 3.231 | 3.123 | 1.835 |
| 1 | 33568 | bx047093 | AT2G37700 | Fatty acid hydroxylase superfamily | 3.122 | 2.107 | 1.164 |
| 1 | 33614 | bx047153 | AT3G54040 | PAR1 protein | 4.450 | 3.632 | 1.301 |
| 1 | 33688 | bx047240 | AT4G32300 | S-domain-2 5 | 3.977 | 4.590 | 1.017 |
| 1 | 33713 | bx047269 | AT5G20480 | EF-TU receptor | 2.550 | 1.140 | 0.970 |
| 1 | 33733 | bx047292 | AT2G44480 | beta glucosidase 17 | 7.648 | 4.716 | 0.227 |
| 1 | 33997 | bx047633 |  |  | 2.104 | 2.492 | 1.036 |
| 1 | 34037 | bx047687 | AT4G12490 | Bifunctional inhibitor/lipid-transfer protein/seed storage 2S albumin superfamily protein | 2.525 | 0.230 | 0.979 |
| 1 | 34052 | bx047705 | AT2G42280 | basic helix-loop-helix (bHLH) DNA-binding superfamily protein | 2.591 | 2.434 | 1.242 |
| 1 | 34224 | bx047918 | AT1G80840 | WRKY DNA-binding protein 40 | 3.780 | 2.796 | 0.647 |
| 1 | 34243 | bx047940 | AT5G06280 | unknown protein | 2.660 | 1.843 | 1.386 |
| 1 | 34495 | bx048251 |  |  | 4.279 | 3.218 | 1.845 |
| 1 | 34515 | bx048276 | AT5G48380 | BAK1-interacting receptor-like kinase 1 | 2.365 | 1.699 | -0.199 |
| 1 | 34615 | bx048399 | AT4G19645 | TRAM, LAG1 and CLN8 (TLC) lipid-sensing domain containing protein | 3.531 | -0.113 | 1.401 |
| 1 | 34703 | bx048505 | AT4G30780 | unknown protein | 0.346 | 2.347 | -0.374 |
| 1 | 34808 | bx048627 | AT2G26640 | 3-ketoacyl-CoA synthase 11 | 2.844 | 2.042 | 0.323 |
| 1 | 34890 | bx048739 | AT1G04770 | Tetratricopeptide repeat (TPR)-like superfamily protein | 4.004 | 3.409 | 1.566 |
| 1 | 35266 | bx049220 | AT5G56960 | basic helix-loop-helix (bHLH) DNA-binding family protein | 4.646 | 3.136 | -1.380 |
| 1 | 35453 | bx049452 | AT3G21250 | ARABIDOPSIS THALIANA MULTIDRUG RESISTANCE-ASSOCIATED PROTEIN 6, ATP-binding cassette C8, multidrug resistance-associated protein 6 | 3.179 | 2.821 | 2.322 |
| 1 | 35553 | bx049593 | AT2G19570 | cytidine deaminase 1 | 3.586 | 2.017 | 0.874 |
| 1 | 35572 | bx049621 | AT1G13510 | Protein of unknown function (DUF1262) | 2.904 | 2.732 | 0.843 |
| 1 | 35654 | bx049737 | AT4G04220 | receptor like protein 46 | -0.010 | 2.418 | -0.237 |
| 1 | 35815 | bx049958 | AT5G43470 | RECOGNITION OF PERONOSPORA PARASITICA 8, RESISTANT TO CMV(Y) 1, HYPERSENSITIVE RESPONSE TO TCV, Disease resistance protein (CC-NBS-LRR class) family | 2.553 | 0.030 | 0.252 |
| 1 | 36035 | bx050228 | AT1G07530 | ARABIDOPSIS THALIANA GRAS (GAI, RGA, SCR) 2, SCARECROW-like 14 | 2.796 | 2.757 | 1.173 |
| 1 | 36059 | bx050263 | AT2G38470 | WRKY DNA-binding protein 33 | 2.756 | 2.807 | 1.959 |
| 1 | 36105 | bx050317 | AT1G58170 | Disease resistance-responsive (dirigent-like protein) family protein | 4.389 | 3.824 | 0.790 |
| 1 | 36167 | bx050387 | AT1G79670 | RESISTANCE TO FUSARIUM OXYSPORUM 1, Wall-associated kinase family protein | 4.831 | 4.616 | 3.253 |
| 1 | 36250 | bx050481 |  |  | 3.277 | 2.752 | 0.461 |
| 1 | 36418 | bx050692 | AT4G19660 | NPR1-like protein 4 | 0.507 | 2.756 | -2.633 |
| 1 | 36501 | bx050796 | AT5G07360 | Amidase family protein | 3.258 | 4.024 | 1.940 |
| 1 | 36605 | bx050923 | AT3G12360 | INCREASED TOLERANCE TO NACL, Ankyrin repeat family protein | 4.286 | 4.245 | -0.181 |
| 1 | 36610 | bx050933 | AT3G21250 | ARABIDOPSIS THALIANA MULTIDRUG RESISTANCE-ASSOCIATED PROTEIN 6, ATP-binding cassette C8, multidrug resistance-associated protein 6 | 2.742 | 2.288 | 2.020 |
| 1 | 36634 | bx050959 | AT4G15120 | VQ motif-containing protein | 4.530 | 4.147 | 1.545 |
| 1 | 37002 | bx051438 |  |  | 3.933 | 3.241 | -0.063 |
| 1 | 37269 | bx051776 | AT5G60900 | receptor-like protein kinase 1 | 2.221 | 2.416 | 1.164 |
| 1 | 37458 | bx052006 | AT3G47110 | Leucine-rich repeat protein kinase family protein | 2.181 | 2.854 | 0.464 |
| 1 | 37764 | bx052393 | AT3G12500 | PATHOGENESIS-RELATED 3, basic chitinase | 4.001 | 3.115 | 0.841 |
| 1 | 38050 | bx052787 | AT1G73805 | SAR Deficient 1, Calmodulin binding protein-like | 4.367 | 4.112 | -0.061 |
| 1 | 38088 | bx052833 | AT5G11880 | Pyridoxal-dependent decarboxylase family protein | 6.150 | 3.562 | 0.533 |
| 1 | 38154 | bx052915 | AT5G54160 | O-methyltransferase 1 | 1.210 | 2.782 | 0.101 |
| 1 | 38293 | bx053093 | AT5G49690 | UDP-Glycosyltransferase superfamily protein | 3.213 | 2.767 | 1.237 |
| 1 | 38405 | bx053235 |  |  | 3.620 | 2.647 | 1.635 |
| 1 | 38499 | bx053367 |  |  | 3.762 | 3.313 | 0.179 |
| 1 | 38702 | bx053631 | AT5G07440 | glutamate dehydrogenase 2 | 2.773 | 2.476 | 1.423 |
| 1 | 38809 | bx053760 |  |  | 2.659 | 2.048 | 0.519 |
| 1 | 38840 | bx053804 | AT2G20840 | Secretory carrier membrane protein (SCAMP) family protein | 3.909 | 2.943 | -0.313 |
| 1 | 39027 | bx054044 | AT4G28460 | unknown protein | 4.088 | 1.761 | -0.018 |
| 1 | 39048 | bx054073 | AT4G19390 | Uncharacterised protein family (UPF0114) | 4.419 | 3.918 | 1.595 |
| 1 | 39088 | bx054124 | AT1G21270 | wall-associated kinase 2 | 2.085 | 4.394 | 0.508 |
| 1 | 39130 | bx054183 | AT1G29290 | unknown protein | 3.645 | 2.486 | 0.016 |
| 1 | 39217 | bx054306 | AT3G23250 | myb domain protein 15 | 5.380 | 5.847 | 3.602 |
| 1 | 39220 | bx054311 | AT4G12735 | unknown protein | 3.288 | 1.863 | 0.063 |
| 1 | 39231 | bx054330 | AT1G15460 | ARABIDOPSIS THALIANA REQUIRES HIGH BORON 4, HCO3- transporter family | 3.831 | 1.592 | -0.079 |
| 1 | 39371 | bx054534 | AT3G53330 | plastocyanin-like domain-containing protein | 3.214 | 3.221 | 1.401 |
| 1 | 39458 | bx054655 |  |  | 2.845 | 3.042 | 0.339 |
| 1 | 39494 | bx054702 | AT4G34200 | embryo sac development arrest 9, D-3-phosphoglycerate dehydrogenase | 2.967 | 2.578 | 1.398 |
| 1 | 39753 | bx055097 | AT4G33050 | embryo sac development arrest 39, calmodulin-binding family protein | 4.035 | 3.603 | 2.080 |
| 1 | 39962 | bx055382 | AT3G09550 | Ankyrin repeat family protein | 3.439 | 3.836 | -0.176 |
| 1 | 40009 | bx055437 |  |  | 4.625 | 3.203 | 1.619 |
| 1 | 40039 | bx055478 | AT3G27890 | NADPH:quinone oxidoreductase | 4.913 | 1.715 | 0.215 |
| 1 | 40186 | bx055661 |  |  | 1.858 | 2.482 | 1.259 |
| 1 | 40227 | bx055717 | AT3G12900 | 2-oxoglutarate (2OG) and Fe(II)-dependent oxygenase superfamily protein | 3.223 | 3.072 | 0.797 |
| 1 | 40337 | bx055849 |  |  | 4.182 | 3.309 | 2.929 |
| 1 | 40390 | bx055910 |  |  | 4.777 | 4.082 | 2.217 |
| 1 | 40438 | bx055967 | AT4G36810 | geranylgeranyl pyrophosphate synthase 1 | 7.134 | 6.464 | 3.718 |
| 1 | 40532 | bx056082 |  |  | 4.788 | 3.325 | 2.182 |
| 1 | 40789 | bx056404 | AT3G51680 | short-chain dehydrogenase/reductase 2, NAD(P)-binding Rossmann-fold superfamily protein | 5.222 | 4.109 | 1.959 |
| 1 | 41078 | bx056758 | AT5G48380 | BAK1-interacting receptor-like kinase 1 | 2.680 | 3.198 | -0.075 |
| 1 | 41397 | bx057140 | AT1G61190 | LRR and NB-ARC domains-containing disease resistance protein | 2.959 | 3.128 | -0.002 |
| 1 | 41648 | bx057456 |  |  | 2.166 | 2.516 | 0.929 |
| 1 | 41659 | bx057469 |  |  | 3.556 | 2.790 | 1.222 |
| 1 | 41878 | bx057735 |  |  | 5.968 | 5.701 | 3.816 |
| 1 | 42357 | bx058304 | AT5G37490 | ARM repeat superfamily protein | 4.617 | 3.579 | 2.181 |
| 1 | 42408 | bx058366 | AT1G14040 | EXS (ERD1/XPR1/SYG1) family protein | 3.991 | 4.474 | 2.537 |
| 1 | 42575 | bx058574 | AT5G36110 | cytochrome P450, family 716, subfamily A, polypeptide 1, cytochrome P450, family 716, subfamily A, polypeptide 1 | 5.605 | 3.760 | 2.786 |
| 1 | 42594 | bx058598 |  |  | 1.663 | 3.732 | 0.603 |
| 1 | 42674 | bx058694 | AT4G35160 | O-methyltransferase family protein | 2.927 | 2.345 | 0.571 |
| 1 | 42695 | bx058721 | AT1G27730 | salt tolerance zinc finger | 3.173 | 2.458 | 2.275 |
| 1 | 43216 | bx059366 | AT1G10040 | alpha/beta-Hydrolases superfamily protein | 2.111 | 2.799 | 0.928 |
| 1 | 43469 | bx059684 | AT1G80840 | WRKY DNA-binding protein 40 | 3.828 | 3.775 | 1.445 |
| 1 | 43606 | bx059858 | AT5G65300 | unknown protein | 3.639 | 1.767 | 0.039 |
| 1 | 43692 | bx059963 | AT3G51680 | short-chain dehydrogenase/reductase 2, NAD(P)-binding Rossmann-fold superfamily protein | 5.771 | 6.496 | 3.788 |
| 1 | 43794 | bx060083 |  |  | 2.689 | 1.169 | 0.560 |
| 1 | 43835 | bx060134 | AT4G18880 | ARABIDOPSIS THALIANA HEAT SHOCK TRANSCRIPTION FACTOR A4A, heat shock transcription factor A4A | 4.292 | 4.344 | 1.904 |
| 1 | 43891 | bx060207 |  |  | 4.170 | 1.520 | -0.947 |
| 1 | 44215 | bx060594 | AT1G32780 | GroES-like zinc-binding dehydrogenase family protein | 6.772 | 5.113 | 2.883 |
| 1 | 44434 | bx060860 |  |  | 4.855 | 3.754 | 1.848 |
| 1 | 44445 | bx060879 | AT4G17030 | expansin-like B1 | 5.091 | 5.307 | 3.511 |
| 1 | 44591 | bx061057 |  |  | 2.479 | 1.352 | 0.256 |
| 1 | 44856 | bx061380 | AT3G54450 | Major facilitator superfamily protein | 5.757 | 4.919 | 4.197 |
| 1 | 44985 | bx061538 |  |  | 1.960 | 2.780 | 1.035 |
| 1 | 45120 | bx061708 | AT3G54200 | Late embryogenesis abundant (LEA) hydroxyproline-rich glycoprotein family | 3.417 | 1.577 | 0.755 |
| 1 | 45195 | bx061802 | AT4G34135 | UDP-glucosyltransferase 73B2 | 1.609 | 2.904 | -0.517 |
| 1 | 45273 | bx061898 |  |  | 3.837 | 1.017 | 0.356 |
| 1 | 45764 | bx062508 | AT4G23810 | WRKY family transcription factor | 2.839 | 2.874 | 0.011 |
| 1 | 45831 | bx062602 | AT4G02780 | GA REQUIRING 1, CPP synthase, ARABIDOPSIS THALIANA ENT-COPALYL DIPHOSPHATE SYNTHETASE 1, Terpenoid cyclases/Protein prenyltransferases superfamily protein | 5.747 | 4.189 | 2.634 |
| 1 | 46004 | bx062832 | AT1G51760 | JASMONIC ACID RESPONSIVE 3, IAA-ALANINE RESISTANT 3, peptidase M20/M25/M40 family protein | 5.755 | 4.273 | 3.932 |
| 1 | 46271 | bx063180 | AT3G61510 | ARABIDOPSIS THALIANA 1-AMINOCYCLOPROPANE-1-CARBOXYLATE SYNTHASE 1, ACC synthase 1 | 3.190 | 2.625 | 0.091 |
| 1 | 46708 | bx063711 | AT1G76360 | Protein kinase superfamily protein | 2.435 | 2.447 | -0.779 |
| 1 | 46802 | bx063818 | AT5G02490 | Heat shock protein 70 (Hsp 70) family protein | 2.312 | 2.305 | 1.146 |
| 1 | 46839 | bx063865 |  |  | 5.596 | 5.570 | 3.849 |
| 1 | 46840 | bx063867 | AT1G32928 | unknown protein | 2.080 | 3.253 | 1.181 |
| 1 | 46871 | bx063905 |  |  | 2.625 | 1.925 | 0.672 |
| 1 | 46879 | bx063914 | AT5G12340 | unknown protein | 3.544 | 2.700 | 0.013 |
| 1 | 47212 | bx064289 |  |  | 2.410 | 1.573 | 1.255 |
| 1 | 47557 | bx064680 |  |  | 2.966 | 1.385 | 1.834 |
| 1 | 47948 | bx065113 | AT3G28580 | P-loop containing nucleoside triphosphate hydrolases superfamily protein | 3.631 | 2.930 | 0.491 |
| 1 | 48315 | bx065541 | AT1G22065 | unknown protein | 3.760 | 1.572 | 0.456 |
| 1 | 48368 | bx065615 |  |  | 4.986 | 4.949 | 3.344 |
| 1 | 48422 | bx065689 |  |  | 3.342 | 1.825 | 1.455 |
| 1 | 48658 | bx065987 | AT3G52400 | syntaxin of plants 122 | 4.451 | 3.564 | 1.151 |
| 1 | 48692 | bx066030 |  |  | 2.713 | 2.925 | 0.630 |
| 1 | 48764 | bx066126 | AT3G22590 | PLANT HOMOLOGOUS TO PARAFIBROMIN | 1.889 | 2.546 | 0.287 |
| 1 | 48786 | bx066153 | AT2G38470 | WRKY DNA-binding protein 33 | 3.576 | 2.952 | 0.154 |
| 1 | 48905 | bx066301 | AT4G02860 | Phenazine biosynthesis PhzC/PhzF protein | 2.433 | 2.721 | 1.711 |
| 1 | 49026 | bx066461 | AT1G02205 | ECERIFERUM 1, Fatty acid hydroxylase superfamily | 4.317 | 3.836 | 2.966 |
| 1 | 49230 | bx066711 |  |  | 4.217 | 4.051 | 1.624 |
| 1 | 49269 | bx066757 | AT1G01120 | 3-ketoacyl-CoA synthase 1 | 2.902 | 0.898 | 0.005 |
| 1 | 49325 | bx066823 |  |  | 1.939 | 3.440 | -0.490 |
| 1 | 49617 | bx067160 | AT5G24600 | Protein of unknown function, DUF599 | 2.541 | 1.259 | 0.802 |
| 1 | 50164 | bx067801 |  |  | 2.622 | 1.533 | 1.719 |
| 1 | 51039 | bx068840 |  |  | 2.595 | 2.803 | 1.616 |
| 1 | 51509 | bx069511 |  |  | 2.453 | 2.357 | 1.572 |
| 1 | 52023 | bx070284 | AT4G24480 | Protein kinase superfamily protein | 4.535 | 3.970 | 2.034 |
| 1 | 52600 | bx071140 | AT3G23230 | Integrase-type DNA-binding superfamily protein | 5.864 | 5.631 | 4.112 |
| 1 | 52795 | bx071462 | AT3G51790 | transmembrane protein G1P-related 1 | 2.973 | 2.450 | 1.244 |
| 1 | 52856 | bx071564 |  |  | 3.185 | 2.630 | 2.263 |
| 1 | 53009 | bx071749 |  |  | 4.966 | 3.239 | -0.174 |
| 1 | 53108 | bx071860 | AT5G22380 | NAC domain containing protein 90 | 3.524 | 1.961 | 0.506 |
| 1 | 53123 | bx071877 |  |  | 3.875 | 1.692 | 2.235 |
| 1 | 53594 | bx072417 | AT3G60160 | ATP-binding cassette C9, multidrug resistance-associated protein 9 | 2.351 | 0.664 | 0.854 |
| 1 | 53625 | bx072454 | AT2G47485 | unknown protein | 3.604 | 0.882 | -0.155 |
| 1 | 53762 | bx072621 |  |  | 2.778 | 1.460 | 1.359 |
| 1 | 53864 | bx072741 | AT5G39390 | Leucine-rich repeat protein kinase family protein | 3.257 | 2.682 | -0.405 |
| 1 | 53894 | bx072777 |  |  | 3.132 | 3.115 | 1.000 |
| 1 | 53920 | bx072808 | AT4G34050 | caffeoyl coenzyme A O-methyltransferase 1, S-adenosyl-L-methionine-dependent methyltransferases superfamily protein | 3.186 | 2.825 | 2.061 |
| 1 | 53923 | bx072811 |  |  | 2.907 | 2.413 | 0.951 |
| 1 | 54028 | bx072929 |  |  | 4.615 | 4.406 | 2.490 |
| 1 | 54111 | bx073024 |  |  | 2.634 | 1.098 | 1.559 |
| 1 | 54529 | bx073502 |  |  | 2.988 | 3.095 | 1.878 |
| 1 | 54654 | bx073642 | AT5G17540 | HXXXD-type acyl-transferase family protein | 2.790 | 2.129 | -0.750 |
| 1 | 54943 | bx073950 |  |  | 4.994 | 3.876 | 1.110 |
| 1 | 55402 | bx074529 | AT4G34050 | caffeoyl coenzyme A O-methyltransferase 1, S-adenosyl-L-methionine-dependent methyltransferases superfamily protein | 3.144 | 2.730 | 1.985 |
| 1 | 55420 | bx074552 | AT3G06350 | MATERNAL EFFECT EMBRYO ARREST 32, EMBRYO DEFECTIVE 3004, dehydroquinate dehydratase, putative / shikimate dehydrogenase, putative | 3.451 | 1.914 | 0.534 |
| 1 | 55567 | bx074734 | AT2G25060 | early nodulin-like protein 14 | 3.852 | 3.866 | 1.985 |
| 1 | 55591 | bx074760 |  |  | 2.435 | 2.685 | 1.319 |
| 1 | 55625 | bx074814 | AT1G14040 | EXS (ERD1/XPR1/SYG1) family protein | 3.660 | 3.960 | 1.941 |
| 1 | 55918 | bx075296 | AT4G20970 | basic helix-loop-helix (bHLH) DNA-binding superfamily protein | 2.446 | 2.883 | 1.608 |
| 1 | 55991 | bx075404 | AT1G66120 | AMP-dependent synthetase and ligase family protein | 4.955 | 4.778 | 3.292 |
| 1 | 56001 | bx075433 | AT2G46330 | arabinogalactan protein 16 | 3.016 | 2.496 | 1.126 |
| 1 | 56003 | bx075436 |  |  | 4.348 | 5.254 | 3.032 |
| 1 | 56022 | bx075473 | AT2G36730 | Pentatricopeptide repeat (PPR) superfamily protein | 3.116 | 3.502 | 1.537 |
| 1 | 56053 | bx075525 |  |  | 4.360 | 1.688 | 1.202 |
| 1 | 56141 | bx075631 | AT3G48280 | cytochrome P450, family 71, subfamily A, polypeptide 25, cytochrome P450, family 71, subfamily A, polypeptide 25 | 4.241 | 4.181 | 1.759 |
| 1 | 56201 | bx075703 |  |  | 4.733 | 4.402 | 0.124 |
| 1 | 56245 | bx075766 | AT5G14310 | carboxyesterase 16 | 2.517 | 2.208 | 0.585 |
| 1 | 56251 | bx075772 | AT3G01830 | Calcium-binding EF-hand family protein | 4.840 | 3.537 | -0.315 |
| 1 | 56335 | bx075902 |  |  | 3.782 | 3.889 | 0.743 |
| 1 | 56386 | bx075966 | AT5G36160 | Tyrosine transaminase family protein | 2.788 | 1.655 | 0.227 |
| 1 | 56497 | bx076097 | AT3G07700 | Protein kinase superfamily protein | 3.068 | 1.355 | 0.972 |
| 1 | 56746 | bx076381 |  |  | 4.525 | 3.377 | -0.113 |
| 1 | 56976 | bx076626 | AT5G40850 | urophorphyrin methylase 1 | 2.699 | 2.371 | 1.648 |
| 1 | 57279 | bx076999 |  |  | 3.727 | 3.183 | 2.015 |
| 1 | 57335 | bx077071 | AT4G08850 | Leucine-rich repeat receptor-like protein kinase family protein | 2.347 | 1.210 | 0.141 |
| 1 | 57433 | bx077216 | AT4G17215 | Pollen Ole e 1 allergen and extensin family protein | 3.062 | 1.713 | 0.016 |
| 1 | 57454 | bx077242 |  |  | 3.242 | 3.085 | 0.018 |
| 1 | 57579 | bx077395 |  |  | 5.155 | 4.627 | 0.812 |
| 1 | 57587 | bx077405 | AT5G60900 | receptor-like protein kinase 1 | 2.710 | 2.111 | 0.319 |
| 1 | 57697 | bx077531 | AT5G25930 | Protein kinase family protein with leucine-rich repeat domain | 2.645 | 0.954 | -1.016 |
| 1 | 57910 | bx077794 | AT5G22860 | Serine carboxypeptidase S28 family protein | 3.161 | 1.988 | 0.230 |
| 1 | 58503 | bx078488 |  |  | 5.107 | 4.896 | 3.575 |
| 1 | 58710 | bx078735 |  |  | 3.041 | 1.783 | -0.132 |
| 1 | 58724 | bx078753 | AT1G32700 | PLATZ transcription factor family protein | 2.787 | 2.272 | 0.610 |
| 1 | 58754 | bx078791 |  |  | 3.777 | 4.249 | -0.189 |
| 1 | 58792 | bx078841 | AT3G16340 | ATP-binding cassette G29, pleiotropic drug resistance 1 | 2.722 | 0.679 | -0.357 |
| 1 | 59133 | bx079245 |  |  | 2.392 | 0.999 | 1.711 |
| 1 | 59479 | bx079644 |  |  | 3.061 | 0.790 | 0.017 |
| 1 | 59511 | bx079681 | AT3G47580 | Leucine-rich repeat protein kinase family protein | 2.478 | 2.119 | 1.520 |
| 1 | 59955 | bx080237 | AT3G03060 | P-loop containing nucleoside triphosphate hydrolases superfamily protein | 2.016 | 2.472 | 1.413 |
| 1 | 60504 | bx080946 | AT5G60900 | receptor-like protein kinase 1 | 2.377 | 2.212 | 1.262 |
| 1 | 60688 | bx081179 |  |  | 1.490 | 2.342 | 0.895 |
| 1 | 60934 | bx081488 | AT2G36090 | F-box family protein | 3.229 | 0.886 | 0.391 |
| 1 | 61013 | bx081587 | AT1G19390 | Wall-associated kinase family protein | 3.834 | 4.160 | 2.373 |
| 1 | 61059 | bx081639 |  |  | 3.255 | 2.649 | 1.756 |
| 1 | 61102 | bx081688 | AT1G59870 | PENETRATION 3, ARABIDOPSIS PLEIOTROPIC DRUG RESISTANCE 8, Arabidopsis thaliana ATP-binding cassette G36, ATP-binding cassette G36, ABC-2 and Plant PDR ABC-type transporter family protein | 2.964 | 1.818 | 0.462 |
| 1 | 61259 | bx081907 | AT4G31800 | ARABIDOPSIS THALIANA WRKY DNA-BINDING PROTEIN 18, WRKY DNA-binding protein 18 | 3.813 | 3.786 | 1.602 |
| 1 | 61347 | bx082043 |  |  | 2.886 | 4.900 | 1.111 |
| 1 | 61362 | bx082070 |  |  | 3.101 | 2.713 | 2.160 |
| 1 | 61447 | bx082199 | AT3G57830 | Leucine-rich repeat protein kinase family protein | 2.631 | 1.180 | 1.421 |
| 1 | 61630 | bx082492 | AT2G26190 | calmodulin-binding family protein | 3.126 | 3.144 | 1.444 |
| 2 | 376 | bx000669 | AT4G19950 | unknown protein | -2.339 | -1.524 | -1.846 |
| 2 | 412 | bx000734 | AT1G43650 | nodulin MtN21 /EamA-like transporter family protein | -3.005 | -2.323 | -2.312 |
| 2 | 523 | bx000923 | AT1G69910 | Protein kinase superfamily protein | -2.258 | -1.256 | -2.334 |
| 2 | 555 | bx000981 | AT5G52780 | Protein of unknown function (DUF3464) | -2.125 | -2.674 | -1.030 |
| 2 | 595 | bx001052 | AT4G21760 | beta-glucosidase 47 | -5.390 | -3.694 | -3.887 |
| 2 | 1068 | bx001825 | AT3G13720 | PRENYLATED RAB ACCEPTOR 1.F3, PRA1 (Prenylated rab acceptor) family protein | -2.427 | -2.333 | -2.057 |
| 2 | 1294 | bx002184 |  |  | -2.683 | -2.591 | -2.922 |
| 2 | 1354 | bx002282 | AT3G28890 | receptor like protein 43 | -3.227 | -2.829 | -3.379 |
| 2 | 1489 | bx002481 | AT1G34640 | peptidases | -2.446 | -1.878 | -2.031 |
| 2 | 2023 | bx003304 | AT4G32140 | EamA-like transporter family | -1.367 | -2.335 | -1.114 |
| 2 | 2040 | bx003332 | AT2G46940 | unknown protein | -3.297 | -3.138 | -2.191 |
| 2 | 2084 | bx003391 |  |  | -2.873 | -2.514 | -1.875 |
| 2 | 2172 | bx003523 | AT4G14770 | TESMIN/TSO1-like CXC 2 | -2.799 | -2.098 | -2.300 |
| 2 | 2180 | bx003534 |  |  | -2.517 | -2.566 | -1.983 |
| 2 | 2430 | bx003905 | AT4G21960 | Peroxidase superfamily protein | -3.193 | -2.787 | -2.608 |
| 2 | 2434 | bx003914 | AT1G55850 | cellulose synthase like E1 | -3.063 | -2.851 | -1.725 |
| 2 | 2440 | bx003921 | AT1G73660 | protein tyrosine kinase family protein | -2.355 | -2.755 | -1.667 |
| 2 | 2491 | bx004006 | AT2G05160 | CCCH-type zinc fingerfamily protein with RNA-binding domain | -2.147 | -2.604 | -2.292 |
| 2 | 2610 | bx004188 | AT5G09820 | Plastid-lipid associated protein PAP / fibrillin family protein | -2.612 | -1.938 | -1.292 |
| 2 | 2683 | bx004299 | AT5G22860 | Serine carboxypeptidase S28 family protein | -3.331 | -4.014 | -2.432 |
| 2 | 3022 | bx004795 | AT1G60790 | TRICHOME BIREFRINGENCE-LIKE 2, Plant protein of unknown function (DUF828) | -2.377 | -1.753 | -1.529 |
| 2 | 3280 | bx005167 | AT2G39730 | rubisco activase | -0.959 | -2.853 | -1.135 |
| 2 | 3452 | bx005420 | AT5G14750 | WEREWOLF 1, WEREWOLF, myb domain protein 66 | -4.295 | -3.337 | -1.900 |
| 2 | 3510 | bx005501 | AT5G16000 | NSP-interacting kinase 1 | -2.853 | -1.696 | -3.005 |
| 2 | 3535 | bx005542 | AT5G23100 | Protein of unknown function, DUF617 | -2.773 | -1.611 | -2.458 |
| 2 | 3544 | bx005561 | AT2G46500 | UBIQUITIN-LIKE DOMAIN KINASE GAMMA 4, phosphoinositide 4-kinase gamma 4 | -1.794 | -2.325 | -2.019 |
| 2 | 3590 | bx005625 | AT5G12900 | unknown protein | -2.771 | -2.412 | -2.613 |
| 2 | 3605 | bx005648 | AT5G22620 | phosphoglycerate/bisphosphoglycerate mutase family protein | -0.701 | -2.463 | -0.363 |
| 2 | 3669 | bx005733 | AT2G01950 | VASCULAR HIGHWAY 1, BRI1-like 2 | -2.327 | -2.330 | -2.537 |
| 2 | 3705 | bx005789 |  |  | -3.168 | -2.826 | -2.859 |
| 2 | 3732 | bx005829 | AT3G26210 | cytochrome P450, family 71, subfamily B, polypeptide 23, cytochrome P450, family 71, subfamily B, polypeptide 23 | -2.375 | -2.657 | -1.943 |
| 2 | 3861 | bx006007 | AT4G12800 | photosystem I subunit l | -2.883 | -2.055 | -1.936 |
| 2 | 3952 | bx006151 |  |  | -2.330 | -1.096 | -1.638 |
| 2 | 4056 | bx006301 |  |  | -3.243 | -3.100 | -2.536 |
| 2 | 4110 | bx006382 | AT2G13360 | L-serine:glyoxylate aminotransferase, ALANINE:GLYOXYLATE AMINOTRANSFERASE 1, alanine:glyoxylate aminotransferase | -1.515 | -2.466 | -1.207 |
| 2 | 4140 | bx006421 | AT2G39730 | rubisco activase | -1.434 | -3.939 | -1.500 |
| 2 | 4371 | bx006768 | AT4G39870 | TLD-domain containing nucleolar protein | -3.012 | -3.346 | -2.921 |
| 2 | 4496 | bx006949 | AT5G43300 | glycerophosphodiester phosphodiesterase 3, PLC-like phosphodiesterases superfamily protein | -1.352 | -2.422 | -1.596 |
| 2 | 4509 | bx006968 | AT2G42200 | squamosa promoter binding protein-like 9 | -3.009 | -4.065 | -2.896 |
| 2 | 4513 | bx006973 | AT2G44500 | O-fucosyltransferase family protein | -2.372 | -2.057 | -2.191 |
| 2 | 4525 | bx006987 | AT4G32980 | homeobox gene 1 | -3.268 | -3.329 | -2.854 |
| 2 | 4534 | bx007003 | AT1G30700 | FAD-binding Berberine family protein | -3.377 | -3.136 | -2.144 |
| 2 | 4545 | bx007019 | AT4G27730 | ARABIDOPSIS THALIANA OLIGOPEPTIDE TRANSPORTER 6, oligopeptide transporter 1 | -3.119 | -3.481 | -1.617 |
| 2 | 4583 | bx007066 |  |  | -2.297 | -2.398 | -1.878 |
| 2 | 4629 | bx007139 | AT4G12690 | Plant protein of unknown function (DUF868) | -2.403 | -1.533 | -2.442 |
| 2 | 4665 | bx007195 | AT2G01918 | PsbQ-like 3 | -2.788 | -2.784 | -1.404 |
| 2 | 4757 | bx007319 | AT2G42200 | squamosa promoter binding protein-like 9 | -4.215 | -3.239 | -3.639 |
| 2 | 4773 | bx007345 | AT3G63060 | EID1-like 3 | -2.089 | -3.867 | -0.864 |
| 2 | 4807 | bx007397 | AT5G65700 | BARELY ANY MERISTEM 1, Leucine-rich receptor-like protein kinase family protein | -2.313 | -2.502 | -2.012 |
| 2 | 4905 | bx007534 | AT5G50570 | SQUAMOSA PROMOTER-BINDING PROTEIN LIKE 13A, SQUAMOSA PROMOTER-BINDING PROTEIN LIKE 13, Squamosa promoter-binding protein-like (SBP domain) transcription factor family protein | -3.730 | -3.680 | -3.913 |
| 2 | 5051 | bx007747 | AT1G55850 | cellulose synthase like E1 | -2.698 | -2.350 | -1.053 |
| 2 | 5087 | bx007796 | AT5G54530 | Protein of unknown function, DUF538 | -2.802 | -2.036 | -2.178 |
| 2 | 5098 | bx007817 |  |  | -2.822 | -2.850 | -2.838 |
| 2 | 5228 | bx007991 |  |  | -2.397 | -2.033 | -2.622 |
| 2 | 5402 | bx008243 | AT1G31810 | Formin Homology 14 | -1.823 | -2.600 | -1.039 |
| 2 | 5424 | bx008266 | AT3G63250 | HOMOCYSTEINE METHYLTRANSFERASE-2, homocysteine methyltransferase 2 | -2.418 | -2.256 | -1.806 |
| 2 | 5448 | bx008305 | AT1G09530 | PHOTOCURRENT 1, PHYTOCHROME-ASSOCIATED PROTEIN 3, phytochrome interacting factor 3 | -3.173 | -3.552 | -2.260 |
| 2 | 5634 | bx008574 |  |  | -2.665 | -2.443 | -1.833 |
| 2 | 5645 | bx008588 | AT3G10910 | RING/U-box superfamily protein | -2.894 | -2.210 | -2.191 |
| 2 | 5796 | bx008809 | AT1G67750 | Pectate lyase family protein | -1.999 | -3.530 | -2.209 |
| 2 | 5924 | bx008973 | AT4G36360 | beta-galactosidase 3 | -2.579 | -2.156 | -2.087 |
| 2 | 5952 | bx009005 | AT1G29300 | unfertilized embryo sac 1, Plant protein of unknown function (DUF641) | -2.509 | -1.844 | -1.808 |
| 2 | 6113 | bx009221 | AT5G13840 | FIZZY-related 3 | -1.735 | -2.941 | -2.029 |
| 2 | 6149 | bx009275 | AT5G28650 | ARABIDOPSIS THALIANA WRKY DNA-BINDING PROTEIN 74, WRKY DNA-binding protein 74 | -2.780 | -1.437 | -2.047 |
| 2 | 6234 | bx009397 | AT1G67570 | Protein of unknown function (DUF3537) | -2.444 | -2.193 | -2.406 |
| 2 | 6238 | bx009401 | AT2G41990 | unknown protein | -2.722 | -1.372 | -1.862 |
| 2 | 6303 | bx009491 | AT2G19810 | Oxidation-related Zinc Finger 1, CCCH-type zinc finger family protein | -3.090 | -3.127 | -2.329 |
| 2 | 6319 | bx009508 | AT5G67390 | unknown protein | -2.459 | -1.302 | -1.782 |
| 2 | 6345 | bx009553 |  |  | -3.019 | -2.736 | -2.152 |
| 2 | 6361 | bx009579 | AT3G50685 | unknown protein | -2.944 | -3.059 | -2.241 |
| 2 | 6541 | bx009849 | AT5G24080 | Protein kinase superfamily protein | -2.632 | -2.244 | -2.143 |
| 2 | 6611 | bx009948 | AT4G27710 | cytochrome P450, family 709, subfamily B, polypeptide 3, cytochrome P450, family 709, subfamily B, polypeptide 3 | -2.467 | -3.055 | -0.617 |
| 2 | 6688 | bx010052 | AT1G50180 | NB-ARC domain-containing disease resistance protein | -2.930 | -2.188 | -2.653 |
| 2 | 6705 | bx010076 | AT3G26300 | cytochrome P450, family 71, subfamily B, polypeptide 34, cytochrome P450, family 71, subfamily B, polypeptide 34 | -3.376 | -4.107 | -2.738 |
| 2 | 6714 | bx010091 | AT5G41040 | HXXXD-type acyl-transferase family protein | -3.150 | -2.842 | -3.337 |
| 2 | 6730 | bx010112 | AT5G12080 | mechanosensitive channel of small conductance-like 10 | -2.624 | -2.128 | -2.940 |
| 2 | 6738 | bx010125 | AT4G32980 | homeobox gene 1 | -2.495 | -3.105 | -2.642 |
| 2 | 6809 | bx010216 |  |  | -2.857 | -2.915 | -1.190 |
| 2 | 6874 | bx010303 | AT5G17050 | UDP-glucosyl transferase 78D2 | -3.172 | -2.822 | -2.078 |
| 2 | 6877 | bx010306 | AT4G38430 | rho guanyl-nucleotide exchange factor 1 | -2.592 | -1.027 | -2.061 |
| 2 | 6891 | bx010326 | AT5G47640 | nuclear factor Y, subunit B2, nuclear factor Y, subunit B2 | -3.148 | -2.420 | -1.953 |
| 2 | 6989 | bx010473 |  |  | -2.477 | -1.977 | -2.310 |
| 2 | 7020 | bx010520 | AT5G47240 | nudix hydrolase homolog 8 | -2.843 | -2.345 | -2.553 |
| 2 | 7077 | bx010607 | AT4G32980 | homeobox gene 1 | -2.608 | -3.234 | -2.945 |
| 2 | 7096 | bx010633 | AT4G30130 | Protein of unknown function (DUF630 and DUF632) | -2.817 | -2.279 | -3.082 |
| 2 | 7246 | bx010831 | AT3G55550 | Concanavalin A-like lectin protein kinase family protein | -2.604 | -2.035 | -2.362 |
| 2 | 7348 | bx010973 | AT1G55480 | protein containing PDZ domain, a K-box domain, and a TPR region | -1.076 | -2.411 | -1.119 |
| 2 | 7353 | bx010981 | AT4G29310 | Protein of unknown function (DUF1005) | -2.436 | -1.903 | -2.353 |
| 2 | 7361 | bx010990 | AT2G04480 | unknown protein | -2.669 | -1.267 | -2.631 |
| 2 | 7463 | bx011139 | AT1G55850 | cellulose synthase like E1 | -2.731 | -2.354 | -1.088 |
| 2 | 7591 | bx011315 | AT1G55850 | cellulose synthase like E1 | -2.011 | -2.360 | -2.058 |
| 2 | 7762 | bx011569 | AT3G19320 | Leucine-rich repeat (LRR) family protein | -2.690 | -0.804 | -2.337 |
| 2 | 7823 | bx011668 | AT4G09890 | Protein of unknown function (DUF3511) | -2.043 | -2.731 | -2.174 |
| 2 | 7904 | bx011779 | AT1G68725 | arabinogalactan protein 19 | -2.457 | -1.696 | -2.697 |
| 2 | 7993 | bx011895 |  |  | -2.320 | -2.327 | -1.257 |
| 2 | 7998 | bx011903 | AT5G39530 | Protein of unknown function (DUF1997) | -2.475 | -3.094 | -1.962 |
| 2 | 8075 | bx012004 | AT1G29240 | Protein of unknown function (DUF688) | -2.428 | -1.459 | -1.723 |
| 2 | 8373 | bx012423 | AT4G29090 | Ribonuclease H-like superfamily protein | -2.743 | -2.875 | -2.749 |
| 2 | 8393 | bx012453 | AT1G11340 | S-locus lectin protein kinase family protein | -2.143 | -3.697 | -2.628 |
| 2 | 8457 | bx012548 |  |  | -2.561 | -2.106 | -1.315 |
| 2 | 8558 | bx012695 | AT4G32272 | Nucleotide/sugar transporter family protein | -2.381 | -4.288 | -2.047 |
| 2 | 8599 | bx012752 | AT4G27800 | PROTEIN PHOSPHATASE 1, thylakoid-associated phosphatase 38 | -2.544 | -1.914 | -1.380 |
| 2 | 8722 | bx012924 | AT4G33000 | SOS3-LIKE CALCIUM BINDING PROTEIN 8, calcineurin B-like protein 10 | -2.409 | -2.971 | -0.569 |
| 2 | 8737 | bx012942 | AT4G24050 | NAD(P)-binding Rossmann-fold superfamily protein | -2.624 | -3.767 | -2.361 |
| 2 | 8917 | bx013192 | AT3G48660 | Protein of unknown function (DUF 3339) | -4.570 | -4.389 | -3.674 |
| 2 | 9080 | bx013434 | AT1G75500 | Walls Are Thin 1 | -2.440 | -2.010 | -2.340 |
| 2 | 9135 | bx013524 | AT4G10310 | high-affinity K+ transporter 1, high-affinity K+ transporter 1 | -3.314 | -3.215 | -1.949 |
| 2 | 9455 | bx013993 | AT5G52780 | Protein of unknown function (DUF3464) | -1.945 | -2.741 | -2.048 |
| 2 | 9554 | bx014125 | AT5G58960 | GRAVITROPIC IN THE LIGHT, Plant protein of unknown function (DUF641) | -2.788 | -2.865 | -2.740 |
| 2 | 9722 | bx014371 |  |  | -2.396 | -1.711 | -2.185 |
| 2 | 9769 | bx014439 | AT4G21760 | beta-glucosidase 47 | -5.470 | -3.976 | -3.658 |
| 2 | 9887 | bx014600 | AT3G21790 | UDP-Glycosyltransferase superfamily protein | -3.432 | -2.114 | -3.461 |
| 2 | 9903 | bx014620 | AT4G14770 | TESMIN/TSO1-like CXC 2 | -3.230 | -2.832 | -2.640 |
| 2 | 10320 | bx015260 | AT1G11720 | starch synthase 3 | -2.344 | -2.240 | -0.799 |
| 2 | 10721 | bx015922 | AT2G47240 | LONG-CHAIN ACYL-COA SYNTHASE 1, ECERIFERUM 8, AMP-dependent synthetase and ligase family protein | -2.441 | -2.188 | -1.717 |
| 2 | 11182 | bx016668 |  |  | -3.607 | -2.072 | -2.795 |
| 2 | 11864 | bx017752 | AT5G58090 | O-Glycosyl hydrolases family 17 protein | -2.515 | -1.785 | -1.359 |
| 2 | 12032 | bx018036 |  |  | -3.007 | -2.472 | -2.801 |
| 2 | 12099 | bx018159 | AT5G17170 | enhancer of sos3-1, rubredoxin family protein | -3.312 | -2.836 | -2.097 |
| 2 | 12368 | bx018555 | AT5G17680 | disease resistance protein (TIR-NBS-LRR class), putative | -3.041 | -2.450 | -3.230 |
| 2 | 12721 | bx019040 | AT1G17180 | glutathione S-transferase TAU 25 | -2.664 | -2.130 | -2.791 |
| 2 | 13126 | bx019596 | AT3G05160 | Major facilitator superfamily protein | -1.839 | -2.659 | -1.408 |
| 2 | 13189 | bx019681 | AT5G20190 | Tetratricopeptide repeat (TPR)-like superfamily protein | -1.371 | -2.595 | -1.602 |
| 2 | 13505 | bx020104 | AT1G74940 | Protein of unknown function (DUF581) | -2.607 | -1.720 | -1.475 |
| 2 | 13641 | bx020280 | AT1G08830 | copper/zinc superoxide dismutase 1 | -3.437 | -1.747 | -3.725 |
| 2 | 13776 | bx020447 | AT3G50820 | OXYGEN EVOLVING COMPLEX SUBUNIT 33 KDA, photosystem II subunit O-2 | -2.313 | -2.185 | -1.380 |
| 2 | 14093 | bx020872 | AT2G32440 | ARABIDOPSIS ENT-KAURENOIC ACID HYDROXYLASE 2, ent-kaurenoic acid hydroxylase 2 | -3.374 | -2.372 | -2.076 |
| 2 | 14247 | bx021083 | AT3G48200 | unknown protein | -2.631 | -3.109 | -1.976 |
| 2 | 14283 | bx021137 | AT5G12080 | mechanosensitive channel of small conductance-like 10 | -3.723 | -3.600 | -2.654 |
| 2 | 14318 | bx021182 | AT1G68520 | B-box type zinc finger protein with CCT domain | -3.040 | -2.103 | -1.741 |
| 2 | 14339 | bx021209 | AT5G51550 | EXORDIUM like 3 | -2.481 | -1.625 | -2.050 |
| 2 | 14507 | bx021419 | AT2G43060 | ILI1 binding bHLH 1 | -3.036 | -2.295 | -1.893 |
| 2 | 14541 | bx021465 | AT1G02205 | ECERIFERUM 1, Fatty acid hydroxylase superfamily | -3.680 | -2.466 | -2.491 |
| 2 | 14645 | bx021608 | AT5G38260 | Protein kinase superfamily protein | -2.965 | -3.023 | -2.757 |
| 2 | 14927 | bx021979 | AT4G37340 | cytochrome P450, family 81, subfamily D, polypeptide 3, cytochrome P450, family 81, subfamily D, polypeptide 3 | -3.060 | -3.124 | -1.755 |
| 2 | 14989 | bx022058 | AT5G20935 | unknown protein | -2.256 | -2.713 | -1.316 |
| 2 | 15061 | bx022154 | AT1G74880 | NADH dehydrogenase-like complex ), NAD(P)H:plastoquinone dehydrogenase complex subunit O | -1.704 | -2.694 | -1.746 |
| 2 | 15062 | bx022155 | AT1G03010 | Phototropic-responsive NPH3 family protein | -4.576 | -4.030 | -3.708 |
| 2 | 15129 | bx022242 | AT3G16250 | Photosynthetic NDH subcomplex B 3, NDH-dependent cyclic electron flow 1 | -4.039 | -4.007 | -2.764 |
| 2 | 15188 | bx022322 | AT1G63930 | from the Czech 'roh' meaning 'corner' | -2.359 | -1.832 | -2.412 |
| 2 | 15326 | bx022499 | ATMG00820 | Reverse transcriptase (RNA-dependent DNA polymerase) | -2.969 | -3.119 | -2.707 |
| 2 | 15404 | bx022604 | AT5G42760 | Leucine carboxyl methyltransferase | -1.872 | -2.687 | -2.077 |
| 2 | 15430 | bx022632 | AT4G35090 | catalase 2 | -2.304 | -2.057 | -2.104 |
| 2 | 15571 | bx022819 | AT5G55580 | Mitochondrial transcription termination factor family protein | -3.699 | -3.327 | -3.973 |
| 2 | 15664 | bx022941 | AT1G45474 | photosystem I light harvesting complex gene 5 | -2.540 | -2.497 | -1.337 |
| 2 | 15908 | bx023267 | AT1G23090 | sulfate transporter 91 | -2.772 | -2.592 | -2.019 |
| 2 | 15938 | bx023308 | AT2G24280 | alpha/beta-Hydrolases superfamily protein | -1.489 | -2.877 | -1.802 |
| 2 | 16041 | bx023448 | AT1G75100 | J-domain protein required for chloroplast accumulation response 1 | -2.913 | -2.339 | -1.479 |
| 2 | 16045 | bx023454 |  |  | -2.999 | -3.110 | -1.884 |
| 2 | 16163 | bx023623 | AT4G13500 | unknown protein | -2.365 | -2.278 | -1.779 |
| 2 | 16264 | bx023764 | AT1G60470 | galactinol synthase 4 | -2.322 | -2.108 | -2.230 |
| 2 | 16293 | bx023800 |  |  | -2.114 | -2.412 | -0.295 |
| 2 | 16368 | bx023912 | AT1G51400 | Photosystem II 5 kD protein | -2.676 | -2.625 | -1.865 |
| 2 | 16469 | bx024041 | AT5G11590 | TINY2, Integrase-type DNA-binding superfamily protein | -2.402 | -2.475 | -2.270 |
| 2 | 16539 | bx024130 | AT1G01780 | PLIM2b, GATA type zinc finger transcription factor family protein | -4.308 | -4.317 | -4.679 |
| 2 | 16581 | bx024194 | AT3G05900 | neurofilament protein-related | -2.462 | -2.198 | -2.063 |
| 2 | 16744 | bx024448 | AT5G06900 | cytochrome P450, family 93, subfamily D, polypeptide 1, cytochrome P450, family 93, subfamily D, polypeptide 1 | -3.017 | -3.220 | -2.269 |
| 2 | 16920 | bx024670 |  |  | -2.732 | -1.968 | -2.256 |
| 2 | 17074 | bx024863 | AT1G08380 | photosystem I subunit O | -2.675 | -2.673 | -1.896 |
| 2 | 17107 | bx024906 | AT5G22390 | Protein of unknown function (DUF3049) | -1.880 | -2.426 | -1.308 |
| 2 | 17129 | bx024938 | AT2G16385 | unknown protein | -3.130 | -2.540 | -2.500 |
| 2 | 17143 | bx024956 | AT1G23740 | alkenal/one oxidoreductase, Oxidoreductase, zinc-binding dehydrogenase family protein | -1.521 | -2.361 | -1.075 |
| 2 | 17230 | bx025070 | AT3G19800 | Protein of unknown function (DUF177) | -1.912 | -3.195 | -2.151 |
| 2 | 17350 | bx025228 | AT2G18328 | RAD-like 4 | -2.115 | -2.589 | -2.073 |
| 2 | 17399 | bx025294 | AT4G13500 | unknown protein | -2.622 | -2.361 | -1.248 |
| 2 | 17527 | bx025481 | AT5G22390 | Protein of unknown function (DUF3049) | -1.738 | -2.346 | -0.894 |
| 2 | 17606 | bx025593 |  |  | -3.398 | -4.064 | -1.965 |
| 2 | 17635 | bx025629 | AT4G21200 | ARABIDOPSIS THALIANA GIBBERELLIN 2-OXIDASE 8, gibberellin 2-oxidase 8 | -2.430 | -3.064 | -1.045 |
| 2 | 17844 | bx025904 | AT2G36145 | unknown protein | -2.317 | -2.329 | -2.002 |
| 2 | 17899 | bx025972 | AT4G23990 | ARABIDOPSIS THALIANA CELLULOSE SYNTHASE-LIKE G3, cellulose synthase like G3 | -4.687 | -3.982 | -3.353 |
| 2 | 17919 | bx025998 | AT5G48020 | 2-oxoglutarate (2OG) and Fe(II)-dependent oxygenase superfamily protein | -3.397 | -5.076 | -3.421 |
| 2 | 17947 | bx026044 |  |  | -2.520 | -2.022 | -1.981 |
| 2 | 17952 | bx026049 |  |  | -3.068 | -2.481 | -2.301 |
| 2 | 18019 | bx026134 | AT1G68560 | thermoinhibition resistant germination 1, altered xyloglucan 3, alpha-xylosidase 1 | -1.948 | -2.509 | -1.173 |
| 2 | 18182 | bx026351 | AT5G16590 | Leucine rich repeat protein 1, Leucine-rich repeat protein kinase family protein | -3.852 | -4.296 | -4.017 |
| 2 | 18234 | bx026424 | AT1G73590 | ARABIDOPSIS THALIANA PIN-FORMED 1, Auxin efflux carrier family protein | -2.722 | -1.927 | -2.456 |
| 2 | 18277 | bx026479 | AT1G75280 | NmrA-like negative transcriptional regulator family protein | -3.411 | -3.871 | -2.649 |
| 2 | 18309 | bx026519 | AT5G37660 | plasmodesmata-located protein 7 | -2.139 | -2.935 | -1.962 |
| 2 | 18314 | bx026524 | AT1G11080 | serine carboxypeptidase-like 31 | -5.308 | -4.050 | -4.604 |
| 2 | 18322 | bx026535 | AT1G65870 | Disease resistance-responsive (dirigent-like protein) family protein | -2.984 | -3.468 | -2.343 |
| 2 | 18393 | bx026621 | AT5G05340 | Peroxidase superfamily protein | -4.577 | -3.934 | -3.795 |
| 2 | 18408 | bx026643 | AT1G20160 | Subtilisin-like serine endopeptidase family protein | -2.679 | -3.002 | -2.848 |
| 2 | 18453 | bx026710 | AT3G01990 | ACT domain repeat 6 | -1.978 | -3.100 | -2.233 |
| 2 | 18557 | bx026846 | AT5G57670 | Protein kinase superfamily protein | -3.131 | -3.282 | -2.618 |
| 2 | 18725 | bx027079 | AT5G03610 | GDSL-like Lipase/Acylhydrolase superfamily protein | -2.416 | -1.865 | -2.231 |
| 2 | 18784 | bx027155 |  |  | -3.764 | -2.817 | -3.293 |
| 2 | 18879 | bx027283 | AT1G11340 | S-locus lectin protein kinase family protein | -3.370 | -3.647 | -2.686 |
| 2 | 19098 | bx027579 | AT2G20940 | Protein of unknown function (DUF1279) | -2.397 | -3.087 | -2.349 |
| 2 | 19189 | bx027701 | AT2G33180 | unknown protein | -3.328 | -3.903 | -2.357 |
| 2 | 19286 | bx027839 | AT5G56840 | myb-like transcription factor family protein | -2.887 | -2.493 | -1.322 |
| 2 | 19368 | bx027948 | AT2G29660 | zinc finger (C2H2 type) family protein | -2.672 | -2.515 | -1.686 |
| 2 | 19404 | bx027997 | AT2G41120 | unknown protein | -1.826 | -2.949 | -1.159 |
| 2 | 19475 | bx028085 | AT1G11340 | S-locus lectin protein kinase family protein | -3.107 | -3.687 | -2.680 |
| 2 | 19544 | bx028175 |  |  | -2.417 | -1.817 | -1.406 |
| 2 | 19584 | bx028223 | AT5G44510 | target of AVRB operation1 | -2.436 | -1.387 | -1.739 |
| 2 | 19788 | bx028489 | AT5G67370 | Protein of unknown function (DUF1230) | -2.475 | -2.620 | -1.056 |
| 2 | 19815 | bx028525 | AT3G14067 | Subtilase family protein | -3.176 | -3.265 | -3.042 |
| 2 | 19914 | bx028656 |  |  | -3.015 | -2.941 | -2.947 |
| 2 | 19915 | bx028657 | AT3G21870 | cyclin p2;1 | -1.440 | -2.966 | -1.430 |
| 2 | 19924 | bx028674 | AT5G23850 | Arabidopsis thaliana protein of unknown function (DUF821) | -2.366 | -1.536 | -1.652 |
| 2 | 19971 | bx028737 | AT5G03250 | Phototropic-responsive NPH3 family protein | -3.060 | -2.709 | -1.863 |
| 2 | 20084 | bx028918 | AT3G59400 | GENOMES UNCOUPLED 4, enzyme binding;tetrapyrrole binding | -2.807 | -2.434 | -2.178 |
| 2 | 20118 | bx028968 | AT1G55850 | cellulose synthase like E1 | -2.718 | -2.360 | -1.072 |
| 2 | 20129 | bx028985 | AT3G17840 | receptor-like kinase 902 | -2.637 | -2.354 | -2.698 |
| 2 | 20133 | bx028995 | AT3G18670 | Ankyrin repeat family protein | -4.158 | -2.798 | -2.413 |
| 2 | 20147 | bx029010 |  |  | -3.764 | -4.781 | -1.563 |
| 2 | 20225 | bx029111 | AT5G47240 | nudix hydrolase homolog 8 | -2.817 | -2.373 | -2.523 |
| 2 | 20233 | bx029120 | AT1G04110 | STOMATAL DENSITY AND DISTRIBUTION, Subtilase family protein | -3.141 | -3.149 | -2.584 |
| 2 | 20302 | bx029202 |  |  | -1.585 | -2.410 | -1.880 |
| 2 | 20451 | bx029393 | AT5G66520 | Tetratricopeptide repeat (TPR)-like superfamily protein | -3.450 | -3.121 | -2.455 |
| 2 | 20456 | bx029401 | AT5G19740 | Peptidase M28 family protein | -2.498 | -2.466 | -1.172 |
| 2 | 20475 | bx029426 | AT3G09050 | unknown protein | -2.847 | -1.364 | -2.192 |
| 2 | 20513 | bx029477 |  |  | -2.394 | -1.970 | -2.668 |
| 2 | 20741 | bx029782 | AT3G22530 | unknown protein | -2.426 | -2.097 | -2.462 |
| 2 | 20833 | bx029904 | AT3G54390 | sequence-specific DNA binding transcription factors | -2.447 | -1.411 | -1.727 |
| 2 | 21110 | bx030287 | AT4G00820 | IQ-domain 17 | -2.419 | -2.059 | -2.113 |
| 2 | 21117 | bx030302 | AT1G26220 | Acyl-CoA N-acyltransferases (NAT) superfamily protein | -2.873 | -2.454 | -1.278 |
| 2 | 21371 | bx030644 | AT1G64500 | Glutaredoxin family protein | -1.444 | -2.806 | -1.331 |
| 2 | 21480 | bx030783 | AT1G11440 | unknown protein | -2.483 | -1.309 | -2.693 |
| 2 | 21495 | bx030810 | AT1G22380 | UDP-glucosyl transferase 85A3 | -2.116 | -2.348 | -1.653 |
| 2 | 21521 | bx030846 | AT3G08040 | MANGANESE ACCUMULATOR 1, FERRIC REDUCTASE DEFECTIVE 3, MATE efflux family protein | -3.274 | -3.427 | -3.428 |
| 2 | 21632 | bx030980 | AT1G26600 | CLAVATA3/ESR-RELATED 9 | -2.950 | -3.437 | -3.097 |
| 2 | 21640 | bx030991 | AT3G50440 | ARABIDOPSIS THALIANA METHYL ESTERASE 10, methyl esterase 10 | -2.350 | -1.984 | -2.651 |
| 2 | 21707 | bx031097 | AT1G15410 | aspartate-glutamate racemase family | -3.806 | -3.850 | -3.046 |
| 2 | 21723 | bx031115 | AT5G18940 | Mo25 family protein | -1.780 | -2.761 | -0.537 |
| 2 | 21747 | bx031151 | AT1G67980 | caffeoyl-CoA 3-O-methyltransferase | -2.575 | -1.724 | -1.544 |
| 2 | 21908 | bx031377 | AT2G22590 | UDP-Glycosyltransferase superfamily protein | -3.222 | -3.250 | -1.937 |
| 2 | 21922 | bx031395 | AT1G12330 | unknown protein | -2.229 | -2.431 | -2.526 |
| 2 | 22005 | bx031511 | AT4G22990 | Major Facilitator Superfamily with SPX (SYG1/Pho81/XPR1) domain-containing protein | -1.743 | -3.336 | -2.159 |
| 2 | 22210 | bx031806 | AT5G19500 | Tryptophan/tyrosine permease | -2.642 | -1.774 | -1.447 |
| 2 | 22265 | bx031883 | AT1G23980 | RING/U-box superfamily protein | -2.356 | -1.937 | -2.052 |
| 2 | 22280 | bx031904 | AT5G19740 | Peptidase M28 family protein | -2.518 | -2.513 | -1.184 |
| 2 | 22300 | bx031942 | AT1G54570 | Esterase/lipase/thioesterase family protein | -3.145 | -2.626 | -1.384 |
| 2 | 22665 | bx032451 | AT5G38510 | Rhomboid-related intramembrane serine protease family protein | -2.513 | -2.090 | -1.513 |
| 2 | 22781 | bx032628 | AT5G22860 | Serine carboxypeptidase S28 family protein | -3.137 | -3.643 | -2.491 |
| 2 | 22784 | bx032633 | AT1G65230 | Uncharacterized conserved protein (DUF2358) | -2.873 | -2.511 | -1.648 |
| 2 | 22831 | bx032698 | AT1G32900 | granule bound starch synthase 1, UDP-Glycosyltransferase superfamily protein | -2.830 | -2.327 | -2.852 |
| 2 | 23416 | bx033548 | AT5G17680 | disease resistance protein (TIR-NBS-LRR class), putative | -3.817 | -3.091 | -4.103 |
| 2 | 23616 | bx033824 | AT3G51300 | ARABIDOPSIS THALIANA RHO-RELATED PROTEIN FROM PLANTS 1, Arabidopsis RAC-like 11, RHO-related protein from plants 1 | -2.449 | -2.898 | -2.254 |
| 2 | 23845 | bx034152 | AT3G18670 | Ankyrin repeat family protein | -4.069 | -2.860 | -2.391 |
| 2 | 23854 | bx034163 | AT1G28100 | unknown protein | -2.341 | -2.252 | -1.168 |
| 2 | 23929 | bx034276 | ATMG00820 | Reverse transcriptase (RNA-dependent DNA polymerase) | -2.996 | -3.256 | -2.736 |
| 2 | 23946 | bx034301 | AT1G02850 | beta glucosidase 11 | -1.048 | -2.901 | -0.647 |
| 2 | 24064 | bx034470 | AT3G61230 | PLIM2c, GATA type zinc finger transcription factor family protein | -4.226 | -4.404 | -4.315 |
| 2 | 24131 | bx034571 | AT4G22200 | potassium transport 2/3 | -3.008 | -2.381 | -2.377 |
| 2 | 24303 | bx034811 | AT2G47860 | Phototropic-responsive NPH3 family protein | -3.398 | -3.129 | -3.070 |
| 2 | 24318 | bx034842 | AT4G33000 | SOS3-LIKE CALCIUM BINDING PROTEIN 8, calcineurin B-like protein 10 | -2.656 | -2.607 | -0.759 |
| 2 | 24465 | bx035067 | AT5G17170 | enhancer of sos3-1, rubredoxin family protein | -3.056 | -2.614 | -2.430 |
| 2 | 24495 | bx035111 | AT3G25500 | ARABIDOPSIS THALIANA FORMIN HOMOLOGY 1, formin homology 1 | -2.680 | -2.053 | -2.958 |
| 2 | 24534 | bx035163 | AT5G06210 | RNA binding (RRM/RBD/RNP motifs) family protein | -2.914 | -2.750 | -2.945 |
| 2 | 24633 | bx035302 | AT1G45474 | photosystem I light harvesting complex gene 5 | -2.846 | -2.311 | -1.284 |
| 2 | 24738 | bx035440 | AT1G74160 | unknown protein | -2.681 | -1.471 | -2.005 |
| 2 | 24801 | bx035532 | AT3G21670 | Major facilitator superfamily protein | -2.737 | -3.119 | -0.806 |
| 2 | 24841 | bx035599 | AT1G68520 | B-box type zinc finger protein with CCT domain | -2.998 | -2.114 | -1.755 |
| 2 | 24888 | bx035661 | AT5G47240 | nudix hydrolase homolog 8 | -2.815 | -2.368 | -2.570 |
| 2 | 24934 | bx035719 | AT5G13770 | Pentatricopeptide repeat (PPR-like) superfamily protein | -2.490 | -2.197 | -1.447 |
| 2 | 24974 | bx035777 | AT2G32150 | Haloacid dehalogenase-like hydrolase (HAD) superfamily protein | -2.533 | -2.988 | -2.349 |
| 2 | 25282 | bx036220 | AT3G05410 | Photosystem II reaction center PsbP family protein | -2.931 | -2.889 | -1.605 |
| 2 | 25293 | bx036235 | AT3G18670 | Ankyrin repeat family protein | -3.562 | -2.899 | -1.764 |
| 2 | 25550 | bx036629 | AT1G12370 | UV RESISTANCE 2, photolyase 1 | -2.628 | -2.341 | -1.014 |
| 2 | 25662 | bx036810 | AT5G41540 | Disease resistance protein (TIR-NBS-LRR class) family | -2.759 | -1.939 | -2.634 |
| 2 | 25682 | bx036838 | AT1G70760 | NADH dehydrogenase-like complex L, CHLORORESPIRATORY REDUCTION 23, inorganic carbon transport protein-related | -2.787 | -2.776 | -1.388 |
| 2 | 25734 | bx036921 | AT3G51300 | ARABIDOPSIS THALIANA RHO-RELATED PROTEIN FROM PLANTS 1, Arabidopsis RAC-like 11, RHO-related protein from plants 1 | -2.488 | -2.828 | -2.476 |
| 2 | 25744 | bx036938 | AT1G22070 | TGA1A-related gene 3 | -1.869 | -2.486 | -2.208 |
| 2 | 25750 | bx036944 | AT5G60210 | ROP interactive partner 5 | -2.480 | -2.157 | -1.701 |
| 2 | 26074 | bx037450 | AT4G13150 | unknown protein | -0.743 | -2.446 | -0.604 |
| 2 | 26105 | bx037514 | AT5G38510 | Rhomboid-related intramembrane serine protease family protein | -2.577 | -2.067 | -1.551 |
| 2 | 26148 | bx037600 | AT5G48460 | Actin binding Calponin homology (CH) domain-containing protein | -2.406 | -2.371 | -2.526 |
| 2 | 26181 | bx037645 | AT5G11090 | serine-rich protein-related | -1.508 | -2.315 | -1.356 |
| 2 | 26194 | bx037663 | AT3G05900 | neurofilament protein-related | -2.558 | -1.707 | -1.539 |
| 2 | 26206 | bx037679 | AT1G74110 | cytochrome P450, family 78, subfamily A, polypeptide 10, cytochrome P450, family 78, subfamily A, polypeptide 10 | -3.943 | -3.573 | -1.804 |
| 2 | 26243 | bx037727 | AT4G22890 | PGR5-LIKE A | -3.132 | -3.313 | -1.772 |
| 2 | 26248 | bx037734 | AT3G52740 | unknown protein | -1.823 | -2.708 | -1.738 |
| 2 | 26254 | bx037741 | AT3G49650 | P-loop containing nucleoside triphosphate hydrolases superfamily protein | -1.984 | -2.384 | -2.338 |
| 2 | 26323 | bx037839 | AT1G21090 | Cupredoxin superfamily protein | -2.834 | -1.661 | -2.627 |
| 2 | 26326 | bx037843 | AT3G09550 | Ankyrin repeat family protein | -4.359 | -4.282 | -2.956 |
| 2 | 26338 | bx037861 |  |  | -4.995 | -3.370 | -4.305 |
| 2 | 26561 | bx038159 | AT5G42905 | Polynucleotidyl transferase, ribonuclease H-like superfamily protein | -4.573 | -3.398 | -2.829 |
| 2 | 26652 | bx038281 | AT5G42905 | Polynucleotidyl transferase, ribonuclease H-like superfamily protein | -5.098 | -4.158 | -2.619 |
| 2 | 26673 | bx038308 | AT5G62360 | Plant invertase/pectin methylesterase inhibitor superfamily protein | -3.450 | -2.738 | -1.838 |
| 2 | 26884 | bx038586 | AT5G12900 | unknown protein | -2.634 | -2.346 | -2.476 |
| 2 | 26916 | bx038632 |  |  | -1.917 | -3.093 | -2.089 |
| 2 | 27090 | bx038855 | AT5G19740 | Peptidase M28 family protein | -2.526 | -2.509 | -1.191 |
| 2 | 27158 | bx038944 | AT5G13170 | senescence-associated gene 29 | -2.055 | -2.696 | -0.846 |
| 2 | 27171 | bx038962 | AT5G42560 | Abscisic acid-responsive (TB2/DP1, HVA22) family protein | -2.362 | -1.837 | -2.044 |
| 2 | 27214 | bx039019 |  |  | -2.742 | -2.567 | -1.645 |
| 2 | 27280 | bx039111 | AT4G22190 | unknown protein | -2.730 | -3.991 | -3.306 |
| 2 | 27313 | bx039156 | ATMG00310 | RNA-directed DNA polymerase (reverse transcriptase)-related family protein | -3.673 | -3.824 | -2.590 |
| 2 | 27389 | bx039248 | AT2G41120 | unknown protein | -1.602 | -2.574 | -1.049 |
| 2 | 27471 | bx039355 | AT1G19250 | flavin-dependent monooxygenase 1 | -2.501 | -2.710 | -1.798 |
| 2 | 27549 | bx039453 | AT3G58760 | Integrin-linked protein kinase family | -2.362 | -3.513 | -2.407 |
| 2 | 27565 | bx039473 |  |  | -3.320 | -3.320 | -3.120 |
| 2 | 27577 | bx039489 |  |  | -3.245 | -2.538 | -1.861 |
| 2 | 27605 | bx039526 | AT3G56290 | unknown protein | -1.544 | -2.458 | -1.548 |
| 2 | 27674 | bx039619 | AT5G63270 | RPM1-interacting protein 4 (RIN4) family protein | -1.112 | -2.380 | -0.844 |
| 2 | 27714 | bx039672 | AT4G23160 | cysteine-rich RLK (RECEPTOR-like protein kinase) 8 | -2.944 | -4.307 | -2.911 |
| 2 | 27756 | bx039725 | AT4G33280 | AP2/B3-like transcriptional factor family protein | -2.405 | -1.275 | -1.660 |
| 2 | 27841 | bx039840 | AT5G06940 | Leucine-rich repeat receptor-like protein kinase family protein | -2.541 | -1.787 | -2.768 |
| 2 | 27857 | bx039862 | AT2G24280 | alpha/beta-Hydrolases superfamily protein | -1.529 | -2.751 | -1.607 |
| 2 | 27891 | bx039906 | AT3G14470 | NB-ARC domain-containing disease resistance protein | -2.909 | -2.785 | -3.070 |
| 2 | 27983 | bx040024 | AT4G23990 | ARABIDOPSIS THALIANA CELLULOSE SYNTHASE-LIKE G3, cellulose synthase like G3 | -4.713 | -3.828 | -3.259 |
| 2 | 28023 | bx040074 | AT2G13360 | L-serine:glyoxylate aminotransferase, ALANINE:GLYOXYLATE AMINOTRANSFERASE 1, alanine:glyoxylate aminotransferase | -2.727 | -2.369 | -1.152 |
| 2 | 28029 | bx040082 | AT4G18130 | phytochrome E | -2.323 | -1.591 | -1.323 |
| 2 | 28046 | bx040106 | AT5G62360 | Plant invertase/pectin methylesterase inhibitor superfamily protein | -1.305 | -2.306 | -1.235 |
| 2 | 28102 | bx040175 |  |  | -1.570 | -2.321 | -1.536 |
| 2 | 28149 | bx040238 | AT5G64620 | cell wall / vacuolar inhibitor of fructosidase 2 | -2.962 | -3.349 | -2.490 |
| 2 | 28210 | bx040312 |  |  | -1.505 | -2.456 | -1.596 |
| 2 | 28260 | bx040376 | AT3G57990 | unknown protein | -1.984 | -2.312 | -1.542 |
| 2 | 28290 | bx040417 | AT2G45580 | cytochrome P450, family 76, subfamily C, polypeptide 3, cytochrome P450, family 76, subfamily C, polypeptide 3 | -2.123 | -2.763 | -1.621 |
| 2 | 28363 | bx040503 | AT3G50950 | HOPZ-ACTIVATED RESISTANCE 1 | -2.357 | -1.442 | -1.441 |
| 2 | 28400 | bx040555 | AT2G17820 | histidine kinase 1 | -2.982 | -2.646 | -1.808 |
| 2 | 28430 | bx040592 | AT2G45220 | Plant invertase/pectin methylesterase inhibitor superfamily | -1.608 | -4.224 | -0.627 |
| 2 | 28491 | bx040669 | AT3G61230 | PLIM2c, GATA type zinc finger transcription factor family protein | -4.096 | -4.443 | -4.669 |
| 2 | 28530 | bx040721 |  |  | -4.208 | -3.076 | -3.003 |
| 2 | 28615 | bx040828 | AT1G29440 | SAUR-like auxin-responsive protein family | -2.417 | -1.357 | -2.002 |
| 2 | 28644 | bx040861 | AT1G47670 | Transmembrane amino acid transporter family protein | -2.344 | -2.296 | -1.762 |
| 2 | 28678 | bx040904 | AT2G32300 | uclacyanin 1 | -2.697 | -3.484 | -2.413 |
| 2 | 28730 | bx040974 |  |  | -4.009 | -5.070 | -1.517 |
| 2 | 28766 | bx041022 | AT5G52420 | unknown protein | -2.824 | -2.398 | -1.368 |
| 2 | 28779 | bx041037 | AT5G26780 | serine hydroxymethyltransferase 2 | -1.451 | -2.555 | -1.244 |
| 2 | 28965 | bx041273 | AT1G71970 | unknown protein | -3.274 | -1.884 | -2.653 |
| 2 | 29069 | bx041403 | AT4G39210 | Glucose-1-phosphate adenylyltransferase family protein | -2.404 | -2.512 | -2.332 |
| 2 | 29141 | bx041494 | AT1G56210 | Heavy metal transport/detoxification superfamily protein | -2.553 | -1.577 | -2.498 |
| 2 | 29162 | bx041525 |  |  | -2.743 | -1.971 | -2.275 |
| 2 | 29169 | bx041532 | AT1G44000 | unknown protein | -1.643 | -2.499 | -1.655 |
| 2 | 29339 | bx041745 |  |  | -2.233 | -1.411 | -2.316 |
| 2 | 29356 | bx041770 | AT5G65730 | xyloglucan endotransglucosylase/hydrolase 6 | -2.371 | -1.607 | -2.312 |
| 2 | 29431 | bx041864 | AT1G14720 | xyloglucan endotransglycosylase related 2, ENDOXYLOGLUCAN TRANSFERASE A2, xyloglucan endotransglucosylase/hydrolase 28 | -3.012 | -2.102 | -2.329 |
| 2 | 29498 | bx041954 | AT4G14010 | ralf-like 32 | -2.854 | -2.479 | -1.195 |
| 2 | 29517 | bx041979 | AT5G37660 | plasmodesmata-located protein 7 | -2.733 | -3.646 | -2.074 |
| 2 | 29533 | bx042001 |  |  | -3.466 | -3.081 | -2.618 |
| 2 | 29544 | bx042012 | AT5G27690 | Heavy metal transport/detoxification superfamily protein | -3.103 | -2.588 | -1.986 |
| 2 | 29630 | bx042121 | AT1G74160 | unknown protein | -2.535 | -1.864 | -2.015 |
| 2 | 29681 | bx042191 | AT4G23800 | 3xHigh Mobility Group-box2, HMG (high mobility group) box protein | -1.532 | -2.385 | -1.626 |
| 2 | 29693 | bx042204 | AT1G21340 | Dof-type zinc finger DNA-binding family protein | -3.129 | -3.978 | -3.237 |
| 2 | 29724 | bx042241 | AT2G03090 | EXPANSIN 15, expansin A15 | -5.673 | -5.584 | -5.475 |
| 2 | 29749 | bx042271 | AT3G18670 | Ankyrin repeat family protein | -2.830 | -1.805 | -2.591 |
| 2 | 29826 | bx042365 |  |  | -3.318 | -2.637 | -1.788 |
| 2 | 29865 | bx042411 | AT5G66330 | Leucine-rich repeat (LRR) family protein | -2.974 | -2.992 | -3.365 |
| 2 | 29954 | bx042521 | AT4G21445 | unknown protein | -2.829 | -2.310 | -1.141 |
| 2 | 30081 | bx042669 | AT4G21960 | Peroxidase superfamily protein | -3.356 | -2.595 | -2.376 |
| 2 | 30185 | bx042814 |  |  | -3.300 | -2.879 | -3.164 |
| 2 | 30189 | bx042818 | AT5G54530 | Protein of unknown function, DUF538 | -2.785 | -2.039 | -2.177 |
| 2 | 30198 | bx042832 | AT3G16330 | unknown protein | -4.396 | -3.713 | -1.918 |
| 2 | 30253 | bx042897 | AT4G32870 | Polyketide cyclase/dehydrase and lipid transport superfamily protein | -3.155 | -2.268 | -1.554 |
| 2 | 30316 | bx042983 | AT5G50710 | unknown protein | -4.164 | -3.118 | -3.966 |
| 2 | 30362 | bx043046 | AT4G39870 | TLD-domain containing nucleolar protein | -2.659 | -2.524 | -2.372 |
| 2 | 30369 | bx043054 | AT1G10540 | nucleobase-ascorbate transporter 8 | -2.448 | -1.962 | -1.667 |
| 2 | 30492 | bx043212 | AT5G67370 | Protein of unknown function (DUF1230) | -2.424 | -2.643 | -1.088 |
| 2 | 30500 | bx043221 |  |  | -2.412 | -1.955 | -2.392 |
| 2 | 30509 | bx043230 | AT1G12900 | glyceraldehyde 3-phosphate dehydrogenase A subunit 2 | -2.184 | -2.495 | -1.400 |
| 2 | 30534 | bx043259 |  |  | -2.756 | -1.523 | -2.263 |
| 2 | 30551 | bx043282 | AT1G60470 | galactinol synthase 4 | -1.831 | -2.646 | -1.829 |
| 2 | 30619 | bx043377 | AT4G13500 | unknown protein | -2.351 | -2.317 | -1.809 |
| 2 | 30644 | bx043414 |  |  | -2.882 | -1.631 | -3.138 |
| 2 | 30787 | bx043591 | AT4G37630 | cyclin d5;1 | -1.397 | -2.488 | -0.956 |
| 2 | 30829 | bx043642 | AT1G15550 | GA REQUIRING 4, ARABIDOPSIS THALIANA GIBBERELLIN 3 BETA-HYDROXYLASE 1, gibberellin 3-oxidase 1 | -2.345 | -2.430 | -2.213 |
| 2 | 30869 | bx043698 | AT4G22730 | Leucine-rich repeat protein kinase family protein | -2.918 | -2.155 | -1.990 |
| 2 | 30896 | bx043734 | AT4G24000 | ARABIDOPSIS THALIANA CELLULOSE SYNTHASE LIKE G2, cellulose synthase like G2 | -3.227 | -2.434 | -1.823 |
| 2 | 30905 | bx043749 |  |  | -2.682 | -2.101 | -2.303 |
| 2 | 30906 | bx043750 | AT4G23340 | 2-oxoglutarate (2OG) and Fe(II)-dependent oxygenase superfamily protein | -3.339 | -3.202 | -3.690 |
| 2 | 30974 | bx043837 |  |  | -3.104 | -3.429 | -1.950 |
| 2 | 31083 | bx043971 | AT5G51550 | EXORDIUM like 3 | -4.051 | -2.526 | -3.531 |
| 2 | 31114 | bx044002 | AT4G11960 | PGR5-like B | -2.938 | -3.198 | -1.923 |
| 2 | 31278 | bx044226 |  |  | -2.051 | -2.748 | -1.233 |
| 2 | 31302 | bx044260 | AT1G64150 | Uncharacterized protein family (UPF0016) | -2.442 | -2.676 | -1.543 |
| 2 | 31343 | bx044311 | AT5G37660 | plasmodesmata-located protein 7 | -2.157 | -2.960 | -1.999 |
| 2 | 31376 | bx044351 | AT1G79850 | PLASTID RIBOSOMAL SMALL SUBUNIT PROTEIN 17, PIGMENT DEFECTIVE 347, ribosomal protein S17 | -3.108 | -2.912 | -1.851 |
| 2 | 31402 | bx044388 | AT5G62170 | unknown protein | -3.380 | -4.729 | -4.059 |
| 2 | 31404 | bx044390 | AT5G44680 | DNA glycosylase superfamily protein | -2.053 | -2.187 | -2.364 |
| 2 | 31555 | bx044573 | AT1G11720 | starch synthase 3 | -2.392 | -2.279 | -0.776 |
| 2 | 31772 | bx044841 | AT2G40230 | HXXXD-type acyl-transferase family protein | -3.917 | -2.222 | -3.487 |
| 2 | 31819 | bx044902 | AT2G23300 | Leucine-rich repeat protein kinase family protein | -2.827 | -2.221 | -2.912 |
| 2 | 31859 | bx044951 | AT5G25280 | serine-rich protein-related | -2.478 | -1.719 | -1.764 |
| 2 | 32289 | bx045501 | AT1G45474 | photosystem I light harvesting complex gene 5 | -2.490 | -2.375 | -1.516 |
| 2 | 32313 | bx045531 | AT1G20696 | high mobility group B3 | -3.153 | -3.327 | -3.119 |
| 2 | 32384 | bx045612 | AT3G55240 | Plant protein 1589 of unknown function | -2.838 | -3.670 | -2.004 |
| 2 | 32544 | bx045811 |  |  | -2.162 | -2.972 | -1.912 |
| 2 | 32579 | bx045860 |  |  | -2.479 | -1.863 | -1.784 |
| 2 | 32640 | bx045926 |  |  | -2.429 | -2.080 | -2.735 |
| 2 | 32749 | bx046063 | AT1G18170 | FKBP-like peptidyl-prolyl cis-trans isomerase family protein | -2.448 | -2.781 | -1.681 |
| 2 | 32773 | bx046097 | AT4G32140 | EamA-like transporter family | -1.296 | -2.370 | -1.085 |
| 2 | 32874 | bx046222 | AT5G58140 | NON PHOTOTROPIC HYPOCOTYL 1-LIKE, phototropin 2 | -2.348 | -1.987 | -0.910 |
| 2 | 32901 | bx046258 | AT1G60790 | TRICHOME BIREFRINGENCE-LIKE 2, Plant protein of unknown function (DUF828) | -2.332 | -1.736 | -1.536 |
| 2 | 33041 | bx046429 |  |  | -2.284 | -1.903 | -2.556 |
| 2 | 33083 | bx046483 | AT3G51670 | SEC14 cytosolic factor family protein / phosphoglyceride transfer family protein | -2.594 | -2.376 | -2.471 |
| 2 | 33178 | bx046607 | AT1G23090 | sulfate transporter 91 | -3.178 | -2.664 | -2.351 |
| 2 | 33301 | bx046769 | AT5G42860 | unknown protein | -2.046 | -2.732 | -1.299 |
| 2 | 33373 | bx046857 | AT1G09340 | heteroglycan-interacting protein 1.3, CHLOROPLAST STEM-LOOP BINDING PROTEIN OF 41 KDA, chloroplast RNA binding | -1.595 | -2.524 | -1.771 |
| 2 | 33605 | bx047142 |  |  | -1.946 | -2.315 | -1.905 |
| 2 | 33931 | bx047542 | AT3G13960 | growth-regulating factor 5 | -2.250 | -3.170 | -2.703 |
| 2 | 33949 | bx047562 |  |  | -2.822 | -3.242 | -2.493 |
| 2 | 34026 | bx047672 | AT2G44940 | Integrase-type DNA-binding superfamily protein | -3.570 | -3.483 | -1.365 |
| 2 | 34109 | bx047776 | AT4G36360 | beta-galactosidase 3 | -2.333 | -2.110 | -1.759 |
| 2 | 34121 | bx047789 | AT1G19530 | unknown protein | -3.766 | -3.512 | -4.139 |
| 2 | 34143 | bx047817 |  |  | -1.587 | -2.875 | -1.419 |
| 2 | 34285 | bx047995 | AT2G36985 | ROTUNDIFOLIA4, DEVIL 16, DVL family protein | -4.153 | -2.241 | -4.020 |
| 2 | 34293 | bx048005 | AT1G68040 | S-adenosyl-L-methionine-dependent methyltransferases superfamily protein | -2.762 | -2.819 | -1.114 |
| 2 | 34314 | bx048029 | AT4G24150 | growth-regulating factor 8 | -3.646 | -3.188 | -3.848 |
| 2 | 34329 | bx048048 |  |  | -2.385 | -2.621 | -1.866 |
| 2 | 34375 | bx048099 | AT1G72670 | IQ-domain 8 | -1.457 | -2.412 | -1.441 |
| 2 | 34385 | bx048110 | AT4G10340 | light harvesting complex of photosystem II 5 | -1.926 | -2.578 | -1.555 |
| 2 | 34441 | bx048191 |  |  | -2.355 | -1.846 | -1.239 |
| 2 | 34631 | bx048417 | AT5G45740 | Ubiquitin domain-containing protein | -2.544 | -2.407 | -1.474 |
| 2 | 34639 | bx048427 | AT5G50740 | Heavy metal transport/detoxification superfamily protein | -2.382 | -2.100 | -2.139 |
| 2 | 34704 | bx048506 | AT1G27600 | IRREGULAR XYLEM 9-LIKE, IRREGULAR XYLEM 9 Homolog, Nucleotide-diphospho-sugar transferases superfamily protein | -3.197 | -2.604 | -2.584 |
| 2 | 34705 | bx048507 |  |  | -2.943 | -2.149 | -1.649 |
| 2 | 34743 | bx048549 | AT3G17040 | high chlorophyll fluorescent 107 | -2.425 | -1.380 | -1.492 |
| 2 | 34796 | bx048614 | AT5G51660 | cleavage and polyadenylation specificity factor 160 | -2.573 | -2.667 | -1.647 |
| 2 | 34803 | bx048622 | AT5G09820 | Plastid-lipid associated protein PAP / fibrillin family protein | -2.654 | -1.951 | -1.293 |
| 2 | 34806 | bx048625 | ATMG00820 | Reverse transcriptase (RNA-dependent DNA polymerase) | -3.003 | -3.271 | -2.702 |
| 2 | 34842 | bx048680 | AT1G09340 | heteroglycan-interacting protein 1.3, CHLOROPLAST STEM-LOOP BINDING PROTEIN OF 41 KDA, chloroplast RNA binding | -1.994 | -2.357 | -1.509 |
| 2 | 34885 | bx048733 | AT3G12120 | fatty acid desaturase 2 | -2.197 | -2.440 | -2.065 |
| 2 | 34990 | bx048865 | AT5G51550 | EXORDIUM like 3 | -3.267 | -2.169 | -2.361 |
| 2 | 35005 | bx048882 | AT4G28250 | expansin B3 | -2.616 | -2.415 | -2.504 |
| 2 | 35063 | bx048958 | AT1G74940 | Protein of unknown function (DUF581) | -2.568 | -1.775 | -1.333 |
| 2 | 35148 | bx049068 | AT2G47240 | LONG-CHAIN ACYL-COA SYNTHASE 1, ECERIFERUM 8, AMP-dependent synthetase and ligase family protein | -2.581 | -2.271 | -1.854 |
| 2 | 35233 | bx049167 | AT3G21870 | cyclin p2;1 | -1.578 | -2.895 | -1.411 |
| 2 | 35237 | bx049173 |  |  | -3.287 | -2.541 | -3.420 |
| 2 | 35252 | bx049199 |  |  | -3.328 | -1.952 | -2.043 |
| 2 | 35283 | bx049244 |  |  | -3.222 | -2.764 | -2.716 |
| 2 | 35439 | bx049437 |  |  | -2.620 | -2.655 | -2.941 |
| 2 | 35577 | bx049627 | AT4G37080 | Protein of unknown function, DUF547 | -3.095 | -3.124 | -3.551 |
| 2 | 35579 | bx049629 | AT1G64150 | Uncharacterized protein family (UPF0016) | -2.624 | -3.327 | -1.965 |
| 2 | 35656 | bx049739 | AT2G38010 | Neutral/alkaline non-lysosomal ceramidase | -2.645 | -2.666 | -2.461 |
| 2 | 35703 | bx049802 | AT3G01440 | PsbQ-like 2, Photosynthetic NDH subcomplex L 3, PsbQ-like 1 | -4.140 | -3.507 | -1.808 |
| 2 | 35715 | bx049817 | AT5G16000 | NSP-interacting kinase 1 | -2.284 | -1.938 | -2.323 |
| 2 | 35841 | bx049992 |  |  | -2.448 | -1.806 | -1.675 |
| 2 | 35887 | bx050048 | AT3G52290 | IQ-domain 3 | -2.367 | -2.653 | -1.995 |
| 2 | 35889 | bx050050 | AT1G21090 | Cupredoxin superfamily protein | -3.392 | -1.853 | -3.370 |
| 2 | 36058 | bx050261 |  |  | -2.437 | -2.716 | -1.983 |
| 2 | 36779 | bx051144 | AT4G02530 | chloroplast thylakoid lumen protein | -2.379 | -2.179 | -1.790 |
| 2 | 36821 | bx051196 |  |  | -3.111 | -2.868 | -2.094 |
| 2 | 36917 | bx051326 | AT2G47010 | unknown protein | -2.750 | -3.000 | -2.969 |
| 2 | 37004 | bx051443 | AT5G42760 | Leucine carboxyl methyltransferase | -2.566 | -2.608 | -2.323 |
| 2 | 37009 | bx051449 | AT2G39730 | rubisco activase | -1.130 | -2.796 | -1.356 |
| 2 | 37189 | bx051685 | AT1G23740 | alkenal/one oxidoreductase, Oxidoreductase, zinc-binding dehydrogenase family protein | -1.371 | -2.467 | -1.456 |
| 2 | 37314 | bx051833 | AT2G32500 | Stress responsive alpha-beta barrel domain protein | -1.327 | -3.758 | -1.769 |
| 2 | 37325 | bx051847 |  |  | -0.836 | -2.486 | -1.159 |
| 2 | 37372 | bx051906 | AT1G09540 | ARABIDOPSIS THALIANA MYB DOMAIN PROTEIN 61, myb domain protein 61 | -2.810 | -2.572 | -2.741 |
| 2 | 37522 | bx052092 | AT4G03280 | PROTON GRADIENT REGULATION 1, photosynthetic electron transfer C | -3.056 | -2.178 | -1.508 |
| 2 | 37721 | bx052344 | AT1G74880 | NADH dehydrogenase-like complex ), NAD(P)H:plastoquinone dehydrogenase complex subunit O | -1.665 | -2.648 | -1.696 |
| 2 | 37747 | bx052374 | AT1G75100 | J-domain protein required for chloroplast accumulation response 1 | -2.973 | -2.334 | -1.473 |
| 2 | 38045 | bx052782 | AT1G11410 | S-locus lectin protein kinase family protein | -2.950 | -3.632 | -2.669 |
| 2 | 38061 | bx052801 | AT1G76990 | ACT domain repeat 3 | -2.618 | -2.257 | -2.479 |
| 2 | 38204 | bx052976 | AT1G69040 | ACT domain repeat 4 | -2.095 | -3.013 | -1.683 |
| 2 | 38220 | bx052997 | AT3G19270 | cytochrome P450, family 707, subfamily A, polypeptide 4, cytochrome P450, family 707, subfamily A, polypeptide 4 | -4.430 | -2.846 | -2.467 |
| 2 | 38226 | bx053004 |  |  | -3.642 | -3.944 | -3.757 |
| 2 | 38267 | bx053058 | AT3G07040 | RESISTANCE TO PSEUDOMONAS SYRINGAE 3, RESISTANCE TO P. SYRINGAE PV MACULICOLA 1, NB-ARC domain-containing disease resistance protein | -2.813 | -1.943 | -2.115 |
| 2 | 38599 | bx053493 | AT3G06150 | unknown protein | -2.318 | -2.906 | -1.996 |
| 2 | 38789 | bx053737 | AT5G06210 | RNA binding (RRM/RBD/RNP motifs) family protein | -2.985 | -2.615 | -2.327 |
| 2 | 38810 | bx053761 | AT1G63870 | Disease resistance protein (TIR-NBS-LRR class) family | -2.999 | -2.535 | -2.615 |
| 2 | 38814 | bx053765 | AT1G66780 | MATE efflux family protein | -2.044 | -2.405 | -0.907 |
| 2 | 38833 | bx053794 | AT4G37250 | Leucine-rich repeat protein kinase family protein | -2.817 | -2.104 | -2.678 |
| 2 | 38859 | bx053831 | AT1G07570 | Protein kinase superfamily protein | -2.441 | -2.559 | -1.783 |
| 2 | 39009 | bx054019 |  |  | -2.653 | -2.244 | -2.137 |
| 2 | 39086 | bx054121 |  |  | -1.758 | -2.682 | -1.857 |
| 2 | 39092 | bx054129 |  |  | -2.625 | -3.452 | -2.452 |
| 2 | 39129 | bx054182 | AT5G19740 | Peptidase M28 family protein | -2.510 | -2.493 | -1.208 |
| 2 | 39181 | bx054260 |  |  | -1.637 | -2.370 | -1.761 |
| 2 | 39269 | bx054390 | AT3G21510 | histidine-containing phosphotransmitter 1 | -2.418 | -3.318 | -2.260 |
| 2 | 39288 | bx054420 | AT1G80340 | ARABIDOPSIS THALIANA GIBBERELLIN-3-OXIDASE 2, gibberellin 3-oxidase 2 | -3.957 | -2.699 | -3.172 |
| 2 | 39395 | bx054566 | AT5G40270 | HD domain-containing metal-dependent phosphohydrolase family protein | -3.210 | -2.435 | -1.672 |
| 2 | 39520 | bx054736 |  |  | -3.540 | -3.076 | -2.538 |
| 2 | 39551 | bx054779 |  |  | -2.322 | -2.179 | -1.643 |
| 2 | 39682 | bx054980 |  |  | -2.465 | -2.877 | -2.609 |
| 2 | 39926 | bx055333 | AT2G33400 | unknown protein | -1.405 | -2.646 | -1.641 |
| 2 | 39948 | bx055362 | AT3G44940 | Protein of unknown function (DUF1635) | -3.149 | -2.797 | -3.057 |
| 2 | 39965 | bx055386 | AT1G77145 | Protein of unknown function (DUF506) | -1.236 | -3.492 | 0.150 |
| 2 | 40026 | bx055457 | AT2G14080 | Disease resistance protein (TIR-NBS-LRR class) family | -1.989 | -2.894 | -1.255 |
| 2 | 40029 | bx055460 | AT1G77210 | sugar transport protein 14, sugar transporter 14 | -3.039 | -3.230 | -2.811 |
| 2 | 40066 | bx055517 | AT5G10250 | DEFECTIVELY ORGANIZED TRIBUTARIES 3, Phototropic-responsive NPH3 family protein | -2.973 | -2.774 | -2.008 |
| 2 | 40080 | bx055538 |  |  | -0.360 | -2.759 | -0.384 |
| 2 | 40159 | bx055629 | AT2G35880 | TPX2 (targeting protein for Xklp2) protein family | -1.505 | -2.620 | -1.596 |
| 2 | 40391 | bx055911 | AT4G15920 | Nodulin MtN3 family protein | -2.488 | -1.720 | -1.347 |
| 2 | 40432 | bx055960 | AT1G72430 | SAUR-like auxin-responsive protein family | -2.652 | -3.371 | -3.054 |
| 2 | 40497 | bx056042 | AT5G05330 | HMG-box (high mobility group) DNA-binding family protein | -2.788 | -2.282 | -1.684 |
| 2 | 40529 | bx056079 | AT5G02040 | prenylated RAB acceptor 1.A1 | -1.658 | -3.078 | -1.525 |
| 2 | 40531 | bx056081 | AT2G38010 | Neutral/alkaline non-lysosomal ceramidase | -4.439 | -4.884 | -3.259 |
| 2 | 40545 | bx056103 | AT5G52100 | chlororespiration reduction 1, Dihydrodipicolinate reductase, bacterial/plant | -2.654 | -3.221 | -2.004 |
| 2 | 40600 | bx056171 |  |  | -2.331 | -2.194 | -1.880 |
| 2 | 40673 | bx056265 |  |  | -2.722 | -1.806 | -1.438 |
| 2 | 40731 | bx056332 |  |  | -2.311 | -1.723 | -1.303 |
| 2 | 40811 | bx056431 |  |  | -2.852 | -1.818 | -1.583 |
| 2 | 40850 | bx056476 | AT5G52780 | Protein of unknown function (DUF3464) | -2.744 | -1.544 | -1.828 |
| 2 | 40895 | bx056527 | AT1G06520 | glycerol-3-phosphate acyltransferase 1 | -2.815 | -3.469 | -1.679 |
| 2 | 40994 | bx056654 | AT4G21200 | ARABIDOPSIS THALIANA GIBBERELLIN 2-OXIDASE 8, gibberellin 2-oxidase 8 | -2.392 | -3.077 | -1.056 |
| 2 | 41004 | bx056669 | AT1G11120 | unknown protein | -2.498 | -1.336 | -2.001 |
| 2 | 41047 | bx056721 |  |  | -1.761 | -2.579 | -0.703 |
| 2 | 41170 | bx056863 | AT5G19520 | mechanosensitive channel of small conductance-like 9 | -2.351 | -1.828 | -2.418 |
| 2 | 41284 | bx057010 | AT4G39870 | TLD-domain containing nucleolar protein | -3.539 | -3.433 | -2.962 |
| 2 | 41332 | bx057065 |  |  | -1.733 | -2.327 | -1.798 |
| 2 | 41523 | bx057301 |  |  | -3.188 | -2.006 | -2.557 |
| 2 | 41578 | bx057373 | AT2G28580 | Plant protein of unknown function (DUF247) | -3.473 | -3.997 | -2.965 |
| 2 | 41580 | bx057375 | AT1G03220 | Eukaryotic aspartyl protease family protein | -2.056 | -2.585 | -1.986 |
| 2 | 41582 | bx057377 |  |  | -2.740 | -2.408 | -2.518 |
| 2 | 41684 | bx057496 |  |  | -3.808 | -2.225 | -3.388 |
| 2 | 41751 | bx057576 | AT1G64185 | Lactoylglutathione lyase / glyoxalase I family protein | -2.097 | -2.526 | -1.596 |
| 2 | 41869 | bx057725 |  |  | -1.609 | -2.313 | -1.412 |
| 2 | 41993 | bx057865 |  |  | -2.483 | -2.902 | -1.183 |
| 2 | 41995 | bx057867 |  |  | -2.453 | -2.461 | -2.291 |
| 2 | 42063 | bx057942 | AT5G42650 | DELAYED DEHISCENCE 2, CYTOCHROME P450 74A, allene oxide synthase | -2.991 | -1.935 | -2.322 |
| 2 | 42170 | bx058075 | AT4G39350 | cellulose synthase A2 | -2.605 | -2.404 | -2.160 |
| 2 | 42358 | bx058305 |  |  | -1.684 | -2.406 | -1.869 |
| 2 | 42432 | bx058393 | AT1G74880 | NADH dehydrogenase-like complex ), NAD(P)H:plastoquinone dehydrogenase complex subunit O | -1.682 | -2.653 | -1.697 |
| 2 | 42630 | bx058639 | AT1G06330 | Heavy metal transport/detoxification superfamily protein | -2.603 | -1.268 | -2.438 |
| 2 | 42649 | bx058660 |  |  | -2.810 | -1.985 | -2.458 |
| 2 | 42687 | bx058712 | AT2G25735 | unknown protein | -3.537 | -2.996 | -3.500 |
| 2 | 43362 | bx059547 | AT3G52870 | IQ calmodulin-binding motif family protein | -1.954 | -2.306 | -1.910 |
| 2 | 43456 | bx059668 | AT3G18670 | Ankyrin repeat family protein | -3.994 | -2.945 | -2.374 |
| 2 | 43493 | bx059713 |  |  | -2.083 | -2.241 | -2.338 |
| 2 | 43609 | bx059862 | AT4G21445 | unknown protein | -2.576 | -2.774 | -0.969 |
| 2 | 44031 | bx060381 | AT4G26620 | Sucrase/ferredoxin-like family protein | -2.516 | -2.544 | -2.688 |
| 2 | 44129 | bx060491 |  |  | -1.884 | -2.671 | -1.125 |
| 2 | 44186 | bx060557 |  |  | -2.778 | -2.302 | -2.474 |
| 2 | 44190 | bx060561 | AT5G57480 | P-loop containing nucleoside triphosphate hydrolases superfamily protein | -3.022 | -2.621 | -2.052 |
| 2 | 44269 | bx060657 |  |  | -2.391 | -2.781 | -2.585 |
| 2 | 44574 | bx061035 |  |  | -2.790 | -2.404 | -3.118 |
| 2 | 44696 | bx061180 | AT1G21500 | unknown protein | -3.276 | -3.592 | -3.332 |
| 2 | 44740 | bx061230 | AT5G06470 | Glutaredoxin family protein | -1.450 | -2.640 | -1.772 |
| 2 | 44775 | bx061274 |  |  | -2.602 | -2.405 | -2.027 |
| 2 | 44782 | bx061285 |  |  | -2.395 | -1.572 | -1.351 |
| 2 | 44813 | bx061323 |  |  | -3.052 | -3.342 | -2.712 |
| 2 | 44882 | bx061412 | AT4G25040 | Uncharacterised protein family (UPF0497) | -2.145 | -2.418 | -1.575 |
| 2 | 44957 | bx061504 |  |  | -2.278 | -1.626 | -2.424 |
| 2 | 44991 | bx061545 |  |  | -2.857 | -2.679 | -2.982 |
| 2 | 45053 | bx061625 | AT5G64740 | PROCUSTE 1, ISOXABEN RESISTANT 2, cellulose synthase 6 | -2.351 | -2.541 | -2.210 |
| 2 | 45154 | bx061753 | AT1G58420 | Uncharacterised conserved protein UCP031279 | -2.351 | -1.271 | -1.590 |
| 2 | 45194 | bx061801 |  |  | -3.159 | -2.848 | -3.038 |
| 2 | 45298 | bx061927 | AT4G09010 | thylakoid lumen 29, ascorbate peroxidase 4 | -2.085 | -2.617 | -1.993 |
| 2 | 45376 | bx062020 |  |  | -3.260 | -2.240 | -2.291 |
| 2 | 45410 | bx062058 |  |  | -2.491 | -3.414 | -2.896 |
| 2 | 45845 | bx062620 |  |  | -1.586 | -2.468 | -1.592 |
| 2 | 45879 | bx062668 | AT1G16850 | unknown protein | -2.257 | -2.506 | -1.565 |
| 2 | 45937 | bx062743 | AT5G63860 | UVB-RESISTANCE 8, Regulator of chromosome condensation (RCC1) family protein | -2.387 | -1.925 | -1.434 |
| 2 | 46028 | bx062868 | AT4G26590 | ARABIDOPSIS THALIANA OLIGOPEPTIDE TRANSPORTER 5, oligopeptide transporter 5 | -2.610 | -2.777 | -2.756 |
| 2 | 46031 | bx062874 |  |  | -3.737 | -5.390 | -0.915 |
| 2 | 46076 | bx062933 |  |  | -1.968 | -2.751 | -2.256 |
| 2 | 46129 | bx062996 |  |  | -4.179 | -3.782 | -3.466 |
| 2 | 46191 | bx063074 |  |  | -2.873 | -2.094 | -1.457 |
| 2 | 46209 | bx063095 | AT5G40250 | RING/U-box superfamily protein | -2.598 | -2.285 | -2.423 |
| 2 | 46312 | bx063234 | AT1G53990 | GDSL-motif lipase 3 | -2.675 | -2.507 | -1.934 |
| 2 | 46387 | bx063323 |  |  | -2.402 | -2.949 | -1.191 |
| 2 | 46403 | bx063340 | AT1G21090 | Cupredoxin superfamily protein | -3.401 | -2.053 | -3.180 |
| 2 | 46761 | bx063772 |  |  | -3.677 | -3.365 | -3.075 |
| 2 | 46804 | bx063820 | AT3G16250 | Photosynthetic NDH subcomplex B 3, NDH-dependent cyclic electron flow 1 | -3.851 | -4.444 | -2.729 |
| 2 | 46863 | bx063896 | AT3G54400 | Eukaryotic aspartyl protease family protein | -3.133 | -2.312 | -2.586 |
| 2 | 46880 | bx063915 | AT1G11580 | methylesterase PCR A | -2.683 | -2.550 | -2.787 |
| 2 | 46886 | bx063921 | AT3G19540 | Protein of unknown function (DUF620) | -2.772 | -1.725 | -2.051 |
| 2 | 47114 | bx064177 | AT1G11580 | methylesterase PCR A | -2.435 | -1.557 | -2.062 |
| 2 | 47174 | bx064244 |  |  | -3.221 | -3.097 | -2.158 |
| 2 | 47229 | bx064306 | AT3G48200 | unknown protein | -3.776 | -3.109 | -2.192 |
| 2 | 47374 | bx064470 |  |  | -2.917 | -3.160 | -1.828 |
| 2 | 47550 | bx064672 | AT1G31320 | LOB domain-containing protein 4 | -2.585 | -2.732 | -2.903 |
| 2 | 47587 | bx064712 | AT3G25400 | unknown protein | -3.130 | -2.479 | -2.427 |
| 2 | 47608 | bx064734 | AT1G13710 | KLUH, cytochrome P450, family 78, subfamily A, polypeptide 5, cytochrome P450, family 78, subfamily A, polypeptide 5 | -3.291 | -3.314 | -1.697 |
| 2 | 47638 | bx064765 | AT5G63410 | Leucine-rich repeat protein kinase family protein | -2.627 | -1.847 | -2.526 |
| 2 | 47653 | bx064783 | AT3G52490 | Double Clp-N motif-containing P-loop nucleoside triphosphate hydrolases superfamily protein | -2.446 | -1.013 | -2.530 |
| 2 | 47873 | bx065028 |  |  | -2.487 | -2.042 | -1.053 |
| 2 | 47911 | bx065072 | AT5G39340 | ARABIDOPSIS THALIANA HISTIDINE-CONTAINING PHOSPHOTRANSMITTER 2, histidine-containing phosphotransmitter 3 | -1.886 | -2.835 | -2.199 |
| 2 | 48014 | bx065190 |  |  | -2.349 | -2.238 | -2.598 |
| 2 | 48042 | bx065223 | AT5G01780 | 2-oxoglutarate-dependent dioxygenase family protein | -2.042 | -3.415 | -0.706 |
| 2 | 48104 | bx065290 | AT5G14420 | RING domain ligase2 | -2.120 | -3.197 | -2.439 |
| 2 | 48127 | bx065318 |  |  | -2.791 | -2.725 | -2.711 |
| 2 | 48288 | bx065502 | AT5G43290 | ARABIDOPSIS THALIANA WRKY DNA-BINDING PROTEIN 49, WRKY DNA-binding protein 49 | -4.239 | -2.962 | -4.198 |
| 2 | 48292 | bx065507 |  |  | -1.746 | -2.370 | -0.901 |
| 2 | 48414 | bx065676 | AT1G69530 | EXPANSIN 1, expansin A1 | -2.830 | -1.932 | -2.393 |
| 2 | 48418 | bx065683 | AT3G61920 | unknown protein | -2.179 | -3.398 | -1.747 |
| 2 | 48546 | bx065843 | AT3G25270 | Ribonuclease H-like superfamily protein | -2.080 | -2.768 | -1.450 |
| 2 | 48603 | bx065915 |  |  | -1.864 | -2.792 | -2.184 |
| 2 | 48605 | bx065917 |  |  | -2.709 | -1.902 | -2.556 |
| 2 | 48687 | bx066022 | AT2G43060 | ILI1 binding bHLH 1 | -2.989 | -2.311 | -2.106 |
| 2 | 48776 | bx066140 | AT1G11080 | serine carboxypeptidase-like 31 | -4.842 | -4.142 | -4.391 |
| 2 | 48827 | bx066207 | AT3G28857 | Paclobutrazol Resistance 5, basic helix-loop-helix (bHLH) DNA-binding family protein | -4.312 | -2.666 | -3.314 |
| 2 | 48989 | bx066408 |  |  | -2.428 | -3.302 | -0.810 |
| 2 | 49036 | bx066473 | AT1G01320 | Tetratricopeptide repeat (TPR)-like superfamily protein | -2.666 | -1.982 | -1.435 |
| 2 | 49090 | bx066541 | AT2G47240 | LONG-CHAIN ACYL-COA SYNTHASE 1, ECERIFERUM 8, AMP-dependent synthetase and ligase family protein | -2.515 | -2.117 | -2.013 |
| 2 | 49118 | bx066574 | AT5G14750 | WEREWOLF 1, WEREWOLF, myb domain protein 66 | -4.142 | -3.467 | -1.854 |
| 2 | 49132 | bx066590 | AT1G80870 | Protein kinase superfamily protein | -3.297 | -2.016 | -2.135 |
| 2 | 49192 | bx066668 |  |  | -2.946 | -2.436 | -2.286 |
| 2 | 49214 | bx066692 |  |  | -3.202 | -2.499 | -2.597 |
| 2 | 49283 | bx066773 |  |  | -3.453 | -2.073 | -2.215 |
| 2 | 49306 | bx066800 |  |  | -1.275 | -2.953 | -0.314 |
| 2 | 49485 | bx067008 |  |  | -2.448 | -2.274 | -2.352 |
| 2 | 49843 | bx067433 |  |  | -3.072 | -2.580 | -2.384 |
| 2 | 49937 | bx067539 |  |  | -1.321 | -2.378 | -1.176 |
| 2 | 50087 | bx067711 |  |  | -1.629 | -2.557 | -1.743 |
| 2 | 50202 | bx067843 |  |  | -1.982 | -2.634 | -1.875 |
| 2 | 50205 | bx067846 | AT5G57670 | Protein kinase superfamily protein | -2.607 | -3.290 | -2.610 |
| 2 | 50270 | bx067922 |  |  | -1.108 | -2.906 | -1.253 |
| 2 | 50464 | bx068138 |  |  | -2.819 | -2.503 | -1.333 |
| 2 | 50496 | bx068177 |  |  | -2.624 | -1.495 | -2.293 |
| 2 | 50530 | bx068216 | AT3G45780 | ROOT PHOTOTROPISM 1, NONPHOTOTROPIC HYPOCOTYL 1, phototropin 1 | -2.542 | -2.219 | -1.986 |
| 2 | 50792 | bx068550 | AT1G07010 | Shewenella-like protein phosphatase 1, Calcineurin-like metallo-phosphoesterase superfamily protein | -2.414 | -2.684 | -1.556 |
| 2 | 50827 | bx068589 |  |  | -2.513 | -2.161 | -1.599 |
| 2 | 50968 | bx068746 | AT1G42970 | glyceraldehyde-3-phosphate dehydrogenase B subunit | -1.557 | -2.706 | -1.691 |
| 2 | 51000 | bx068791 |  |  | -2.188 | -2.544 | -1.214 |
| 2 | 51048 | bx068852 | AT5G26780 | serine hydroxymethyltransferase 2 | -1.599 | -2.357 | -1.111 |
| 2 | 51131 | bx068953 | AT5G49730 | ferric reduction oxidase 6 | -3.484 | -2.659 | -1.810 |
| 2 | 51144 | bx068968 | AT4G27800 | PROTEIN PHOSPHATASE 1, thylakoid-associated phosphatase 38 | -2.572 | -1.831 | -1.402 |
| 2 | 51734 | bx069858 | AT4G28200 | unknown protein | -2.366 | -1.862 | -1.263 |
| 2 | 52057 | bx070330 | AT5G42800 | dihydroflavonol 4-reductase | -2.282 | -2.805 | -2.253 |
| 2 | 52117 | bx070416 |  |  | -2.100 | -2.445 | -2.088 |
| 2 | 52154 | bx070469 | AT3G12830 | SAUR-like auxin-responsive protein family | -2.252 | -2.753 | -1.455 |
| 2 | 52220 | bx070555 |  |  | -2.792 | -2.696 | -2.023 |
| 2 | 52297 | bx070659 | AT1G44000 | unknown protein | -1.523 | -2.555 | -1.660 |
| 2 | 52433 | bx070898 |  |  | -2.519 | -1.848 | -1.680 |
| 2 | 52505 | bx071001 | AT4G37930 | SERINE HYDROXYMETHYLTRANSFERASE 1, serine transhydroxymethyltransferase 1 | -1.125 | -2.363 | -1.106 |
| 2 | 52554 | bx071073 | AT5G19520 | mechanosensitive channel of small conductance-like 9 | -2.915 | -1.798 | -2.827 |
| 2 | 52828 | bx071528 | AT1G23390 | Kelch repeat-containing F-box family protein | -1.418 | -3.479 | -1.677 |
| 2 | 52958 | bx071693 | AT3G07350 | Protein of unknown function (DUF506) | -1.179 | -2.345 | -0.627 |
| 2 | 53038 | bx071784 |  |  | -2.423 | -1.906 | -2.010 |
| 2 | 53056 | bx071805 | AT5G07010 | ARABIDOPSIS THALIANA SULFOTRANSFERASE 2A, sulfotransferase 2A | -4.154 | -2.756 | -2.676 |
| 2 | 53086 | bx071838 | AT4G14740 | Plant protein of unknown function (DUF828) with plant pleckstrin homology-like region | -3.123 | -2.776 | -3.250 |
| 2 | 53164 | bx071921 |  |  | -2.316 | -1.938 | -0.948 |
| 2 | 53212 | bx071977 | AT5G19740 | Peptidase M28 family protein | -2.587 | -2.606 | -1.220 |
| 2 | 53341 | bx072121 |  |  | -2.311 | -2.068 | -1.723 |
| 2 | 53407 | bx072193 | AT1G24130 | Transducin/WD40 repeat-like superfamily protein | -3.325 | -3.291 | -2.133 |
| 2 | 53573 | bx072385 | AT5G54530 | Protein of unknown function, DUF538 | -3.816 | -2.580 | -3.169 |
| 2 | 53720 | bx072572 |  |  | -2.439 | -2.231 | -1.487 |
| 2 | 53952 | bx072845 |  |  | -2.697 | -2.127 | -2.184 |
| 2 | 54018 | bx072919 |  |  | -2.476 | -1.919 | -1.517 |
| 2 | 54079 | bx072986 | AT4G29080 | indole-3-acetic acid inducible 27, phytochrome-associated protein 2 | -2.068 | -2.432 | -2.350 |
| 2 | 54234 | bx073159 |  |  | -1.672 | -2.479 | -1.537 |
| 2 | 54274 | bx073202 |  |  | -1.457 | -2.323 | -1.632 |
| 2 | 54278 | bx073207 |  |  | -3.023 | -2.759 | -2.074 |
| 2 | 54281 | bx073210 |  |  | -2.382 | -2.879 | -1.476 |
| 2 | 54319 | bx073254 | AT5G56750 | N-MYC downregulated-like 1 | -2.082 | -2.862 | -2.493 |
| 2 | 54476 | bx073440 | AT2G43180 | Phosphoenolpyruvate carboxylase family protein | -2.487 | -1.501 | -1.835 |
| 2 | 54494 | bx073462 |  |  | -2.769 | -2.391 | -2.639 |
| 2 | 54526 | bx073499 |  |  | -2.460 | -3.269 | -1.074 |
| 2 | 54531 | bx073504 | AT5G60900 | receptor-like protein kinase 1 | -2.415 | -1.620 | -2.099 |
| 2 | 54684 | bx073675 |  |  | -2.554 | -2.267 | -2.059 |
| 2 | 54788 | bx073784 | AT5G48940 | Leucine-rich repeat transmembrane protein kinase family protein | -2.648 | -1.668 | -2.595 |
| 2 | 54885 | bx073887 |  |  | -2.792 | -2.519 | -2.381 |
| 2 | 54929 | bx073936 |  |  | -2.243 | -3.182 | -2.327 |
| 2 | 55369 | bx074485 | AT3G14810 | mechanosensitive channel of small conductance-like 5 | -3.495 | -3.175 | -2.414 |
| 2 | 55423 | bx074555 | AT2G16385 | unknown protein | -3.170 | -2.588 | -2.528 |
| 2 | 55525 | bx074682 | AT3G15530 | S-adenosyl-L-methionine-dependent methyltransferases superfamily protein | -2.468 | -1.600 | -2.348 |
| 2 | 55568 | bx074735 |  |  | -0.859 | -2.470 | -1.108 |
| 2 | 55712 | bx074994 | AT1G75590 | SAUR-like auxin-responsive protein family | -2.835 | -1.909 | -1.533 |
| 2 | 55848 | bx075198 | AT3G06130 | Heavy metal transport/detoxification superfamily protein | -2.483 | -1.960 | -2.276 |
| 2 | 55859 | bx075213 |  |  | -1.745 | -2.880 | -2.040 |
| 2 | 55945 | bx075337 | AT2G44190 | QWRF domain containing 5, EMBRYO DEFECTIVE 3116, ENDOSPERM DEFECTIVE 1, Family of unknown function (DUF566) | -1.313 | -2.415 | -1.593 |
| 2 | 55998 | bx075420 | AT1G75710 | C2H2-like zinc finger protein | -2.271 | -2.397 | -1.759 |
| 2 | 56011 | bx075450 | AT1G28100 | unknown protein | -2.569 | -2.270 | -1.018 |
| 2 | 56025 | bx075478 |  |  | -2.795 | -2.035 | -2.964 |
| 2 | 56081 | bx075559 |  |  | -3.235 | -1.989 | -3.155 |
| 2 | 56415 | bx075997 | AT5G25590 | Protein of unknown function (DUF630 and DUF632) | -1.950 | -2.716 | -2.231 |
| 2 | 56539 | bx076144 |  |  | -3.386 | -3.600 | -2.477 |
| 2 | 56934 | bx076583 | AT5G54060 | UDP-glucose:flavonoid 3-o-glucosyltransferase | -1.853 | -3.484 | -1.225 |
| 2 | 56973 | bx076623 | AT2G41510 | cytokinin oxidase/dehydrogenase 1 | -1.483 | -2.539 | -1.500 |
| 2 | 57124 | bx076803 | AT5G25530 | DNAJ heat shock family protein | -1.862 | -3.101 | -1.633 |
| 2 | 57222 | bx076933 | AT5G38510 | Rhomboid-related intramembrane serine protease family protein | -2.552 | -2.013 | -1.761 |
| 2 | 57377 | bx077128 |  |  | -0.601 | -2.465 | -0.671 |
| 2 | 57389 | bx077151 | AT2G33180 | unknown protein | -3.122 | -3.876 | -2.452 |
| 2 | 57432 | bx077214 | AT4G11960 | PGR5-like B | -2.281 | -2.537 | -1.468 |
| 2 | 57448 | bx077235 | AT1G74880 | NADH dehydrogenase-like complex ), NAD(P)H:plastoquinone dehydrogenase complex subunit O | -1.618 | -2.691 | -1.696 |
| 2 | 57476 | bx077276 |  |  | -2.314 | -1.779 | -2.246 |
| 2 | 57598 | bx077416 |  |  | -4.472 | -4.709 | -3.392 |
| 2 | 57607 | bx077433 |  |  | -2.649 | -4.458 | -2.665 |
| 2 | 57841 | bx077706 | AT4G00460 | RHO guanyl-nucleotide exchange factor 3 | -2.042 | -2.883 | -2.256 |
| 2 | 57981 | bx077893 | AT2G44480 | beta glucosidase 17 | -3.031 | -2.169 | -1.747 |
| 2 | 57987 | bx077904 | AT1G65230 | Uncharacterized conserved protein (DUF2358) | -2.767 | -2.482 | -1.627 |
| 2 | 58076 | bx078008 | AT4G25570 | Cytochrome b561/ferric reductase transmembrane protein family | -2.639 | -2.737 | -1.397 |
| 2 | 58098 | bx078032 | AT5G67370 | Protein of unknown function (DUF1230) | -2.382 | -2.563 | -1.104 |
| 2 | 58162 | bx078102 |  |  | -1.542 | -2.394 | -1.188 |
| 2 | 58198 | bx078142 | AT1G70760 | NADH dehydrogenase-like complex L, CHLORORESPIRATORY REDUCTION 23, inorganic carbon transport protein-related | -2.783 | -3.407 | -1.787 |
| 2 | 58684 | bx078703 | AT1G17870 | ethylene-dependent gravitropism-deficient and yellow-green-like 3 | -2.359 | -2.226 | -0.844 |
| 2 | 58699 | bx078720 | AT5G49730 | ferric reduction oxidase 6 | -3.362 | -2.845 | -2.127 |
| 2 | 58861 | bx078926 | AT1G65230 | Uncharacterized conserved protein (DUF2358) | -3.582 | -2.825 | -1.669 |
| 2 | 58874 | bx078943 | AT1G74880 | NADH dehydrogenase-like complex ), NAD(P)H:plastoquinone dehydrogenase complex subunit O | -1.589 | -2.674 | -1.694 |
| 2 | 58963 | bx079047 | AT5G59870 | histone H2A 6 | -1.436 | -2.550 | -1.775 |
| 2 | 59030 | bx079126 | AT1G08380 | photosystem I subunit O | -1.683 | -2.469 | -1.530 |
| 2 | 59095 | bx079203 |  |  | -2.170 | -2.498 | -2.229 |
| 2 | 59459 | bx079622 |  |  | -3.755 | -3.987 | -2.387 |
| 2 | 59465 | bx079628 | AT5G54530 | Protein of unknown function, DUF538 | -3.642 | -2.613 | -3.617 |
| 2 | 59475 | bx079639 |  |  | -2.940 | -2.814 | -2.807 |
| 2 | 59535 | bx079715 | AT4G23496 | SPIRAL1-like5 | -2.533 | -3.163 | -2.478 |
| 2 | 59558 | bx079745 |  |  | -3.812 | -5.423 | -4.555 |
| 2 | 59591 | bx079783 |  |  | -4.409 | -2.734 | -2.840 |
| 2 | 59635 | bx079829 | AT1G65450 | HXXXD-type acyl-transferase family protein | -2.326 | -2.709 | -1.990 |
| 2 | 59642 | bx079836 | AT5G02140 | Pathogenesis-related thaumatin superfamily protein | -2.754 | -1.698 | -2.995 |
| 2 | 59761 | bx079985 | AT4G32980 | homeobox gene 1 | -3.434 | -3.412 | -2.992 |
| 2 | 59924 | bx080189 | AT1G15125 | S-adenosyl-L-methionine-dependent methyltransferases superfamily protein | -2.786 | -2.708 | -1.076 |
| 2 | 59982 | bx080270 | AT5G48460 | Actin binding Calponin homology (CH) domain-containing protein | -3.503 | -1.921 | -2.511 |
| 2 | 60164 | bx080512 |  |  | -3.762 | -2.421 | -2.655 |
| 2 | 60204 | bx080561 |  |  | -2.144 | -3.428 | -2.591 |
| 2 | 60225 | bx080585 |  |  | -2.699 | -3.973 | -2.894 |
| 2 | 60228 | bx080588 | AT1G45207 | Remorin family protein | -3.397 | -3.202 | -2.575 |
| 2 | 60385 | bx080799 | AT1G53400 | Ubiquitin domain-containing protein | -2.937 | -2.638 | -1.441 |
| 2 | 60487 | bx080926 |  |  | -2.363 | -2.382 | -2.451 |
| 2 | 60519 | bx080967 | AT4G39210 | Glucose-1-phosphate adenylyltransferase family protein | -2.450 | -2.470 | -2.316 |
| 2 | 60577 | bx081040 | AT5G52100 | chlororespiration reduction 1, Dihydrodipicolinate reductase, bacterial/plant | -2.596 | -2.904 | -1.966 |
| 2 | 60633 | bx081113 | AT5G04820 | ARABIDOPSIS THALIANA OVATE FAMILY PROTEIN 13, ovate family protein 13 | -2.466 | -1.938 | -1.536 |
| 2 | 60670 | bx081156 |  |  | -3.791 | -4.065 | -3.537 |
| 2 | 60698 | bx081189 |  |  | -2.426 | -2.320 | -2.222 |
| 2 | 60862 | bx081388 | AT3G19184 | AP2/B3-like transcriptional factor family protein | -1.763 | -3.148 | -1.783 |
| 2 | 60888 | bx081421 | AT1G45207 | Remorin family protein | -2.215 | -2.026 | -2.337 |
| 2 | 60913 | bx081452 | AT5G62710 | Leucine-rich repeat protein kinase family protein | -2.312 | -1.261 | -2.098 |
| 2 | 60953 | bx081517 |  |  | -2.606 | -2.161 | -1.629 |
| 2 | 60957 | bx081521 | AT1G24020 | MLP-like protein 423 | -4.266 | -4.729 | -1.402 |
| 2 | 61008 | bx081581 | AT3G56040 | UDP-glucose pyrophosphorylase 3 | -3.787 | -3.306 | -3.698 |
| 2 | 61079 | bx081663 |  |  | -2.589 | -1.703 | -1.848 |
| 2 | 61091 | bx081676 |  |  | -3.463 | -2.199 | -2.721 |
| 2 | 61165 | bx081769 | AT4G36850 | PQ-loop repeat family protein / transmembrane family protein | -3.830 | -3.524 | -2.384 |
| 2 | 61358 | bx082065 | AT3G54070 | Ankyrin repeat family protein | -3.962 | -2.990 | -2.424 |
| 2 | 61385 | bx082113 |  |  | -2.347 | -2.436 | -2.518 |
| 2 | 61408 | bx082142 | AT3G48200 | unknown protein | -3.407 | -3.014 | -2.260 |
| 2 | 61435 | bx082184 | AT5G10250 | DEFECTIVELY ORGANIZED TRIBUTARIES 3, Phototropic-responsive NPH3 family protein | -2.819 | -2.769 | -1.956 |
| 2 | 61530 | bx082343 |  |  | -2.891 | -2.168 | -2.272 |
| 2 | 61632 | bx082495 |  |  | -1.619 | -2.365 | -1.782 |
| 2 | 61635 | bx082499 |  |  | -2.842 | -2.301 | -2.274 |
| 3 | 181 | bx000332 |  |  | -1.378 | -1.885 | -3.030 |
| 3 | 281 | bx000511 |  |  | -1.343 | -1.727 | -2.575 |
| 3 | 427 | bx000759 | AT4G37470 | alpha/beta-Hydrolases superfamily protein | -0.366 | -2.412 | -2.274 |
| 3 | 515 | bx000908 | AT1G18250 | Pathogenesis-related thaumatin superfamily protein | -2.675 | -3.231 | -3.829 |
| 3 | 526 | bx000927 |  |  | -1.022 | -1.303 | -2.383 |
| 3 | 541 | bx000957 | AT4G39350 | cellulose synthase A2 | -1.887 | -2.403 | -2.473 |
| 3 | 556 | bx000982 | AT4G24510 | ECERIFERUM 2, HXXXD-type acyl-transferase family protein | -2.631 | -3.253 | -3.133 |
| 3 | 564 | bx000998 | AT3G20015 | Eukaryotic aspartyl protease family protein | -1.814 | -2.647 | -3.456 |
| 3 | 650 | bx001144 | AT1G04680 | Pectin lyase-like superfamily protein | -2.280 | -1.942 | -2.777 |
| 3 | 768 | bx001355 | AT5G26660 | myb domain protein 86 | -1.129 | -2.793 | -2.899 |
| 3 | 793 | bx001391 | AT3G23590 | REF4-related 1 | -2.107 | -2.180 | -2.894 |
| 3 | 1024 | bx001761 |  |  | -1.399 | -1.956 | -2.655 |
| 3 | 1361 | bx002294 | AT3G01500 | ARABIDOPSIS THALIANA SALICYLIC ACID-BINDING PROTEIN 3, BETA CARBONIC ANHYDRASE 1, carbonic anhydrase 1 | -2.446 | -4.493 | -3.878 |
| 3 | 1663 | bx002746 | AT3G18670 | Ankyrin repeat family protein | -2.203 | -1.604 | -2.654 |
| 3 | 1674 | bx002764 | AT2G27310 | F-box family protein | -1.088 | -1.918 | -2.889 |
| 3 | 1860 | bx003053 |  |  | -2.480 | -4.902 | -5.064 |
| 3 | 1937 | bx003163 |  |  | -3.527 | -4.477 | -4.619 |
| 3 | 2101 | bx003417 | AT3G53720 | cation/H+ exchanger 20, cation/H+ exchanger 20, cation/H+ exchanger 20 | -2.156 | -1.820 | -3.567 |
| 3 | 2134 | bx003472 | AT3G24660 | transmembrane kinase-like 1 | -2.131 | -2.057 | -2.690 |
| 3 | 2396 | bx003855 |  |  | -1.945 | -2.195 | -2.897 |
| 3 | 2443 | bx003925 | AT5G35630 | GLUTAMINE SYNTHETASE LIKE 1, glutamine synthetase 2 | -2.093 | -1.391 | -2.931 |
| 3 | 2481 | bx003988 | AT5G54190 | protochlorophyllide oxidoreductase A | -1.938 | -2.339 | -3.210 |
| 3 | 2525 | bx004053 | AT5G65670 | indole-3-acetic acid inducible 9 | -1.313 | -1.995 | -2.696 |
| 3 | 2540 | bx004076 | AT5G55540 | LOPPED 1, tornado 1 | -2.018 | -1.993 | -3.304 |
| 3 | 2625 | bx004213 | AT3G17350 | unknown protein | -2.863 | -3.598 | -4.421 |
| 3 | 2678 | bx004291 | AT3G54140 | ARABIDOPSIS THALIANA PEPTIDE TRANSPORTER 1, peptide transporter 1 | -0.665 | -1.804 | -2.585 |
| 3 | 2734 | bx004370 | AT5G61480 | TDIF receptor, PHLOEM INTERCALATED WITH XYLEM, Leucine-rich repeat protein kinase family protein | -1.601 | -2.092 | -2.820 |
| 3 | 2827 | bx004500 | AT1G18610 | Galactose oxidase/kelch repeat superfamily protein | -2.096 | -2.720 | -3.586 |
| 3 | 2838 | bx004521 | AT1G76610 | Protein of unknown function, DUF617 | -2.296 | -2.874 | -3.342 |
| 3 | 2958 | bx004711 | AT4G22340 | cytidinediphosphate diacylglycerol synthase 2 | -1.793 | -2.297 | -3.224 |
| 3 | 3096 | bx004902 | AT3G01500 | ARABIDOPSIS THALIANA SALICYLIC ACID-BINDING PROTEIN 3, BETA CARBONIC ANHYDRASE 1, carbonic anhydrase 1 | -1.736 | -3.581 | -3.450 |
| 3 | 3209 | bx005070 | AT1G62400 | high leaf temperature 1, Protein kinase superfamily protein | -1.864 | -1.931 | -2.530 |
| 3 | 3247 | bx005116 | AT5G48740 | Leucine-rich repeat protein kinase family protein | -2.347 | -2.419 | -3.052 |
| 3 | 3257 | bx005128 | AT4G38050 | Xanthine/uracil permease family protein | -2.020 | -1.401 | -2.469 |
| 3 | 3330 | bx005240 | AT4G28950 | Arabidopsis RAC-like 7, RHO-related protein from plants 9 | -1.398 | -2.038 | -3.280 |
| 3 | 3358 | bx005280 | AT3G24770 | CLAVATA3/ESR-RELATED 41 | -3.360 | -2.556 | -4.648 |
| 3 | 3393 | bx005324 |  |  | -1.983 | -1.403 | -2.779 |
| 3 | 3396 | bx005328 | AT5G33370 | GDSL-like Lipase/Acylhydrolase superfamily protein | -2.536 | -2.771 | -3.282 |
| 3 | 3479 | bx005456 | AT1G34670 | myb domain protein 93 | -0.716 | -2.437 | -2.511 |
| 3 | 3489 | bx005470 |  |  | -2.068 | -3.300 | -3.785 |
| 3 | 3634 | bx005685 | AT1G76610 | Protein of unknown function, DUF617 | -2.339 | -3.016 | -3.244 |
| 3 | 3674 | bx005740 |  |  | -1.744 | -2.311 | -2.798 |
| 3 | 3743 | bx005843 | AT1G30690 | Sec14p-like phosphatidylinositol transfer family protein | -1.472 | -2.554 | -2.725 |
| 3 | 3778 | bx005893 | AT3G60140 | SENESCENCE-RELATED GENE 2, DARK INDUCIBLE 2, BETA GLUCOSIDASE 30, Glycosyl hydrolase superfamily protein | -2.211 | -2.153 | -3.888 |
| 3 | 4013 | bx006241 | AT2G22620 | Rhamnogalacturonate lyase family protein | -2.530 | -1.896 | -3.956 |
| 3 | 4043 | bx006281 | AT2G30400 | ARABIDOPSIS THALIANA OVATE FAMILY PROTEIN 2, ovate family protein 2 | -1.906 | -3.197 | -2.704 |
| 3 | 4181 | bx006479 | AT5G15630 | IRREGULAR XYLEM 6, COBRA-LIKE4, COBRA-like extracellular glycosyl-phosphatidyl inositol-anchored protein family | -2.023 | -1.824 | -2.694 |
| 3 | 4312 | bx006673 |  |  | -2.792 | -3.171 | -3.564 |
| 3 | 4651 | bx007175 | AT4G37760 | squalene epoxidase 3 | -2.060 | -2.467 | -3.495 |
| 3 | 4702 | bx007243 | AT1G04110 | STOMATAL DENSITY AND DISTRIBUTION, Subtilase family protein | -2.485 | -3.662 | -3.133 |
| 3 | 4734 | bx007288 | AT1G10120 | basic helix-loop-helix (bHLH) DNA-binding superfamily protein | -1.944 | -2.208 | -3.273 |
| 3 | 4891 | bx007515 | AT5G10510 | PLETHORA 3, AINTEGUMENTA-like 6 | -2.373 | -2.317 | -3.605 |
| 3 | 4902 | bx007528 | AT5G14570 | high affinity nitrate transporter 2.7 | -2.305 | -3.232 | -3.337 |
| 3 | 5137 | bx007869 | AT3G01500 | ARABIDOPSIS THALIANA SALICYLIC ACID-BINDING PROTEIN 3, BETA CARBONIC ANHYDRASE 1, carbonic anhydrase 1 | -0.837 | -2.733 | -1.974 |
| 3 | 5203 | bx007956 | AT1G78430 | ROP interactive partner 2 | -1.583 | -2.042 | -2.428 |
| 3 | 5214 | bx007973 | AT5G67370 | Protein of unknown function (DUF1230) | 0.065 | -2.620 | -1.794 |
| 3 | 5247 | bx008014 | AT1G62360 | WALDMEISTER 1, WALDMEISTER, SHOOT MERISTEMLESS, SHOOTLESS, BUMBERSHOOT 1, BUMBERSHOOT, KNOX/ELK homeobox transcription factor | -1.857 | -2.535 | -2.797 |
| 3 | 5356 | bx008174 |  |  | -2.129 | -1.972 | -2.796 |
| 3 | 5362 | bx008185 | AT1G49960 | Xanthine/uracil permease family protein | -1.910 | -3.281 | -3.963 |
| 3 | 5404 | bx008245 | AT2G47700 | RED AND FAR-RED INSENSITIVE 2, RING/U-box superfamily protein | -1.276 | -1.917 | -2.694 |
| 3 | 5416 | bx008257 | AT5G42260 | beta glucosidase 12 | -2.421 | -2.148 | -3.952 |
| 3 | 5418 | bx008259 |  |  | -4.211 | -4.478 | -4.977 |
| 3 | 5422 | bx008264 | AT1G75030 | thaumatin-like protein 3 | -1.635 | -3.091 | -4.325 |
| 3 | 5535 | bx008437 | AT4G23500 | Pectin lyase-like superfamily protein | -1.159 | -1.385 | -2.344 |
| 3 | 5538 | bx008441 | AT3G51930 | Transducin/WD40 repeat-like superfamily protein | -0.289 | -2.395 | -2.296 |
| 3 | 5551 | bx008462 | AT3G61640 | arabinogalactan protein 20 | -1.068 | -1.518 | -2.385 |
| 3 | 5644 | bx008587 | AT1G68585 | unknown protein | -2.764 | -2.869 | -3.751 |
| 3 | 5691 | bx008659 | AT3G09510 | Ribonuclease H-like superfamily protein | -1.725 | -1.646 | -2.376 |
| 3 | 5782 | bx008790 | AT5G07180 | ERECTA-like 2 | -1.873 | -3.609 | -2.786 |
| 3 | 5802 | bx008815 | AT3G53190 | Pectin lyase-like superfamily protein | -1.829 | -1.722 | -3.111 |
| 3 | 5807 | bx008822 | AT1G70280 | NHL domain-containing protein | -1.248 | -1.341 | -2.432 |
| 3 | 5894 | bx008940 |  |  | -1.328 | -2.382 | -3.943 |
| 3 | 5943 | bx008994 | AT2G41610 | unknown protein | -2.472 | -2.783 | -4.592 |
| 3 | 5944 | bx008995 | AT1G66250 | O-Glycosyl hydrolases family 17 protein | -2.308 | -2.598 | -2.914 |
| 3 | 5991 | bx009059 |  |  | 0.123 | -2.984 | -3.313 |
| 3 | 6072 | bx009165 | AT2G42410 | zinc finger protein 11 | -2.507 | -2.293 | -2.887 |
| 3 | 6254 | bx009425 | AT1G63100 | GRAS family transcription factor | -1.546 | -2.765 | -2.970 |
| 3 | 6286 | bx009474 | AT3G51740 | inflorescence meristem receptor-like kinase 2 | -1.097 | -3.015 | -2.676 |
| 3 | 6514 | bx009816 | AT5G48170 | SNEEZY, SLEEPY2, F-box family protein | -0.862 | -2.323 | -2.558 |
| 3 | 6528 | bx009835 | AT4G23160 | cysteine-rich RLK (RECEPTOR-like protein kinase) 8 | -1.245 | -2.646 | -1.723 |
| 3 | 6530 | bx009837 | AT2G36885 | unknown protein | -0.965 | -3.679 | -2.345 |
| 3 | 6576 | bx009896 | AT1G75030 | thaumatin-like protein 3 | -1.481 | -2.305 | -3.150 |
| 3 | 6585 | bx009909 | AT1G28110 | serine carboxypeptidase-like 45 | -1.523 | -2.280 | -3.079 |
| 3 | 6601 | bx009937 | AT1G07850 | Protein of unknown function (DUF604) | -2.540 | -2.262 | -3.218 |
| 3 | 6617 | bx009955 |  |  | -2.671 | -2.792 | -3.131 |
| 3 | 6689 | bx010054 | AT4G03500 | Ankyrin repeat family protein | -1.241 | -2.521 | -2.008 |
| 3 | 6746 | bx010136 | AT5G66920 | SKU5 similar 17, SKU5 similar 17 | -1.403 | -2.405 | -3.630 |
| 3 | 6935 | bx010392 | AT1G72430 | SAUR-like auxin-responsive protein family | -1.770 | -3.207 | -4.067 |
| 3 | 6941 | bx010403 | AT4G20940 | Leucine-rich receptor-like protein kinase family protein | -1.272 | -1.514 | -2.640 |
| 3 | 7099 | bx010636 | AT3G51970 | ARABIDOPSIS THALIANA STEROL O-ACYLTRANSFERASE 1, acyl-CoA sterol acyl transferase 1 | -1.620 | -3.070 | -3.767 |
| 3 | 7166 | bx010729 | AT2G12400 | unknown protein | -1.955 | -2.675 | -4.403 |
| 3 | 7167 | bx010730 | AT5G50150 | Protein of Unknown Function (DUF239) | -1.569 | -1.515 | -2.566 |
| 3 | 7193 | bx010765 |  |  | -0.962 | -1.369 | -2.341 |
| 3 | 7571 | bx011284 | AT1G09920 | TRAF-type zinc finger-related | -2.311 | -1.604 | -2.685 |
| 3 | 7693 | bx011470 | AT1G04680 | Pectin lyase-like superfamily protein | -2.746 | -3.377 | -4.075 |
| 3 | 7699 | bx011483 | AT5G64080 | xylogen protein 1, Bifunctional inhibitor/lipid-transfer protein/seed storage 2S albumin superfamily protein | -1.778 | -1.985 | -2.318 |
| 3 | 7848 | bx011706 | AT5G54850 | unknown protein | -0.873 | -1.902 | -2.421 |
| 3 | 7908 | bx011786 | AT1G68725 | arabinogalactan protein 19 | -0.748 | -1.800 | -2.353 |
| 3 | 7929 | bx011814 | AT1G09450 | Haspin-related gene, Protein kinase superfamily protein | -0.714 | -2.686 | -1.938 |
| 3 | 8018 | bx011930 | AT1G06660 | JASON, unknown protein | -1.731 | -1.753 | -2.388 |
| 3 | 8020 | bx011932 | AT3G52110 | unknown protein | -1.793 | -2.466 | -2.871 |
| 3 | 8089 | bx012026 | AT1G71380 | ARABIDOPSIS THALIANA GLYCOSYL HYDROLASE 9B3, cellulase 3 | -2.078 | -2.483 | -2.978 |
| 3 | 8144 | bx012107 | AT5G47800 | Phototropic-responsive NPH3 family protein | -1.970 | -1.808 | -3.130 |
| 3 | 8510 | bx012629 | AT3G45650 | nitrate excretion transporter1 | -5.047 | -6.311 | -6.626 |
| 3 | 8547 | bx012676 | AT4G13210 | Pectin lyase-like superfamily protein | -2.345 | -3.985 | -5.927 |
| 3 | 8626 | bx012789 | AT1G68520 | B-box type zinc finger protein with CCT domain | -2.573 | -2.640 | -3.432 |
| 3 | 8678 | bx012858 | AT4G22570 | adenine phosphoribosyl transferase 3 | -1.798 | -1.913 | -3.014 |
| 3 | 8687 | bx012872 | AT2G16070 | plastid division2 | -1.704 | -2.516 | -2.942 |
| 3 | 8861 | bx013113 | AT5G15900 | TRICHOME BIREFRINGENCE-LIKE 19 | -0.852 | -2.775 | -2.679 |
| 3 | 8993 | bx013313 | AT3G51895 | sulfate transporter 3;1 | -0.913 | -2.555 | -2.259 |
| 3 | 9038 | bx013378 | AT4G20970 | basic helix-loop-helix (bHLH) DNA-binding superfamily protein | -1.756 | -2.633 | -2.156 |
| 3 | 9090 | bx013447 | AT4G00950 | maternal effect embryo arrest 47, Protein of unknown function (DUF688) | -1.991 | -3.068 | -3.530 |
| 3 | 9243 | bx013680 | AT5G23860 | tubulin beta 8 | -1.689 | -2.893 | -3.043 |
| 3 | 9412 | bx013933 | AT1G21880 | lysm domain GPI-anchored protein 1 precursor | -1.232 | -1.525 | -2.790 |
| 3 | 9464 | bx014005 |  |  | -1.543 | -1.799 | -2.421 |
| 3 | 9676 | bx014304 | AT4G14960 | Tubulin/FtsZ family protein | -0.736 | -1.629 | -2.431 |
| 3 | 9678 | bx014308 | AT3G20015 | Eukaryotic aspartyl protease family protein | -1.849 | -1.542 | -2.703 |
| 3 | 9778 | bx014448 | AT4G34880 | Amidase family protein | -1.327 | -1.303 | -2.448 |
| 3 | 9956 | bx014692 | AT4G34500 | Protein kinase superfamily protein | -2.079 | -2.303 | -2.445 |
| 3 | 9977 | bx014719 | AT4G14960 | Tubulin/FtsZ family protein | -1.289 | -1.613 | -2.757 |
| 3 | 10124 | bx014937 | AT3G60080 | RING/U-box superfamily protein | -2.256 | -3.180 | -3.303 |
| 3 | 10845 | bx016105 | AT5G42260 | beta glucosidase 12 | -2.407 | -1.814 | -2.806 |
| 3 | 11384 | bx016983 | AT3G14860 | NHL domain-containing protein | -1.067 | -2.070 | -2.331 |
| 3 | 11423 | bx017052 | AT5G46230 | Protein of unknown function, DUF538 | -1.270 | -1.688 | -2.420 |
| 3 | 11430 | bx017061 | AT3G01500 | ARABIDOPSIS THALIANA SALICYLIC ACID-BINDING PROTEIN 3, BETA CARBONIC ANHYDRASE 1, carbonic anhydrase 1 | -1.881 | -4.491 | -2.892 |
| 3 | 11514 | bx017194 | AT1G79620 | Leucine-rich repeat protein kinase family protein | -0.686 | -1.523 | -2.450 |
| 3 | 11613 | bx017344 |  |  | -1.085 | -2.406 | -1.817 |
| 3 | 11985 | bx017961 | AT5G10250 | DEFECTIVELY ORGANIZED TRIBUTARIES 3, Phototropic-responsive NPH3 family protein | -0.513 | -2.741 | -2.902 |
| 3 | 12175 | bx018272 | AT1G77920 | bZIP transcription factor family protein | -1.814 | -2.502 | -2.339 |
| 3 | 12259 | bx018403 | AT2G39870 | unknown protein | -1.536 | -1.934 | -2.494 |
| 3 | 12341 | bx018514 | AT2G41705 | camphor resistance CrcB family protein | -1.791 | -1.936 | -2.787 |
| 3 | 12623 | bx018919 | AT1G07200 | Double Clp-N motif-containing P-loop nucleoside triphosphate hydrolases superfamily protein | -1.710 | -1.881 | -2.790 |
| 3 | 12652 | bx018952 | AT5G19190 | unknown protein | -1.853 | -2.800 | -2.932 |
| 3 | 12909 | bx019301 | AT2G47500 | P-loop nucleoside triphosphate hydrolases superfamily protein with CH (Calponin Homology) domain | -1.322 | -2.426 | -3.043 |
| 3 | 13099 | bx019558 |  |  | -1.345 | -2.194 | -3.170 |
| 3 | 13622 | bx020253 | AT2G27380 | extensin proline-rich 1 | -1.375 | -2.405 | -3.901 |
| 3 | 14025 | bx020778 | AT1G04880 | HMG (high mobility group) box protein with ARID/BRIGHT DNA-binding domain | -2.488 | -2.962 | -3.222 |
| 3 | 14178 | bx020994 | AT1G10750 | Protein of Unknown Function (DUF239) | -1.766 | -1.686 | -2.312 |
| 3 | 14403 | bx021286 | AT5G41460 | Protein of unknown function (DUF604) | -1.766 | -1.779 | -2.478 |
| 3 | 14543 | bx021468 | AT5G06720 | peroxidase 2 | -1.075 | -1.795 | -2.416 |
| 3 | 14596 | bx021536 | AT1G69870 | nitrate transporter 1.7 | -2.371 | -3.534 | -3.322 |
| 3 | 14748 | bx021737 | AT5G45970 | RHO-RELATED PROTEIN FROM PLANTS 7, ARABIDOPSIS THALIANA RAC 2, Arabidopsis RAC-like 2, RAC-like 2 | -1.981 | -2.169 | -2.997 |
| 3 | 14871 | bx021908 | AT4G31840 | early nodulin-like protein 15 | -1.497 | -2.365 | -2.676 |
| 3 | 15204 | bx022342 | AT2G04850 | Auxin-responsive family protein | -1.830 | -2.015 | -3.552 |
| 3 | 15327 | bx022500 | AT2G05790 | O-Glycosyl hydrolases family 17 protein | -1.550 | -2.516 | -2.414 |
| 3 | 15343 | bx022522 | AT5G48500 | unknown protein | -1.714 | -1.754 | -2.753 |
| 3 | 15463 | bx022676 | AT1G65560 | Zinc-binding dehydrogenase family protein | -1.196 | -2.978 | -4.449 |
| 3 | 15503 | bx022728 | AT2G32500 | Stress responsive alpha-beta barrel domain protein | -0.888 | -3.784 | -1.879 |
| 3 | 15658 | bx022935 | AT5G15230 | GAST1 protein homolog 4 | -1.445 | -2.858 | -4.423 |
| 3 | 15659 | bx022936 | AT5G67210 | IRX15-LIKE, Protein of unknown function (DUF579) | -2.497 | -2.367 | -2.910 |
| 3 | 15733 | bx023034 |  |  | -1.860 | -2.606 | -3.848 |
| 3 | 15767 | bx023084 | AT1G13080 | cytochrome P450, family 71, subfamily B, polypeptide 2, cytochrome P450, family 71, subfamily B, polypeptide 2 | -1.603 | -2.114 | -2.577 |
| 3 | 15984 | bx023371 | AT4G26320 | arabinogalactan protein 13 | -1.165 | -2.019 | -2.988 |
| 3 | 16073 | bx023496 | AT2G15480 | UDP-glucosyl transferase 73B5 | -2.080 | -2.345 | -2.873 |
| 3 | 16166 | bx023627 | AT3G01190 | Peroxidase superfamily protein | -1.970 | -3.605 | -4.797 |
| 3 | 16193 | bx023669 | AT4G22570 | adenine phosphoribosyl transferase 3 | -1.857 | -1.851 | -2.805 |
| 3 | 16221 | bx023709 | AT5G64080 | xylogen protein 1, Bifunctional inhibitor/lipid-transfer protein/seed storage 2S albumin superfamily protein | -1.773 | -2.205 | -2.473 |
| 3 | 16557 | bx024158 | AT4G38970 | fructose-bisphosphate aldolase 2 | -1.295 | -2.800 | -1.994 |
| 3 | 16625 | bx024276 | AT4G32140 | EamA-like transporter family | -0.835 | -2.258 | -3.044 |
| 3 | 16840 | bx024570 | AT2G02950 | phytochrome kinase substrate 1 | -2.166 | -3.128 | -2.639 |
| 3 | 16862 | bx024598 | AT1G79620 | Leucine-rich repeat protein kinase family protein | -1.328 | -1.634 | -2.364 |
| 3 | 16865 | bx024601 | AT5G14740 | CARBONIC ANHYDRASE 18, BETA CARBONIC ANHYDRASE 2, carbonic anhydrase 2 | -2.140 | -3.710 | -2.912 |
| 3 | 16878 | bx024615 | AT5G06710 | homeobox from Arabidopsis thaliana | -2.724 | -2.998 | -3.605 |
| 3 | 16915 | bx024664 | AT5G14570 | high affinity nitrate transporter 2.7 | -2.336 | -3.269 | -3.458 |
| 3 | 16921 | bx024671 | AT2G44300 | Bifunctional inhibitor/lipid-transfer protein/seed storage 2S albumin superfamily protein | -1.914 | -1.685 | -2.949 |
| 3 | 17046 | bx024827 | AT5G59000 | RING/FYVE/PHD zinc finger superfamily protein | -1.393 | -2.265 | -2.416 |
| 3 | 17295 | bx025148 |  |  | -1.134 | -1.877 | -2.323 |
| 3 | 17474 | bx025409 | AT4G08810 | calcium ion binding | -1.272 | -1.598 | -2.395 |
| 3 | 17568 | bx025539 | AT4G17220 | microtubule-associated proteins 70-5 | -1.757 | -1.398 | -2.516 |
| 3 | 17600 | bx025586 | AT5G53420 | CCT motif family protein | -1.442 | -1.741 | -2.697 |
| 3 | 17664 | bx025670 |  |  | -1.085 | -2.366 | -1.775 |
| 3 | 17679 | bx025693 | AT4G14550 | SOLITARY ROOT, indole-3-acetic acid inducible 14 | -1.795 | -3.681 | -5.220 |
| 3 | 17854 | bx025914 | AT1G67910 | unknown protein | -2.354 | -2.593 | -4.536 |
| 3 | 17864 | bx025928 | AT4G36360 | beta-galactosidase 3 | -2.001 | -2.683 | -2.507 |
| 3 | 17898 | bx025971 | AT1G28110 | serine carboxypeptidase-like 45 | -1.539 | -2.382 | -3.136 |
| 3 | 17956 | bx026055 | AT5G62350 | Plant invertase/pectin methylesterase inhibitor superfamily protein | -1.812 | -1.456 | -2.748 |
| 3 | 17993 | bx026102 | AT5G10430 | arabinogalactan protein 4 | -1.033 | -1.969 | -2.630 |
| 3 | 18276 | bx026477 | AT5G22860 | Serine carboxypeptidase S28 family protein | -1.023 | -3.477 | -2.701 |
| 3 | 18403 | bx026636 | AT5G35630 | GLUTAMINE SYNTHETASE LIKE 1, glutamine synthetase 2 | -0.945 | -2.532 | -1.695 |
| 3 | 18441 | bx026693 | AT1G72220 | RING/U-box superfamily protein | -2.628 | -2.019 | -4.130 |
| 3 | 18490 | bx026759 | AT4G31940 | cytochrome P450, family 82, subfamily C, polypeptide 4, cytochrome P450, family 82, subfamily C, polypeptide 4 | -0.424 | -2.861 | -2.806 |
| 3 | 18519 | bx026797 | AT3G26932 | dsRNA-binding protein 3 | -1.492 | -2.445 | -2.501 |
| 3 | 18521 | bx026799 | AT1G10200 | WLIM1, GATA type zinc finger transcription factor family protein | -1.669 | -1.791 | -2.887 |
| 3 | 18719 | bx027071 |  |  | -2.206 | -3.116 | -3.319 |
| 3 | 18865 | bx027259 | AT5G48740 | Leucine-rich repeat protein kinase family protein | -2.206 | -2.108 | -2.944 |
| 3 | 18964 | bx027406 | AT4G12420 | Cupredoxin superfamily protein | -0.669 | -2.222 | -2.794 |
| 3 | 18984 | bx027432 | AT5G37478 | TPX2 (targeting protein for Xklp2) protein family | -2.480 | -3.149 | -5.152 |
| 3 | 19042 | bx027508 | AT2G28200 | C2H2-type zinc finger family protein | -1.381 | -2.116 | -2.819 |
| 3 | 19340 | bx027911 | AT5G14740 | CARBONIC ANHYDRASE 18, BETA CARBONIC ANHYDRASE 2, carbonic anhydrase 2 | -2.017 | -3.585 | -2.771 |
| 3 | 19402 | bx027994 | AT4G16447 | unknown protein | -1.737 | -2.517 | -4.339 |
| 3 | 19569 | bx028205 | AT5G59305 | unknown protein | -1.270 | -1.489 | -2.678 |
| 3 | 19769 | bx028466 | AT1G62870 | unknown protein | -1.469 | -1.767 | -2.343 |
| 3 | 19816 | bx028526 | AT3G01500 | ARABIDOPSIS THALIANA SALICYLIC ACID-BINDING PROTEIN 3, BETA CARBONIC ANHYDRASE 1, carbonic anhydrase 1 | -2.662 | -3.686 | -3.435 |
| 3 | 19849 | bx028568 | AT3G52480 | unknown protein | -2.250 | -2.545 | -3.761 |
| 3 | 19993 | bx028774 | AT3G18670 | Ankyrin repeat family protein | -2.274 | -1.505 | -2.536 |
| 3 | 20008 | bx028800 | AT5G60490 | FASCICLIN-like arabinogalactan-protein 12 | -1.988 | -4.848 | -5.450 |
| 3 | 20019 | bx028815 | AT5G50150 | Protein of Unknown Function (DUF239) | -2.663 | -2.418 | -4.070 |
| 3 | 20060 | bx028885 | AT3G49750 | receptor like protein 44 | -1.260 | -2.343 | -2.108 |
| 3 | 20188 | bx029061 | AT1G72160 | Sec14p-like phosphatidylinositol transfer family protein | -1.253 | -1.855 | -2.600 |
| 3 | 20238 | bx029126 | AT5G60210 | ROP interactive partner 5 | -1.777 | -1.914 | -3.449 |
| 3 | 20576 | bx029558 | AT2G17940 | Plant protein of unknown function (DUF827) | -1.717 | -1.381 | -2.893 |
| 3 | 20706 | bx029739 | AT3G63120 | cyclin p1;1 | -2.166 | -2.293 | -3.207 |
| 3 | 20726 | bx029761 |  |  | -1.877 | -1.484 | -2.339 |
| 3 | 20774 | bx029827 | AT2G04480 | unknown protein | -2.775 | -2.798 | -4.350 |
| 3 | 20804 | bx029866 | AT3G52980 | Zinc finger (CCCH-type) family protein / RNA recognition motif (RRM)-containing protein | -2.224 | -2.381 | -2.627 |
| 3 | 20830 | bx029900 | AT3G59010 | pectin methylesterase 61 | -3.936 | -3.578 | -5.596 |
| 3 | 20846 | bx029920 | AT2G04850 | Auxin-responsive family protein | -1.557 | -1.782 | -3.359 |
| 3 | 20891 | bx029979 | AT4G21450 | PapD-like superfamily protein | -1.801 | -1.879 | -2.469 |
| 3 | 21011 | bx030147 | AT5G62710 | Leucine-rich repeat protein kinase family protein | -2.107 | -3.742 | -3.622 |
| 3 | 21025 | bx030162 | AT3G63088 | DEVIL 14, ROTUNDIFOLIA like 14 | -2.048 | -2.720 | -2.994 |
| 3 | 21125 | bx030314 | AT2G04480 | unknown protein | -3.003 | -3.327 | -3.687 |
| 3 | 21134 | bx030327 |  |  | -1.317 | -2.008 | -3.044 |
| 3 | 21147 | bx030340 | AT1G69870 | nitrate transporter 1.7 | -1.915 | -3.603 | -3.147 |
| 3 | 21236 | bx030461 | AT1G75290 | NAD(P)-binding Rossmann-fold superfamily protein | -1.914 | -3.766 | -2.807 |
| 3 | 21313 | bx030571 | AT3G23590 | REF4-related 1 | -2.118 | -2.069 | -3.489 |
| 3 | 21419 | bx030705 | AT1G22400 | ARABIDOPSIS THALIANA UDP-GLUCOSYL TRANSFERASE 85A1, UDP-Glycosyltransferase superfamily protein | -2.064 | -2.896 | -2.636 |
| 3 | 21526 | bx030851 |  |  | -2.548 | -2.946 | -4.428 |
| 3 | 21620 | bx030964 | AT3G21420 | LATERAL BRANCHING OXIDOREDUCTASE 1, 2-oxoglutarate (2OG) and Fe(II)-dependent oxygenase superfamily protein | -2.635 | -2.242 | -3.419 |
| 3 | 21696 | bx031081 | AT2G34930 | disease resistance family protein / LRR family protein | -2.048 | -2.158 | -3.599 |
| 3 | 21770 | bx031190 | AT4G34560 | unknown protein | -2.190 | -1.915 | -3.226 |
| 3 | 21850 | bx031294 | AT2G33030 | receptor like protein 25 | -2.889 | -3.234 | -4.063 |
| 3 | 21852 | bx031296 | AT4G03500 | Ankyrin repeat family protein | -1.159 | -2.584 | -2.096 |
| 3 | 22396 | bx032070 | AT4G27560 | UDP-Glycosyltransferase superfamily protein | -1.041 | -3.148 | -2.067 |
| 3 | 22512 | bx032219 | AT5G58960 | GRAVITROPIC IN THE LIGHT, Plant protein of unknown function (DUF641) | -2.279 | -2.971 | -3.518 |
| 3 | 22517 | bx032226 |  |  | -0.586 | -2.100 | -2.498 |
| 3 | 22571 | bx032296 | AT3G24240 | Leucine-rich repeat receptor-like protein kinase family protein | -2.100 | -2.449 | -2.929 |
| 3 | 22670 | bx032463 | AT4G16370 | oligopeptide transporter | -1.889 | -1.616 | -3.202 |
| 3 | 22949 | bx032869 | AT5G06720 | peroxidase 2 | -1.096 | -1.824 | -2.370 |
| 3 | 22954 | bx032875 | AT1G17020 | senescence-related gene 1 | -1.259 | -2.152 | -2.427 |
| 3 | 23044 | bx033010 | AT1G47270 | tubby like protein 6 | -2.082 | -2.328 | -2.709 |
| 3 | 23207 | bx033235 | AT2G41480 | Peroxidase superfamily protein | -2.104 | -1.305 | -2.629 |
| 3 | 23255 | bx033301 | AT5G40230 | nodulin MtN21 /EamA-like transporter family protein | -1.192 | -2.678 | -1.828 |
| 3 | 23275 | bx033335 | AT4G29080 | indole-3-acetic acid inducible 27, phytochrome-associated protein 2 | -1.377 | -2.062 | -2.528 |
| 3 | 23310 | bx033388 | AT2G32500 | Stress responsive alpha-beta barrel domain protein | 0.706 | -2.425 | -1.684 |
| 3 | 23331 | bx033425 | AT5G10250 | DEFECTIVELY ORGANIZED TRIBUTARIES 3, Phototropic-responsive NPH3 family protein | -0.186 | -5.240 | -3.814 |
| 3 | 23494 | bx033661 | AT5G60490 | FASCICLIN-like arabinogalactan-protein 12 | -1.634 | -2.238 | -2.808 |
| 3 | 23529 | bx033710 | AT5G64600 | O-fucosyltransferase family protein | -1.371 | -2.179 | -3.014 |
| 3 | 23772 | bx034045 |  |  | -0.545 | -2.248 | -2.723 |
| 3 | 23881 | bx034202 | AT1G20090 | Arabidopsis RAC-like 4, RHO-related protein from plants 2 | -2.500 | -2.233 | -4.390 |
| 3 | 23960 | bx034321 | AT1G42970 | glyceraldehyde-3-phosphate dehydrogenase B subunit | -0.415 | -2.900 | -1.850 |
| 3 | 24119 | bx034554 | AT4G31590 | CELLULOSE-SYNTHASE LIKE C5, Cellulose-synthase-like C5 | -1.483 | -1.881 | -2.318 |
| 3 | 24136 | bx034577 | AT5G55580 | Mitochondrial transcription termination factor family protein | -2.599 | -3.154 | -3.876 |
| 3 | 24220 | bx034693 | AT1G11740 | ankyrin repeat family protein | -1.529 | -1.986 | -2.554 |
| 3 | 24381 | bx034941 | AT5G10250 | DEFECTIVELY ORGANIZED TRIBUTARIES 3, Phototropic-responsive NPH3 family protein | -0.872 | -3.061 | -3.218 |
| 3 | 24422 | bx035004 | AT5G23960 | terpene synthase 21 | -0.719 | -2.329 | -1.547 |
| 3 | 24432 | bx035015 | AT5G62580 | ARM repeat superfamily protein | -2.197 | -2.452 | -3.243 |
| 3 | 24800 | bx035530 | AT1G75840 | RHO-LIKE GTP BINDING PROTEIN 4, ARABIDOPSIS THALIANA GERANYLGERANYLATED PROTEIN 3, RAC-like GTP binding protein 5 | -2.522 | -2.232 | -3.756 |
| 3 | 24850 | bx035616 | AT2G41705 | camphor resistance CrcB family protein | -1.709 | -1.997 | -2.816 |
| 3 | 24952 | bx035750 |  |  | -1.932 | -2.082 | -2.713 |
| 3 | 25005 | bx035823 | AT2G47010 | unknown protein | -1.463 | -2.142 | -3.210 |
| 3 | 25073 | bx035923 | AT5G56840 | myb-like transcription factor family protein | -2.280 | -3.473 | -4.921 |
| 3 | 25096 | bx035957 | AT1G28110 | serine carboxypeptidase-like 45 | -1.595 | -2.292 | -3.129 |
| 3 | 25131 | bx036006 | AT1G45616 | receptor like protein 6 | -2.537 | -2.799 | -3.840 |
| 3 | 25256 | bx036183 | AT1G79620 | Leucine-rich repeat protein kinase family protein | -0.895 | -1.729 | -2.604 |
| 3 | 25364 | bx036337 | AT4G35920 | mid1-complementing activity 1, PLAC8 family protein | -2.149 | -2.135 | -3.163 |
| 3 | 25637 | bx036771 | AT5G10250 | DEFECTIVELY ORGANIZED TRIBUTARIES 3, Phototropic-responsive NPH3 family protein | -0.254 | -3.901 | -3.458 |
| 3 | 25719 | bx036900 |  |  | -1.428 | -2.477 | -3.031 |
| 3 | 25803 | bx037019 | AT5G40230 | nodulin MtN21 /EamA-like transporter family protein | -0.776 | -2.582 | -1.998 |
| 3 | 25847 | bx037081 | AT2G33310 | auxin-induced protein 13 | -1.611 | -2.156 | -2.343 |
| 3 | 25996 | bx037326 | AT5G48740 | Leucine-rich repeat protein kinase family protein | -2.277 | -2.345 | -3.197 |
| 3 | 26082 | bx037465 | AT5G10250 | DEFECTIVELY ORGANIZED TRIBUTARIES 3, Phototropic-responsive NPH3 family protein | 0.125 | -5.206 | -4.068 |
| 3 | 26184 | bx037649 | AT4G32295 | unknown protein | -1.757 | -2.078 | -3.187 |
| 3 | 26185 | bx037651 | AT3G01840 | Protein kinase superfamily protein | -1.030 | -2.545 | -3.679 |
| 3 | 26214 | bx037689 | AT1G51440 | alpha/beta-Hydrolases superfamily protein | -2.720 | -2.387 | -3.533 |
| 3 | 26261 | bx037752 | AT1G52510 | alpha/beta-Hydrolases superfamily protein | -0.480 | -2.687 | -3.575 |
| 3 | 26287 | bx037787 | AT1G69080 | Adenine nucleotide alpha hydrolases-like superfamily protein | -3.439 | -3.501 | -4.381 |
| 3 | 26335 | bx037857 | AT4G35160 | O-methyltransferase family protein | -2.193 | -3.374 | -4.863 |
| 3 | 26386 | bx037925 | AT5G05840 | Protein of unknown function (DUF620) | -0.694 | -1.741 | -2.507 |
| 3 | 26459 | bx038017 | AT1G52820 | 2-oxoglutarate (2OG) and Fe(II)-dependent oxygenase superfamily protein | -2.868 | -1.813 | -3.606 |
| 3 | 26475 | bx038044 | AT5G55930 | ARABIDOPSIS THALIANA OLIGOPEPTIDE TRANSPORTER 1, oligopeptide transporter 1 | -2.495 | -2.023 | -2.952 |
| 3 | 26510 | bx038087 | AT2G41250 | Haloacid dehalogenase-like hydrolase (HAD) superfamily protein | -0.391 | -2.401 | -1.588 |
| 3 | 26514 | bx038092 | AT1G59950 | NAD(P)-linked oxidoreductase superfamily protein | 0.177 | -2.932 | -2.752 |
| 3 | 26671 | bx038306 | AT5G54670 | KINESIN-LIKE PROTEIN IN ARABIDOPSIS THALIANA C, kinesin 3 | -1.321 | -1.527 | -2.786 |
| 3 | 26720 | bx038371 | AT3G16360 | HPT phosphotransmitter 4 | -2.058 | -2.380 | -3.031 |
| 3 | 26853 | bx038544 | AT5G25190 | ethylene and salt inducible 3, Integrase-type DNA-binding superfamily protein | -2.206 | -1.389 | -2.652 |
| 3 | 26868 | bx038564 | AT4G24910 | Protein of unknown function (DUF579) | -2.098 | -1.998 | -3.873 |
| 3 | 26893 | bx038602 | AT1G08880 | histone H2A 5, gamma histone variant H2AX, GAMMA H2AX, Histone superfamily protein | -0.491 | -2.626 | -0.993 |
| 3 | 26932 | bx038652 | AT3G04030 | Homeodomain-like superfamily protein | -1.734 | -2.206 | -2.735 |
| 3 | 26945 | bx038668 | AT1G69160 | unknown protein | -2.791 | -2.859 | -4.608 |
| 3 | 26965 | bx038695 | AT3G10810 | zinc finger (C3HC4-type RING finger) family protein | -1.589 | -4.133 | -4.771 |
| 3 | 26990 | bx038725 | AT4G29360 | O-Glycosyl hydrolases family 17 protein | -1.186 | -2.170 | -2.749 |
| 3 | 26992 | bx038729 | AT1G16170 | unknown protein | -1.165 | -1.427 | -2.404 |
| 3 | 26997 | bx038734 | AT1G09390 | GDSL-like Lipase/Acylhydrolase superfamily protein | -1.909 | -2.801 | -2.978 |
| 3 | 27196 | bx038995 | AT2G03760 | ARABIDOPSIS THALIANA SULFOTRANSFERASE 1, sulphotransferase 12 | -1.562 | -2.887 | -2.042 |
| 3 | 27201 | bx039002 | AT1G58170 | Disease resistance-responsive (dirigent-like protein) family protein | -1.420 | -1.513 | -2.410 |
| 3 | 27274 | bx039105 |  |  | -2.150 | -2.300 | -2.553 |
| 3 | 27316 | bx039160 | AT3G62650 | unknown protein | -0.939 | -2.565 | -2.269 |
| 3 | 27355 | bx039207 | AT5G02010 | RHO guanyl-nucleotide exchange factor 7 | -1.790 | -2.412 | -3.877 |
| 3 | 27364 | bx039217 | AT5G54270 | light-harvesting chlorophyll B-binding protein 3 | -1.343 | -2.762 | -2.098 |
| 3 | 27392 | bx039253 | AT3G30530 | basic leucine-zipper 42 | -3.434 | -3.448 | -4.275 |
| 3 | 27423 | bx039291 | AT3G18890 | translocon at the inner envelope membrane of chloroplasts 62, NAD(P)-binding Rossmann-fold superfamily protein | -0.293 | -2.519 | -1.606 |
| 3 | 27481 | bx039366 | AT2G30790 | photosystem II subunit P-2 | -2.332 | -3.737 | -3.216 |
| 3 | 27575 | bx039487 | AT4G15830 | ARM repeat superfamily protein | -1.139 | -2.322 | -2.164 |
| 3 | 27606 | bx039527 | AT1G10200 | WLIM1, GATA type zinc finger transcription factor family protein | -1.551 | -1.424 | -2.311 |
| 3 | 27628 | bx039555 | AT5G48740 | Leucine-rich repeat protein kinase family protein | -2.787 | -2.443 | -3.261 |
| 3 | 27636 | bx039567 | AT2G39200 | MILDEW RESISTANCE LOCUS O 12, Seven transmembrane MLO family protein | -0.798 | -2.329 | -2.477 |
| 3 | 27683 | bx039632 |  |  | 0.664 | -3.050 | -2.850 |
| 3 | 27712 | bx039669 |  |  | -1.847 | -3.145 | -2.737 |
| 3 | 27720 | bx039682 | AT4G14380 | unknown protein | -1.935 | -2.359 | -2.899 |
| 3 | 27745 | bx039713 | AT5G46050 | ARABIDOPSIS THALIANA PEPTIDE TRANSPORTER 3, peptide transporter 3 | -0.388 | -2.468 | -3.045 |
| 3 | 27766 | bx039739 | AT1G11915 | unknown protein | -2.221 | -2.023 | -3.209 |
| 3 | 27822 | bx039819 | AT5G19160 | TRICHOME BIREFRINGENCE-LIKE 11 | -2.415 | -2.941 | -3.228 |
| 3 | 27897 | bx039916 | AT1G61820 | beta glucosidase 46 | -1.707 | -2.826 | -4.049 |
| 3 | 27898 | bx039917 | AT5G10250 | DEFECTIVELY ORGANIZED TRIBUTARIES 3, Phototropic-responsive NPH3 family protein | -0.030 | -3.741 | -3.572 |
| 3 | 27900 | bx039919 | AT1G66480 | plastid movement impaired 2 | -1.275 | -2.582 | -3.718 |
| 3 | 27908 | bx039927 | AT5G57685 | LESS SUSCEPTIBLE TO BSCTV 1, ARABIDOPSIS THALIANA GLUTAMINE DUMPER 3, glutamine dumper 3 | -2.095 | -2.683 | -2.990 |
| 3 | 27923 | bx039947 |  |  | -2.323 | -2.199 | -2.834 |
| 3 | 27930 | bx039955 | AT2G32990 | glycosyl hydrolase 9B8 | -1.601 | -2.443 | -2.706 |
| 3 | 28048 | bx040111 | AT5G48500 | unknown protein | -2.853 | -3.326 | -3.858 |
| 3 | 28106 | bx040179 | AT1G66150 | transmembrane kinase 1 | -1.319 | -1.450 | -2.428 |
| 3 | 28117 | bx040193 | AT4G18570 | Tetratricopeptide repeat (TPR)-like superfamily protein | -1.802 | -3.503 | -4.123 |
| 3 | 28127 | bx040209 |  |  | -0.989 | -2.537 | -3.475 |
| 3 | 28302 | bx040430 |  |  | -2.048 | -4.623 | -5.684 |
| 3 | 28314 | bx040444 | AT5G35630 | GLUTAMINE SYNTHETASE LIKE 1, glutamine synthetase 2 | -0.865 | -2.568 | -1.800 |
| 3 | 28318 | bx040448 | AT1G49430 | LATERAL ROOT DEVELOPMENT 2, long-chain acyl-CoA synthetase 2 | -1.852 | -2.740 | -3.512 |
| 3 | 28321 | bx040451 | AT3G23590 | REF4-related 1 | -2.049 | -1.760 | -2.673 |
| 3 | 28328 | bx040460 | AT1G66150 | transmembrane kinase 1 | -1.983 | -1.942 | -2.470 |
| 3 | 28368 | bx040511 | AT3G29030 | ARABIDOPSIS THALIANA EXPANSIN A5, ARABIDOPSIS THALIANA EXPANSIN 5, expansin A5 | -3.524 | -2.634 | -5.264 |
| 3 | 28369 | bx040512 | AT1G75240 | zinc-finger homeodomain 5, homeobox protein 33 | -1.792 | -3.692 | -3.790 |
| 3 | 28391 | bx040545 | AT3G54950 | patatin-related phospholipase IIIbeta, PATATIN-LIKE PROTEIN 7, patatin-like protein 6 | -0.853 | -2.114 | -3.052 |
| 3 | 28407 | bx040564 | AT3G53960 | Major facilitator superfamily protein | -0.878 | -2.136 | -2.935 |
| 3 | 28420 | bx040580 | AT4G37925 | NADH dehydrogenase-like complex M, subunit NDH-M of NAD(P)H:plastoquinone dehydrogenase complex | -1.257 | -2.705 | -1.885 |
| 3 | 28607 | bx040817 |  |  | -0.969 | -2.193 | -3.028 |
| 3 | 28753 | bx041004 |  |  | -3.055 | -2.324 | -3.575 |
| 3 | 28858 | bx041141 | AT3G01500 | ARABIDOPSIS THALIANA SALICYLIC ACID-BINDING PROTEIN 3, BETA CARBONIC ANHYDRASE 1, carbonic anhydrase 1 | -2.524 | -4.644 | -3.940 |
| 3 | 28974 | bx041286 | AT3G21090 | ATP-binding cassette G15, ABC-2 type transporter family protein | -2.753 | -3.964 | -4.815 |
| 3 | 28987 | bx041302 | AT5G10250 | DEFECTIVELY ORGANIZED TRIBUTARIES 3, Phototropic-responsive NPH3 family protein | -0.475 | -3.586 | -3.393 |
| 3 | 29065 | bx041398 | AT4G03400 | DWARF IN LIGHT 2, Auxin-responsive GH3 family protein | -1.680 | -1.543 | -2.890 |
| 3 | 29126 | bx041472 | AT3G21690 | MATE efflux family protein | -1.260 | -2.858 | -2.239 |
| 3 | 29204 | bx041571 | AT4G38620 | myb domain protein 4 | -2.232 | -4.281 | -4.801 |
| 3 | 29241 | bx041616 | AT5G50130 | NAD(P)-binding Rossmann-fold superfamily protein | -2.031 | -2.412 | -3.601 |
| 3 | 29252 | bx041630 |  |  | -2.075 | -2.157 | -4.054 |
| 3 | 29286 | bx041673 |  |  | -1.510 | -3.317 | -3.490 |
| 3 | 29363 | bx041781 | AT2G38760 | annexin 3 | -1.619 | -2.046 | -2.335 |
| 3 | 29590 | bx042069 | AT5G04700 | Ankyrin repeat family protein | -2.252 | -1.692 | -2.676 |
| 3 | 29679 | bx042188 | AT4G17900 | PLATZ transcription factor family protein | -2.067 | -1.646 | -2.525 |
| 3 | 29690 | bx042201 | AT2G37900 | Major facilitator superfamily protein | -1.930 | -4.122 | -4.185 |
| 3 | 29754 | bx042276 | AT4G28380 | Leucine-rich repeat (LRR) family protein | -3.808 | -2.890 | -5.382 |
| 3 | 29770 | bx042294 | AT5G36110 | cytochrome P450, family 716, subfamily A, polypeptide 1, cytochrome P450, family 716, subfamily A, polypeptide 1 | -2.069 | -1.991 | -2.634 |
| 3 | 29802 | bx042333 | AT5G40250 | RING/U-box superfamily protein | -2.794 | -3.389 | -3.689 |
| 3 | 29804 | bx042335 |  |  | -1.772 | -1.513 | -2.777 |
| 3 | 29867 | bx042414 | AT1G31335 | unknown protein | -1.748 | -2.396 | -2.235 |
| 3 | 29893 | bx042445 | AT5G64600 | O-fucosyltransferase family protein | -1.317 | -2.068 | -3.246 |
| 3 | 29937 | bx042502 |  |  | -3.530 | -3.568 | -4.221 |
| 3 | 29949 | bx042516 | AT3G16520 | UDP-glucosyl transferase 88A1 | -3.068 | -3.937 | -6.334 |
| 3 | 30000 | bx042575 | AT3G01860 | unknown protein | -3.015 | -3.371 | -4.989 |
| 3 | 30006 | bx042582 | AT5G22580 | Stress responsive A/B Barrel Domain | -1.373 | -1.966 | -2.757 |
| 3 | 30007 | bx042583 | AT1G77020 | DNAJ heat shock N-terminal domain-containing protein | -1.421 | -1.961 | -2.852 |
| 3 | 30008 | bx042584 | AT1G70710 | CELLULASE 1, glycosyl hydrolase 9B1 | -2.733 | -3.162 | -4.651 |
| 3 | 30039 | bx042618 | AT5G52450 | MATE efflux family protein | -0.699 | -2.587 | -1.132 |
| 3 | 30089 | bx042680 | AT5G42785 | unknown protein | -2.427 | -2.645 | -2.917 |
| 3 | 30164 | bx042786 | AT4G00165 | Bifunctional inhibitor/lipid-transfer protein/seed storage 2S albumin superfamily protein | -2.391 | -3.684 | -4.401 |
| 3 | 30167 | bx042791 |  |  | -2.202 | -2.264 | -2.606 |
| 3 | 30322 | bx042990 |  |  | -1.299 | -2.698 | -3.077 |
| 3 | 30484 | bx043201 | AT1G14190 | Glucose-methanol-choline (GMC) oxidoreductase family protein | -1.776 | -1.647 | -3.162 |
| 3 | 30649 | bx043420 | AT5G62710 | Leucine-rich repeat protein kinase family protein | -2.111 | -3.786 | -3.708 |
| 3 | 30656 | bx043429 | AT3G18280 | Bifunctional inhibitor/lipid-transfer protein/seed storage 2S albumin superfamily protein | -1.210 | -3.138 | -4.851 |
| 3 | 30708 | bx043490 | AT3G50440 | ARABIDOPSIS THALIANA METHYL ESTERASE 10, methyl esterase 10 | -1.561 | -1.895 | -2.897 |
| 3 | 30744 | bx043536 | AT4G38660 | Pathogenesis-related thaumatin superfamily protein | -1.800 | -1.927 | -2.504 |
| 3 | 30868 | bx043697 |  |  | 1.156 | -2.499 | -2.125 |
| 3 | 30938 | bx043790 | AT3G23590 | REF4-related 1 | -2.430 | -1.991 | -3.023 |
| 3 | 31013 | bx043883 | AT4G13920 | receptor like protein 50 | -2.149 | -2.510 | -3.440 |
| 3 | 31154 | bx044055 | AT1G70890 | MLP-like protein 43 | -2.638 | -2.433 | -3.854 |
| 3 | 31160 | bx044062 | AT4G27430 | COP1-interacting protein 7 | -2.299 | -3.409 | -3.533 |
| 3 | 31295 | bx044250 | AT3G18250 | Putative membrane lipoprotein | -1.260 | -2.465 | -2.654 |
| 3 | 31300 | bx044257 | AT4G35420 | tetraketide alpha-pyrone reductase 1, dihydroflavonol 4-reductase-like1 | -1.303 | -3.209 | -3.678 |
| 3 | 31347 | bx044315 | AT2G36885 | unknown protein | -0.974 | -3.540 | -2.373 |
| 3 | 31412 | bx044399 | AT4G30320 | CAP (Cysteine-rich secretory proteins, Antigen 5, and Pathogenesis-related 1 protein) superfamily protein | -3.232 | -3.534 | -4.331 |
| 3 | 31457 | bx044450 |  |  | -2.351 | -1.150 | -2.628 |
| 3 | 31537 | bx044549 | AT3G42725 | Putative membrane lipoprotein | -1.434 | -3.117 | -2.845 |
| 3 | 31550 | bx044565 | AT1G68060 | microtubule-associated proteins 70-1 | -2.485 | -1.981 | -3.356 |
| 3 | 31558 | bx044576 | AT1G67980 | caffeoyl-CoA 3-O-methyltransferase | -1.651 | -2.889 | -4.552 |
| 3 | 31643 | bx044686 | AT5G50290 | unknown protein | -1.977 | -1.747 | -2.792 |
| 3 | 31645 | bx044688 | AT2G38370 | Plant protein of unknown function (DUF827) | -0.919 | -1.695 | -2.699 |
| 3 | 31707 | bx044764 | AT1G16770 | unknown protein | -0.525 | -2.311 | -2.138 |
| 3 | 31709 | bx044766 | AT1G09540 | ARABIDOPSIS THALIANA MYB DOMAIN PROTEIN 61, myb domain protein 61 | -2.535 | -2.824 | -3.313 |
| 3 | 31742 | bx044808 |  |  | -0.454 | -2.906 | -1.325 |
| 3 | 31759 | bx044825 |  |  | -2.001 | -3.651 | -3.395 |
| 3 | 31773 | bx044842 |  |  | -2.038 | -1.924 | -2.405 |
| 3 | 31786 | bx044858 | AT4G24340 | Phosphorylase superfamily protein | -2.867 | -2.772 | -3.687 |
| 3 | 31902 | bx045006 | AT2G36885 | unknown protein | -0.929 | -3.414 | -2.354 |
| 3 | 32008 | bx045144 | AT1G77920 | bZIP transcription factor family protein | -1.895 | -2.556 | -2.288 |
| 3 | 32040 | bx045191 | AT3G17090 | Protein phosphatase 2C family protein | -1.032 | -2.494 | -1.705 |
| 3 | 32097 | bx045256 | AT3G01500 | ARABIDOPSIS THALIANA SALICYLIC ACID-BINDING PROTEIN 3, BETA CARBONIC ANHYDRASE 1, carbonic anhydrase 1 | -2.594 | -4.660 | -3.374 |
| 3 | 32136 | bx045302 |  |  | -1.138 | -3.843 | -3.216 |
| 3 | 32291 | bx045503 | AT2G26180 | IQ-domain 6 | -1.379 | -2.721 | -2.316 |
| 3 | 32393 | bx045623 | AT5G55340 | MBOAT (membrane bound O-acyl transferase) family protein | -1.428 | -3.036 | -3.823 |
| 3 | 32645 | bx045933 | AT5G46040 | Major facilitator superfamily protein | -0.563 | -2.053 | -2.309 |
| 3 | 32756 | bx046071 | AT3G44730 | ARABIDOPSIS KINESIN-LIKE PROTEIN 1, kinesin-like protein 1 | -2.307 | -2.676 | -3.421 |
| 3 | 32792 | bx046120 | AT4G37760 | squalene epoxidase 3 | -1.698 | -2.306 | -2.978 |
| 3 | 32893 | bx046249 | AT4G14550 | SOLITARY ROOT, indole-3-acetic acid inducible 14 | -1.686 | -3.243 | -4.201 |
| 3 | 32941 | bx046306 | AT5G49800 | Polyketide cyclase/dehydrase and lipid transport superfamily protein | -0.832 | -1.953 | -3.050 |
| 3 | 33019 | bx046404 | AT4G21960 | Peroxidase superfamily protein | -2.302 | -2.969 | -3.344 |
| 3 | 33071 | bx046468 | AT5G25190 | ethylene and salt inducible 3, Integrase-type DNA-binding superfamily protein | -1.202 | -1.378 | -2.490 |
| 3 | 33175 | bx046604 | AT3G59920 | RAB GDP dissociation inhibitor 2 | -2.094 | -1.192 | -2.775 |
| 3 | 33226 | bx046668 | AT3G25400 | unknown protein | -2.303 | -3.218 | -3.125 |
| 3 | 33376 | bx046860 |  |  | -2.620 | -2.814 | -3.371 |
| 3 | 33535 | bx047054 |  |  | -1.653 | -2.527 | -2.216 |
| 3 | 33604 | bx047141 |  |  | -0.843 | -2.446 | -2.912 |
| 3 | 33620 | bx047159 | AT5G11420 | Protein of unknown function, DUF642 | -3.224 | -4.929 | -6.070 |
| 3 | 33709 | bx047265 |  |  | -1.556 | -2.787 | -3.745 |
| 3 | 33775 | bx047338 | AT1G22480 | Cupredoxin superfamily protein | -1.702 | -1.733 | -3.394 |
| 3 | 33815 | bx047392 | AT3G15730 | phospholipase D alpha 1 | -1.549 | -2.516 | -1.965 |
| 3 | 33882 | bx047482 |  |  | -1.827 | -1.932 | -3.015 |
| 3 | 33889 | bx047493 | AT3G13430 | RING/U-box superfamily protein | -1.103 | -3.175 | -4.079 |
| 3 | 33954 | bx047569 | AT5G12330 | LATERAL ROOT PRIMORDIUM 1, Lateral root primordium (LRP) protein-related | -1.454 | -1.973 | -2.782 |
| 3 | 34057 | bx047711 | AT3G25130 | unknown protein | -2.866 | -4.025 | -5.475 |
| 3 | 34315 | bx048030 | AT1G56720 | Protein kinase superfamily protein | -1.326 | -1.824 | -2.584 |
| 3 | 34316 | bx048031 | AT4G15140 | unknown protein | -1.261 | -2.331 | -1.792 |
| 3 | 34362 | bx048084 | AT5G53420 | CCT motif family protein | -1.398 | -1.803 | -2.467 |
| 3 | 34405 | bx048138 | AT3G04730 | indoleacetic acid-induced protein 16 | 0.119 | -3.651 | -3.402 |
| 3 | 34424 | bx048163 | AT5G01310 | APRATAXIN-like | -2.722 | -2.858 | -3.983 |
| 3 | 34466 | bx048219 | AT1G19510 | RADIALIS-LIKE SANT/MYB 4, RAD-like 5 | -0.975 | -2.741 | -3.417 |
| 3 | 34557 | bx048325 | AT1G03820 | unknown protein | -2.835 | -4.071 | -5.987 |
| 3 | 34582 | bx048360 |  |  | -1.751 | -2.174 | -3.014 |
| 3 | 34697 | bx048498 | AT2G35770 | serine carboxypeptidase-like 28 | -0.563 | -1.612 | -2.308 |
| 3 | 34771 | bx048583 | AT1G51940 | protein kinase family protein / peptidoglycan-binding LysM domain-containing protein | -2.318 | -3.056 | -3.775 |
| 3 | 34977 | bx048850 | AT3G49650 | P-loop containing nucleoside triphosphate hydrolases superfamily protein | -1.519 | -2.545 | -3.752 |
| 3 | 35027 | bx048915 | AT2G30933 | Carbohydrate-binding X8 domain superfamily protein | -1.694 | -1.916 | -3.463 |
| 3 | 35219 | bx049151 |  |  | -2.053 | -1.935 | -3.439 |
| 3 | 35224 | bx049157 | AT1G05250 | Peroxidase superfamily protein | -1.328 | -3.254 | -4.175 |
| 3 | 35277 | bx049238 | AT3G22790 | Kinase interacting (KIP1-like) family protein | -1.873 | -1.672 | -2.880 |
| 3 | 35522 | bx049553 | AT1G30690 | Sec14p-like phosphatidylinositol transfer family protein | -1.466 | -2.510 | -2.558 |
| 3 | 35534 | bx049570 |  |  | -1.926 | -2.833 | -4.293 |
| 3 | 35546 | bx049582 |  |  | -1.695 | -1.851 | -2.554 |
| 3 | 35600 | bx049655 | AT1G61800 | ARABIDOPSIS GLUCOSE-6-PHOSPHATE/PHOSPHATE TRANSLOCATOR 2, glucose-6-phosphate/phosphate translocator 2 | -2.447 | -3.903 | -3.268 |
| 3 | 35636 | bx049715 | AT5G35870 | unknown protein | -2.802 | -2.817 | -3.657 |
| 3 | 35660 | bx049745 | AT1G63640 | P-loop nucleoside triphosphate hydrolases superfamily protein with CH (Calponin Homology) domain | -1.501 | -1.689 | -2.383 |
| 3 | 35724 | bx049828 |  |  | -2.647 | -1.588 | -2.952 |
| 3 | 35732 | bx049836 | AT4G27300 | S-locus lectin protein kinase family protein | -2.097 | -1.996 | -3.232 |
| 3 | 35824 | bx049971 | AT5G35670 | IQ-domain 33 | -1.386 | -2.400 | -2.631 |
| 3 | 35872 | bx050032 | AT2G47500 | P-loop nucleoside triphosphate hydrolases superfamily protein with CH (Calponin Homology) domain | -1.839 | -1.742 | -2.333 |
| 3 | 35928 | bx050099 |  |  | -1.945 | -3.019 | -3.492 |
| 3 | 35986 | bx050171 | AT1G26770 | ARABIDOPSIS THALIANA EXPANSIN ALPHA 1.1, ARABIDOPSIS THALIANA EXPANSIN 10, expansin A10 | -3.466 | -2.699 | -5.225 |
| 3 | 36092 | bx050300 | AT3G02885 | GAST1 protein homolog 5 | -2.122 | -2.069 | -2.495 |
| 3 | 36130 | bx050346 |  |  | -2.855 | -3.390 | -3.866 |
| 3 | 36201 | bx050426 | AT3G10080 | RmlC-like cupins superfamily protein | -3.270 | -4.562 | -4.015 |
| 3 | 36205 | bx050430 | AT4G16146 | cAMP-regulated phosphoprotein 19-related protein | -1.752 | -2.668 | -2.227 |
| 3 | 36238 | bx050466 | AT5G01780 | 2-oxoglutarate-dependent dioxygenase family protein | 1.068 | -2.308 | -1.267 |
| 3 | 36545 | bx050850 | AT2G45600 | alpha/beta-Hydrolases superfamily protein | -2.088 | -4.770 | -4.212 |
| 3 | 36551 | bx050858 | AT5G42760 | Leucine carboxyl methyltransferase | -0.402 | -2.539 | -2.128 |
| 3 | 36645 | bx050974 | AT4G31940 | cytochrome P450, family 82, subfamily C, polypeptide 4, cytochrome P450, family 82, subfamily C, polypeptide 4 | -0.644 | -2.928 | -2.994 |
| 3 | 36846 | bx051226 | AT3G44940 | Protein of unknown function (DUF1635) | -2.326 | -2.332 | -3.290 |
| 3 | 37150 | bx051641 | AT5G51460 | Haloacid dehalogenase-like hydrolase (HAD) superfamily protein | -0.704 | -2.718 | -3.539 |
| 3 | 37213 | bx051713 | AT1G21880 | lysm domain GPI-anchored protein 1 precursor | -2.054 | -2.242 | -2.731 |
| 3 | 37332 | bx051856 |  |  | -2.067 | -2.517 | -2.572 |
| 3 | 37716 | bx052339 | AT2G03350 | Protein of unknown function, DUF538 | -1.786 | -1.656 | -2.586 |
| 3 | 37775 | bx052408 |  |  | -3.569 | -5.534 | -5.639 |
| 3 | 37891 | bx052577 | AT5G50150 | Protein of Unknown Function (DUF239) | -2.084 | -2.007 | -3.630 |
| 3 | 37981 | bx052696 | AT4G03500 | Ankyrin repeat family protein | -1.244 | -2.435 | -2.009 |
| 3 | 38007 | bx052734 | AT3G05470 | Actin-binding FH2 (formin homology 2) family protein | -2.489 | -2.265 | -3.189 |
| 3 | 38048 | bx052785 | AT1G10380 | Putative membrane lipoprotein | -2.969 | -4.301 | -4.224 |
| 3 | 38237 | bx053016 | AT3G56290 | unknown protein | -1.219 | -2.634 | -1.661 |
| 3 | 38276 | bx053067 | AT1G04040 | HAD superfamily, subfamily IIIB acid phosphatase | -1.442 | -1.713 | -2.907 |
| 3 | 38363 | bx053184 | AT1G27500 | Tetratricopeptide repeat (TPR)-like superfamily protein | -1.922 | -1.520 | -3.068 |
| 3 | 39025 | bx054040 | AT3G49650 | P-loop containing nucleoside triphosphate hydrolases superfamily protein | -2.154 | -2.765 | -2.607 |
| 3 | 39041 | bx054063 | AT3G51550 | FERONIA, Malectin/receptor-like protein kinase family protein | -1.180 | -2.676 | -3.242 |
| 3 | 39065 | bx054093 | AT1G27620 | HXXXD-type acyl-transferase family protein | -1.944 | -4.204 | -5.875 |
| 3 | 39113 | bx054161 | AT3G62980 | TRANSPORT INHIBITOR RESPONSE 1, F-box/RNI-like superfamily protein | -1.944 | -1.876 | -2.439 |
| 3 | 39147 | bx054213 | AT2G32990 | glycosyl hydrolase 9B8 | -1.553 | -2.353 | -2.607 |
| 3 | 39228 | bx054324 | AT2G33580 | Protein kinase superfamily protein | -1.158 | -2.508 | -3.379 |
| 3 | 39276 | bx054403 | AT3G23590 | REF4-related 1 | -2.134 | -2.199 | -3.084 |
| 3 | 39295 | bx054428 | AT5G48050 | unknown protein | -3.404 | -4.738 | -4.615 |
| 3 | 39325 | bx054473 | AT5G26780 | serine hydroxymethyltransferase 2 | -1.255 | -2.444 | -1.739 |
| 3 | 39335 | bx054487 |  |  | 0.050 | -2.889 | -1.441 |
| 3 | 39480 | bx054682 |  |  | -0.993 | -3.652 | -1.965 |
| 3 | 39501 | bx054709 | AT5G01360 | TRICHOME BIREFRINGENCE-LIKE 3, Plant protein of unknown function (DUF828) | -1.487 | -3.860 | -5.069 |
| 3 | 39512 | bx054725 |  |  | -1.189 | -3.087 | -4.414 |
| 3 | 39628 | bx054896 | AT3G14470 | NB-ARC domain-containing disease resistance protein | -2.795 | -3.456 | -3.856 |
| 3 | 39651 | bx054929 | AT3G05330 | A. THALIANA TAN1, TANGLED, cyclin family | -1.203 | -2.355 | -1.956 |
| 3 | 39695 | bx055005 | AT5G54670 | KINESIN-LIKE PROTEIN IN ARABIDOPSIS THALIANA C, kinesin 3 | -1.332 | -1.480 | -2.779 |
| 3 | 39701 | bx055016 | AT5G48740 | Leucine-rich repeat protein kinase family protein | -2.240 | -2.154 | -2.892 |
| 3 | 39957 | bx055375 | AT3G44050 | P-loop containing nucleoside triphosphate hydrolases superfamily protein | -1.358 | -2.318 | -1.871 |
| 3 | 39982 | bx055406 | AT3G52500 | Eukaryotic aspartyl protease family protein | -1.164 | -2.457 | -2.841 |
| 3 | 40003 | bx055431 | AT3G01220 | homeobox protein 20 | -1.791 | -2.741 | -2.635 |
| 3 | 40011 | bx055439 | AT1G07530 | ARABIDOPSIS THALIANA GRAS (GAI, RGA, SCR) 2, SCARECROW-like 14 | -1.891 | -3.798 | -3.817 |
| 3 | 40063 | bx055514 | ATMG00820 | Reverse transcriptase (RNA-dependent DNA polymerase) | -1.458 | -3.196 | -3.090 |
| 3 | 40255 | bx055751 |  |  | -1.042 | -2.036 | -2.699 |
| 3 | 40557 | bx056116 | AT3G63120 | cyclin p1;1 | -2.234 | -2.691 | -2.735 |
| 3 | 40653 | bx056239 | AT2G06040 | unknown protein | -1.816 | -1.579 | -2.501 |
| 3 | 40699 | bx056294 | AT2G26040 | regulatory components of ABA receptor 14, PYR1-like 2 | -2.246 | -2.543 | -2.851 |
| 3 | 40756 | bx056362 | AT2G21300 | ATP binding microtubule motor family protein | -1.475 | -2.406 | -2.499 |
| 3 | 40782 | bx056394 | AT1G04250 | indole-3-acetic acid inducible 17, AUXIN RESISTANT 3, AUX/IAA transcriptional regulator family protein | -1.126 | -1.887 | -2.598 |
| 3 | 40787 | bx056400 | AT3G57830 | Leucine-rich repeat protein kinase family protein | -3.027 | -1.481 | -3.589 |
| 3 | 40875 | bx056505 | AT4G21980 | AUTOPHAGY-RELATED 8A, AUTOPHAGY 8A, Ubiquitin-like superfamily protein | -0.843 | -2.335 | -2.397 |
| 3 | 40962 | bx056615 | AT3G29575 | ABI five binding protein 3 | 0.005 | -1.913 | -2.350 |
| 3 | 41052 | bx056726 | AT5G60900 | receptor-like protein kinase 1 | -2.298 | -2.338 | -2.776 |
| 3 | 41147 | bx056835 | AT1G46264 | SCHIZORIZA, heat shock transcription factor B4, heat shock transcription factor B4 | -1.659 | -2.119 | -2.337 |
| 3 | 41226 | bx056937 |  |  | -1.190 | -2.009 | -2.751 |
| 3 | 41337 | bx057070 | AT2G01275 | RING/FYVE/PHD zinc finger superfamily protein | -1.631 | -2.327 | -2.084 |
| 3 | 41365 | bx057102 | AT2G30070 | POTASSIUM UPTAKE TRANSPORTER 1, potassium transporter 1 | -2.285 | -2.510 | -2.777 |
| 3 | 41416 | bx057164 | AT5G37790 | Protein kinase superfamily protein | -1.776 | -1.626 | -2.858 |
| 3 | 41445 | bx057203 | AT5G26730 | Fasciclin-like arabinogalactan family protein | -1.548 | -2.784 | -3.554 |
| 3 | 41540 | bx057321 | AT3G24670 | Pectin lyase-like superfamily protein | -2.767 | -4.006 | -4.359 |
| 3 | 41547 | bx057334 | AT3G15550 | unknown protein | -1.351 | -2.666 | -1.924 |
| 3 | 41570 | bx057363 | AT1G18550 | ATP binding microtubule motor family protein | -1.693 | -2.957 | -2.452 |
| 3 | 41630 | bx057435 | AT4G36930 | SPATULA, basic helix-loop-helix (bHLH) DNA-binding superfamily protein | -1.531 | -2.600 | -2.709 |
| 3 | 41680 | bx057491 |  |  | -1.303 | -2.142 | -3.335 |
| 3 | 41740 | bx057563 |  |  | -0.517 | -2.350 | -2.335 |
| 3 | 41761 | bx057587 |  |  | -2.242 | -3.044 | -3.341 |
| 3 | 41889 | bx057746 |  |  | -2.830 | -3.510 | -4.431 |
| 3 | 41897 | bx057754 | AT1G04680 | Pectin lyase-like superfamily protein | -2.730 | -4.392 | -5.265 |
| 3 | 41920 | bx057779 |  |  | -3.087 | -2.567 | -3.892 |
| 3 | 41984 | bx057855 | AT3G01300 | Protein kinase superfamily protein | -0.938 | -2.633 | -2.985 |
| 3 | 42004 | bx057877 | AT1G49320 | unknown seed protein like 1 | -2.107 | -4.182 | -3.767 |
| 3 | 42522 | bx058506 |  |  | -2.524 | -3.297 | -3.016 |
| 3 | 42672 | bx058689 | AT3G10080 | RmlC-like cupins superfamily protein | -2.301 | -2.457 | -3.510 |
| 3 | 42704 | bx058733 | AT1G29200 | O-fucosyltransferase family protein | -1.900 | -1.696 | -2.662 |
| 3 | 42774 | bx058824 |  |  | -2.401 | -3.259 | -3.350 |
| 3 | 42834 | bx058898 | AT1G29195 | unknown protein | -0.579 | -1.848 | -2.727 |
| 3 | 42863 | bx058933 | AT5G58000 | Reticulon family protein | -2.112 | -2.515 | -2.624 |
| 3 | 42950 | bx059042 | AT1G02040 | C2H2-type zinc finger family protein | -2.035 | -2.098 | -3.566 |
| 3 | 43419 | bx059622 | AT5G09220 | amino acid permease 2 | -1.364 | -2.328 | -2.689 |
| 3 | 43525 | bx059754 | AT3G13980 | unknown protein | -3.109 | -2.236 | -3.953 |
| 3 | 43531 | bx059760 | AT5G23730 | REPRESSOR OF UV-B PHOTOMORPHOGENESIS 2, EARLY FLOWERING BY OVEREXPRESSION 2, Transducin/WD40 repeat-like superfamily protein | -0.860 | -2.307 | -2.715 |
| 3 | 43561 | bx059800 | AT2G46300 | Late embryogenesis abundant (LEA) hydroxyproline-rich glycoprotein family | -1.715 | -1.967 | -2.616 |
| 3 | 43572 | bx059815 | AT4G37750 | DRAGON, COMPLEMENTING A PROTEIN KINASE C MUTANT 1, AINTEGUMENTA, Integrase-type DNA-binding superfamily protein | -2.353 | -2.464 | -3.527 |
| 3 | 43580 | bx059824 |  |  | -2.048 | -1.712 | -2.733 |
| 3 | 43704 | bx059976 | AT4G22190 | unknown protein | -1.478 | -1.797 | -3.041 |
| 3 | 43745 | bx060028 |  |  | -2.480 | -1.322 | -3.262 |
| 3 | 43812 | bx060107 | AT5G35630 | GLUTAMINE SYNTHETASE LIKE 1, glutamine synthetase 2 | -0.885 | -2.554 | -1.721 |
| 3 | 43924 | bx060250 |  |  | -2.789 | -3.549 | -3.830 |
| 3 | 43935 | bx060263 |  |  | -1.789 | -2.165 | -2.735 |
| 3 | 44150 | bx060513 | AT1G28110 | serine carboxypeptidase-like 45 | -1.896 | -2.300 | -3.327 |
| 3 | 44196 | bx060569 | ATMG00810 | DNA/RNA polymerases superfamily protein | -1.547 | -2.514 | -2.371 |
| 3 | 44516 | bx060967 | AT3G08490 | unknown protein | -2.803 | -3.464 | -4.233 |
| 3 | 44581 | bx061042 | AT1G06320 | unknown protein | -0.471 | -2.447 | -1.106 |
| 3 | 44668 | bx061148 | AT1G18550 | ATP binding microtubule motor family protein | -1.694 | -3.128 | -2.904 |
| 3 | 44679 | bx061160 |  |  | -2.657 | -2.356 | -3.479 |
| 3 | 44693 | bx061176 | AT5G45970 | RHO-RELATED PROTEIN FROM PLANTS 7, ARABIDOPSIS THALIANA RAC 2, Arabidopsis RAC-like 2, RAC-like 2 | -2.036 | -2.442 | -3.481 |
| 3 | 44702 | bx061187 | AT1G69780 | Homeobox-leucine zipper protein family | -1.701 | -2.435 | -2.450 |
| 3 | 44876 | bx061405 | AT5G64600 | O-fucosyltransferase family protein | -1.569 | -2.483 | -3.390 |
| 3 | 44964 | bx061511 | AT3G18060 | transducin family protein / WD-40 repeat family protein | -2.132 | -2.826 | -3.203 |
| 3 | 45000 | bx061556 |  |  | -2.014 | -3.879 | -3.750 |
| 3 | 45091 | bx061674 | AT2G29125 | DEVIL 13, ROTUNDIFOLIA like 2 | -2.127 | -3.028 | -3.373 |
| 3 | 45127 | bx061715 | AT1G62360 | WALDMEISTER 1, WALDMEISTER, SHOOT MERISTEMLESS, SHOOTLESS, BUMBERSHOOT 1, BUMBERSHOOT, KNOX/ELK homeobox transcription factor | -1.724 | -2.252 | -2.470 |
| 3 | 45258 | bx061880 | AT2G39200 | MILDEW RESISTANCE LOCUS O 12, Seven transmembrane MLO family protein | -1.169 | -2.207 | -2.819 |
| 3 | 45495 | bx062169 |  |  | -0.866 | -2.590 | -1.599 |
| 3 | 45906 | bx062703 |  |  | -1.389 | -2.235 | -2.519 |
| 3 | 46054 | bx062900 |  |  | 0.038 | -2.484 | -2.717 |
| 3 | 46120 | bx062987 |  |  | -1.491 | -1.644 | -2.613 |
| 3 | 46160 | bx063031 |  |  | -0.888 | -2.860 | -3.516 |
| 3 | 46329 | bx063258 |  |  | -1.023 | -2.837 | -1.909 |
| 3 | 46348 | bx063281 | AT2G03720 | morphogenesis of root hair 6, Adenine nucleotide alpha hydrolases-like superfamily protein | -3.453 | -3.718 | -4.636 |
| 3 | 46381 | bx063317 | AT5G64667 | inflorescence deficient in abscission (IDA)-like 2 | -2.199 | -2.003 | -3.013 |
| 3 | 46452 | bx063407 | AT5G33370 | GDSL-like Lipase/Acylhydrolase superfamily protein | -2.264 | -2.789 | -3.269 |
| 3 | 46470 | bx063428 | AT3G20015 | Eukaryotic aspartyl protease family protein | -1.931 | -2.137 | -3.079 |
| 3 | 46561 | bx063540 | AT4G18050 | P-glycoprotein 9, ATP-binding cassette B9, P-glycoprotein 9 | -0.908 | -1.892 | -2.615 |
| 3 | 46607 | bx063592 | AT2G23700 | Protein of unknown function, DUF547 | -1.664 | -1.561 | -2.341 |
| 3 | 46646 | bx063637 | AT3G13445 | TRANSCRIPTION FACTOR IID-1, TATA binding protein 1 | -1.466 | -2.666 | -2.236 |
| 3 | 46713 | bx063716 | AT3G18170 | Glycosyltransferase family 61 protein | -1.547 | -2.430 | -3.156 |
| 3 | 46724 | bx063728 |  |  | -1.565 | -2.680 | -2.353 |
| 3 | 46736 | bx063742 | AT3G03190 | ARABIDOPSIS GLUTATHIONE-S-TRANSFERASE 6, glutathione S-transferase F11 | -1.087 | -1.667 | -2.507 |
| 3 | 46792 | bx063808 | AT3G14170 | Plant protein of unknown function (DUF936) | -1.857 | -4.025 | -4.377 |
| 3 | 46975 | bx064019 |  |  | -2.523 | -2.441 | -4.105 |
| 3 | 47070 | bx064127 |  |  | -1.883 | -2.453 | -2.689 |
| 3 | 47107 | bx064169 | AT2G30395 | ovate family protein 17 | -2.433 | -3.270 | -4.638 |
| 3 | 47227 | bx064304 |  |  | -0.549 | -2.419 | -2.072 |
| 3 | 47352 | bx064446 | AT5G47800 | Phototropic-responsive NPH3 family protein | -2.129 | -2.020 | -3.027 |
| 3 | 47462 | bx064575 | AT3G07880 | SUPERCENTIPEDE1, Immunoglobulin E-set superfamily protein | -3.095 | -3.628 | -3.926 |
| 3 | 47509 | bx064625 | AT1G70280 | NHL domain-containing protein | -1.967 | -2.220 | -3.449 |
| 3 | 47545 | bx064667 |  |  | -1.804 | -2.827 | -2.246 |
| 3 | 47605 | bx064731 | AT1G68795 | CLAVATA3/ESR-RELATED 12 | -2.854 | -4.071 | -4.025 |
| 3 | 47640 | bx064767 | AT3G22410 | Sec14p-like phosphatidylinositol transfer family protein | -2.817 | -4.102 | -4.973 |
| 3 | 47650 | bx064779 | AT3G01500 | ARABIDOPSIS THALIANA SALICYLIC ACID-BINDING PROTEIN 3, BETA CARBONIC ANHYDRASE 1, carbonic anhydrase 1 | -2.134 | -4.508 | -3.467 |
| 3 | 47693 | bx064829 |  |  | -1.519 | -2.499 | -2.048 |
| 3 | 47702 | bx064839 | AT5G22380 | NAC domain containing protein 90 | -0.291 | -1.861 | -2.550 |
| 3 | 47752 | bx064897 | AT5G55830 | Concanavalin A-like lectin protein kinase family protein | -1.503 | -2.800 | -2.214 |
| 3 | 47769 | bx064914 |  |  | -1.238 | -1.520 | -2.658 |
| 3 | 47882 | bx065040 | AT5G55950 | Nucleotide/sugar transporter family protein | -2.028 | -2.252 | -3.768 |
| 3 | 48047 | bx065229 |  |  | -1.638 | -2.153 | -2.966 |
| 3 | 48247 | bx065455 |  |  | -2.301 | -2.598 | -2.900 |
| 3 | 48317 | bx065544 | AT2G25790 | Leucine-rich receptor-like protein kinase family protein | -1.657 | -1.517 | -2.476 |
| 3 | 48461 | bx065738 | AT5G55970 | RING/U-box superfamily protein | -1.215 | -2.486 | -2.803 |
| 3 | 48589 | bx065900 | AT2G42110 | unknown protein | -1.350 | -2.370 | -1.984 |
| 3 | 48609 | bx065921 |  |  | -1.449 | -3.229 | -3.454 |
| 3 | 48725 | bx066076 | AT5G55970 | RING/U-box superfamily protein | -1.656 | -1.590 | -3.136 |
| 3 | 48761 | bx066122 | AT5G53470 | acyl-CoA binding protein 1 | -0.969 | -2.623 | -2.697 |
| 3 | 48973 | bx066390 | AT3G22790 | Kinase interacting (KIP1-like) family protein | -1.916 | -1.654 | -2.945 |
| 3 | 48984 | bx066402 |  |  | -1.453 | -1.546 | -2.372 |
| 3 | 49091 | bx066543 | AT1G12740 | cytochrome P450, family 87, subfamily A, polypeptide 2, cytochrome P450, family 87, subfamily A, polypeptide 2 | -2.204 | -2.638 | -3.223 |
| 3 | 49110 | bx066564 | AT2G32500 | Stress responsive alpha-beta barrel domain protein | -1.098 | -3.652 | -1.876 |
| 3 | 49185 | bx066661 | AT5G38300 | unknown protein | -1.176 | -2.299 | -2.978 |
| 3 | 49256 | bx066742 | AT5G06800 | myb-like HTH transcriptional regulator family protein | -1.697 | -1.515 | -2.431 |
| 3 | 49314 | bx066811 | AT1G08830 | copper/zinc superoxide dismutase 1 | -1.082 | -3.230 | -3.104 |
| 3 | 49343 | bx066845 | AT5G08640 | flavonol synthase 1 | -0.723 | -5.180 | -5.717 |
| 3 | 49529 | bx067059 | AT1G51940 | protein kinase family protein / peptidoglycan-binding LysM domain-containing protein | -1.937 | -2.107 | -2.751 |
| 3 | 49611 | bx067154 | AT5G50150 | Protein of Unknown Function (DUF239) | -1.590 | -1.770 | -2.485 |
| 3 | 49671 | bx067227 |  |  | -1.155 | -1.376 | -2.313 |
| 3 | 49731 | bx067303 |  |  | -0.763 | -2.610 | -2.792 |
| 3 | 49935 | bx067537 | AT5G63410 | Leucine-rich repeat protein kinase family protein | -2.114 | -2.098 | -2.617 |
| 3 | 49980 | bx067589 |  |  | -1.059 | -4.563 | -3.957 |
| 3 | 50112 | bx067741 |  |  | -1.592 | -2.111 | -2.991 |
| 3 | 50321 | bx067980 | AT2G32440 | ARABIDOPSIS ENT-KAURENOIC ACID HYDROXYLASE 2, ent-kaurenoic acid hydroxylase 2 | -1.894 | -2.634 | -3.064 |
| 3 | 50691 | bx068411 | AT3G19800 | Protein of unknown function (DUF177) | -1.753 | -2.566 | -2.187 |
| 3 | 50717 | bx068448 | AT5G19190 | unknown protein | -1.846 | -2.816 | -2.939 |
| 3 | 50796 | bx068555 | AT2G04780 | FASCICLIN-like arabinoogalactan 7 | -1.597 | -1.259 | -2.558 |
| 3 | 51002 | bx068793 | AT3G52110 | unknown protein | -1.506 | -2.710 | -2.700 |
| 3 | 51164 | bx068996 | AT2G02650 | Ribonuclease H-like superfamily protein | -2.461 | -3.693 | -3.736 |
| 3 | 51173 | bx069006 | AT3G50440 | ARABIDOPSIS THALIANA METHYL ESTERASE 10, methyl esterase 10 | -1.726 | -2.271 | -2.432 |
| 3 | 51334 | bx069251 | AT3G07870 | F-box and associated interaction domains-containing protein | -0.931 | -2.750 | -2.281 |
| 3 | 51552 | bx069562 |  |  | -2.118 | -2.774 | -3.126 |
| 3 | 51831 | bx070021 |  |  | -1.784 | -2.542 | -2.311 |
| 3 | 51842 | bx070035 |  |  | -1.807 | -2.870 | -2.813 |
| 3 | 51848 | bx070041 |  |  | -1.924 | -2.543 | -3.137 |
| 3 | 52296 | bx070658 |  |  | -0.812 | -3.512 | -3.301 |
| 3 | 52353 | bx070757 |  |  | -1.737 | -2.886 | -2.219 |
| 3 | 52509 | bx071006 | AT3G61230 | PLIM2c, GATA type zinc finger transcription factor family protein | -3.781 | -4.291 | -4.623 |
| 3 | 52766 | bx071411 |  |  | -1.451 | -2.313 | -2.489 |
| 3 | 52769 | bx071417 |  |  | -1.895 | -2.537 | -2.451 |
| 3 | 52906 | bx071634 |  |  | -1.545 | -2.311 | -2.905 |
| 3 | 53139 | bx071893 | AT4G11950 | Protein of unknown function (DUF1191) | -1.384 | -2.786 | -3.938 |
| 3 | 53167 | bx071925 |  |  | -1.455 | -2.037 | -2.409 |
| 3 | 53226 | bx071994 | AT4G00950 | maternal effect embryo arrest 47, Protein of unknown function (DUF688) | -2.630 | -3.858 | -4.057 |
| 3 | 53260 | bx072030 |  |  | -1.631 | -1.821 | -2.698 |
| 3 | 53311 | bx072088 |  |  | -2.123 | -3.088 | -3.590 |
| 3 | 53838 | bx072711 |  |  | -2.280 | -3.071 | -4.069 |
| 3 | 53869 | bx072751 |  |  | -2.087 | -3.488 | -3.016 |
| 3 | 54099 | bx073010 |  |  | -1.343 | -2.785 | -3.374 |
| 3 | 54184 | bx073104 |  |  | -1.301 | -1.478 | -2.449 |
| 3 | 54317 | bx073251 | AT2G37090 | IRREGULAR XYLEM 9, Nucleotide-diphospho-sugar transferases superfamily protein | -0.771 | -2.405 | -3.566 |
| 3 | 54427 | bx073386 |  |  | -1.663 | -4.450 | -3.753 |
| 3 | 54471 | bx073435 | AT3G30380 | alpha/beta-Hydrolases superfamily protein | -1.837 | -2.180 | -2.779 |
| 3 | 54520 | bx073493 |  |  | -1.422 | -1.511 | -2.596 |
| 3 | 54550 | bx073526 |  |  | -3.164 | -2.768 | -4.210 |
| 3 | 54683 | bx073673 | AT3G61610 | Galactose mutarotase-like superfamily protein | -0.843 | -2.050 | -2.384 |
| 3 | 54869 | bx073868 | AT1G06920 | ARABIDOPSIS THALIANA OVATE FAMILY PROTEIN 4, ovate family protein 4 | -2.401 | -2.617 | -3.076 |
| 3 | 54892 | bx073894 |  |  | -2.662 | -2.540 | -3.336 |
| 3 | 54905 | bx073907 |  |  | -2.592 | -1.672 | -3.776 |
| 3 | 54951 | bx073958 | AT4G37580 | UNUSUAL SUGAR RESPONSE 2, HOOKLESS 1, CONSTITUTIVE PHOTOMORPHOGENIC 3, Acyl-CoA N-acyltransferases (NAT) superfamily protein | -0.640 | -3.151 | -2.327 |
| 3 | 55013 | bx074027 |  |  | -0.844 | -2.212 | -2.717 |
| 3 | 55150 | bx074183 | AT5G40700 | unknown protein | -1.853 | -3.076 | -2.403 |
| 3 | 55227 | bx074297 | AT4G38840 | SAUR-like auxin-responsive protein family | -2.102 | -1.451 | -2.867 |
| 3 | 55279 | bx074363 | AT5G48740 | Leucine-rich repeat protein kinase family protein | -2.180 | -2.270 | -3.074 |
| 3 | 55318 | bx074419 |  |  | -2.665 | -3.000 | -3.244 |
| 3 | 55693 | bx074933 | AT1G26760 | SET domain protein 35 | -0.942 | -1.844 | -2.651 |
| 3 | 55758 | bx075085 | AT1G77390 | TARDY ASYNCHRONOUS MEIOSIS, CYCLIN A1;2 | -0.303 | -2.379 | -1.577 |
| 3 | 55804 | bx075145 | AT5G23870 | Pectinacetylesterase family protein | -1.255 | -1.998 | -3.347 |
| 3 | 55883 | bx075243 |  |  | -1.257 | -2.366 | -1.636 |
| 3 | 55906 | bx075270 |  |  | -2.074 | -2.579 | -3.095 |
| 3 | 55957 | bx075350 | AT4G33330 | glucuronic acid substitution of xylan 2, plant glycogenin-like starch initiation protein 3 | -1.898 | -2.494 | -2.784 |
| 3 | 56047 | bx075508 |  |  | -2.308 | -1.987 | -2.703 |
| 3 | 56128 | bx075617 |  |  | -2.276 | -2.273 | -3.501 |
| 3 | 56177 | bx075673 |  |  | -1.425 | -2.243 | -3.358 |
| 3 | 56187 | bx075685 | AT5G05690 | DWARF 3, CYTOCHROME P450 90A1, CONSTITUTIVE PHOTOMORPHOGENIC DWARF, CABBAGE 3, Cytochrome P450 superfamily protein | -0.715 | -1.763 | -2.781 |
| 3 | 56312 | bx075849 | AT3G02750 | Protein phosphatase 2C family protein | -1.427 | -2.444 | -2.125 |
| 3 | 56379 | bx075957 |  |  | -1.725 | -2.602 | -2.819 |
| 3 | 56411 | bx075992 | AT1G05420 | ARABIDOPSIS THALIANA OVATE FAMILY PROTEIN 12, ovate family protein 12 | -1.882 | -2.101 | -3.552 |
| 3 | 56417 | bx076000 |  |  | -2.200 | -2.102 | -3.782 |
| 3 | 56598 | bx076210 |  |  | -1.614 | -2.347 | -3.139 |
| 3 | 56674 | bx076301 | AT5G49360 | beta-xylosidase 1 | -1.651 | -1.786 | -2.430 |
| 3 | 56681 | bx076309 | AT2G22670 | indoleacetic acid-induced protein 8 | -1.510 | -2.216 | -2.526 |
| 3 | 56740 | bx076375 |  |  | -1.844 | -2.206 | -3.327 |
| 3 | 56914 | bx076562 | AT4G03965 | RING/U-box superfamily protein | -1.674 | -1.749 | -2.808 |
| 3 | 56984 | bx076636 |  |  | -1.134 | -2.048 | -3.239 |
| 3 | 57007 | bx076661 |  |  | -0.220 | -1.864 | -2.531 |
| 3 | 57058 | bx076720 | AT3G25855 | Copper transport protein family | -0.011 | -3.192 | -0.756 |
| 3 | 57127 | bx076809 | AT2G41705 | camphor resistance CrcB family protein | -1.744 | -1.945 | -2.708 |
| 3 | 57522 | bx077327 |  |  | -1.684 | -2.256 | -2.739 |
| 3 | 58020 | bx077945 | AT3G52290 | IQ-domain 3 | -1.464 | -2.376 | -3.665 |
| 3 | 58141 | bx078078 | AT5G38450 | cytochrome P450, family 735, subfamily A, polypeptide 1, cytochrome P450, family 735, subfamily A, polypeptide 1 | -1.253 | -1.912 | -2.451 |
| 3 | 58167 | bx078107 | AT1G52343 | unknown protein | -1.749 | -2.253 | -3.086 |
| 3 | 58246 | bx078193 |  |  | -1.406 | -2.381 | -2.232 |
| 3 | 58383 | bx078350 | AT1G30860 | RING/U-box superfamily protein | -1.610 | -2.505 | -2.051 |
| 3 | 58572 | bx078568 | AT2G27480 | Calcium-binding EF-hand family protein | -1.128 | -2.438 | -2.006 |
| 3 | 58608 | bx078613 | AT5G23870 | Pectinacetylesterase family protein | -1.467 | -2.539 | -3.086 |
| 3 | 58631 | bx078642 |  |  | -1.270 | -1.678 | -2.554 |
| 3 | 58802 | bx078855 | AT1G09450 | Haspin-related gene, Protein kinase superfamily protein | -1.400 | -3.550 | -2.393 |
| 3 | 58921 | bx078994 | AT1G30690 | Sec14p-like phosphatidylinositol transfer family protein | -1.466 | -2.597 | -2.788 |
| 3 | 59112 | bx079223 | AT5G10150 | Domain of unknown function (DUF966) | -3.302 | -3.143 | -3.864 |
| 3 | 59246 | bx079373 | AT3G01860 | unknown protein | -1.827 | -1.025 | -2.332 |
| 3 | 59251 | bx079379 | AT2G47010 | unknown protein | -1.880 | -2.507 | -3.168 |
| 3 | 59293 | bx079433 |  |  | -1.828 | -2.347 | -3.422 |
| 3 | 59482 | bx079649 | AT2G42290 | Leucine-rich repeat protein kinase family protein | -1.787 | -1.341 | -2.859 |
| 3 | 59548 | bx079732 |  |  | -1.989 | -2.369 | -3.980 |
| 3 | 59665 | bx079864 |  |  | -1.676 | -2.075 | -2.316 |
| 3 | 59722 | bx079936 |  |  | -0.694 | -2.405 | -2.472 |
| 3 | 59790 | bx080019 |  |  | -1.906 | -2.696 | -2.563 |
| 3 | 59832 | bx080079 | AT4G38050 | Xanthine/uracil permease family protein | -2.012 | -1.389 | -2.486 |
| 3 | 59950 | bx080225 | AT1G54200 | unknown protein | -2.040 | -2.194 | -3.423 |
| 3 | 59974 | bx080261 |  |  | -1.585 | -3.188 | -3.387 |
| 3 | 60071 | bx080390 |  |  | -1.558 | -1.331 | -2.603 |
| 3 | 60167 | bx080517 | AT1G65450 | HXXXD-type acyl-transferase family protein | -2.357 | -2.275 | -2.723 |
| 3 | 60202 | bx080559 | AT1G25270 | nodulin MtN21 /EamA-like transporter family protein | -1.975 | -5.217 | -4.127 |
| 3 | 60337 | bx080737 |  |  | -2.121 | -3.006 | -3.501 |
| 3 | 60362 | bx080767 | AT5G03760 | RESISTANT TO AGROBACTERIUM TRANSFORMATION 4, CELLULOSE SYNTHASE LIKE A9, Nucleotide-diphospho-sugar transferases superfamily protein | -1.867 | -2.110 | -3.525 |
| 3 | 60367 | bx080774 | AT5G50130 | NAD(P)-binding Rossmann-fold superfamily protein | -2.022 | -2.408 | -3.700 |
| 3 | 60373 | bx080780 | AT1G17020 | senescence-related gene 1 | -2.725 | -3.794 | -4.730 |
| 3 | 60511 | bx080956 |  |  | -2.147 | -1.546 | -2.710 |
| 3 | 60580 | bx081044 | AT3G44050 | P-loop containing nucleoside triphosphate hydrolases superfamily protein | -1.522 | -2.676 | -2.162 |
| 3 | 60589 | bx081053 | AT4G24670 | tryptophan aminotransferase related 2 | -1.499 | -2.088 | -2.992 |
| 3 | 60605 | bx081075 | AT1G12570 | Glucose-methanol-choline (GMC) oxidoreductase family protein | -1.593 | -2.750 | -3.301 |
| 3 | 60654 | bx081139 | AT1G29380 | Carbohydrate-binding X8 domain superfamily protein | -2.765 | -3.623 | -4.402 |
| 3 | 60671 | bx081157 |  |  | -3.829 | -3.901 | -5.191 |
| 3 | 60794 | bx081300 | AT4G22570 | adenine phosphoribosyl transferase 3 | -1.931 | -2.012 | -2.466 |
| 3 | 60801 | bx081309 | AT3G45650 | nitrate excretion transporter1 | -1.175 | -1.663 | -2.600 |
| 3 | 60852 | bx081373 | AT3G06145 | unknown protein | -1.362 | -1.939 | -2.473 |
| 3 | 60868 | bx081395 |  |  | -1.266 | -2.689 | -2.335 |
| 3 | 60893 | bx081428 | AT4G00820 | IQ-domain 17 | -1.098 | -1.786 | -2.952 |
| 3 | 60923 | bx081468 | AT4G33330 | glucuronic acid substitution of xylan 2, plant glycogenin-like starch initiation protein 3 | -1.780 | -1.909 | -3.582 |
| 3 | 60948 | bx081510 | AT2G44300 | Bifunctional inhibitor/lipid-transfer protein/seed storage 2S albumin superfamily protein | -1.992 | -1.691 | -3.049 |
| 3 | 60958 | bx081522 | AT5G22860 | Serine carboxypeptidase S28 family protein | -1.564 | -5.170 | -5.163 |
| 3 | 61030 | bx081607 |  |  | -3.558 | -5.102 | -5.146 |
| 3 | 61056 | bx081636 |  |  | -2.155 | -1.707 | -2.508 |
| 3 | 61093 | bx081678 | AT3G22540 | Protein of unknown function (DUF1677) | -2.463 | -3.082 | -3.579 |
| 3 | 61119 | bx081709 | AT3G23590 | REF4-related 1 | -2.152 | -1.765 | -2.472 |
| 3 | 61238 | bx081877 |  |  | -1.838 | -2.430 | -3.589 |
| 3 | 61274 | bx081929 | AT5G19820 | embryo defective 2734, ARM repeat superfamily protein | -1.540 | -2.399 | -3.069 |
| 3 | 61275 | bx081930 | AT3G23590 | REF4-related 1 | -2.021 | -1.175 | -2.721 |
| 3 | 61308 | bx081982 | AT5G48900 | Pectin lyase-like superfamily protein | -2.427 | -3.890 | -5.412 |
| 3 | 61309 | bx081983 |  |  | 0.014 | -2.340 | -2.304 |
| 3 | 61344 | bx082037 | AT5G52100 | chlororespiration reduction 1, Dihydrodipicolinate reductase, bacterial/plant | -1.595 | -2.135 | -2.319 |
| 3 | 61351 | bx082055 | AT5G38070 | RING/FYVE/PHD zinc finger superfamily protein | -0.896 | -2.118 | -2.449 |
| 3 | 61525 | bx082331 | AT1G75090 | DNA glycosylase superfamily protein | -1.921 | -2.610 | -3.002 |
| 3 | 61566 | bx082404 |  |  | -2.100 | -2.800 | -3.421 |
| 3 | 61614 | bx082474 | AT4G15830 | ARM repeat superfamily protein | -0.889 | -2.324 | -1.714 |
| 4 | 37 | bx000066 |  |  | 0.356 | 1.141 | -3.223 |
| 4 | 261 | bx000472 | AT2G45290 | Transketolase | 1.476 | 1.079 | -2.408 |
| 4 | 562 | bx000993 | AT5G20610 | unknown protein | -0.618 | 1.560 | -2.441 |
| 4 | 614 | bx001087 | AT5G11890 | EMBRYO DEFECTIVE 3135, unknown protein | -0.440 | -0.339 | -3.596 |
| 4 | 880 | bx001531 | AT2G14890 | arabinogalactan protein 9 | -1.053 | -1.427 | -3.815 |
| 4 | 921 | bx001588 | AT3G43190 | ARABIDOPSIS THALIANA SUCROSE SYNTHASE 4, sucrose synthase 4 | -1.211 | -2.261 | -4.799 |
| 4 | 933 | bx001609 | AT5G55860 | Plant protein of unknown function (DUF827) | -1.043 | -1.245 | -4.207 |
| 4 | 1041 | bx001787 | AT1G11260 | sugar transporter 1 | -0.251 | -0.395 | -2.768 |
| 4 | 1110 | bx001898 | AT4G21350 | plant U-box 8 | -1.675 | -2.499 | -4.779 |
| 4 | 1168 | bx001992 | AT4G12350 | myb domain protein 42 | -1.674 | -2.265 | -4.901 |
| 4 | 1267 | bx002144 | AT2G47360 | unknown protein | -1.978 | -2.061 | -4.749 |
| 4 | 1282 | bx002167 | AT5G17420 | MURUS 10, IRREGULAR XYLEM 3, CELLULOSE SYNTHASE CATALYTIC SUBUNIT 7, Cellulose synthase family protein | -1.112 | -1.617 | -4.411 |
| 4 | 1362 | bx002296 | AT1G75680 | glycosyl hydrolase 9B7 | -1.199 | -1.346 | -4.197 |
| 4 | 1458 | bx002430 | AT3G61640 | arabinogalactan protein 20 | -1.610 | -2.206 | -5.206 |
| 4 | 1535 | bx002551 | AT2G30340 | LOB domain-containing protein 13 | -1.403 | -1.057 | -2.659 |
| 4 | 1561 | bx002588 | AT3G53260 | phenylalanine ammonia-lyase 2 | -1.328 | -1.383 | -3.369 |
| 4 | 1765 | bx002912 |  |  | -2.276 | -0.596 | -3.239 |
| 4 | 1890 | bx003093 | AT3G22400 | Arabidopsis thaliana lipoxygenase 5, PLAT/LH2 domain-containing lipoxygenase family protein | 0.219 | -0.163 | -3.279 |
| 4 | 1916 | bx003132 | AT4G32340 | Tetratricopeptide repeat (TPR)-like superfamily protein | -0.769 | -1.622 | -3.222 |
| 4 | 1994 | bx003258 |  |  | -0.918 | -0.799 | -2.888 |
| 4 | 2071 | bx003373 | AT4G28500 | SECONDARY WALL-ASSOCIATED NAC DOMAIN PROTEIN 2, NAC domain containing protein 73 | -1.566 | -0.690 | -3.109 |
| 4 | 2284 | bx003694 |  |  | 1.029 | 0.290 | -2.460 |
| 4 | 2407 | bx003872 |  |  | -1.193 | -1.474 | -2.888 |
| 4 | 2446 | bx003930 | AT2G30340 | LOB domain-containing protein 13 | -1.970 | -0.774 | -2.583 |
| 4 | 2596 | bx004161 | AT1G66920 | Protein kinase superfamily protein | -0.064 | -0.338 | -2.427 |
| 4 | 2860 | bx004549 | AT5G24910 | EUI-like p450 A1, cytochrome P450, family 714, subfamily A, polypeptide 1 | -1.811 | -2.199 | -5.730 |
| 4 | 3015 | bx004786 | AT1G27440 | Exostosin family protein | -1.370 | -1.403 | -5.062 |
| 4 | 3063 | bx004853 | AT1G02180 | ferredoxin-related | -1.148 | -0.968 | -3.176 |
| 4 | 3328 | bx005238 |  |  | -0.024 | -2.775 | -4.451 |
| 4 | 3378 | bx005305 |  |  | -1.649 | -1.982 | -5.392 |
| 4 | 3540 | bx005554 | AT1G71695 | Peroxidase superfamily protein | 0.031 | -1.201 | -4.250 |
| 4 | 3838 | bx005979 | AT4G33330 | glucuronic acid substitution of xylan 2, plant glycogenin-like starch initiation protein 3 | -1.060 | -1.591 | -3.398 |
| 4 | 3841 | bx005983 | AT5G18460 | Protein of Unknown Function (DUF239) | -0.882 | -0.822 | -2.845 |
| 4 | 3901 | bx006075 | AT3G51895 | sulfate transporter 3;1 | -2.085 | -3.528 | -6.367 |
| 4 | 4179 | bx006476 | AT2G29130 | laccase 2 | -0.894 | -2.309 | -5.267 |
| 4 | 4246 | bx006570 | AT5G14260 | Rubisco methyltransferase family protein | -0.947 | -1.299 | -2.876 |
| 4 | 4582 | bx007065 | AT5G15630 | IRREGULAR XYLEM 6, COBRA-LIKE4, COBRA-like extracellular glycosyl-phosphatidyl inositol-anchored protein family | -1.347 | -1.656 | -5.848 |
| 4 | 4619 | bx007126 | AT3G19310 | PLC-like phosphodiesterases superfamily protein | -0.864 | -0.797 | -4.200 |
| 4 | 4853 | bx007461 | AT2G38080 | LACCASE 4, IRREGULAR XYLEM 12, ARABIDOPSIS LACCASE-LIKE MULTICOPPER OXIDASE 4, Laccase/Diphenol oxidase family protein | -1.827 | -1.798 | -5.354 |
| 4 | 5088 | bx007798 | AT5G42800 | dihydroflavonol 4-reductase | -1.253 | -1.194 | -3.376 |
| 4 | 5122 | bx007852 | AT2G45850 | AT hook motif DNA-binding family protein | -0.589 | -0.944 | -2.535 |
| 4 | 5179 | bx007926 |  |  | -0.720 | -1.133 | -3.443 |
| 4 | 5281 | bx008063 | AT1G29200 | O-fucosyltransferase family protein | -1.258 | -1.773 | -5.475 |
| 4 | 5336 | bx008149 | AT1G24620 | EF hand calcium-binding protein family | -1.836 | -1.296 | -3.699 |
| 4 | 5411 | bx008252 | AT5G15490 | UDP-glucose dehydrogenase 3, UDP-glucose 6-dehydrogenase family protein | -0.227 | -0.860 | -3.220 |
| 4 | 5479 | bx008359 |  |  | -0.384 | -1.765 | -2.941 |
| 4 | 5680 | bx008644 | AT1G17030 | unknown protein | -1.196 | -2.005 | -4.319 |
| 4 | 5724 | bx008713 | AT5G45910 | GDSL-like Lipase/Acylhydrolase superfamily protein | -1.045 | -1.304 | -2.753 |
| 4 | 5810 | bx008826 | AT5G47530 | Auxin-responsive family protein | -1.130 | -2.036 | -4.601 |
| 4 | 5855 | bx008887 | AT5G55950 | Nucleotide/sugar transporter family protein | -1.731 | -3.277 | -6.014 |
| 4 | 5995 | bx009065 |  |  | -2.246 | -0.071 | -2.852 |
| 4 | 6054 | bx009140 | AT5G60020 | laccase 17 | -2.559 | -0.421 | -2.500 |
| 4 | 6075 | bx009169 | AT2G46770 | NAC SECONDARY WALL THICKENING PROMOTING FACTOR1, Arabidopsis NAC domain containing protein 43, NAC (No Apical Meristem) domain transcriptional regulator superfamily protein | 1.116 | -2.460 | -2.676 |
| 4 | 6123 | bx009240 | AT1G68200 | Zinc finger C-x8-C-x5-C-x3-H type family protein | -1.194 | -2.351 | -5.094 |
| 4 | 6248 | bx009417 | AT3G18660 | glucuronic acid substitution of xylan 1, plant glycogenin-like starch initiation protein 1 | -2.134 | -1.454 | -5.638 |
| 4 | 6331 | bx009525 | AT3G13430 | RING/U-box superfamily protein | -2.492 | -2.208 | -4.802 |
| 4 | 6359 | bx009574 | AT5G05390 | laccase 12 | -0.562 | -1.672 | -4.262 |
| 4 | 6389 | bx009616 | AT5G60020 | laccase 17 | -1.121 | -2.528 | -6.017 |
| 4 | 6414 | bx009655 |  |  | -1.744 | -2.913 | -5.385 |
| 4 | 6744 | bx010134 | AT1G03470 | Kinase interacting (KIP1-like) family protein | -1.345 | -2.412 | -4.779 |
| 4 | 6832 | bx010240 | AT5G60020 | laccase 17 | -3.022 | -1.006 | -6.003 |
| 4 | 6903 | bx010352 | AT5G02070 | Protein kinase family protein | 1.767 | 1.106 | -2.547 |
| 4 | 7138 | bx010695 | AT4G32050 | neurochondrin family protein | -0.148 | 1.832 | -4.270 |
| 4 | 7357 | bx010986 | AT5G07620 | Protein kinase superfamily protein | -0.889 | -1.829 | -3.438 |
| 4 | 7367 | bx010997 | AT3G51325 | RING/U-box superfamily protein | -1.544 | -1.840 | -4.528 |
| 4 | 7775 | bx011584 | AT3G49260 | IQ-domain 21 | -0.852 | -1.367 | -4.574 |
| 4 | 7786 | bx011605 | AT1G71070 | Core-2/I-branching beta-1,6-N-acetylglucosaminyltransferase family protein | -1.153 | -1.783 | -5.447 |
| 4 | 7819 | bx011664 | AT2G26250 | FIDDLEHEAD, 3-ketoacyl-CoA synthase 10 | -1.792 | 0.148 | -2.325 |
| 4 | 7873 | bx011739 | AT1G60010 | unknown protein | -1.034 | -1.341 | -2.538 |
| 4 | 8001 | bx011906 | AT3G07490 | calmodulin-like 3, ARF-GAP domain 11 | -0.500 | -0.834 | -2.422 |
| 4 | 8154 | bx012120 | AT5G61340 | unknown protein | -1.611 | -1.800 | -4.440 |
| 4 | 8204 | bx012182 |  |  | -2.199 | 0.625 | -3.409 |
| 4 | 8344 | bx012374 | AT1G22710 | SUCROSE TRANSPORTER 1, ARABIDOPSIS THALIANA SUCROSE-PROTON SYMPORTER 2, sucrose-proton symporter 2 | -0.826 | -1.542 | -2.591 |
| 4 | 8353 | bx012389 | AT3G07040 | RESISTANCE TO PSEUDOMONAS SYRINGAE 3, RESISTANCE TO P. SYRINGAE PV MACULICOLA 1, NB-ARC domain-containing disease resistance protein | 0.062 | -1.528 | -2.678 |
| 4 | 8539 | bx012666 | AT1G09415 | NIM1-interacting 3 | -0.135 | -0.501 | -2.730 |
| 4 | 8621 | bx012780 | AT5G26330 | Cupredoxin superfamily protein | -0.833 | -1.211 | -3.412 |
| 4 | 8816 | bx013048 | AT3G62020 | germin-like protein 10 | -0.966 | -1.047 | -4.020 |
| 4 | 8884 | bx013146 | AT4G02890 | Ubiquitin family protein | -0.042 | -0.137 | -2.702 |
| 4 | 8947 | bx013239 | AT1G22710 | SUCROSE TRANSPORTER 1, ARABIDOPSIS THALIANA SUCROSE-PROTON SYMPORTER 2, sucrose-proton symporter 2 | -0.831 | -1.304 | -2.408 |
| 4 | 9017 | bx013345 | AT2G36910 | P-GLYCOPROTEIN 1, ARABIDOPSIS THALIANA P GLYCOPROTEIN1, ATP-binding cassette B1, ATP binding cassette subfamily B1 | -0.931 | -1.004 | -2.562 |
| 4 | 9030 | bx013364 | AT5G48930 | hydroxycinnamoyl-CoA shikimate/quinate hydroxycinnamoyl transferase | -0.364 | -0.225 | -2.342 |
| 4 | 9398 | bx013911 |  |  | -1.507 | -1.817 | -4.007 |
| 4 | 9555 | bx014126 | AT2G37040 | PHE ammonia lyase 1 | -0.727 | -1.326 | -2.634 |
| 4 | 10037 | bx014806 | AT5G55860 | Plant protein of unknown function (DUF827) | -1.212 | -1.099 | -4.361 |
| 4 | 10864 | bx016134 | AT4G08900 | arginine amidohydrolase 1, arginase | -0.338 | -0.157 | -3.136 |
| 4 | 13078 | bx019527 |  |  | -2.253 | -0.557 | -3.265 |
| 4 | 13389 | bx019955 | AT3G21240 | 4-coumarate:CoA ligase 2 | -0.698 | -1.041 | -2.452 |
| 4 | 14077 | bx020852 | AT5G03710 | unknown protein | -1.053 | -1.121 | -2.440 |
| 4 | 14369 | bx021242 | AT1G22480 | Cupredoxin superfamily protein | -1.156 | -1.357 | -4.437 |
| 4 | 14693 | bx021669 | AT5G17600 | RING/U-box superfamily protein | -1.124 | -0.896 | -2.457 |
| 4 | 15027 | bx022109 | AT2G32520 | alpha/beta-Hydrolases superfamily protein | -1.349 | -1.584 | -4.057 |
| 4 | 15504 | bx022729 | AT3G07510 | unknown protein | -1.027 | -3.052 | -5.020 |
| 4 | 15596 | bx022847 | AT4G21740 | unknown protein | -1.841 | -1.746 | -3.569 |
| 4 | 15805 | bx023139 | AT2G28315 | Nucleotide/sugar transporter family protein | -1.412 | -1.590 | -5.717 |
| 4 | 15849 | bx023195 | AT2G38080 | LACCASE 4, IRREGULAR XYLEM 12, ARABIDOPSIS LACCASE-LIKE MULTICOPPER OXIDASE 4, Laccase/Diphenol oxidase family protein | -1.130 | -1.468 | -5.278 |
| 4 | 16176 | bx023645 | AT1G47410 | unknown protein | -1.341 | -1.287 | -5.058 |
| 4 | 16181 | bx023655 | AT5G54670 | KINESIN-LIKE PROTEIN IN ARABIDOPSIS THALIANA C, kinesin 3 | -1.358 | -1.535 | -3.138 |
| 4 | 16765 | bx024478 | AT1G14700 | purple acid phosphatase 3 | -1.531 | 0.305 | -3.473 |
| 4 | 16766 | bx024479 | AT2G20562 | unknown protein | -0.562 | -0.353 | -2.347 |
| 4 | 16837 | bx024567 |  |  | -0.141 | -0.829 | -2.667 |
| 4 | 16858 | bx024592 | AT4G25640 | FLOWER FLAVONOID TRANSPORTER, detoxifying efflux carrier 35 | 1.527 | -0.917 | -2.874 |
| 4 | 16880 | bx024617 |  |  | -0.362 | -0.851 | -2.435 |
| 4 | 17315 | bx025175 | AT5G67210 | IRX15-LIKE, Protein of unknown function (DUF579) | -1.381 | -1.787 | -6.142 |
| 4 | 17331 | bx025202 | AT5G40230 | nodulin MtN21 /EamA-like transporter family protein | -1.729 | -1.434 | -3.372 |
| 4 | 17356 | bx025237 | AT2G36570 | Leucine-rich repeat protein kinase family protein | -0.735 | -1.611 | -3.369 |
| 4 | 17631 | bx025624 | AT2G33850 | unknown protein | -1.229 | -1.122 | -2.354 |
| 4 | 17703 | bx025725 | AT4G23690 | Disease resistance-responsive (dirigent-like protein) family protein | -1.224 | -2.662 | -5.357 |
| 4 | 17723 | bx025747 | AT5G44030 | IRREGULAR XYLEM 5, cellulose synthase A4 | -1.560 | -2.000 | -4.439 |
| 4 | 17967 | bx026068 | AT3G27960 | Tetratricopeptide repeat (TPR)-like superfamily protein | -0.496 | -0.960 | -2.704 |
| 4 | 18086 | bx026227 | AT1G72520 | Arabidopsis thaliana lipoxygenase 4, PLAT/LH2 domain-containing lipoxygenase family protein | 0.832 | -0.517 | -2.481 |
| 4 | 18667 | bx026994 | AT4G10955 | alpha/beta-Hydrolases superfamily protein | -0.703 | -1.028 | -2.369 |
| 4 | 18981 | bx027426 | AT5G41330 | BTB/POZ domain with WD40/YVTN repeat-like protein | -1.134 | -0.815 | -2.613 |
| 4 | 19230 | bx027765 | AT5G60490 | FASCICLIN-like arabinogalactan-protein 12 | -2.336 | -2.417 | -6.810 |
| 4 | 19382 | bx027966 | AT5G16490 | ROP-interactive CRIB motif-containing protein 4 | -2.025 | -2.367 | -6.686 |
| 4 | 19446 | bx028053 | AT2G26250 | FIDDLEHEAD, 3-ketoacyl-CoA synthase 10 | -0.406 | -0.323 | -2.793 |
| 4 | 19469 | bx028079 | AT5G03760 | RESISTANT TO AGROBACTERIUM TRANSFORMATION 4, CELLULOSE SYNTHASE LIKE A9, Nucleotide-diphospho-sugar transferases superfamily protein | -1.808 | -1.895 | -4.426 |
| 4 | 19828 | bx028542 | AT2G36880 | methionine adenosyltransferase 3 | 0.050 | -1.326 | -2.512 |
| 4 | 19868 | bx028590 | AT1G19300 | PARVUS, GAOLAOZHUANGREN 1, GALACTURONOSYLTRANSFERASE-LIKE 1, Nucleotide-diphospho-sugar transferases superfamily protein | -1.028 | -2.245 | -5.744 |
| 4 | 19963 | bx028726 | AT5G09520 | Pro-Glu-Leu\|Ile\|Val-Pro-Lys 2, hydroxyproline-rich glycoprotein family protein | -1.254 | -1.241 | -2.821 |
| 4 | 19983 | bx028758 | AT2G26250 | FIDDLEHEAD, 3-ketoacyl-CoA synthase 10 | -0.361 | -0.399 | -2.433 |
| 4 | 20128 | bx028984 | AT1G13635 | DNA glycosylase superfamily protein | -1.032 | -0.696 | -4.878 |
| 4 | 20179 | bx029051 | AT3G21240 | 4-coumarate:CoA ligase 2 | -0.713 | -1.238 | -2.932 |
| 4 | 20230 | bx029117 | AT2G46770 | NAC SECONDARY WALL THICKENING PROMOTING FACTOR1, Arabidopsis NAC domain containing protein 43, NAC (No Apical Meristem) domain transcriptional regulator superfamily protein | -1.353 | -2.064 | -4.049 |
| 4 | 20345 | bx029261 | AT2G40480 | Plant protein of unknown function (DUF827) | -0.846 | -1.703 | -2.857 |
| 4 | 20416 | bx029353 | AT2G26250 | FIDDLEHEAD, 3-ketoacyl-CoA synthase 10 | -1.316 | -0.554 | -2.495 |
| 4 | 20499 | bx029462 | AT1G21070 | Nucleotide-sugar transporter family protein | -2.137 | -1.335 | -4.845 |
| 4 | 20709 | bx029742 |  |  | -1.117 | -1.904 | -4.366 |
| 4 | 21065 | bx030225 | AT2G38320 | TRICHOME BIREFRINGENCE-LIKE 34 | -1.124 | -0.947 | -5.194 |
| 4 | 21108 | bx030283 | AT3G28050 | nodulin MtN21 /EamA-like transporter family protein | -1.720 | -1.406 | -3.156 |
| 4 | 21171 | bx030374 | AT2G33990 | IQ-domain 9 | -1.343 | -2.150 | -3.974 |
| 4 | 21310 | bx030568 | AT1G02640 | beta-xylosidase 2 | -1.467 | -2.314 | -6.426 |
| 4 | 21341 | bx030603 | AT2G25735 | unknown protein | -0.752 | -2.054 | -4.115 |
| 4 | 21407 | bx030690 | AT4G17220 | microtubule-associated proteins 70-5 | -2.440 | -1.681 | -4.139 |
| 4 | 21546 | bx030877 | AT5G42710 | unknown protein | -1.013 | -0.921 | -4.657 |
| 4 | 21567 | bx030900 |  |  | -1.983 | -1.733 | -4.851 |
| 4 | 21651 | bx031007 | AT3G14870 | Plant protein of unknown function (DUF641) | -1.442 | -1.285 | -3.191 |
| 4 | 21780 | bx031203 | AT3G55990 | TRICHOME BIREFRINGENCE-LIKE 29, ESKIMO 1, Plant protein of unknown function (DUF828) | -2.045 | -1.766 | -5.287 |
| 4 | 21842 | bx031284 | AT2G31930 | unknown protein | -1.738 | -1.765 | -5.661 |
| 4 | 22424 | bx032106 | AT3G43430 | RING/U-box superfamily protein | 1.382 | -1.799 | -2.513 |
| 4 | 22880 | bx032774 |  |  | -2.293 | -0.529 | -3.201 |
| 4 | 22965 | bx032890 | AT1G75280 | NmrA-like negative transcriptional regulator family protein | -1.395 | -0.925 | -2.675 |
| 4 | 23666 | bx033896 | AT1G47410 | unknown protein | -1.350 | -1.274 | -4.983 |
| 4 | 24602 | bx035257 | AT2G36880 | methionine adenosyltransferase 3 | 0.073 | -1.238 | -2.416 |
| 4 | 24836 | bx035594 | AT3G56050 | Protein kinase family protein | -1.074 | -1.002 | -2.846 |
| 4 | 24838 | bx035596 | AT2G27740 | Family of unknown function (DUF662) | -1.610 | -1.291 | -4.507 |
| 4 | 25192 | bx036094 | AT5G40230 | nodulin MtN21 /EamA-like transporter family protein | -1.769 | -1.520 | -3.666 |
| 4 | 26151 | bx037603 | AT3G51550 | FERONIA, Malectin/receptor-like protein kinase family protein | -1.516 | -0.836 | -2.921 |
| 4 | 26160 | bx037616 | AT5G01360 | TRICHOME BIREFRINGENCE-LIKE 3, Plant protein of unknown function (DUF828) | -0.875 | -1.263 | -5.284 |
| 4 | 26192 | bx037661 | AT1G27920 | microtubule-associated protein 65-8 | -1.333 | -1.304 | -5.078 |
| 4 | 26322 | bx037838 | AT2G46300 | Late embryogenesis abundant (LEA) hydroxyproline-rich glycoprotein family | -1.550 | -2.054 | -3.731 |
| 4 | 26357 | bx037891 | AT4G27595 | Plant protein of unknown function (DUF827) | -1.476 | -1.565 | -5.261 |
| 4 | 26431 | bx037980 | AT3G15050 | IQ-domain 10 | -1.340 | -1.323 | -5.343 |
| 4 | 26476 | bx038045 | AT1G08250 | Arabidopsis thaliana arogenate dehydratase 6, arogenate dehydratase 6 | 1.524 | -0.593 | -2.827 |
| 4 | 26679 | bx038318 | AT1G09610 | Protein of unknown function (DUF579) | -1.945 | -1.897 | -5.855 |
| 4 | 26752 | bx038415 |  |  | -1.395 | -1.075 | -4.090 |
| 4 | 26778 | bx038448 | AT5G60490 | FASCICLIN-like arabinogalactan-protein 12 | -1.274 | -1.926 | -5.764 |
| 4 | 26795 | bx038472 | AT3G56230 | BTB/POZ domain-containing protein | -0.819 | -0.915 | -3.957 |
| 4 | 26840 | bx038528 | AT1G07120 | unknown protein | -1.339 | -1.579 | -4.292 |
| 4 | 26882 | bx038583 | AT5G16600 | myb domain protein 43 | -0.377 | -0.688 | -2.429 |
| 4 | 26942 | bx038665 | AT4G33550 | Bifunctional inhibitor/lipid-transfer protein/seed storage 2S albumin superfamily protein | -0.754 | -1.200 | -4.220 |
| 4 | 26967 | bx038698 | AT4G37240 | unknown protein | -1.422 | -1.598 | -4.036 |
| 4 | 26975 | bx038708 | AT1G77110 | PIN-FORMED 6, Auxin efflux carrier family protein | -1.472 | -3.185 | -5.483 |
| 4 | 27099 | bx038867 | AT1G19300 | PARVUS, GAOLAOZHUANGREN 1, GALACTURONOSYLTRANSFERASE-LIKE 1, Nucleotide-diphospho-sugar transferases superfamily protein | 0.348 | -1.037 | -3.753 |
| 4 | 27102 | bx038871 | AT3G27960 | Tetratricopeptide repeat (TPR)-like superfamily protein | -1.836 | -1.422 | -3.796 |
| 4 | 27141 | bx038921 | AT5G03170 | ARABIDOPSIS FASCICLIN-LIKE ARABINOGALACTAN-PROTEIN 11, FASCICLIN-like arabinogalactan-protein 11 | -1.529 | -1.388 | -5.142 |
| 4 | 27146 | bx038926 | AT5G42930 | alpha/beta-Hydrolases superfamily protein | 1.064 | -0.674 | -2.355 |
| 4 | 27153 | bx038936 | AT5G48920 | tracheary element differentiation-related 7 | -1.044 | 0.170 | -4.363 |
| 4 | 27170 | bx038961 | AT1G20850 | xylem cysteine peptidase 2 | -1.536 | -0.411 | -3.205 |
| 4 | 27210 | bx039012 | AT3G60470 | Plant protein of unknown function (DUF247) | -0.004 | -1.663 | -2.310 |
| 4 | 27219 | bx039024 | AT5G60490 | FASCICLIN-like arabinogalactan-protein 12 | -0.923 | -1.847 | -5.159 |
| 4 | 27220 | bx039025 | AT5G34940 | glucuronidase 3 | -0.603 | -1.485 | -3.643 |
| 4 | 27483 | bx039371 |  |  | 0.635 | -0.642 | -3.195 |
| 4 | 27601 | bx039522 |  |  | -0.566 | -1.125 | -4.790 |
| 4 | 27626 | bx039552 | AT4G00370 | anion transporter 2, Major facilitator superfamily protein | -1.147 | 0.501 | -3.079 |
| 4 | 27676 | bx039621 | AT3G06840 | unknown protein | -1.753 | -2.117 | -3.935 |
| 4 | 27677 | bx039622 | AT4G27435 | Protein of unknown function (DUF1218) | -2.061 | -1.547 | -4.591 |
| 4 | 27739 | bx039706 | AT3G52790 | peptidoglycan-binding LysM domain-containing protein | -1.664 | -2.045 | -5.559 |
| 4 | 27771 | bx039748 |  |  | -1.270 | -1.855 | -3.678 |
| 4 | 27774 | bx039751 |  |  | -1.590 | -1.957 | -5.464 |
| 4 | 27776 | bx039754 | AT3G27200 | Cupredoxin superfamily protein | -1.993 | -0.537 | -3.632 |
| 4 | 27814 | bx039809 | AT5G05390 | laccase 12 | -0.537 | -1.934 | -6.127 |
| 4 | 27877 | bx039888 |  |  | -1.035 | -2.384 | -5.143 |
| 4 | 27942 | bx039970 |  |  | -0.921 | -2.155 | -3.970 |
| 4 | 28036 | bx040093 | AT1G31940 | unknown protein | -0.919 | -2.640 | -4.405 |
| 4 | 28043 | bx040101 | AT5G02440 | unknown protein | -0.047 | -0.074 | -3.128 |
| 4 | 28052 | bx040115 |  |  | -0.656 | -0.708 | -3.814 |
| 4 | 28128 | bx040210 | AT1G52240 | phytochrome interacting RopGEF 1, RHO guanyl-nucleotide exchange factor 11 | -2.053 | -0.614 | -2.458 |
| 4 | 28173 | bx040266 | AT4G25240 | SKU5 similar 1 | -0.896 | -0.997 | -2.510 |
| 4 | 28187 | bx040285 | AT2G47500 | P-loop nucleoside triphosphate hydrolases superfamily protein with CH (Calponin Homology) domain | -0.682 | -0.734 | -3.698 |
| 4 | 28196 | bx040297 |  |  | -1.140 | -2.359 | -3.967 |
| 4 | 28324 | bx040454 | AT2G03200 | Eukaryotic aspartyl protease family protein | -2.138 | -1.094 | -4.329 |
| 4 | 28330 | bx040462 |  |  | -1.188 | -1.614 | -3.910 |
| 4 | 28432 | bx040594 | AT3G21550 | Arabidopsis thaliana DUF679 domain membrane protein 2, DUF679 domain membrane protein 2 | -1.204 | 0.017 | -3.182 |
| 4 | 28567 | bx040765 |  |  | -1.423 | -1.177 | -3.099 |
| 4 | 28585 | bx040789 | AT1G51680 | ARABIDOPSIS THALIANA 4-COUMARATE:COA LIGASE 1, 4-coumarate:CoA ligase 1 | -0.711 | -1.125 | -2.674 |
| 4 | 28611 | bx040824 | AT5G59310 | lipid transfer protein 4 | -1.020 | -0.553 | -3.708 |
| 4 | 28788 | bx041048 | AT3G44220 | Late embryogenesis abundant (LEA) hydroxyproline-rich glycoprotein family | -1.266 | -2.018 | -7.214 |
| 4 | 28806 | bx041075 | AT1G27440 | Exostosin family protein | -1.687 | -1.836 | -4.785 |
| 4 | 28828 | bx041101 | AT3G16920 | chitinase-like protein 2 | -1.703 | -1.629 | -4.709 |
| 4 | 28857 | bx041140 | AT3G16920 | chitinase-like protein 2 | -1.543 | -1.202 | -4.889 |
| 4 | 28879 | bx041168 | AT1G65870 | Disease resistance-responsive (dirigent-like protein) family protein | -2.437 | -0.610 | -3.648 |
| 4 | 28960 | bx041267 | AT3G12955 | SAUR-like auxin-responsive protein family | -1.508 | -1.533 | -5.507 |
| 4 | 29015 | bx041336 | AT1G01780 | PLIM2b, GATA type zinc finger transcription factor family protein | -1.822 | -2.909 | -5.284 |
| 4 | 29100 | bx041439 | AT1G58170 | Disease resistance-responsive (dirigent-like protein) family protein | -1.783 | -1.526 | -4.336 |
| 4 | 29132 | bx041479 | AT4G28500 | SECONDARY WALL-ASSOCIATED NAC DOMAIN PROTEIN 2, NAC domain containing protein 73 | -1.532 | -0.914 | -4.566 |
| 4 | 29183 | bx041548 | AT2G34410 | REDUCED WALL ACETYLATION 3, O-acetyltransferase family protein | -0.846 | -0.943 | -3.143 |
| 4 | 29417 | bx041849 |  |  | -1.031 | -2.284 | -3.948 |
| 4 | 29736 | bx042254 |  |  | -1.887 | -1.714 | -4.857 |
| 4 | 29847 | bx042391 | AT5G52340 | exocyst subunit exo70 family protein A2 | -1.098 | -1.771 | -3.771 |
| 4 | 30078 | bx042666 | AT5G43230 | unknown protein | -1.481 | -1.402 | -3.206 |
| 4 | 30111 | bx042715 | AT1G26560 | beta glucosidase 40 | -1.186 | -0.157 | -2.350 |
| 4 | 30144 | bx042760 | AT5G02640 | unknown protein | -1.009 | -1.503 | -2.726 |
| 4 | 30269 | bx042918 | AT4G38840 | SAUR-like auxin-responsive protein family | -2.448 | -1.670 | -4.419 |
| 4 | 30517 | bx043241 | AT5G46295 | unknown protein | -0.866 | -1.095 | -2.463 |
| 4 | 30522 | bx043246 | AT2G45040 | Matrixin family protein | -1.023 | -1.993 | -4.465 |
| 4 | 30538 | bx043265 | AT2G44500 | O-fucosyltransferase family protein | -0.317 | -0.529 | -2.408 |
| 4 | 30607 | bx043357 | AT3G15050 | IQ-domain 10 | -1.347 | -2.003 | -5.659 |
| 4 | 30756 | bx043551 |  |  | -2.359 | -1.007 | -3.750 |
| 4 | 30915 | bx043760 | AT5G66920 | SKU5 similar 17, SKU5 similar 17 | -1.406 | -2.047 | -3.825 |
| 4 | 31063 | bx043945 | AT1G74110 | cytochrome P450, family 78, subfamily A, polypeptide 10, cytochrome P450, family 78, subfamily A, polypeptide 10 | 0.780 | -2.740 | -3.332 |
| 4 | 31242 | bx044175 | AT2G45850 | AT hook motif DNA-binding family protein | -0.588 | -0.942 | -2.471 |
| 4 | 31377 | bx044352 | AT2G28780 | unknown protein | -1.688 | -1.620 | -4.392 |
| 4 | 31400 | bx044385 | AT2G04850 | Auxin-responsive family protein | -0.443 | -0.836 | -3.852 |
| 4 | 31522 | bx044529 |  |  | -0.447 | -0.292 | -2.366 |
| 4 | 31632 | bx044672 | AT1G47410 | unknown protein | -1.513 | -1.606 | -3.653 |
| 4 | 31651 | bx044694 |  |  | -1.198 | -0.654 | -3.145 |
| 4 | 32270 | bx045475 | AT2G29420 | GLUTATHIONE S-TRANSFERASE 25, glutathione S-transferase tau 7 | -0.883 | -0.953 | -2.381 |
| 4 | 32351 | bx045575 | AT3G15540 | MASSUGU 2, indole-3-acetic acid inducible 19 | -0.082 | -1.908 | -3.472 |
| 4 | 32408 | bx045639 | AT5G60210 | ROP interactive partner 5 | -1.650 | -1.658 | -3.632 |
| 4 | 32482 | bx045731 | AT1G11600 | cytochrome P450, family 77, subfamily B, polypeptide 1, cytochrome P450, family 77, subfamily B, polypeptide 1 | -2.927 | -1.189 | -3.632 |
| 4 | 32611 | bx045894 | AT1G29200 | O-fucosyltransferase family protein | -1.459 | -1.803 | -4.030 |
| 4 | 32765 | bx046086 | AT2G46930 | Pectinacetylesterase family protein | -0.872 | -1.992 | -3.651 |
| 4 | 32894 | bx046250 | AT2G28315 | Nucleotide/sugar transporter family protein | -1.796 | -2.585 | -6.009 |
| 4 | 32997 | bx046376 | AT1G65680 | expansin B2 | -1.027 | -0.877 | -2.744 |
| 4 | 33039 | bx046427 | AT2G36026 | Ovate family protein | -1.598 | -2.064 | -3.925 |
| 4 | 33174 | bx046603 | AT2G04850 | Auxin-responsive family protein | -1.560 | -1.672 | -3.781 |
| 4 | 33311 | bx046782 | AT5G34940 | glucuronidase 3 | -0.309 | -1.352 | -3.085 |
| 4 | 33427 | bx046917 | AT5G60490 | FASCICLIN-like arabinogalactan-protein 12 | -0.851 | -2.155 | -5.318 |
| 4 | 33531 | bx047049 | AT3G52790 | peptidoglycan-binding LysM domain-containing protein | -1.656 | -2.021 | -5.146 |
| 4 | 33595 | bx047129 | AT5G60540 | EMBRYO DEFECTIVE 2407, pyridoxine biosynthesis 2 | -0.936 | -1.626 | -4.222 |
| 4 | 33722 | bx047280 | AT5G25830 | GATA transcription factor 12 | -2.145 | -1.715 | -4.497 |
| 4 | 33756 | bx047316 | AT5G49350 | Glycine-rich protein family | -1.485 | -2.621 | -5.731 |
| 4 | 33816 | bx047393 |  |  | -1.712 | -1.523 | -4.985 |
| 4 | 33858 | bx047449 | AT3G08500 | myb domain protein 83 | -1.614 | -2.284 | -4.669 |
| 4 | 33983 | bx047616 | AT2G37080 | ROP interactive partner 3 | -1.757 | -1.694 | -3.923 |
| 4 | 34059 | bx047713 | AT2G36880 | methionine adenosyltransferase 3 | 0.035 | -1.280 | -2.655 |
| 4 | 34068 | bx047723 | AT1G63300 | Myosin heavy chain-related protein | -1.485 | -1.615 | -5.201 |
| 4 | 34073 | bx047730 | AT2G26250 | FIDDLEHEAD, 3-ketoacyl-CoA synthase 10 | -1.529 | -0.358 | -2.435 |
| 4 | 34169 | bx047846 | AT2G41610 | unknown protein | -1.824 | -2.046 | -4.864 |
| 4 | 34250 | bx047947 | AT3G20860 | NIMA-related kinase 5 | -0.506 | -1.974 | -3.756 |
| 4 | 34449 | bx048201 | AT1G10940 | SNF1-related protein kinase 2.4, SUCROSE NONFERMENTING 1-RELATED PROTEIN KINASE 2-4, ARABIDOPSIS SERINE/THREONINE KINASE 1, Protein kinase superfamily protein | -0.886 | -0.776 | -2.340 |
| 4 | 34522 | bx048286 | AT5G58800 | Quinone reductase family protein | -1.381 | -1.459 | -3.488 |
| 4 | 34695 | bx048495 |  |  | -1.119 | -0.763 | -3.085 |
| 4 | 34767 | bx048579 | AT5G04200 | metacaspase 2f, metacaspase 9 | -0.606 | 0.210 | -2.417 |
| 4 | 34855 | bx048696 |  |  | -2.126 | -0.781 | -4.341 |
| 4 | 34957 | bx048821 | AT4G38840 | SAUR-like auxin-responsive protein family | -2.549 | -1.172 | -3.503 |
| 4 | 35088 | bx048993 | AT4G18780 | LEAF WILTING 2, IRREGULAR XYLEM 1, CELLULOSE SYNTHASE 8, cellulose synthase family protein | -1.262 | -1.493 | -5.167 |
| 4 | 35155 | bx049076 |  |  | -1.522 | -2.248 | -4.138 |
| 4 | 35263 | bx049214 | AT5G37478 | TPX2 (targeting protein for Xklp2) protein family | -1.464 | -2.333 | -5.766 |
| 4 | 35380 | bx049367 | AT1G75680 | glycosyl hydrolase 9B7 | -1.302 | -1.481 | -2.804 |
| 4 | 35563 | bx049609 | AT5G53486 | unknown protein | -0.215 | -1.351 | -2.974 |
| 4 | 35659 | bx049744 | AT3G52900 | Family of unknown function (DUF662) | -1.218 | -0.394 | -2.476 |
| 4 | 35661 | bx049746 | AT2G33385 | actin-related protein C2B | -1.516 | -1.427 | -5.165 |
| 4 | 35854 | bx050010 | AT4G28610 | phosphate starvation response 1, phosphate starvation response 1 | -2.211 | -1.614 | -4.566 |
| 4 | 36786 | bx051151 | AT5G25620 | YUCCA6, Flavin-binding monooxygenase family protein | -1.670 | -0.661 | -3.144 |
| 4 | 37232 | bx051734 | AT3G62020 | germin-like protein 10 | -1.327 | -0.989 | -2.620 |
| 4 | 37240 | bx051742 | AT3G07040 | RESISTANCE TO PSEUDOMONAS SYRINGAE 3, RESISTANCE TO P. SYRINGAE PV MACULICOLA 1, NB-ARC domain-containing disease resistance protein | -0.085 | -1.390 | -2.343 |
| 4 | 37334 | bx051858 | AT5G55860 | Plant protein of unknown function (DUF827) | -0.847 | -0.227 | -3.033 |
| 4 | 37335 | bx051859 | AT4G17220 | microtubule-associated proteins 70-5 | -2.423 | -1.742 | -3.978 |
| 4 | 37387 | bx051924 | AT3G55990 | TRICHOME BIREFRINGENCE-LIKE 29, ESKIMO 1, Plant protein of unknown function (DUF828) | -1.645 | -1.631 | -4.303 |
| 4 | 37435 | bx051980 |  |  | -1.329 | -1.875 | -4.001 |
| 4 | 37516 | bx052084 | AT3G16920 | chitinase-like protein 2 | -1.273 | -1.018 | -4.503 |
| 4 | 37780 | bx052415 | AT5G07150 | Leucine-rich repeat protein kinase family protein | -1.030 | -1.464 | -2.834 |
| 4 | 38031 | bx052762 |  |  | -0.921 | -0.818 | -2.863 |
| 4 | 38095 | bx052842 | AT5G02420 | unknown protein | -1.554 | -1.955 | -3.702 |
| 4 | 38114 | bx052871 | AT1G49000 | unknown protein | -0.398 | -1.155 | -2.914 |
| 4 | 38235 | bx053013 |  |  | -1.545 | -1.396 | -3.851 |
| 4 | 38315 | bx053123 | AT3G23590 | REF4-related 1 | -1.883 | -1.630 | -4.021 |
| 4 | 38502 | bx053371 | AT5G18460 | Protein of Unknown Function (DUF239) | -0.888 | -0.519 | -2.928 |
| 4 | 38602 | bx053496 | AT3G51970 | ARABIDOPSIS THALIANA STEROL O-ACYLTRANSFERASE 1, acyl-CoA sterol acyl transferase 1 | 1.535 | 0.246 | -3.157 |
| 4 | 38863 | bx053837 |  |  | -1.852 | -0.797 | -3.035 |
| 4 | 39029 | bx054047 | AT4G35160 | O-methyltransferase family protein | -1.146 | -1.858 | -4.535 |
| 4 | 39290 | bx054422 | AT5G54160 | O-methyltransferase 1 | -1.452 | -2.103 | -4.334 |
| 4 | 39353 | bx054509 | AT1G52510 | alpha/beta-Hydrolases superfamily protein | -0.544 | -2.032 | -3.212 |
| 4 | 39450 | bx054641 |  |  | -1.386 | -3.427 | -5.808 |
| 4 | 39505 | bx054714 | AT4G37760 | squalene epoxidase 3 | -1.562 | -0.955 | -2.749 |
| 4 | 39508 | bx054717 | AT5G41790 | COP1-interactive protein 1 | -1.448 | -1.593 | -5.297 |
| 4 | 39584 | bx054833 | AT2G33385 | actin-related protein C2B | -1.639 | -1.564 | -4.589 |
| 4 | 39598 | bx054856 | AT4G33550 | Bifunctional inhibitor/lipid-transfer protein/seed storage 2S albumin superfamily protein | -0.754 | -1.178 | -3.980 |
| 4 | 39638 | bx054910 | AT5G13700 | polyamine oxidase 1 | -1.375 | -2.035 | -4.463 |
| 4 | 39989 | bx055414 | AT2G35150 | EXORDIUM LIKE 7, EXORDIUM like 1 | -2.054 | -2.721 | -5.322 |
| 4 | 40008 | bx055436 | AT3G14170 | Plant protein of unknown function (DUF936) | -1.703 | -1.294 | -5.424 |
| 4 | 40046 | bx055487 | AT5G42260 | beta glucosidase 12 | -1.542 | -0.080 | -2.354 |
| 4 | 40433 | bx055961 | AT1G05260 | RARE COLD INDUCIBLE GENE 3, Peroxidase superfamily protein | -2.243 | -1.210 | -3.729 |
| 4 | 40551 | bx056109 | AT4G22900 | Protein of unknown function (DUF1191) | -1.168 | -2.108 | -3.545 |
| 4 | 40737 | bx056340 | AT1G27930 | Protein of unknown function (DUF579) | -1.444 | -0.689 | -3.352 |
| 4 | 40837 | bx056462 |  |  | -0.895 | -2.164 | -3.721 |
| 4 | 40879 | bx056509 | AT2G25630 | beta glucosidase 14 | -2.102 | -0.862 | -2.574 |
| 4 | 40959 | bx056611 | AT2G48110 | reduced epidermal fluorescence 4 | -1.026 | -0.831 | -2.894 |
| 4 | 41095 | bx056777 | AT3G13600 | calmodulin-binding family protein | 0.133 | -0.972 | -2.850 |
| 4 | 41244 | bx056964 |  |  | 1.120 | -2.650 | -3.715 |
| 4 | 41392 | bx057134 | AT3G52770 | LITTLE ZIPPER 3, protein binding | -0.850 | -2.776 | -4.444 |
| 4 | 41409 | bx057153 |  |  | -2.259 | -2.731 | -5.674 |
| 4 | 41473 | bx057240 | AT1G76070 | unknown protein | -1.671 | -1.342 | -2.926 |
| 4 | 41676 | bx057487 |  |  | -1.084 | -0.403 | -2.649 |
| 4 | 41931 | bx057793 |  |  | -0.112 | -0.042 | -2.669 |
| 4 | 41957 | bx057822 | AT5G60490 | FASCICLIN-like arabinogalactan-protein 12 | -2.395 | -2.215 | -4.516 |
| 4 | 42043 | bx057921 |  |  | -1.026 | -1.488 | -2.859 |
| 4 | 42328 | bx058273 | AT1G61170 | unknown protein | -1.277 | -1.377 | -3.238 |
| 4 | 42586 | bx058589 | AT4G03500 | Ankyrin repeat family protein | -0.594 | -0.714 | -2.730 |
| 4 | 42783 | bx058834 | AT5G36890 | beta glucosidase 42 | -1.158 | -0.927 | -3.514 |
| 4 | 43031 | bx059144 |  |  | -0.791 | -0.910 | -3.441 |
| 4 | 43032 | bx059145 | AT1G04110 | STOMATAL DENSITY AND DISTRIBUTION, Subtilase family protein | 2.008 | -1.604 | -2.722 |
| 4 | 43061 | bx059178 | AT2G37090 | IRREGULAR XYLEM 9, Nucleotide-diphospho-sugar transferases superfamily protein | -1.112 | -1.614 | -5.645 |
| 4 | 43140 | bx059273 |  |  | -1.539 | -1.616 | -3.178 |
| 4 | 43183 | bx059326 | AT4G18780 | LEAF WILTING 2, IRREGULAR XYLEM 1, CELLULOSE SYNTHASE 8, cellulose synthase family protein | -1.158 | -1.223 | -4.904 |
| 4 | 43249 | bx059403 |  |  | -0.659 | -1.714 | -2.893 |
| 4 | 43475 | bx059692 | AT5G11790 | N-MYC downregulated-like 2 | -0.678 | -1.017 | -2.413 |
| 4 | 43588 | bx059835 | AT5G42710 | unknown protein | -1.090 | -0.930 | -2.778 |
| 4 | 43765 | bx060054 | AT2G38025 | Cysteine proteinases superfamily protein | -2.390 | 0.817 | -2.201 |
| 4 | 44107 | bx060467 | AT1G79180 | myb domain protein 63 | -0.504 | -0.396 | -2.993 |
| 4 | 44232 | bx060612 | AT3G45010 | serine carboxypeptidase-like 48 | -0.959 | -0.082 | -2.493 |
| 4 | 44235 | bx060616 |  |  | -1.832 | -0.693 | -2.369 |
| 4 | 44245 | bx060627 | AT5G60900 | receptor-like protein kinase 1 | -1.651 | -0.490 | -2.411 |
| 4 | 44249 | bx060633 | AT5G47635 | Pollen Ole e 1 allergen and extensin family protein | -1.709 | -1.478 | -5.391 |
| 4 | 44325 | bx060723 | AT5G60490 | FASCICLIN-like arabinogalactan-protein 12 | 1.289 | -2.111 | -2.768 |
| 4 | 44481 | bx060924 | AT4G14760 | kinase interacting (KIP1-like) family protein | -2.108 | -1.152 | -3.786 |
| 4 | 44654 | bx061132 | AT3G52490 | Double Clp-N motif-containing P-loop nucleoside triphosphate hydrolases superfamily protein | -2.020 | -0.784 | -2.440 |
| 4 | 44977 | bx061529 |  |  | -0.928 | -1.461 | -2.966 |
| 4 | 45063 | bx061636 |  |  | -1.841 | -1.998 | -4.934 |
| 4 | 45204 | bx061815 | AT3G61750 | Cytochrome b561/ferric reductase transmembrane with DOMON related domain | -1.433 | -2.112 | -5.436 |
| 4 | 45841 | bx062615 | AT3G07040 | RESISTANCE TO PSEUDOMONAS SYRINGAE 3, RESISTANCE TO P. SYRINGAE PV MACULICOLA 1, NB-ARC domain-containing disease resistance protein | 0.301 | -1.402 | -3.036 |
| 4 | 46103 | bx062964 | AT1G74100 | CORONATINE INDUCED-7, ARABIDOPSIS SULFOTRANSFERASE 5A, sulfotransferase 16 | -0.413 | -3.039 | -4.573 |
| 4 | 46195 | bx063079 |  |  | -1.003 | -1.785 | -3.192 |
| 4 | 46260 | bx063165 |  |  | -1.414 | -1.880 | -3.619 |
| 4 | 46563 | bx063542 |  |  | -2.072 | -1.977 | -4.108 |
| 4 | 46580 | bx063560 | AT1G73630 | EF hand calcium-binding protein family | 0.567 | -0.076 | -2.499 |
| 4 | 46662 | bx063655 | AT5G13700 | polyamine oxidase 1 | -0.798 | -1.881 | -3.714 |
| 4 | 46894 | bx063929 | AT1G13635 | DNA glycosylase superfamily protein | -1.486 | -1.021 | -2.785 |
| 4 | 46920 | bx063961 | AT5G47180 | Plant VAMP (vesicle-associated membrane protein) family protein | -3.078 | -2.264 | -5.874 |
| 4 | 47099 | bx064159 | AT2G37080 | ROP interactive partner 3 | -1.878 | -1.698 | -3.838 |
| 4 | 47309 | bx064394 | AT4G27435 | Protein of unknown function (DUF1218) | -1.847 | -1.796 | -3.797 |
| 4 | 47554 | bx064677 |  |  | -0.258 | -0.786 | -2.401 |
| 4 | 47714 | bx064853 |  |  | -1.755 | -1.843 | -5.952 |
| 4 | 48145 | bx065341 | AT3G45650 | nitrate excretion transporter1 | -1.118 | -1.550 | -2.780 |
| 4 | 48255 | bx065463 | AT4G27730 | ARABIDOPSIS THALIANA OLIGOPEPTIDE TRANSPORTER 6, oligopeptide transporter 1 | -0.816 | -0.901 | -2.526 |
| 4 | 48481 | bx065762 | AT4G28500 | SECONDARY WALL-ASSOCIATED NAC DOMAIN PROTEIN 2, NAC domain containing protein 73 | -1.448 | -1.104 | -4.370 |
| 4 | 48630 | bx065953 | AT4G28500 | SECONDARY WALL-ASSOCIATED NAC DOMAIN PROTEIN 2, NAC domain containing protein 73 | -1.258 | -0.457 | -3.482 |
| 4 | 48930 | bx066334 | AT5G67090 | Subtilisin-like serine endopeptidase family protein | -2.002 | -0.767 | -2.372 |
| 4 | 49429 | bx066942 | AT5G37478 | TPX2 (targeting protein for Xklp2) protein family | -2.033 | -2.622 | -5.055 |
| 4 | 50253 | bx067903 | AT5G47800 | Phototropic-responsive NPH3 family protein | -1.251 | -0.214 | -2.361 |
| 4 | 50334 | bx067993 | AT5G07620 | Protein kinase superfamily protein | -0.502 | -0.388 | -2.314 |
| 4 | 50628 | bx068330 | AT3G55990 | TRICHOME BIREFRINGENCE-LIKE 29, ESKIMO 1, Plant protein of unknown function (DUF828) | -2.039 | -0.996 | -4.772 |
| 4 | 50727 | bx068460 |  |  | 0.020 | -2.523 | -3.647 |
| 4 | 50873 | bx068641 | AT5G42710 | unknown protein | -1.445 | -1.106 | -3.067 |
| 4 | 50880 | bx068649 | AT5G47650 | ARABIDOPSIS THALIANA NUDIX HYDROLASE HOMOLOG 2, nudix hydrolase homolog 2 | -0.049 | -1.979 | -3.147 |
| 4 | 51585 | bx069619 |  |  | -0.868 | -1.446 | -2.746 |
| 4 | 52513 | bx071010 | AT5G45910 | GDSL-like Lipase/Acylhydrolase superfamily protein | -1.028 | -1.193 | -2.660 |
| 4 | 52697 | bx071295 |  |  | -1.079 | -0.954 | -2.348 |
| 4 | 53037 | bx071783 | AT1G80450 | VQ motif-containing protein | -1.266 | -0.607 | -2.396 |
| 4 | 53525 | bx072328 | AT1G66230 | myb domain protein 20 | -0.479 | -1.014 | -2.380 |
| 4 | 53611 | bx072438 | AT2G38080 | LACCASE 4, IRREGULAR XYLEM 12, ARABIDOPSIS LACCASE-LIKE MULTICOPPER OXIDASE 4, Laccase/Diphenol oxidase family protein | -1.050 | -2.224 | -5.007 |
| 4 | 53985 | bx072880 | AT4G12350 | myb domain protein 42 | -1.670 | -1.655 | -3.346 |
| 4 | 53989 | bx072884 | AT5G24600 | Protein of unknown function, DUF599 | -0.911 | -0.588 | -2.685 |
| 4 | 54199 | bx073122 |  |  | -1.015 | -1.780 | -4.088 |
| 4 | 54723 | bx073718 | AT4G02250 | Plant invertase/pectin methylesterase inhibitor superfamily protein | -0.143 | 0.011 | -2.864 |
| 4 | 54725 | bx073720 | AT5G44030 | IRREGULAR XYLEM 5, cellulose synthase A4 | -1.686 | -1.722 | -5.588 |
| 4 | 55014 | bx074028 |  |  | -2.040 | -0.875 | -3.747 |
| 4 | 55238 | bx074312 | AT1G76670 | Nucleotide-sugar transporter family protein | -2.008 | -1.449 | -3.778 |
| 4 | 55276 | bx074360 |  |  | -1.221 | -2.299 | -4.042 |
| 4 | 55350 | bx074460 | AT1G02400 | DOWNSTREAM TARGET OF AGL15 1, ARABIDOPSIS THALIANA GIBBERELLIN 2-OXIDASE 6, Arabidopsis thaliana gibberellin 2-oxidase 4, gibberellin 2-oxidase 6 | 0.764 | -0.866 | -2.668 |
| 4 | 55380 | bx074499 | AT5G60720 | Protein of unknown function, DUF547 | -1.683 | -2.239 | -5.948 |
| 4 | 55488 | bx074640 |  |  | -1.280 | -0.245 | -2.485 |
| 4 | 55593 | bx074763 |  |  | -1.841 | -1.449 | -3.368 |
| 4 | 55611 | bx074794 |  |  | 0.528 | -1.114 | -2.884 |
| 4 | 55819 | bx075164 | AT5G60540 | EMBRYO DEFECTIVE 2407, pyridoxine biosynthesis 2 | -1.210 | -1.540 | -4.062 |
| 4 | 55856 | bx075209 |  |  | -0.405 | -0.840 | -3.111 |
| 4 | 55977 | bx075375 | AT5G49350 | Glycine-rich protein family | -1.363 | -2.618 | -5.463 |
| 4 | 56012 | bx075452 | AT5G60210 | ROP interactive partner 5 | -1.295 | -1.083 | -2.923 |
| 4 | 56152 | bx075646 |  |  | 0.086 | -0.869 | -2.420 |
| 4 | 56394 | bx075975 |  |  | -0.143 | -1.002 | -2.914 |
| 4 | 56649 | bx076268 | AT4G35830 | aconitase 1 | 1.120 | 0.432 | -3.168 |
| 4 | 57255 | bx076972 | AT5G23870 | Pectinacetylesterase family protein | -1.064 | -1.817 | -3.539 |
| 4 | 57293 | bx077014 |  |  | -1.892 | -1.309 | -3.070 |
| 4 | 57551 | bx077360 | AT2G24765 | ARF-LIKE 1, ADP-ribosylation factor 3 | -2.410 | -0.932 | -6.103 |
| 4 | 58018 | bx077943 | AT2G40480 | Plant protein of unknown function (DUF827) | -0.852 | -1.669 | -2.877 |
| 4 | 58156 | bx078095 | AT5G60020 | laccase 17 | -0.890 | 0.080 | -4.258 |
| 4 | 58182 | bx078124 |  |  | -0.064 | -0.462 | -2.812 |
| 4 | 58696 | bx078715 | AT4G27435 | Protein of unknown function (DUF1218) | -1.895 | -1.604 | -4.486 |
| 4 | 58712 | bx078739 |  |  | -1.800 | -1.445 | -3.851 |
| 4 | 58819 | bx078874 |  |  | -1.048 | -0.969 | -3.527 |
| 4 | 59204 | bx079325 | AT2G24600 | Ankyrin repeat family protein | 0.729 | 0.654 | -3.229 |
| 4 | 59264 | bx079393 | AT1G26700 | MILDEW RESISTANCE LOCUS O 14, Seven transmembrane MLO family protein | -0.720 | -0.797 | -2.702 |
| 4 | 59786 | bx080015 | AT5G55860 | Plant protein of unknown function (DUF827) | -1.105 | -1.109 | -3.519 |
| 4 | 59829 | bx080073 | AT1G27920 | microtubule-associated protein 65-8 | -1.432 | -1.542 | -4.829 |
| 4 | 59991 | bx080282 | AT2G33385 | actin-related protein C2B | -1.529 | -1.372 | -4.976 |
| 4 | 60233 | bx080593 | AT5G42510 | Disease resistance-responsive (dirigent-like protein) family protein | -1.215 | -0.362 | -2.931 |
| 4 | 60379 | bx080788 | AT1G27440 | Exostosin family protein | -1.063 | -1.176 | -4.541 |
| 4 | 60492 | bx080931 |  |  | -0.699 | -0.978 | -2.358 |
| 4 | 60549 | bx081004 | AT3G42170 | BED zinc finger ;hAT family dimerisation domain | -0.091 | -1.559 | -3.881 |
| 4 | 61224 | bx081856 | AT3G14170 | Plant protein of unknown function (DUF936) | -1.785 | -1.336 | -5.418 |
| 4 | 61357 | bx082064 | AT4G20110 | VACUOLAR SORTING RECEPTOR 3;1, binding protein of 80 kDa 3;1, VACUOLAR SORTING RECEPTOR 7 | -1.142 | -2.056 | -4.915 |
| 4 | 61656 | bx082533 | AT2G26560 | PATATIN-LIKE PROTEIN 2, phospholipase A 2A | 0.607 | -0.662 | -3.111 |
| 5 | 29 | bx000052 | AT2G36830 | TONOPLAST INTRINSIC PROTEIN 1;1, GAMMA TONOPLAST INTRINSIC PROTEIN 1, gamma tonoplast intrinsic protein | -3.634 | -1.285 | -1.247 |
| 5 | 235 | bx000420 |  |  | -2.518 | -1.237 | -1.271 |
| 5 | 586 | bx001034 |  |  | -2.321 | -1.212 | 0.361 |
| 5 | 811 | bx001422 |  |  | -3.206 | -1.780 | -0.589 |
| 5 | 849 | bx001479 |  |  | -2.538 | -1.438 | -0.359 |
| 5 | 1568 | bx002599 |  |  | -2.635 | -1.321 | -1.481 |
| 5 | 2001 | bx003268 | AT1G15520 | Arabidopsis thaliana ATP-binding cassette G40, ATP-binding cassette G40, pleiotropic drug resistance 12 | -2.857 | -0.634 | -0.246 |
| 5 | 2660 | bx004260 | AT1G28100 | unknown protein | -3.644 | -2.526 | -0.989 |
| 5 | 2828 | bx004501 | AT4G32870 | Polyketide cyclase/dehydrase and lipid transport superfamily protein | -2.430 | -0.951 | -0.744 |
| 5 | 2911 | bx004628 | AT2G04039 | unknown protein | -3.340 | -2.428 | -1.082 |
| 5 | 3268 | bx005148 | AT3G16330 | unknown protein | -4.591 | -2.488 | -2.357 |
| 5 | 3290 | bx005180 | AT4G35100 | PLASMA MEMBRANE INTRINSIC PROTEIN 3A, PLASMA MEMBRANE INTRINSIC PROTEIN 2;7, plasma membrane intrinsic protein 3 | -2.650 | -2.605 | -0.065 |
| 5 | 3454 | bx005422 | AT5G40270 | HD domain-containing metal-dependent phosphohydrolase family protein | -2.488 | -1.822 | -1.107 |
| 5 | 4363 | bx006755 | AT5G23230 | nicotinamidase 2 | -2.462 | -1.185 | -0.955 |
| 5 | 4688 | bx007227 | AT5G18600 | Thioredoxin superfamily protein | -2.706 | 3.370 | -0.958 |
| 5 | 4711 | bx007256 | AT1G78060 | Glycosyl hydrolase family protein | -2.347 | -0.467 | -0.731 |
| 5 | 5483 | bx008365 | AT1G73480 | alpha/beta-Hydrolases superfamily protein | -3.372 | -2.182 | -1.082 |
| 5 | 5843 | bx008871 | AT3G28345 | multi-drug resistance 13, ATP-binding cassette B15, ABC transporter family protein | -2.653 | -0.182 | -1.200 |
| 5 | 5923 | bx008972 | AT5G65640 | beta HLH protein 93 | -3.551 | -1.582 | -1.618 |
| 5 | 6061 | bx009151 | AT4G16410 | unknown protein | -3.131 | -1.582 | -0.509 |
| 5 | 6244 | bx009411 | AT2G28080 | UDP-Glycosyltransferase superfamily protein | -3.258 | -1.057 | -1.159 |
| 5 | 7588 | bx011311 | AT1G08800 | Protein of unknown function, DUF593 | -3.883 | -1.361 | -1.422 |
| 5 | 7664 | bx011422 | AT5G04250 | Cysteine proteinases superfamily protein | -2.897 | -2.315 | -0.738 |
| 5 | 7732 | bx011528 | AT5G12960 | Putative glycosyl hydrolase of unknown function (DUF1680) | -2.457 | -0.787 | -1.038 |
| 5 | 7948 | bx011838 | AT3G26740 | CCR-like | -3.147 | 0.313 | -0.691 |
| 5 | 8503 | bx012619 | AT5G53020 | Ribonuclease P protein subunit P38-related | -4.287 | -1.147 | -1.383 |
| 5 | 8740 | bx012945 | AT5G41210 | glutathione S-transferase THETA 1 | -3.776 | -2.512 | -0.971 |
| 5 | 9265 | bx013710 | AT2G36830 | TONOPLAST INTRINSIC PROTEIN 1;1, GAMMA TONOPLAST INTRINSIC PROTEIN 1, gamma tonoplast intrinsic protein | -3.169 | -1.192 | -1.002 |
| 5 | 9313 | bx013778 |  |  | -2.994 | -1.252 | -1.198 |
| 5 | 9848 | bx014552 | AT1G22540 | Major facilitator superfamily protein | -5.575 | -3.817 | -2.499 |
| 5 | 11649 | bx017401 | AT3G03080 | Zinc-binding dehydrogenase family protein | -2.422 | -1.365 | -1.378 |
| 5 | 12605 | bx018897 | AT1G10070 | branched-chain amino acid transaminase 2 | -2.688 | -1.035 | 0.167 |
| 5 | 12831 | bx019194 | AT5G50790 | Nodulin MtN3 family protein | -4.134 | -1.949 | 0.182 |
| 5 | 13484 | bx020078 | AT1G35430 | unknown protein | -3.164 | -1.884 | -0.388 |
| 5 | 13532 | bx020137 | AT2G46410 | CAPRICE, Homeodomain-like superfamily protein | -2.541 | -1.885 | -0.258 |
| 5 | 14089 | bx020867 | AT2G38120 | WAVY ROOTS 5, MODIFIER OF ARF7/NPH4 PHENOTYPES 1, AUXIN RESISTANT 1, Transmembrane amino acid transporter family protein | -2.743 | -1.286 | -1.643 |
| 5 | 14608 | bx021557 |  |  | -3.140 | -1.042 | -0.804 |
| 5 | 14671 | bx021639 | AT5G11460 | Protein of unknown function (DUF581) | -2.361 | -0.957 | -1.148 |
| 5 | 14923 | bx021975 | AT3G29240 | Protein of unknown function (DUF179) | -2.697 | -0.938 | -0.403 |
| 5 | 14933 | bx021986 | AT4G34880 | Amidase family protein | -6.178 | -1.888 | 2.222 |
| 5 | 14962 | bx022023 | AT5G09220 | amino acid permease 2 | -3.276 | -2.315 | -0.773 |
| 5 | 15262 | bx022411 | AT4G14746 | unknown protein | -2.665 | -1.367 | -0.798 |
| 5 | 15364 | bx022553 | AT2G30570 | photosystem II reaction center W | -3.237 | -2.658 | -1.146 |
| 5 | 15441 | bx022647 | AT1G28100 | unknown protein | -3.788 | -2.593 | -1.017 |
| 5 | 15680 | bx022962 | AT3G22210 | unknown protein | -2.899 | -1.993 | -0.993 |
| 5 | 15978 | bx023365 | AT1G04920 | sucrose phosphate synthase 3F | -2.916 | -1.705 | 0.027 |
| 5 | 16015 | bx023411 | AT4G33800 | unknown protein | -2.439 | -1.486 | -0.936 |
| 5 | 16189 | bx023665 | AT1G65870 | Disease resistance-responsive (dirigent-like protein) family protein | -2.621 | -2.087 | -0.915 |
| 5 | 16202 | bx023680 |  |  | -2.374 | -1.373 | -1.265 |
| 5 | 16339 | bx023871 | AT2G36690 | 2-oxoglutarate (2OG) and Fe(II)-dependent oxygenase superfamily protein | -2.706 | -0.688 | -0.840 |
| 5 | 16390 | bx023941 |  |  | -2.482 | -0.834 | 0.623 |
| 5 | 16399 | bx023953 | AT5G41050 | Pollen Ole e 1 allergen and extensin family protein | -2.570 | -0.298 | -0.731 |
| 5 | 16489 | bx024066 | AT4G38960 | B-box type zinc finger family protein | -2.325 | -1.261 | -1.069 |
| 5 | 16614 | bx024258 |  |  | -2.897 | -0.866 | -1.192 |
| 5 | 16656 | bx024320 | AT2G46950 | cytochrome P450, family 709, subfamily B, polypeptide 2, cytochrome P450, family 709, subfamily B, polypeptide 2 | -2.593 | -2.557 | -0.565 |
| 5 | 16698 | bx024378 | AT5G15802 | unknown protein | -2.493 | -1.511 | -0.909 |
| 5 | 16762 | bx024471 | AT4G31500 | SUPERROOT 2, RUNT 1, RED ELONGATED 1, cytochrome P450, family 83, subfamily B, polypeptide 1, ALTERED TRYPTOPHAN REGULATION 4, cytochrome P450, family 83, subfamily B, polypeptide 1 | -2.556 | 0.441 | 1.590 |
| 5 | 16833 | bx024563 | AT1G60550 | enoyl-CoA hydratase/isomerase D | -2.400 | -1.899 | -0.911 |
| 5 | 17093 | bx024887 | AT5G62040 | brother of FT and TFL1, PEBP (phosphatidylethanolamine-binding protein) family protein | -2.397 | -0.662 | -0.425 |
| 5 | 17099 | bx024898 | AT5G51920 | Pyridoxal phosphate (PLP)-dependent transferases superfamily protein | -2.747 | -0.851 | -0.974 |
| 5 | 17121 | bx024928 | AT1G09750 | Eukaryotic aspartyl protease family protein | -3.046 | -0.601 | -1.290 |
| 5 | 17156 | bx024974 | AT5G62140 | unknown protein | -2.529 | -1.187 | -1.214 |
| 5 | 17245 | bx025088 | AT5G55620 | unknown protein | -3.495 | -0.371 | 0.132 |
| 5 | 17479 | bx025417 | AT2G20142 | Toll-Interleukin-Resistance (TIR) domain family protein | -3.003 | -1.685 | -0.891 |
| 5 | 17660 | bx025663 | AT4G25000 | alpha-amylase-like | -2.362 | -1.188 | 1.746 |
| 5 | 17962 | bx026063 | AT1G32960 | Subtilase family protein | -3.060 | -1.469 | -1.648 |
| 5 | 18059 | bx026186 | AT3G15510 | NAC-REGULATED SEED MORPHOLOGY 1, NAC domain containing protein 2, Arabidopsis NAC domain containing protein 56, NAC domain containing protein 2 | -2.749 | -1.616 | -1.519 |
| 5 | 18356 | bx026580 | AT5G23230 | nicotinamidase 2 | -2.395 | -1.194 | -0.942 |
| 5 | 18379 | bx026603 | AT4G37340 | cytochrome P450, family 81, subfamily D, polypeptide 3, cytochrome P450, family 81, subfamily D, polypeptide 3 | -2.597 | -0.794 | 0.402 |
| 5 | 18689 | bx027022 | AT5G60710 | Zinc finger (C3HC4-type RING finger) family protein | -5.104 | -3.930 | -1.900 |
| 5 | 18754 | bx027117 | AT3G07350 | Protein of unknown function (DUF506) | -3.480 | -2.520 | -0.258 |
| 5 | 19279 | bx027832 | AT1G52720 | unknown protein | -2.446 | -1.294 | -1.447 |
| 5 | 19339 | bx027909 | AT2G38695 | unknown protein | -2.927 | -0.776 | -0.446 |
| 5 | 19356 | bx027932 | AT5G21090 | Leucine-rich repeat (LRR) family protein | -2.315 | -1.096 | -0.110 |
| 5 | 19396 | bx027988 | AT1G26940 | Cyclophilin-like peptidyl-prolyl cis-trans isomerase family protein | -2.596 | -0.052 | -0.345 |
| 5 | 19440 | bx028044 | AT4G32480 | Protein of unknown function (DUF506) | -3.806 | -2.392 | -1.131 |
| 5 | 19560 | bx028193 |  |  | -3.361 | -1.575 | -1.312 |
| 5 | 19875 | bx028601 | AT5G56340 | RING/U-box superfamily protein | -2.623 | -1.460 | -1.111 |
| 5 | 20393 | bx029325 | AT3G47340 | DARK INDUCIBLE 6, ARABIDOPSIS THALIANA GLUTAMINE-DEPENDENT ASPARAGINE SYNTHASE 1, glutamine-dependent asparagine synthase 1 | -4.939 | -1.055 | -1.877 |
| 5 | 20753 | bx029800 | AT4G08920 | OUT OF PHASE 2, ELONGATED HYPOCOTYL 4, BLUE LIGHT UNINHIBITED 1, cryptochrome 1 | -3.372 | -1.290 | -1.038 |
| 5 | 20931 | bx030031 | AT3G02645 | Plant protein of unknown function (DUF247) | -2.380 | -1.239 | -0.869 |
| 5 | 21034 | bx030174 | AT1G47960 | cell wall / vacuolar inhibitor of fructosidase 1 | -4.136 | -2.666 | -1.337 |
| 5 | 21406 | bx030688 | AT5G01880 | RING/U-box superfamily protein | -4.121 | -1.023 | 0.556 |
| 5 | 21523 | bx030848 | AT4G29090 | Ribonuclease H-like superfamily protein | -3.810 | -1.939 | -1.342 |
| 5 | 21565 | bx030898 | AT1G74070 | Cyclophilin-like peptidyl-prolyl cis-trans isomerase family protein | -2.414 | -1.328 | -1.146 |
| 5 | 21727 | bx031123 |  |  | -2.415 | -0.689 | -1.207 |
| 5 | 21952 | bx031436 |  |  | -2.439 | -1.351 | -1.352 |
| 5 | 22228 | bx031830 |  |  | -2.465 | -1.687 | -1.198 |
| 5 | 22761 | bx032596 | AT5G01260 | Carbohydrate-binding-like fold | -2.392 | -1.052 | -0.487 |
| 5 | 22849 | bx032721 | AT1G08570 | atypical CYS HIS rich thioredoxin 4, atypical CYS HIS rich thioredoxin 4 | -2.485 | -0.700 | -1.135 |
| 5 | 22884 | bx032779 | AT1G12370 | UV RESISTANCE 2, photolyase 1 | -2.562 | -2.095 | -0.974 |
| 5 | 22997 | bx032938 | AT1G10070 | branched-chain amino acid transaminase 2 | -2.459 | -1.152 | 0.057 |
| 5 | 23297 | bx033371 | AT1G77380 | amino acid permease 3 | -3.380 | -2.401 | -0.911 |
| 5 | 23454 | bx033602 | AT3G45600 | tetraspanin3 | -2.979 | -1.889 | -1.407 |
| 5 | 23502 | bx033670 | AT1G01380 | ENHANCER OF TRY AND CPC 1, Homeodomain-like superfamily protein | -2.476 | -1.847 | -0.249 |
| 5 | 23641 | bx033864 | AT1G35430 | unknown protein | -2.987 | -1.992 | -0.153 |
| 5 | 23654 | bx033882 | AT5G12950 | Putative glycosyl hydrolase of unknown function (DUF1680) | -2.461 | -0.772 | -1.070 |
| 5 | 23793 | bx034075 | AT5G01240 | like AUXIN RESISTANT 1 | -2.766 | -1.268 | -1.607 |
| 5 | 23889 | bx034213 |  |  | -2.826 | -1.694 | -0.981 |
| 5 | 23914 | bx034255 | AT5G40240 | nodulin MtN21 /EamA-like transporter family protein | -2.404 | -1.668 | -0.480 |
| 5 | 24193 | bx034653 | AT2G13360 | L-serine:glyoxylate aminotransferase, ALANINE:GLYOXYLATE AMINOTRANSFERASE 1, alanine:glyoxylate aminotransferase | -3.173 | -2.393 | -1.066 |
| 5 | 24321 | bx034847 | AT4G25000 | alpha-amylase-like | -2.356 | -1.091 | 1.626 |
| 5 | 24662 | bx035340 | AT3G22210 | unknown protein | -2.699 | -2.098 | -1.091 |
| 5 | 24917 | bx035695 | AT1G60550 | enoyl-CoA hydratase/isomerase D | -2.338 | -1.920 | -0.895 |
| 5 | 24919 | bx035697 | AT1G28100 | unknown protein | -4.128 | -2.818 | -1.175 |
| 5 | 25028 | bx035861 | AT4G14746 | unknown protein | -2.603 | -1.441 | -0.825 |
| 5 | 25040 | bx035876 | AT5G51660 | cleavage and polyadenylation specificity factor 160 | -3.478 | -2.467 | -1.659 |
| 5 | 25405 | bx036393 | AT3G15510 | NAC-REGULATED SEED MORPHOLOGY 1, NAC domain containing protein 2, Arabidopsis NAC domain containing protein 56, NAC domain containing protein 2 | -2.413 | -1.406 | -1.388 |
| 5 | 25754 | bx036948 | AT1G01380 | ENHANCER OF TRY AND CPC 1, Homeodomain-like superfamily protein | -2.532 | -1.845 | -0.254 |
| 5 | 25979 | bx037300 | AT5G65760 | Serine carboxypeptidase S28 family protein | -2.927 | -2.270 | -0.985 |
| 5 | 26043 | bx037399 | AT3G26070 | Plastid-lipid associated protein PAP / fibrillin family protein | -2.566 | -1.659 | -0.943 |
| 5 | 26227 | bx037707 | AT4G26530 | Aldolase superfamily protein | -3.226 | -2.516 | -1.283 |
| 5 | 26733 | bx038390 |  |  | -2.363 | -1.321 | -1.186 |
| 5 | 26810 | bx038492 | AT5G17050 | UDP-glucosyl transferase 78D2 | -2.746 | -1.992 | -0.528 |
| 5 | 26841 | bx038531 | AT2G36690 | 2-oxoglutarate (2OG) and Fe(II)-dependent oxygenase superfamily protein | -2.668 | -0.530 | -0.743 |
| 5 | 26961 | bx038689 | AT3G15030 | maternal effect embryo arrest 35, TCP family transcription factor 4 | -2.430 | -0.569 | -2.012 |
| 5 | 27035 | bx038783 | AT2G04039 | unknown protein | -3.492 | -2.439 | -1.113 |
| 5 | 27239 | bx039054 | AT5G56340 | RING/U-box superfamily protein | -2.718 | -1.590 | -1.331 |
| 5 | 27248 | bx039065 | AT1G01320 | Tetratricopeptide repeat (TPR)-like superfamily protein | -2.778 | -1.538 | -0.870 |
| 5 | 27250 | bx039067 | AT1G15520 | Arabidopsis thaliana ATP-binding cassette G40, ATP-binding cassette G40, pleiotropic drug resistance 12 | -2.688 | -0.646 | -1.506 |
| 5 | 27337 | bx039186 | AT5G15802 | unknown protein | -2.545 | -1.592 | -1.070 |
| 5 | 27548 | bx039451 | AT2G28690 | Protein of unknown function (DUF1635) | -2.403 | -1.766 | -0.662 |
| 5 | 27617 | bx039542 | AT4G32480 | Protein of unknown function (DUF506) | -3.701 | -2.430 | -1.134 |
| 5 | 28055 | bx040118 | AT4G23160 | cysteine-rich RLK (RECEPTOR-like protein kinase) 8 | -2.328 | -1.514 | -1.106 |
| 5 | 28346 | bx040482 | AT3G15510 | NAC-REGULATED SEED MORPHOLOGY 1, NAC domain containing protein 2, Arabidopsis NAC domain containing protein 56, NAC domain containing protein 2 | -3.059 | -0.887 | -2.120 |
| 5 | 28823 | bx041096 | AT1G80130 | Tetratricopeptide repeat (TPR)-like superfamily protein | -3.072 | -2.482 | -0.508 |
| 5 | 29180 | bx041545 |  |  | -2.382 | -1.373 | -1.248 |
| 5 | 29222 | bx041592 | AT5G39210 | chlororespiratory reduction 7 | -2.677 | -1.468 | -1.142 |
| 5 | 29571 | bx042047 |  |  | -3.067 | -1.235 | -1.143 |
| 5 | 29606 | bx042090 | AT5G12950 | Putative glycosyl hydrolase of unknown function (DUF1680) | -2.411 | -0.806 | -1.041 |
| 5 | 29821 | bx042359 | AT1G47960 | cell wall / vacuolar inhibitor of fructosidase 1 | -3.540 | -2.711 | -0.523 |
| 5 | 30015 | bx042591 |  |  | -2.351 | -0.717 | -1.526 |
| 5 | 30298 | bx042957 | AT1G28100 | unknown protein | -3.535 | -2.482 | -1.023 |
| 5 | 30877 | bx043706 | AT5G65210 | bZIP transcription factor family protein | -2.550 | -1.323 | -1.100 |
| 5 | 31189 | bx044104 | AT5G53580 | pyridoxal reductase 1, NAD(P)-linked oxidoreductase superfamily protein | -2.415 | -1.794 | -0.962 |
| 5 | 31454 | bx044446 | AT2G02850 | plantacyanin | -2.965 | 1.059 | 0.577 |
| 5 | 31480 | bx044477 | AT1G80530 | Major facilitator superfamily protein | -2.597 | -1.347 | -1.126 |
| 5 | 31493 | bx044494 | AT5G15780 | Pollen Ole e 1 allergen and extensin family protein | -3.926 | -1.905 | -1.291 |
| 5 | 32193 | bx045370 | AT5G66190 | ferredoxin-NADP(+)-oxidoreductase 1, LEAF FNR 1, ferredoxin-NADP(+)-oxidoreductase 1 | -3.648 | -2.516 | -1.411 |
| 5 | 32204 | bx045386 | AT5G45310 | unknown protein | -3.722 | -2.760 | -0.869 |
| 5 | 32338 | bx045559 | AT3G25690 | Arabidopsis thaliana CHLOROPLAST UNUSUAL POSITIONING 1, Hydroxyproline-rich glycoprotein family protein | -2.503 | -1.932 | -0.826 |
| 5 | 32585 | bx045866 |  |  | -2.542 | -1.697 | -1.313 |
| 5 | 32615 | bx045898 | AT1G21280 | unknown protein | -2.934 | -0.549 | -2.613 |
| 5 | 32713 | bx046017 | AT5G05280 | RING/U-box superfamily protein | -2.674 | -0.202 | -0.164 |
| 5 | 33256 | bx046704 | AT1G43730 | RNA-directed DNA polymerase (reverse transcriptase)-related family protein | -2.314 | -1.348 | -0.790 |
| 5 | 33626 | bx047166 |  |  | -2.906 | -0.417 | -0.904 |
| 5 | 33664 | bx047210 | AT3G03080 | Zinc-binding dehydrogenase family protein | -2.527 | -1.481 | -1.485 |
| 5 | 33817 | bx047395 | AT5G38880 | unknown protein | -2.064 | -2.696 | 1.335 |
| 5 | 34186 | bx047868 | AT5G62360 | Plant invertase/pectin methylesterase inhibitor superfamily protein | -3.284 | -1.686 | -1.156 |
| 5 | 34264 | bx047965 | AT3G28345 | multi-drug resistance 13, ATP-binding cassette B15, ABC transporter family protein | -2.616 | -0.079 | -1.164 |
| 5 | 34477 | bx048230 | AT1G04920 | sucrose phosphate synthase 3F | -2.720 | -1.748 | -0.008 |
| 5 | 34619 | bx048404 |  |  | -2.547 | -0.148 | -2.082 |
| 5 | 34851 | bx048692 | AT1G05500 | synaptotagmin 5, ARABIDOPSIS THALIANA SYNAPTOTAGMIN HOMOLOG E, Calcium-dependent lipid-binding (CaLB domain) family protein | -2.432 | -1.658 | -0.574 |
| 5 | 35151 | bx049071 | AT4G29090 | Ribonuclease H-like superfamily protein | -3.791 | -1.859 | -1.366 |
| 5 | 35271 | bx049225 | AT5G50790 | Nodulin MtN3 family protein | -3.888 | -1.958 | 0.163 |
| 5 | 35459 | bx049459 | AT2G22660 | Protein of unknown function (duplicated DUF1399) | -2.393 | 0.200 | -0.234 |
| 5 | 35519 | bx049548 | AT5G54530 | Protein of unknown function, DUF538 | -2.602 | -1.095 | -1.231 |
| 5 | 35544 | bx049580 | AT5G14920 | Gibberellin-regulated family protein | -2.598 | -1.842 | -0.682 |
| 5 | 35940 | bx050112 | AT1G08230 | L-GAMMA-AMINOBUTYRIC ACID TRANSPORTER 1, Transmembrane amino acid transporter family protein | -3.047 | -1.676 | -1.213 |
| 5 | 36185 | bx050406 | AT1G09380 | nodulin MtN21 /EamA-like transporter family protein | -2.714 | -0.812 | -1.729 |
| 5 | 36706 | bx051052 | AT2G23610 | ARABIDOPSIS THALIANA METHYL ESTERASE 3, methyl esterase 3 | -2.838 | -0.844 | -1.088 |
| 5 | 36848 | bx051228 |  |  | -2.969 | -1.227 | -0.984 |
| 5 | 37006 | bx051446 | AT3G26070 | Plastid-lipid associated protein PAP / fibrillin family protein | -2.505 | -1.612 | -0.936 |
| 5 | 37174 | bx051666 |  |  | -2.397 | -1.402 | -1.235 |
| 5 | 37201 | bx051698 | AT4G20260 | ARABIDOPSIS THALIANA PLASMA-MEMBRANE ASSOCIATED CATION-BINDING PROTEIN 1, plasma-membrane associated cation-binding protein 1 | -2.988 | -1.563 | -0.365 |
| 5 | 37316 | bx051837 |  |  | -2.507 | -2.145 | -0.797 |
| 5 | 37689 | bx052300 | AT5G01260 | Carbohydrate-binding-like fold | -2.442 | -1.135 | -0.406 |
| 5 | 37852 | bx052515 | AT1G10070 | branched-chain amino acid transaminase 2 | -2.834 | -1.192 | -0.124 |
| 5 | 38135 | bx052893 | AT3G15510 | NAC-REGULATED SEED MORPHOLOGY 1, NAC domain containing protein 2, Arabidopsis NAC domain containing protein 56, NAC domain containing protein 2 | -2.541 | -1.449 | -1.360 |
| 5 | 38287 | bx053081 | AT2G36870 | xyloglucan endotransglucosylase/hydrolase 32 | -4.687 | -2.621 | -2.356 |
| 5 | 38324 | bx053132 | AT1G60590 | Pectin lyase-like superfamily protein | -5.073 | -0.606 | -1.207 |
| 5 | 38419 | bx053258 |  |  | -3.035 | -1.660 | -1.006 |
| 5 | 38447 | bx053293 | AT3G24670 | Pectin lyase-like superfamily protein | -2.512 | -1.237 | -1.257 |
| 5 | 38543 | bx053429 | AT3G56650 | Mog1/PsbP/DUF1795-like photosystem II reaction center PsbP family protein | -2.344 | -1.051 | -0.761 |
| 5 | 38616 | bx053513 | AT1G11120 | unknown protein | -4.285 | -1.637 | -1.814 |
| 5 | 38890 | bx053869 |  |  | -2.804 | -1.632 | -0.985 |
| 5 | 39521 | bx054737 | AT4G11360 | RING-H2 finger A1B | -2.922 | -0.034 | -0.201 |
| 5 | 39823 | bx055193 | AT3G11820 | PENETRATION1, SYNTAXIN RELATED PROTEIN 1, syntaxin of plants 121 | -3.249 | -0.352 | -0.456 |
| 5 | 40156 | bx055626 |  |  | -2.879 | -1.316 | -0.794 |
| 5 | 40205 | bx055686 | AT3G23740 | unknown protein | -1.340 | -2.700 | 1.301 |
| 5 | 40686 | bx056279 | AT5G40240 | nodulin MtN21 /EamA-like transporter family protein | -2.380 | -1.787 | -0.415 |
| 5 | 40990 | bx056649 | AT2G39370 | MEMBRANE-ASSOCIATED KINASE REGULATOR 4, unknown protein | -3.312 | -1.287 | -2.178 |
| 5 | 41187 | bx056884 |  |  | -2.561 | -1.509 | -1.238 |
| 5 | 41646 | bx057454 | AT2G15480 | UDP-glucosyl transferase 73B5 | -2.751 | -0.926 | -1.155 |
| 5 | 41690 | bx057505 | AT3G22970 | Protein of unknown function (DUF506) | -3.689 | -1.583 | -1.776 |
| 5 | 42325 | bx058270 | AT1G69370 | chorismate mutase 3 | -2.574 | -0.065 | -0.206 |
| 5 | 42352 | bx058298 |  |  | -2.448 | -1.137 | -1.285 |
| 5 | 42738 | bx058776 | AT3G06880 | Transducin/WD40 repeat-like superfamily protein | -2.477 | -0.829 | -1.207 |
| 5 | 42772 | bx058822 |  |  | -3.645 | -1.823 | -2.149 |
| 5 | 42927 | bx059016 |  |  | -2.503 | -1.511 | -0.387 |
| 5 | 43107 | bx059233 |  |  | -2.718 | 0.361 | -0.008 |
| 5 | 43461 | bx059675 | AT5G40270 | HD domain-containing metal-dependent phosphohydrolase family protein | -2.470 | -1.895 | -1.064 |
| 5 | 43562 | bx059801 | AT1G52190 | Major facilitator superfamily protein | -2.804 | 0.816 | -0.414 |
| 5 | 43626 | bx059884 |  |  | -3.310 | -1.989 | -1.756 |
| 5 | 43918 | bx060243 |  |  | -2.504 | -1.181 | -1.175 |
| 5 | 44230 | bx060610 |  |  | -3.120 | -0.671 | -0.996 |
| 5 | 44246 | bx060628 | AT5G23520 | smr (Small MutS Related) domain-containing protein | -2.496 | -0.209 | -0.709 |
| 5 | 44451 | bx060885 |  |  | -2.613 | -0.541 | -1.257 |
| 5 | 45151 | bx061750 |  |  | -2.610 | -1.388 | -1.464 |
| 5 | 45948 | bx062756 | AT5G40940 | putative fasciclin-like arabinogalactan protein 20 | -2.795 | -1.212 | -1.142 |
| 5 | 45949 | bx062757 | AT1G08800 | Protein of unknown function, DUF593 | -3.401 | -1.012 | -0.776 |
| 5 | 46032 | bx062875 |  |  | -2.573 | -1.432 | -1.217 |
| 5 | 46178 | bx063053 |  |  | -2.651 | -1.027 | -1.093 |
| 5 | 46228 | bx063119 | AT2G20142 | Toll-Interleukin-Resistance (TIR) domain family protein | -2.993 | -1.686 | -0.882 |
| 5 | 46588 | bx063569 | AT1G68520 | B-box type zinc finger protein with CCT domain | -2.957 | -1.617 | -1.547 |
| 5 | 46633 | bx063624 | AT5G52220 | unknown protein | -3.761 | -1.386 | -1.651 |
| 5 | 47137 | bx064203 |  |  | -2.750 | -1.369 | -1.589 |
| 5 | 47194 | bx064267 |  |  | -2.377 | -1.270 | -0.809 |
| 5 | 47373 | bx064469 | AT2G28690 | Protein of unknown function (DUF1635) | -2.412 | -1.843 | -0.737 |
| 5 | 48011 | bx065187 |  |  | -2.490 | -1.786 | -0.397 |
| 5 | 48512 | bx065801 | AT1G10070 | branched-chain amino acid transaminase 2 | -2.773 | -1.747 | -0.437 |
| 5 | 48812 | bx066187 | AT1G20330 | FRILL1, COTYLEDON VASCULAR PATTERN 1, sterol methyltransferase 2 | -2.812 | -0.384 | -1.038 |
| 5 | 49150 | bx066617 | AT4G32440 | Plant Tudor-like RNA-binding protein | -2.331 | -1.518 | -0.647 |
| 5 | 49213 | bx066691 | AT2G28080 | UDP-Glycosyltransferase superfamily protein | -3.155 | -1.086 | -1.204 |
| 5 | 49315 | bx066812 |  |  | -2.477 | -0.408 | -0.925 |
| 5 | 49347 | bx066849 | AT5G05280 | RING/U-box superfamily protein | -2.539 | -0.528 | 0.094 |
| 5 | 49350 | bx066853 | AT1G50180 | NB-ARC domain-containing disease resistance protein | -2.858 | -0.103 | 0.455 |
| 5 | 49430 | bx066943 |  |  | -2.568 | -1.170 | -1.029 |
| 5 | 49434 | bx066947 |  |  | -3.667 | -1.400 | -1.629 |
| 5 | 49791 | bx067372 | AT1G44100 | amino acid permease 5 | -3.220 | -2.244 | -0.665 |
| 5 | 49960 | bx067567 |  |  | -2.697 | -1.269 | -1.413 |
| 5 | 50804 | bx068564 |  |  | -2.893 | -1.952 | -1.379 |
| 5 | 51595 | bx069634 | AT1G11720 | starch synthase 3 | -2.628 | -2.204 | -0.761 |
| 5 | 51622 | bx069674 |  |  | -2.406 | -0.934 | -1.190 |
| 5 | 52067 | bx070347 | AT3G15510 | NAC-REGULATED SEED MORPHOLOGY 1, NAC domain containing protein 2, Arabidopsis NAC domain containing protein 56, NAC domain containing protein 2 | -2.452 | -1.383 | -1.320 |
| 5 | 52282 | bx070636 |  |  | -2.509 | 0.006 | 0.481 |
| 5 | 52524 | bx071027 | AT4G35100 | PLASMA MEMBRANE INTRINSIC PROTEIN 3A, PLASMA MEMBRANE INTRINSIC PROTEIN 2;7, plasma membrane intrinsic protein 3 | -2.645 | -2.582 | -0.035 |
| 5 | 52782 | bx071444 |  |  | -2.325 | 0.020 | -0.005 |
| 5 | 52921 | bx071651 |  |  | -2.374 | -1.451 | -1.338 |
| 5 | 53719 | bx072571 | AT1G44350 | IAA-leucine resistant (ILR)-like gene 6 | -2.338 | 0.033 | -0.310 |
| 5 | 54017 | bx072917 |  |  | -2.590 | -1.129 | -0.880 |
| 5 | 54202 | bx073125 | AT5G43530 | Helicase protein with RING/U-box domain | -2.542 | -1.339 | -0.908 |
| 5 | 54276 | bx073205 | AT3G16300 | Uncharacterised protein family (UPF0497) | -3.087 | -0.269 | -0.607 |
| 5 | 54988 | bx073998 |  |  | -2.417 | -0.973 | -1.047 |
| 5 | 55370 | bx074486 | AT1G43650 | nodulin MtN21 /EamA-like transporter family protein | -2.549 | -0.479 | 0.226 |
| 5 | 55456 | bx074602 |  |  | -2.388 | -1.194 | -0.848 |
| 5 | 55734 | bx075029 | AT4G12690 | Plant protein of unknown function (DUF868) | -3.110 | -1.303 | -1.693 |
| 5 | 56031 | bx075486 |  |  | -2.393 | -1.678 | -0.344 |
| 5 | 56544 | bx076149 |  |  | -4.928 | -1.937 | -3.156 |
| 5 | 56905 | bx076553 | AT2G21790 | CRINKLY LEAVES 8, RIBONUCLEOTIDE REDUCTASE LARGE SUBUNIT 1, ribonucleotide reductase 1 | -2.455 | -1.116 | -0.771 |
| 5 | 57216 | bx076927 |  |  | -3.541 | -1.939 | -1.099 |
| 5 | 57324 | bx077053 |  |  | -2.345 | -1.617 | -1.102 |
| 5 | 58001 | bx077924 |  |  | -2.992 | -1.588 | -1.583 |
| 5 | 58128 | bx078064 |  |  | -2.369 | -0.712 | -1.215 |
| 5 | 58168 | bx078108 | AT2G35635 | RELATED TO UBIQUITIN 2, ubiquitin 7 | -2.989 | -1.499 | -1.762 |
| 5 | 58242 | bx078189 |  |  | -3.022 | -1.746 | -1.319 |
| 5 | 58405 | bx078374 |  |  | -2.337 | -0.985 | -1.164 |
| 5 | 59038 | bx079135 |  |  | -2.710 | -1.295 | -1.226 |
| 5 | 59257 | bx079386 | AT2G43730 | Mannose-binding lectin superfamily protein | -2.731 | -1.208 | -1.794 |
| 5 | 59567 | bx079756 |  |  | -3.394 | -0.847 | -0.034 |
| 5 | 59573 | bx079764 | AT1G75260 | oxidoreductases, acting on NADH or NADPH | -2.351 | -0.387 | -1.746 |
| 5 | 59617 | bx079810 |  |  | -2.809 | -1.442 | -0.655 |
| 5 | 59667 | bx079866 | AT2G36830 | TONOPLAST INTRINSIC PROTEIN 1;1, GAMMA TONOPLAST INTRINSIC PROTEIN 1, gamma tonoplast intrinsic protein | -3.334 | -1.297 | -1.106 |
| 5 | 59905 | bx080166 |  |  | -2.345 | -1.192 | -0.997 |
| 5 | 59922 | bx080186 | AT1G73480 | alpha/beta-Hydrolases superfamily protein | -2.827 | -2.077 | -0.931 |
| 5 | 60002 | bx080298 | AT2G36870 | xyloglucan endotransglucosylase/hydrolase 32 | -2.573 | -0.530 | -1.030 |
| 5 | 60159 | bx080503 | AT3G27150 | Galactose oxidase/kelch repeat superfamily protein | -2.682 | -0.689 | 0.003 |
| 5 | 60476 | bx080915 | AT3G25690 | Arabidopsis thaliana CHLOROPLAST UNUSUAL POSITIONING 1, Hydroxyproline-rich glycoprotein family protein | -2.460 | -1.845 | -1.081 |
| 5 | 60619 | bx081096 |  |  | -2.785 | -1.312 | -1.365 |
| 5 | 60821 | bx081335 | AT2G23600 | ARABIDOPSIS THALIANA METHYL ESTERASE 2, ARABIDOPSIS METHYL ESTERASE 8, acetone-cyanohydrin lyase | -2.540 | -0.033 | 1.828 |
| 5 | 61579 | bx082423 |  |  | -3.662 | -1.490 | 4.348 |
| 5 | 61592 | bx082442 |  |  | -2.776 | -1.440 | -0.998 |
