# Supplementary material for: Transcriptome analysis of Pará rubber tree (H. brasiliensis) seedlings under ethylene stimulation
Source: BMC Plant Biol. 2021 Sep 13;21:420. doi: 10.1186/s12870-021-03196-y (PMC8436496; doi:10.1186/s12870-021-03196-y)
Supplement: Supplementary file 3 — Additional file 3 : Table S3. Corresponding probe sets for CPT, REF, and SRPP genes. [file 12870_2021_3196_MOESM3_ESM.docx]

| This study | | | | |
| --- | --- | --- | --- | --- |
| Bx probes | | ET/mock (Ln) | | |
| probes | Identities | 6 hr | 24 hr | 48 hr |
| no hit |  |  |  |  |
| no hit |  |  |  |  |
| BX006670 | 99% |  |  |  |
| BX014453 | 99% |  |  |  |
| BX030069 BX030484 | 99% 99% |  |  |  |
| BX042693 BX039191 | 99% 99% |  |  |  |
| BX017746 | 99% |  |  |  |
| BX037276 | 98% |  |  |  |
| no hit |  |  |  |  |
| BX046228 | 98% |  |  |  |
| no hit |  |  |  |  |
| BX022980 | 99% |  |  |  |
| no hit |  |  |  |  |
| BX002063 | 99% |  |  |  |
| no hit |  |  |  |  |
| BX001875 | 98% |  |  |  |
| no hit |  |  |  |  |
| BX017742 | 98% |  |  |  |
| BX001295 | 99% |  |  |  |
| BX032923 | 99% |  |  |  |
| BX020932 | 100% |  |  |  |
| BX059449 | 99% |  |  |  |
| BX045625 | 95% | 2.13 | 2.35 | 1.07 |
| BX019740 | 100% |  |  |  |
| BX041337 | 100% |  |  |  |
| BX000215 | 99% |  |  |  |
