# Supplementary material for: Transcriptome analysis of Pará rubber tree (H. brasiliensis) seedlings under ethylene stimulation
Source: BMC Plant Biol. 2021 Sep 13;21:420. doi: 10.1186/s12870-021-03196-y (PMC8436496; doi:10.1186/s12870-021-03196-y)
Supplement: Supplementary file 4 — Additional file 4 : Table S4. Probe lists for 234 differentially expressed genes encoding transcription factors. [file 12870_2021_3196_MOESM4_ESM.docx]

| Nakano et al. Supplemental Table 4. | | | |  |  |  |  |  |  |  |  |
| --- | --- | --- | --- | --- | --- | --- | --- | --- | --- | --- | --- |
|  |  | Arabidopsis homologue | | | Et/Mock (Ln) | | |  |  |  |  |
| **G** | **Probe ID** | **AGI** | **TF classification** | **Short description** | **6h** | **24h** | **48h** |  |  |  |  |
|  |  |  |  |  |  |  |  |  |  |  | Ln |
| 0 | bx020251 | AT3G15210 | AP2_ERF | ERF78 | 2.18 | 2.32 | 1.96 |  |  | > | 6.9 |
| 0 | bx017330 | AT3G16770 | AP2_ERF | ERF72 | 1.83 | 2.48 | 2.47 |  |  | > | 5.5 |
| 0 | bx022445 | AT3G16770 | AP2_ERF | ERF72 | 2.81 | 3.05 | 2.14 |  |  | > | 4.2 |
| 0 | bx038100 | AT3G23240 | AP2_ERF | ERF92 | 5.92 | 7.62 | 7.16 |  |  | > | 2.8 |
| 0 | bx040001 | AT3G23240 | AP2_ERF | ERF92 | 3.81 | 7.49 | 8.04 |  |  | > | 1.4 |
| 0 | bx058590 | AT3G23240 | AP2_ERF | ERF92 | 4.22 | 3.35 | 4.40 |  |  | > | -1.4 |
| 0 | bx023675 | AT4G11140 | AP2_ERF | ERF63 | 2.35 | 2.94 | 2.71 |  |  | < | -1.4 |
| 0 | bx039197 | AT4G17500 | AP2_ERF | ERF100 | 5.74 | 5.22 | 4.50 |  |  | < | -2.8 |
| 0 | bx039254 | AT4G17500 | AP2_ERF | ERF100 | 2.03 | 2.69 | 1.64 |  |  | < | -4.2 |
| 0 | bx046661 | AT4G17500 | AP2_ERF | ERF100 | 3.80 | 6.04 | 4.57 |  |  | < | -5.5 |
| 0 | bx011862 | AT5G43410 | AP2_ERF | ERF96 | 4.48 | 5.01 | 4.10 |  |  | < | -6.9 |
| 0 | bx037951 | AT5G61890 | AP2_ERF | ERF114 | 1.90 | 3.62 | 3.89 |  |  |  |  |
| 1 | bx071140 | AT3G23230 | AP2_ERF | ERF98 | 5.86 | 5.63 | 4.11 |  |  |  |  |
| 1 | bx027059 | AT3G23240 | AP2_ERF | ERF92 | 5.24 | 4.95 | 3.18 |  |  |  |  |
| 1 | bx031374 | AT5G50080 | AP2_ERF | ERF110 | 4.36 | 3.87 | 2.47 |  |  |  |  |
| 1 | bx046659 | AT5G51190 | AP2_ERF | ERF105 | 2.69 | 2.70 | 1.40 |  |  |  |  |
| 2 | bx047672 | AT2G44940 | AP2_ERF | ERF34 | -3.57 | -3.48 | -1.37 |  |  |  |  |
| 2 | bx024041 | AT5G11590 | AP2_ERF | ERF41 | -2.40 | -2.48 | -2.27 |  |  |  |  |
| 3 | bx059815 | AT4G37750 | AP2_ERF | - | -2.35 | -2.46 | -3.53 |  |  |  |  |
| 3 | bx007515 | AT5G10510 | AP2_ERF | - | -2.37 | -2.32 | -3.60 |  |  |  |  |
| 3 | bx038544 | AT5G25190 | AP2_ERF | ERF3 | -2.21 | -1.39 | -2.65 |  |  |  |  |
| 3 | bx046468 | AT5G25190 | AP2_ERF | ERF3 | -1.20 | -1.38 | -2.49 |  |  |  |  |
|  |  |  |  | *ERF No and group from Nakano et al. (2006) |  |  |  |  |  |  |  |
|  |  |  |  |  |  |  |  |  |  |  |  |
| 1 | bx012856 | AT1G25560 | AP2/B3 | TEMPRANILLO 1, ETHYLENE RESPONSE DNA BINDING FACTOR 1, AP2/B3 transcription factor family protein | 3.10 | 3.12 | 2.00 |  |  |  |  |
|  |  |  |  |  |  |  |  |  |  |  |  |
| 0 | bx041424 | AT5G62000 | ARF | ORESARA 14, HLS1 SUPPRESSOR, ARF1-BINDING PROTEIN, auxin response factor 2 | 3.97 | 5.47 | 4.94 |  |  |  |  |
|  |  |  |  |  |  |  |  |  |  |  |  |
| 3 | bx020778 | AT1G04880 | ARID | HMG (high mobility group) box protein with ARID/BRIGHT DNA-binding domain | -2.49 | -2.96 | -3.22 |  |  |  |  |
|  |  |  |  |  |  |  |  |  |  |  |  |
| 0 | bx034731 | AT1G07900 | AS2 | LOB domain-containing protein 1 | 1.34 | 1.86 | 4.04 |  |  |  |  |
| 1 | bx029892 | AT3G02550 | AS2 | LOB domain-containing protein 41 | 3.56 | 2.33 | 0.21 |  |  |  |  |
| 2 | bx064672 | AT1G31320 | AS2 | LOB domain-containing protein 4 | -2.58 | -2.73 | -2.90 |  |  |  |  |
| 4 | bx002551 | AT2G30340 | AS2 | LOB domain-containing protein 13 | -1.40 | -1.06 | -2.66 |  |  |  |  |
| 4 | bx003930 | AT2G30340 | AS2 | LOB domain-containing protein 13 | -1.97 | -0.77 | -2.58 |  |  |  |  |
|  |  |  |  |  |  |  |  |  |  |  |  |
| 0 | bx002859 | AT5G49700 | AT-hook | Predicted AT-hook DNA-binding family protein | 2.11 | 3.98 | 3.03 |  |  |  |  |
| 0 | bx028511 | AT5G49700 | AT-hook | Predicted AT-hook DNA-binding family protein | 1.35 | 3.17 | 3.25 |  |  |  |  |
| 1 | bx007431 | AT4G22810 | AT-hook | Predicted AT-hook DNA-binding family protein | 2.59 | 2.92 | 0.37 |  |  |  |  |
| 4 | bx007852 | AT2G45850 | AT-hook | AT hook motif DNA-binding family protein | -0.59 | -0.94 | -2.53 |  |  |  |  |
| 4 | bx044175 | AT2G45850 | AT-hook | AT hook motif DNA-binding family protein | -0.59 | -0.94 | -2.47 |  |  |  |  |
|  |  |  |  |  |  |  |  |  |  |  |  |
| 0 | bx043133 | AT4G32280 | AUX_IAA | IAA29 | 2.66 | 2.94 | 1.95 |  |  |  |  |
| 2 | bx072986 | AT4G29080 | AUX_IAA | IAA27/PAP2 | -2.07 | -2.43 | -2.35 |  |  |  |  |
| 3 | bx056394 | AT1G04250 | AUX_IAA | AXR3 | -1.13 | -1.89 | -2.60 |  |  |  |  |
| 3 | bx076309 | AT2G22670 | AUX_IAA | IAA8 | -1.51 | -2.22 | -2.53 |  |  |  |  |
| 3 | bx037081 | AT2G33310 | AUX_IAA | IAA13 | -1.61 | -2.16 | -2.34 |  |  |  |  |
| 3 | bx048138 | AT3G04730 | AUX_IAA | IAA16 | 0.12 | -3.65 | -3.40 |  |  |  |  |
| 3 | bx025693 | AT4G14550 | AUX_IAA | IAA14 | -1.80 | -3.68 | -5.22 |  |  |  |  |
| 3 | bx046249 | AT4G14550 | AUX_IAA | IAA14 | -1.69 | -3.24 | -4.20 |  |  |  |  |
| 3 | bx033335 | AT4G29080 | AUX_IAA | IAA27/PAP2 | -1.38 | -2.06 | -2.53 |  |  |  |  |
| 3 | bx004053 | AT5G65670 | AUX_IAA | IAA9 | -1.31 | -2.00 | -2.70 |  |  |  |  |
| 4 | bx045575 | AT3G15540 | AUX_IAA | IAA19 | -0.08 | -1.91 | -3.47 |  |  |  |  |
|  |  |  |  | *IAA No from PlnTFDB (http://plntfdb.bio.uni-potsdam.de/v3.0/). | | | |  |  |  |  |
|  |  |  |  |  |  |  |  |  |  |  |  |
| 2 | bx081388 | AT3G19184 | B3 | AP2/B3-like transcriptional factor family protein | -1.76 | -3.15 | -1.78 |  |  |  |  |
| 2 | bx039725 | AT4G33280 | B3 | AP2/B3-like transcriptional factor family protein | -2.40 | -1.28 | -1.66 |  |  |  |  |
|  |  |  |  |  |  |  |  |  |  |  |  |
| 1 | bx003059 | AT5G46760 | bHLH | bHLH005 | 3.00 | 2.99 | 2.16 |  |  |  |  |
| 2 | bx008305 | AT1G09530 | bHLH | bHLH008 | -3.17 | -3.55 | -2.26 |  |  |  |  |
| 0 | bx013190 | AT4G00870 | bHLH | bHLH014 | 2.62 | 3.58 | 1.92 |  |  |  |  |
| 1 | bx045859 | AT4G00870 | bHLH | bHLH014 | 5.74 | 4.13 | 3.22 |  |  |  |  |
| 3 | bx057435 | AT4G36930 | bHLH | bHLH024 | -1.53 | -2.60 | -2.71 |  |  |  |  |
| 0 | bx037851 | AT1G68810 | bHLH | bHLH030 | 2.38 | 3.17 | 3.59 |  |  |  |  |
| 0 | bx054937 | AT1G68810 | bHLH | bHLH030 | 2.45 | 3.33 | 3.70 |  |  |  |  |
| 1 | bx049220 | AT5G56960 | bHLH | bHLH041 | 4.65 | 3.14 | -1.38 |  |  |  |  |
| 0 | bx011999 | AT1G73830 | bHLH | bHLH050 | 1.83 | 4.06 | 3.59 |  |  |  |  |
| 0 | bx051375 | AT1G73830 | bHLH | bHLH050 | 1.53 | 3.39 | 3.39 |  |  |  |  |
| 3 | bx007288 | AT1G10120 | bHLH | bHLH074 | -1.94 | -2.21 | -3.27 |  |  |  |  |
| 5 | bx008972 | AT5G65640 | bHLH | bHLH093 | -3.55 | -1.58 | -1.62 |  |  |  |  |
| 1 | bx015339 | AT2G42280 | bHLH | bHLH130 | 2.81 | 2.75 | 1.66 |  |  |  |  |
| 1 | bx047705 | AT2G42280 | bHLH | bHLH130 | 2.59 | 2.43 | 1.24 |  |  |  |  |
| 0 | bx081277 | AT4G38070 | bHLH | bHLH131-like | 2.74 | 2.55 | 2.02 |  |  |  |  |
| 3 | bx048163 | AT5G01310 | bHLH | bHLH140 | -2.72 | -2.86 | -3.98 |  |  |  |  |
| 2 | bx021419 | AT2G43060 | bHLH | bHLH158 | -3.04 | -2.30 | -1.89 |  |  |  |  |
| 2 | bx066022 | AT2G43060 | bHLH | bHLH158 | -2.99 | -2.31 | -2.11 |  |  |  |  |
| 0 | bx037801 | AT4G20970 | bHLH | bHLH162 | 2.04 | 2.76 | 1.98 |  |  |  |  |
| 1 | bx033064 | AT4G20970 | bHLH | bHLH162 | 2.75 | 2.92 | 1.53 |  |  |  |  |
| 1 | bx075296 | AT4G20970 | bHLH | bHLH162 | 2.45 | 2.88 | 1.61 |  |  |  |  |
| 3 | bx013378 | AT4G20970 | bHLH | bHLH162 | -1.76 | -2.63 | -2.16 |  |  |  |  |
| 0 | bx009188 | AT1G10586 | bHLH | bHLH DNA-binding superfamily protein | 1.91 | 2.72 | 1.94 |  |  |  |  |
| 2 | bx066207 | AT3G28857 | bHLH | Paclobutrazol Resistance 5 | -4.31 | -2.67 | -3.31 |  |  |  |  |
|  |  |  |  | *bHLH No from TAIR (http://www.arabidopsis.org/index.jsp). | | |  |  |  |  |  |
|  |  |  |  |  |  |  |  |  |  |  |  |
| 2 | bx036938 | AT1G22070 | bZIP | TGA1A-related gene 3 | -1.87 | -2.49 | -2.21 |  |  |  |  |
| 3 | bx018272 | AT1G77920 | bZIP | bZIP transcription factor family protein | -1.81 | -2.50 | -2.34 |  |  |  |  |
| 3 | bx045144 | AT1G77920 | bZIP | bZIP transcription factor family protein | -1.89 | -2.56 | -2.29 |  |  |  |  |
| 3 | bx039253 | AT3G30530 | bZIP | basic leucine-zipper 42 | -3.43 | -3.45 | -4.27 |  |  |  |  |
| 5 | bx043706 | AT5G65210 | bZIP | bZIP transcription factor family protein | -2.55 | -1.32 | -1.10 |  |  |  |  |
|  |  |  |  |  |  |  |  |  |  |  |  |
| 0 | bx027026 | AT1G02030 | C2H2ZnF | C2H2-like zinc finger protein | 0.13 | 0.74 | 2.51 |  |  |  |  |
| 0 | bx017773 | AT5G03740 | C2H2ZnF | HISTONE DEACETYLASE 3, histone deacetylase 2C | 1.82 | 1.84 | 3.53 |  |  |  |  |
| 1 | bx038394 | AT1G10480 | C2H2ZnF | zinc finger protein 5 | 4.03 | 3.08 | 2.17 |  |  |  |  |
| 1 | bx002147 | AT1G27730 | C2H2ZnF | salt tolerance zinc finger | 3.11 | 2.02 | 1.43 |  |  |  |  |
| 1 | bx029719 | AT1G27730 | C2H2ZnF | salt tolerance zinc finger | 3.19 | 2.46 | 2.01 |  |  |  |  |
| 1 | bx030935 | AT1G27730 | C2H2ZnF | salt tolerance zinc finger | 3.75 | 3.99 | 1.76 |  |  |  |  |
| 1 | bx058721 | AT1G27730 | C2H2ZnF | salt tolerance zinc finger | 3.17 | 2.46 | 2.27 |  |  |  |  |
| 2 | bx075420 | AT1G75710 | C2H2ZnF | C2H2-like zinc finger protein | -2.27 | -2.40 | -1.76 |  |  |  |  |
| 2 | bx027948 | AT2G29660 | C2H2ZnF | zinc finger (C2H2 type) family protein | -2.67 | -2.52 | -1.69 |  |  |  |  |
| 3 | bx059042 | AT1G02040 | C2H2ZnF | C2H2-type zinc finger family protein | -2.03 | -2.10 | -3.57 |  |  |  |  |
| 3 | bx027508 | AT2G28200 | C2H2ZnF | C2H2-type zinc finger family protein | -1.38 | -2.12 | -2.82 |  |  |  |  |
| 3 | bx009165 | AT2G42410 | C2H2ZnF | zinc finger protein 11 | -2.51 | -2.29 | -2.89 |  |  |  |  |
| 2 | bx004006 | AT2G05160 | C3HZnF | CCCH-type zinc fingerfamily protein with RNA-binding domain | -2.15 | -2.60 | -2.29 |  |  |  |  |
| 2 | bx009491 | AT2G19810 | C3HZnF | Oxidation-related Zinc Finger 1, CCCH-type zinc finger family protein | -3.09 | -3.13 | -2.33 |  |  |  |  |
| 4 | bx009240 | AT1G68200 | C3HZnF | Zinc finger C-x8-C-x5-C-x3-H type family protein | -1.19 | -2.35 | -5.09 |  |  |  |  |
|  |  |  |  |  |  |  |  |  |  |  |  |
| 2 | bx010326 | AT5G47640 | CCAAT | nuclear factor Y, subunit B2, nuclear factor Y, subunit B2 | -3.15 | -2.42 | -1.95 |  |  |  |  |
|  |  |  |  |  |  |  |  |  |  |  |  |
| 1 | bx043267 | AT4G27310 | CO | B-box type zinc finger family protein | 3.48 | 1.38 | 0.54 |  |  |  |  |
| 2 | bx021182 | AT1G68520 | CO | B-box type zinc finger protein with CCT domain | -3.04 | -2.10 | -1.74 |  |  |  |  |
| 2 | bx035599 | AT1G68520 | CO | B-box type zinc finger protein with CCT domain | -3.00 | -2.11 | -1.75 |  |  |  |  |
| 3 | bx012789 | AT1G68520 | CO | B-box type zinc finger protein with CCT domain | -2.57 | -2.64 | -3.43 |  |  |  |  |
| 5 | bx063569 | AT1G68520 | CO | B-box type zinc finger protein with CCT domain | -2.96 | -1.62 | -1.55 |  |  |  |  |
| 5 | bx024066 | AT4G38960 | CO | B-box type zinc finger family protein | -2.32 | -1.26 | -1.07 |  |  |  |  |
|  |  |  |  |  |  |  |  |  |  |  |  |
| 2 | bx003523 | AT4G14770 | CPP | TESMIN/TSO1-like CXC 2 | -2.80 | -2.10 | -2.30 |  |  |  |  |
| 2 | bx014620 | AT4G14770 | CPP | TESMIN/TSO1-like CXC 2 | -3.23 | -2.83 | -2.64 |  |  |  |  |
|  |  |  |  |  |  |  |  |  |  |  |  |
| 1 | bx008613 | AT1G47655 | DOF | Dof-type zinc finger DNA-binding family protein | 2.45 | 1.99 | -0.46 |  |  |  |  |
| 2 | bx042204 | AT1G21340 | DOF | Dof-type zinc finger DNA-binding family protein | -3.13 | -3.98 | -3.24 |  |  |  |  |
|  |  |  |  |  |  |  |  |  |  |  |  |
| 3 | bx038652 | AT3G04030 | GARP | Homeodomain-like superfamily protein | -1.73 | -2.21 | -2.73 |  |  |  |  |
| 3 | bx066742 | AT5G06800 | GARP | myb-like HTH transcriptional regulator family protein | -1.70 | -1.51 | -2.43 |  |  |  |  |
| 4 | bx050010 | AT4G28610 | GARP | phosphate starvation response 1, phosphate starvation response 1 | -2.21 | -1.61 | -4.57 |  |  |  |  |
|  |  |  |  |  |  |  |  |  |  |  |  |
| 4 | bx047280 | AT5G25830 | GATA | GATA transcription factor 12 | -2.14 | -1.72 | -4.50 |  |  |  |  |
|  |  |  |  |  |  |  |  |  |  |  |  |
| 0 | bx062687 | AT1G07530 | GRAS | ARABIDOPSIS THALIANA GRAS (GAI, RGA, SCR) 2, SCARECROW-like 14 | 5.88 | 5.92 | 5.01 |  |  |  |  |
| 0 | bx059468 | AT1G50420 | GRAS | scarecrow-like 3 | 4.01 | 3.60 | 3.61 |  |  |  |  |
| 0 | bx039931 | AT2G37650 | GRAS | GRAS family transcription factor | 2.42 | 4.69 | 4.37 |  |  |  |  |
| 1 | bx008127 | AT1G07530 | GRAS | ARABIDOPSIS THALIANA GRAS (GAI, RGA, SCR) 2, SCARECROW-like 14 | 2.73 | 2.76 | 1.12 |  |  |  |  |
| 1 | bx050228 | AT1G07530 | GRAS | ARABIDOPSIS THALIANA GRAS (GAI, RGA, SCR) 2, SCARECROW-like 14 | 2.80 | 2.76 | 1.17 |  |  |  |  |
| 1 | bx043871 | AT1G66350 | GRAS | RGA-like 1 | 1.93 | 2.65 | 1.27 |  |  |  |  |
| 3 | bx055439 | AT1G07530 | GRAS | ARABIDOPSIS THALIANA GRAS (GAI, RGA, SCR) 2, SCARECROW-like 14 | -1.89 | -3.80 | -3.82 |  |  |  |  |
| 3 | bx009425 | AT1G63100 | GRAS | GRAS family transcription factor | -1.55 | -2.77 | -2.97 |  |  |  |  |
|  |  |  |  |  |  |  |  |  |  |  |  |
| 2 | bx047542 | AT3G13960 | GRF | growth-regulating factor 5 | -2.25 | -3.17 | -2.70 |  |  |  |  |
| 2 | bx048029 | AT4G24150 | GRF | growth-regulating factor 8 | -3.65 | -3.19 | -3.85 |  |  |  |  |
|  |  |  |  |  |  |  |  |  |  |  |  |
| 0 | bx037742 | AT5G53980 | HD | homeobox protein 52 | 3.44 | 5.33 | 3.61 |  |  |  |  |
| 0 | bx078053 | AT5G53980 | HD | homeobox protein 52 | 2.67 | 4.03 | 3.94 |  |  |  |  |
| 2 | bx006987 | AT4G32980 | HD | homeobox gene 1 | -3.27 | -3.33 | -2.85 |  |  |  |  |
| 2 | bx010125 | AT4G32980 | HD | homeobox gene 1 | -2.49 | -3.11 | -2.64 |  |  |  |  |
| 2 | bx010607 | AT4G32980 | HD | homeobox gene 1 | -2.61 | -3.23 | -2.95 |  |  |  |  |
| 2 | bx079985 | AT4G32980 | HD | homeobox gene 1 | -3.43 | -3.41 | -2.99 |  |  |  |  |
| 3 | bx008014 | AT1G62360 | HD | WALDMEISTER 1, WALDMEISTER, SHOOT MERISTEMLESS, SHOOTLESS, BUMBERSHOOT 1, BUMBERSHOOT, KNOX/ELK homeobox transcription factor | -1.86 | -2.54 | -2.80 |  |  |  |  |
| 3 | bx061715 | AT1G62360 | HD | WALDMEISTER 1, WALDMEISTER, SHOOT MERISTEMLESS, SHOOTLESS, BUMBERSHOOT 1, BUMBERSHOOT, KNOX/ELK homeobox transcription factor | -1.72 | -2.25 | -2.47 |  |  |  |  |
| 3 | bx061187 | AT1G69780 | HD | Homeobox-leucine zipper protein family | -1.70 | -2.43 | -2.45 |  |  |  |  |
| 3 | bx055431 | AT3G01220 | HD | homeobox protein 20 | -1.79 | -2.74 | -2.64 |  |  |  |  |
| 3 | bx024615 | AT5G06710 | HD | homeobox from Arabidopsis thaliana | -2.72 | -3.00 | -3.60 |  |  |  |  |
|  |  |  |  |  |  |  |  |  |  |  |  |
| 1 | bx060134 | AT4G18880 | HSF | ARABIDOPSIS THALIANA HEAT SHOCK TRANSCRIPTION FACTOR A4A, heat shock transcription factor A4A | 4.29 | 4.34 | 1.90 |  |  |  |  |
| 3 | bx056835 | AT1G46264 | HSF | SCHIZORIZA, heat shock transcription factor B4, heat shock transcription factor B4 | -1.66 | -2.12 | -2.34 |  |  |  |  |
|  |  |  |  |  |  |  |  |  |  |  |  |
| 2 | bx024130 | AT1G01780 | LIM | PLIM2b, GATA type zinc finger transcription factor family protein | -4.31 | -4.32 | -4.68 |  |  |  |  |
| 2 | bx034470 | AT3G61230 | LIM | PLIM2c, GATA type zinc finger transcription factor family protein | -4.23 | -4.40 | -4.31 |  |  |  |  |
| 2 | bx040669 | AT3G61230 | LIM | PLIM2c, GATA type zinc finger transcription factor family protein | -4.10 | -4.44 | -4.67 |  |  |  |  |
| 3 | bx026799 | AT1G10200 | LIM | WLIM1, GATA type zinc finger transcription factor family protein | -1.67 | -1.79 | -2.89 |  |  |  |  |
| 3 | bx039527 | AT1G10200 | LIM | WLIM1, GATA type zinc finger transcription factor family protein | -1.55 | -1.42 | -2.31 |  |  |  |  |
| 3 | bx071006 | AT3G61230 | LIM | PLIM2c, GATA type zinc finger transcription factor family protein | -3.78 | -4.29 | -4.62 |  |  |  |  |
| 4 | bx041336 | AT1G01780 | LIM | PLIM2b, GATA type zinc finger transcription factor family protein | -1.82 | -2.91 | -5.28 |  |  |  |  |
|  |  |  |  |  |  |  |  |  |  |  |  |
| 3 | bx041571 | AT4G38620 | MYB | MYB004 | -2.23 | -4.28 | -4.80 |  |  |  |  |
| 1 | bx046963 | AT2G31180 | MYB | MYB014 | 3.15 | 3.70 | 1.87 |  |  |  |  |
| 0 | bx079230 | AT3G23250 | MYB | MYB015 | 3.99 | 5.72 | 3.08 |  |  |  |  |
| 1 | bx037814 | AT3G23250 | MYB | MYB015 | 2.36 | 0.21 | -0.93 |  |  |  |  |
| 1 | bx054306 | AT3G23250 | MYB | MYB015 | 5.38 | 5.85 | 3.60 |  |  |  |  |
| 4 | bx072328 | AT1G66230 | MYB | MYB020 | -0.48 | -1.01 | -2.38 |  |  |  |  |
| 4 | bx001992 | AT4G12350 | MYB | MYB042 | -1.67 | -2.27 | -4.90 |  |  |  |  |
| 4 | bx072880 | AT4G12350 | MYB | MYB042 | -1.67 | -1.65 | -3.35 |  |  |  |  |
| 4 | bx038583 | AT5G16600 | MYB | MYB043 | -0.38 | -0.69 | -2.43 |  |  |  |  |
| 2 | bx051906 | AT1G09540 | MYB | MYB061 | -2.81 | -2.57 | -2.74 |  |  |  |  |
| 3 | bx044766 | AT1G09540 | MYB | MYB061 | -2.54 | -2.82 | -3.31 |  |  |  |  |
| 0 | bx009363 | AT1G68320 | MYB | MYB062 | 5.33 | 5.60 | 5.26 |  |  |  |  |
| 0 | bx030547 | AT1G68320 | MYB | MYB062 | 4.55 | 4.79 | 4.82 |  |  |  |  |
| 0 | bx040066 | AT1G68320 | MYB | MYB062 | 2.76 | 4.41 | 2.77 |  |  |  |  |
| 4 | bx060467 | AT1G79180 | MYB | MYB063 | -0.50 | -0.40 | -2.99 |  |  |  |  |
| 2 | bx005420 | AT5G14750 | MYB | MYB066 | -4.29 | -3.34 | -1.90 |  |  |  |  |
| 2 | bx066574 | AT5G14750 | MYB | MYB066 | -4.14 | -3.47 | -1.85 |  |  |  |  |
| 1 | bx003997 | AT4G37260 | MYB | MYB073 | 5.20 | 4.68 | 3.45 |  |  |  |  |
| 4 | bx047449 | AT3G08500 | MYB | MYB083 | -1.61 | -2.28 | -4.67 |  |  |  |  |
| 3 | bx001355 | AT5G26660 | MYB | MYB086 | -1.13 | -2.79 | -2.90 |  |  |  |  |
| 3 | bx005456 | AT1G34670 | MYB | MYB093 | -0.72 | -2.44 | -2.51 |  |  |  |  |
| 1 | bx043069 | AT3G01140 | MYB | MYB106 | 2.43 | 0.38 | -0.23 |  |  |  |  |
| 0 | bx061804 | AT3G06490 | MYB | MYB108 | 1.34 | 3.97 | 4.18 |  |  |  |  |
| 0 | bx075632 | AT3G06490 | MYB | MYB108 | 3.31 | 4.73 | 4.67 |  |  |  |  |
| 0 | bx027904 | AT1G26780 | MYB | MYB117 | 2.43 | 3.56 | 3.35 |  |  |  |  |
| 0 | bx050295 | AT1G26780 | MYB | MYB117 | 2.63 | 3.70 | 3.49 |  |  |  |  |
| 1 | bx022254 | AT1G26780 | MYB | MYB117 | 3.23 | 3.27 | 2.33 |  |  |  |  |
| 0 | bx047540 | AT3G30210 | MYB | MYB121 | 2.98 | 2.93 | 3.51 |  |  |  |  |
| 0 | bx060440 | AT5G35550 | MYB | MYB123 | 1.36 | 3.70 | 4.75 |  |  |  |  |
| 2 | bx027839 | AT5G56840 | MYB | myb-like transcription factor family protein | -2.89 | -2.49 | -1.32 |  |  |  |  |
| 3 | bx035923 | AT5G56840 | MYB | myb-like transcription factor family protein | -2.28 | -3.47 | -4.92 |  |  |  |  |
| 5 | bx020137 | AT2G46410 | MYB | R3-type MYB transcription factor | -2.54 | -1.89 | -0.26 |  |  |  |  |
| 2 | bx025228 | AT2G18328 | MYB | RAD-like 4 | -2.11 | -2.59 | -2.07 |  |  |  |  |
| 3 | bx048219 | AT1G19510 | MYB | RAD-like 5 | -0.98 | -2.74 | -3.42 |  |  |  |  |
| 0 | bx013143 | AT1G75250 | MYB | RAD-like 6 | 1.08 | 2.53 | 1.70 |  |  |  |  |
| 0 | bx051436 | AT1G75250 | MYB | RAD-like 6 | 1.36 | 2.71 | 2.09 |  |  |  |  |
| 5 | bx033670 | AT1G01380 | MYB | Homeodomain-like superfamily protein | -2.48 | -1.85 | -0.25 |  |  |  |  |
| 5 | bx036948 | AT1G01380 | MYB | Homeodomain-like superfamily protein | -2.53 | -1.84 | -0.25 |  |  |  |  |
|  |  |  |  | *MYB No from TAIR (http://www.arabidopsis.org/index.jsp). | | |  |  |  |  |  |
|  |  |  |  |  |  |  |  |  |  |  |  |
| 0 | bx038862 | AT1G69490 | NAC | ANAC029 | 1.24 | 2.30 | 3.09 |  |  |  |  |
| 0 | bx074452 | AT1G69490 | NAC | ANAC029 | 1.12 | 2.19 | 2.97 |  |  |  |  |
| 1 | bx024871 | AT2G17040 | NAC | ANAC036 | 2.97 | 3.35 | 1.88 |  |  |  |  |
| 1 | bx071860 | AT5G22380 | NAC | ANAC090 | 3.52 | 1.96 | 0.51 |  |  |  |  |
| 3 | bx064839 | AT5G22380 | NAC | ANAC090 | -0.29 | -1.86 | -2.55 |  |  |  |  |
| 4 | bx009169 | AT2G46770 | NAC | ANAC043 (NST1) | 1.12 | -2.46 | -2.68 |  |  |  |  |
| 4 | bx029117 | AT2G46770 | NAC | ANAC043 (NST1) | -1.35 | -2.06 | -4.05 |  |  |  |  |
| 4 | bx003373 | AT4G28500 | NAC | ANAC073 (SND2) | -1.57 | -0.69 | -3.11 |  |  |  |  |
| 4 | bx041479 | AT4G28500 | NAC | ANAC073 (SND2) | -1.53 | -0.91 | -4.57 |  |  |  |  |
| 4 | bx065762 | AT4G28500 | NAC | ANAC073 (SND2) | -1.45 | -1.10 | -4.37 |  |  |  |  |
| 4 | bx065953 | AT4G28500 | NAC | ANAC073 (SND2) | -1.26 | -0.46 | -3.48 |  |  |  |  |
| 5 | bx026186 | AT3G15510 | NAC | ANAC056 | -2.75 | -1.62 | -1.52 |  |  |  |  |
| 5 | bx036393 | AT3G15510 | NAC | ANAC056 | -2.41 | -1.41 | -1.39 |  |  |  |  |
| 5 | bx040482 | AT3G15510 | NAC | ANAC056 | -3.06 | -0.89 | -2.12 |  |  |  |  |
| 5 | bx052893 | AT3G15510 | NAC | ANAC056 | -2.54 | -1.45 | -1.36 |  |  |  |  |
| 5 | bx070347 | AT3G15510 | NAC | ANAC056 | -2.45 | -1.38 | -1.32 |  |  |  |  |
|  |  |  |  | *ANAC No from TAIR (http://www.arabidopsis.org/index.jsp). | | | |  |  |  |  |
|  |  |  |  |  |  |  |  |  |  |  |  |
| 2 | bx081113 | AT5G04820 | OFP | ARABIDOPSIS THALIANA OVATE FAMILY PROTEIN 13, ovate family protein 13 | -2.47 | -1.94 | -1.54 |  |  |  |  |
| 3 | bx075992 | AT1G05420 | OFP | ARABIDOPSIS THALIANA OVATE FAMILY PROTEIN 12, ovate family protein 12 | -1.88 | -2.10 | -3.55 |  |  |  |  |
| 3 | bx073868 | AT1G06920 | OFP | ARABIDOPSIS THALIANA OVATE FAMILY PROTEIN 4, ovate family protein 4 | -2.40 | -2.62 | -3.08 |  |  |  |  |
| 3 | bx064169 | AT2G30395 | OFP | ovate family protein 17 | -2.43 | -3.27 | -4.64 |  |  |  |  |
| 3 | bx006281 | AT2G30400 | OFP | ARABIDOPSIS THALIANA OVATE FAMILY PROTEIN 2, ovate family protein 2 | -1.91 | -3.20 | -2.70 |  |  |  |  |
| 4 | bx046427 | AT2G36026 | OFP | Ovate family protein | -1.60 | -2.06 | -3.92 |  |  |  |  |
|  |  |  |  |  |  |  |  |  |  |  |  |
| 2 | bx006968 | AT2G42200 | SBP | squamosa promoter binding protein-like 9 | -3.01 | -4.07 | -2.90 |  |  |  |  |
| 2 | bx007319 | AT2G42200 | SBP | squamosa promoter binding protein-like 9 | -4.22 | -3.24 | -3.64 |  |  |  |  |
| 2 | bx007534 | AT5G50570 | SBP | SQUAMOSA PROMOTER-BINDING PROTEIN LIKE 13A, SQUAMOSA PROMOTER-BINDING PROTEIN LIKE 13, Squamosa promoter-binding protein-like (SBP domain) transcription factor family protein | -3.73 | -3.68 | -3.91 |  |  |  |  |
|  |  |  |  |  |  |  |  |  |  |  |  |
| 1 | bx006074 | AT2G37000 | TCP | TCP family transcription factor | 2.31 | 2.22 | 1.22 |  |  |  |  |
| 5 | bx038689 | AT3G15030 | TCP | maternal effect embryo arrest 35, TCP family transcription factor 4 | -2.43 | -0.57 | -2.01 |  |  |  |  |
|  |  |  |  |  |  |  |  |  |  |  |  |
| 2 | bx029904 | AT3G54390 | Trihelix | sequence-specific DNA binding transcription factors | -2.45 | -1.41 | -1.73 |  |  |  |  |
|  |  |  |  |  |  |  |  |  |  |  |  |
| 3 | bx033010 | AT1G47270 | TUB | tubby like protein 6 | -2.08 | -2.33 | -2.71 |  |  |  |  |
|  |  |  |  |  |  |  |  |  |  |  |  |
| 0 | bx007583 | AT1G13960 | WRKY | WRKY4 | 2.95 | 3.80 | 2.79 |  |  |  |  |
| 0 | bx005246 | AT1G29860 | WRKY | WRKY71 | 1.40 | 1.98 | 2.70 |  |  |  |  |
| 0 | bx049005 | AT1G29860 | WRKY | WRKY71 | 0.80 | 2.42 | 1.26 |  |  |  |  |
| 0 | bx051016 | AT1G29860 | WRKY | WRKY71 | 1.08 | 1.72 | 2.45 |  |  |  |  |
| 0 | bx008303 | AT1G62300 | WRKY | WRKY6 | 2.62 | 2.78 | 2.68 |  |  |  |  |
| 0 | bx077810 | AT4G22070 | WRKY | WRKY31 | 1.79 | 3.12 | 3.73 |  |  |  |  |
| 0 | bx006204 | AT5G13080 | WRKY | WRKY75 | 5.38 | 6.97 | 6.83 |  |  |  |  |
| 0 | bx046970 | AT5G13080 | WRKY | WRKY75 | 4.74 | 4.91 | 4.52 |  |  |  |  |
| 0 | bx054083 | AT5G13080 | WRKY | WRKY75 | 6.34 | 7.10 | 6.22 |  |  |  |  |
| 0 | bx061693 | AT5G13080 | WRKY | WRKY75 | 5.31 | 5.97 | 5.82 |  |  |  |  |
| 1 | bx042142 | AT1G62300 | WRKY | WRKY6 | 2.41 | 1.88 | 1.58 |  |  |  |  |
| 1 | bx023458 | AT1G80840 | WRKY | WRKY40 | 2.95 | 3.67 | -0.47 |  |  |  |  |
| 1 | bx038227 | AT1G80840 | WRKY | WRKY40 | 5.96 | 4.82 | 3.37 |  |  |  |  |
| 1 | bx047918 | AT1G80840 | WRKY | WRKY40 | 3.78 | 2.80 | 0.65 |  |  |  |  |
| 1 | bx059684 | AT1G80840 | WRKY | WRKY40 | 3.83 | 3.77 | 1.45 |  |  |  |  |
| 1 | bx029622 | AT2G30250 | WRKY | WRKY25 | 2.85 | 2.31 | 1.53 |  |  |  |  |
| 1 | bx026110 | AT2G38470 | WRKY | WRKY33 | 3.80 | 3.35 | 0.50 |  |  |  |  |
| 1 | bx026599 | AT2G38470 | WRKY | WRKY33 | 2.81 | 2.34 | 1.11 |  |  |  |  |
| 1 | bx050263 | AT2G38470 | WRKY | WRKY33 | 2.76 | 2.81 | 1.96 |  |  |  |  |
| 1 | bx066153 | AT2G38470 | WRKY | WRKY33 | 3.58 | 2.95 | 0.15 |  |  |  |  |
| 1 | bx002887 | AT3G56400 | WRKY | WRKY70 | 2.15 | 2.95 | -0.09 |  |  |  |  |
| 1 | bx023296 | AT3G56400 | WRKY | WRKY70 | 2.02 | 2.74 | -0.36 |  |  |  |  |
| 1 | bx026667 | AT4G11070 | WRKY | WRKY41 | 2.80 | 3.67 | 0.27 |  |  |  |  |
| 1 | bx062508 | AT4G23810 | WRKY | WRKY53 | 2.84 | 2.87 | 0.01 |  |  |  |  |
| 1 | bx081907 | AT4G31800 | WRKY | WRKY18 | 3.81 | 3.79 | 1.60 |  |  |  |  |
| 1 | bx032062 | AT5G64810 | WRKY | WRKY51 | 4.48 | 4.55 | 1.34 |  |  |  |  |
| 2 | bx009275 | AT5G28650 | WRKY | WRKY74 | -2.78 | -1.44 | -2.05 |  |  |  |  |
| 2 | bx065502 | AT5G43290 | WRKY | WRKY49 | -4.24 | -2.96 | -4.20 |  |  |  |  |
|  |  |  |  | *ANAC No from TAIR (http://www.arabidopsis.org/index.jsp). | | | |  |  |  |  |
|  |  |  |  |  |  |  |  |  |  |  |  |
| 3 | bx040512 | AT1G75240 | ZF_HD | zinc-finger homeodomain 5, homeobox protein 33 | -1.79 | -3.69 | -3.79 |  |  |  |  |
|  |  |  |  |  |  |  |  |  |  |  |  |
| 0 | bx064707 | AT1G19180 | ZIM | jasmonate-zim-domain protein 1 | 3.37 | 5.16 | 3.93 |  |  |  |  |
| 1 | bx022164 | AT1G30135 | ZIM | jasmonate-zim-domain protein 8 | 2.44 | 2.41 | 1.72 |  |  |  |  |
| 1 | bx033017 | AT5G13220 | ZIM | TIFY DOMAIN PROTEIN 9, JASMONATE-ASSOCIATED 1, jasmonate-zim-domain protein 10 | 2.81 | 1.46 | 0.01 |  |  |  |  |
